# The Diet Guidelines: 3 Diets (DG3D) study protocol of a behavioral teaching kitchen intervention for type-2 diabetes prevention among African American adults

**DOI:** 10.1016/j.cct.2025.108109

**Published:** 2025-10-10

**Authors:** Shiba Bailey, Gabrielle M. Turner-McGrievy, Enid A. Keseko, Taylor Duncan, Denine Ward-Johnson, Briana Davis, Sara Wilcox, Daniela B. Friedman, Mark A. Sarzynski, Angela D. Liese

**Affiliations:** a Prevention Research Center, Arnold School of Public Health, University of South Carolina, 921 Assembly Street, Columbia, SC 29208, United States of America; b Department of Health Promotion, Education, and Behavior, Arnold School of Public Health, University of South Carolina, 915 Greene Street, Columbia, SC 29208, USA; c Department of Exercise Science, Arnold School of Public Health, University of South Carolina, 921 Assembly Street, Columbia, SC 29208, United States of America; d Department of Epidemiology and Biostatistics, Arnold School of Public Health, University of South Carolina, 915 Greene Street, Columbia, SC 29208, USA

**Keywords:** African American, Dietary guidelines, Type 2 diabetes prevention, Obesity, Randomized controlled trial, Healthy eating index, Teaching kitchen intervention

## Abstract

**Background:**

The U.S. Dietary Guidelines (USDG) serve as a foundational public health resource, yet their cultural applicability to underserved populations, including Black and African American (AA) adults, remains limited. The AA population is disproportionately affected by obesity and type 2 diabetes mellitus (T2DM), highlighting the need for culturally tailored interventions.

**Objectives:**

To describe the design and methodology of the Dietary Guidelines: 3 Diets (DG3D) study, aimed at evaluating the effectiveness of culturally adapted USDG dietary patterns in improving diet quality and reducing risk factors associated with T2DM among AA adults. The intervention focuses on nutritional education and the promotion of healthy eating behaviors to support sustainable dietary change.

**Methods:**

The DG3D study is a 12-month, single-masked, three-arm randomized behavioral nutrition intervention designed to evaluate three culturally adapted USDG dietary patterns: Healthy US, Mediterranean, and Vegetarian. The study recruited AA adults with overweight or obesity and at least three additional risk factors for T2DM. Participants were randomized to one of three dietary patterns and received group-based classes, cooking demonstrations, and web-based educational content. Primary outcomes include changes in diet quality (Healthy Eating Index [HEI]), body weight, and hemoglobin A1c (HbA1c), assessed at baseline, 6 months, and 12 months.

**Conclusion:**

The DG3D study is a novel and comprehensive randomized trial evaluating culturally tailored dietary patterns in the AA population, a high-risk population. Findings are expected to inform inclusive dietary policy and contribute to reducing health disparities related to obesity and T2DM.

Trial registration: ClinicalTrials.gov identifier NCT05254496

## 1. Introduction

The United States Dietary Guidelines (USDG) serve as a critical framework for the development of nutrition policies and programs aimed at enhancing public health outcomes across the U.S. [1] Jointly established by the U.S. Department of Agriculture (USDA) and the U.S. Department of Health and Human Services (USDHHS), these guidelines provide evidence-based recommendations to improve dietary habits and prevent chronic diseases [1]. These guidelines inform national programs such as the National School Lunch Program and the Supplemental Nutrition Assistance Program (SNAP) [1]. By promoting healthy eating including the consumption of fruits, vegetables, whole grains, and lean proteins, the USDG promote a culture of health and wellness nationwide [1].

Though intended for all Americans [1,2], the research informing the USDG has largely involved White populations [3], raising concerns about their relevance for racial and ethnic minorities, who often have distinct cultural food practices, health risks, and dietary behaviors [4,5].

Adults identifying as Black and African American (AA) exhibit higher rates of obesity compared to their White and Hispanic counterparts [5]. Overweight and obesity are associated with a range of chronic diseases, including type 2 diabetes (T2DM) [6]. T2DM affects over 11 % of the U. S. population [7], and accounts for approximately $412.9 billion in annual economic costs [8]. AA individuals are 60 % more likely to be diagnosed with T2DM than non-Hispanic Whites, [9] yet remain underrepresented in prevention strategies. Previous trials, including the Diabetes Prevention Program (DPP) [10,11] and Look AHEAD [12], show lifestyle changes can reduce T2DM incidence, these interventions were not tailored to AA populations. This study addresses that gap by testing three culturally adapted dietary patterns in a randomized controlled trial among AA adults at risk for T2DM. Diet quality is a key modifiable factor in the development of both obesity and diabetes [13].

The 2020–2025 USDG highlight three examples of healthy dietary patterns: the Healthy U.S.-Style Pattern (Healthy US), the Healthy Mediterranean-Style Pattern (Mediterranean), and the Healthy Vegetarian Pattern (Vegetarian) [2]. Healthy US includes all food groups with an emphasis on nutrient-dense, low-saturated-fat options [2]. The Mediterranean diet promotes fruits, vegetables, legumes, whole grains, and healthy oils, with moderate animal product intake [2]. The Vegetarian diet includes eggs and dairy but excludes meat and seafood, favoring soy products, legumes, nuts, and whole grains [2]. However, few studies have evaluated how these patterns influence T2DM risk in AA populations [3]. Most USDA evidence is observational rather than community-based and randomized [3]. Additionally, cultural adaptations of these dietary patterns have been limited. The Dietary Guidelines: 3 Diet Patterns (DG3D) study was developed to address this gap through a 12-month randomized behavioral nutrition intervention targeting AA adults at risk for T2DM.

Dietary disparities among AA adults are shaped by multiple factors, including food deserts, limited healthcare access, cultural norms around body size, and environmental challenges [14] Structural conditions such as residential segregation, the overconcentration of fast-food outlets, and limited access to safe physical activity spaces exacerbate these issues [14,15]. These barriers contribute to unhealthy dietary patterns and higher consumption of calorie-dense, nutrient-poor foods [14,16].

Increased intake of added sugars, refined grains, and processed oils is linked to obesity and related health outcomes [17,18]. Populations of African ancestry, including AA individuals, may have distinct biological responses to these dietary changes [18]. Genetic factors may influence dietary effects, and AA individuals may derive greater benefit from plant-based diets than White adults [18,19]. Among AA populations, those following vegetarian or vegan diets report lower rates of hyper-tension, T2DM, and high LDL cholesterol compared to meat or fish eaters [4,20].

## 2. Objectives and hypothesis

The primary objective of this trail is to evaluate the impact of three culturally adapted dietary patterns, Healthy US, Mediterranean, and Vegetarian on diet quality and body weight among AA adults.

We hypothesized that when examining culturally adapted versions of the three dietary patterns (Healthy US, Mediterranean, and Vegetarian), that there would be greater improvements in diet quality (Healthy Eating Index score (HEI)) and body weight in the plant-based diet groups (Vegetarian and Mediterranean) as compared to the Healthy US diet group.

This manuscript describes the study protocol, design, and implementation of the DG3D dietary intervention, including the rational to culturally adapting the diets and evaluation methods used in the randomized controlled trial.

## 3. Methods

### 3.1. Study design and setting

**Fig. 1.**
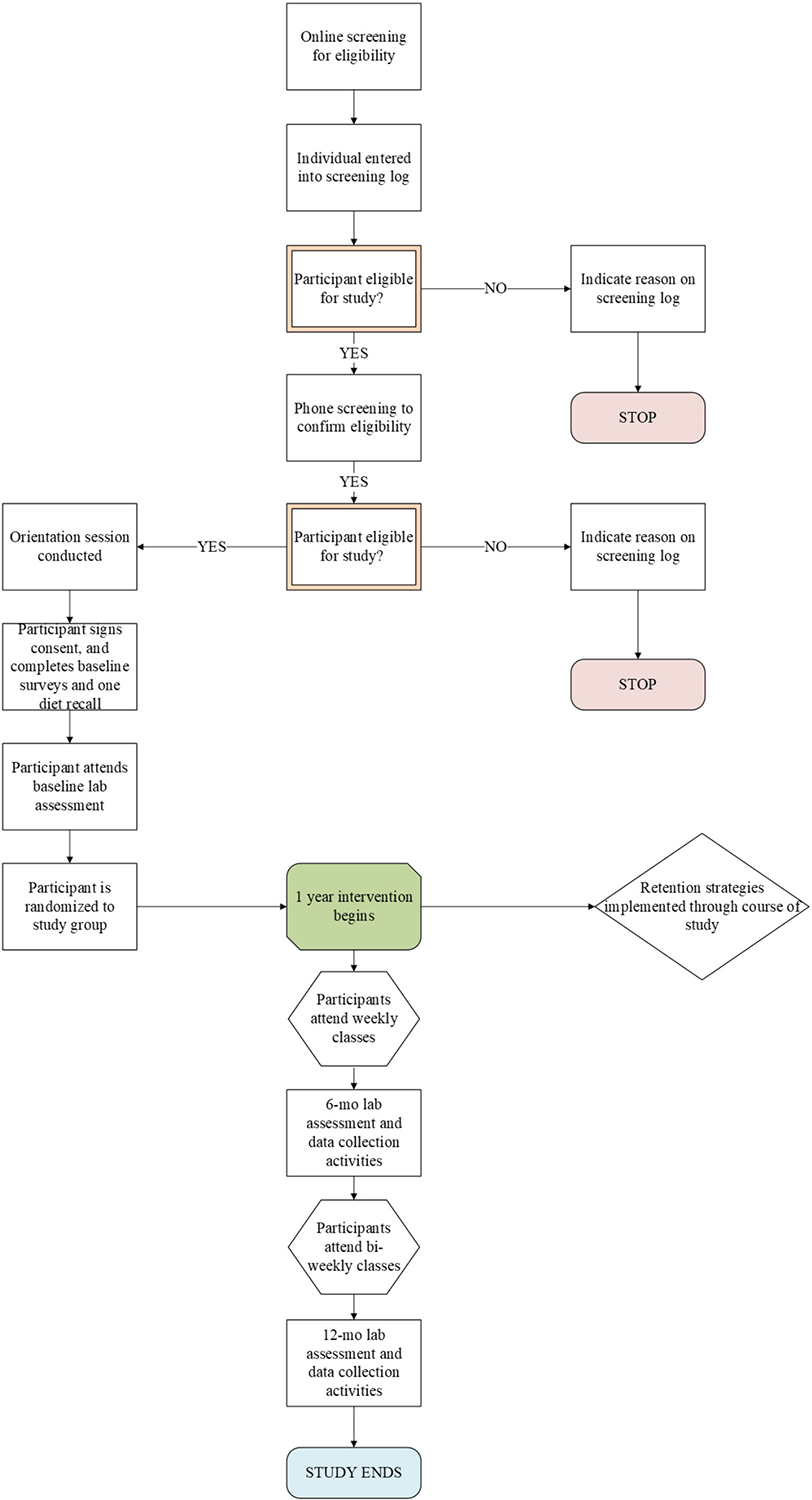
Trial flow diagram of the DG3D intervention.

The DG3D study was conducted in the southeastern U.S. at an academic teaching kitchen or remotely via Zoom (additional details are provided in Section 5). The randomized three-arm behavioral nutrition intervention compared the three USDG dietary patterns among AA adults. The study had two phases. Phase one was a 12-week formative intervention where all material was derived from USDG-related resources, with no cultural tailoring. Participants in phase one then completed focus groups to provide feedback on the intervention content and recipes which was used to refine the intervention content in order to deliver the 12-month trial. Phase one has been described elsewhere [21]. Phase two, which was the 12-month trial is described below (Fig. 1).

The DG3D study was conducted in three cohorts, with a recruitment goal of 198 participants. Cohort 1 began in March 2022, Cohort 2 in March 2023, and Cohort 3 in August 2024. Cohorts were separated to ensure sufficient recruitment, manage space constraints of our demonstration kitchen and teaching classroom, and secure appointments for laboratory assessments.

### 3.2. Informed consent

Before enrollment, prospective participants attended a virtual orientation via Zoom, where the study coordinator explained procedures, dietary patterns, sample meals, and classes. Informed consent was obtained electronically through REDCap. The study was approved by the Institutional Review Board of the University of South Carolina (approval number: IRB Pro00118661).

### 3.3. Recruitment and enrollment

**Fig. 2.**
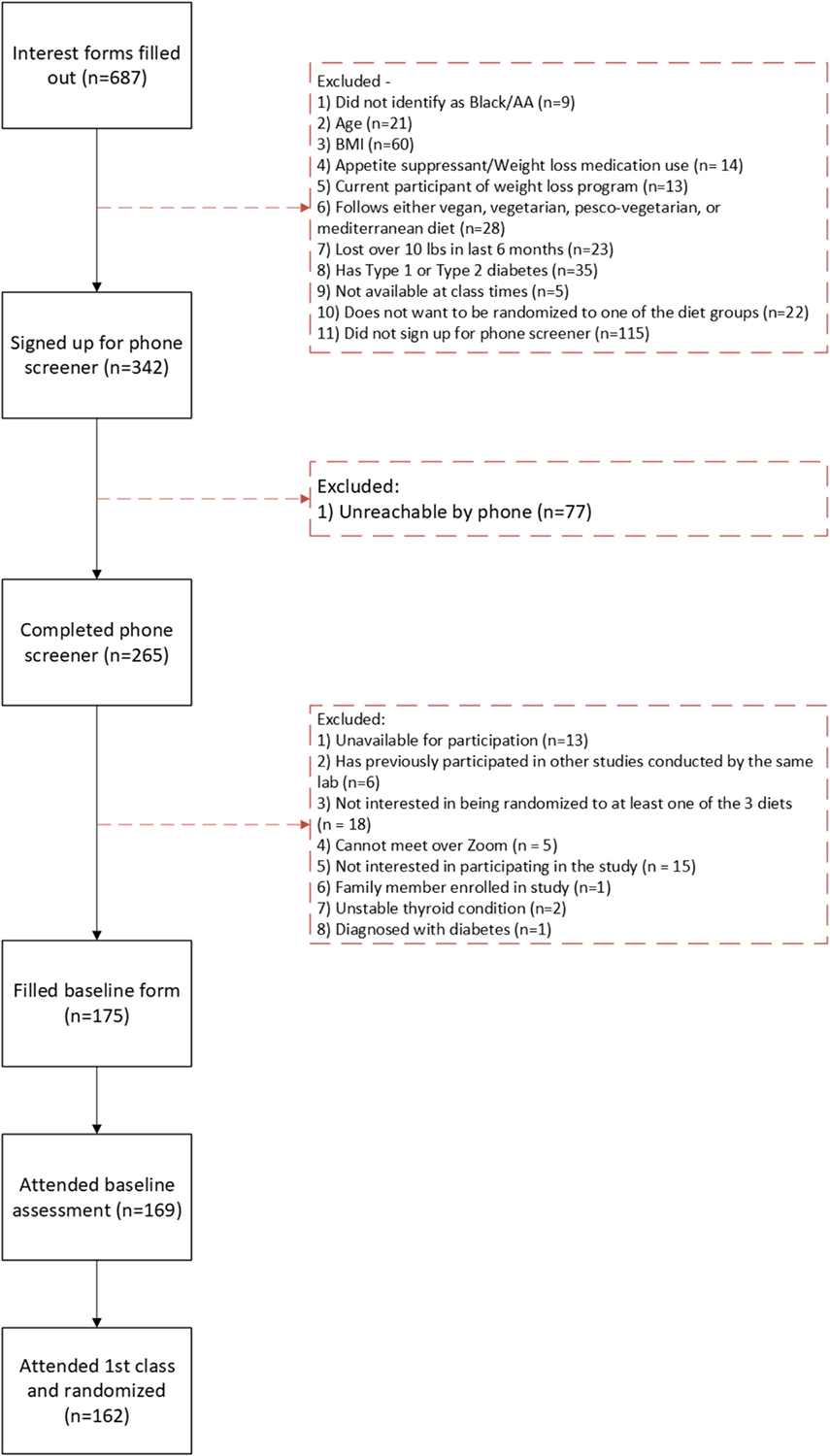
Participant screening and enrollment flowchart for DG3D intervention.

Recruitment was conducted via television, radio, billboards, newspapers, social media, word of mouth, and community events. Culturally tailored strategies targeted African American communities through outreach at churches, barbershops, beauty salons, fraternities, sororities, clinics, and community centers. Interested individuals completed an online survey; those eligible were phone-screened and, if further eligible, invited to an orientation session outlining study procedures, expectations, and timelines. Participants who chose to enroll completed a baseline survey, an ASA-24 dietary recall [22], scheduled a baseline laboratory assessment, and provided informed consent, as previously described (Fig. 2). Randomization and class start dates were as follows: Cohort 1 participants were randomized between March 30 and April 4, 2022, with the first class held on April 4, 2022; Cohort 2 participants were randomized between May 11 and June 1, 2023, with the first class on June 5, 2023; and Cohort 3 participants were randomized between September 11 and September 23, 2024, and the first class was held on September 16, 2024.

### 3.4. Eligibility criteria

Adults with obesity or overweight (BMI 25–49.9 kg/m^2^), who had not been diagnosed with T2DM at the time of recruitment but had ≥3 risk factors for T2DM [23] and who self-identified as AA, were randomized to one of three dietary patterns as presented in the USDG: Healthy US, Mediterranean, or Vegetarian. Table 1 presents the eligibility criteria for participation.

### 3.5. Randomization procedures

Participants who completed all baseline assessments were randomly assigned to one of three dietary patterns. Randomization, stratified by sex and conducted in blocks of nine, ensured balanced group distribution as participants completed assessments on a rolling basis. Allocation sequences were computer-generated by the study statistician. A masked research assistant assigned participants to groups based on these sequences.

### 3.6. Single-masked

The study was a single-masked study. We could not conduct a double-masked study since participants could not be masked to their assigned diet. Study staff involved in randomization and those conducting assessments were masked to participants’ diet assignments. Additionally, participants were instructed not to share assignment details with assessment staff.

### 3.7. Intervention

The intervention was designed to test the impact of culturally tailored USGD dietary patterns on quality of dietary intake and T2DM risk factors among AA adults. The DG3D study consisted of 3 components: 1) group-based classes, 2) remotely delivered content, and 3) intervention diets.

#### 3.7.1. Group-based classes

The intervention included weekly in-person classes at the University of South Carolina’s teaching kitchen, during the first six months, followed by bi-weekly sessions in the final six months. Participants were required to be available to attend classes at all three possible meeting times since participants would be randomized to either class: Mondays from 5:30 pm to 6:45 pm, or 7:00 pm to 8:15 pm, or Tuesdays from 5:30 pm to 6:45 pm. Classes were led by AA identifying staff including a nutrition instructor and a graduate assistant (Registered Dietitian Nutritionist) and featured cooking demonstrations with food samples. To broaden recruitment efforts and accommodate requests to participate remotely, Cohort 3 classes were delivered virtually via Zoom (see Section 5).

#### 3.7.2. Remotely delivered content

During the first six months, participants received face-to-face instruction and had access to weekly class content via the participant website. After six months, classes transitioned from weekly to biweekly meetings. During off-weeks, participants received newsletters, monthly Lunch-n-Learns hosted by staff via Zoom [24], and educational videos. All materials were accessible through the participant website.

#### 3.7.3. Intervention diets and class structure

Recipes from MyPlate [25] formed the basis of the recommendations but were adjusted based on feedback from phase one of the study [21]. All three diets and intervention material were revised to be culturally relevant to the AA population (see Supplementary files).

The weekly session topics were designed by a Registered Dietician using behavioral strategies from the Diabetes Prevention Program (DPP) and guided by the Social Cognitive Theory (SCT) [10,26]. DPP informed content included modules on relapse prevention, stress management, staying on track, and strategies for maintaining motivation. SCT constructs such as self-efficacy, behavioral capability, self-regulation, and reinforcement were embedded in the sessions (Table 2).

A 12-month duration was selected to support sustained behavior change, habit formation, and long-term adherence. This time frame aligns with evidence showing that shorter interventions often fail to maintain outcomes. It reflects best-practice standards in behavioral nutrition research [27,28].

## 4. Data

### 4.1. Assessment protocol

Assessments were conducted at baseline, 6 months, and 12 months at the Clinical Exercise Research Center (CERC), University of South Carolina. This section details the measurement of primary outcomes (diet quality, weight loss, HbA1c) and secondary outcomes (e.g., hip/waist circumference, BP). Participants also completed surveys and dietary recalls. While protocols for Cohorts 1 and 2 were consistent, modifications for Cohort 3 are described in Section 5.

#### 4.1.1. Dietary assessment

Dietary intake was assessed using the National Cancer Institute’s Automated Self-administered 24-h recall (ASA24) [22]. At each assessment period, participants completed three unannounced recalls (two on weekdays and one on a weekend day) within a three-week window. The ASA24 system was accessible online. For participants with limited digital proficiency or access, recalls were administered via telephone by trained interviewers or conducted in person at the university’s computer lab. All participants received in-person training on ASA24 during orientation and completed their initial recall under supervision; subsequent recalls were self-administered. The system allowed completion until 11:59 p.m. on the assigned day. If a recall was missed, it was rescheduled for a different, non-consecutive day. Reminders were issued via text message, email, and phone. Healthy Eating Index (HEI) scores were calculated using the ASA24 SAS code for HEI 2015 [29].

#### 4.1.2. Anthropometric measures

Height was measured at baseline using a calibrated wall-mounted stadiometer (Model S100, Ayerton Corp., Prior Lake, MN). Body weight was recorded at each assessment period with a calibrated digital scale (Healthometer^®^ model 500 KL, McCook, IL). Waist circumference was measured at the level of the iliac crest using a spring-loaded tape [30], and hip circumference was recorded at the fullest part of the buttocks [31]. Two measurements were taken for each, and if they differed by more than 4 mm, a third was obtained. The two readings within a 4 mm difference were used and averaged for analysis. Protocol modifications for Cohort 3 are detailed in Section 5.

#### 4.1.3. Blood pressure (BP)

Following a 5-min rest period, BP was measured with an Omron Hem 705 CP Auto Inflate Blood Pressure Monitor. At least two readings were taken, with up to four possible, and the average of these readings was calculated. If the difference between the first and second readings exceeded 5 mmHg, additional readings were taken, and the average of all the readings was used. BP was the first measurement conducted during the lab assessment to ensure that readings were not affected by potential inaccuracies due to the stress or discomfort caused by the finger stick required for testing HbA1c [32]. Modifications to Cohort 3 are detailed in Section 5 of this paper.

#### 4.1.4. Blood samples and analysis – HbA1c

HbA1c was assessed using the DCA Vantage Analyzer^™^. Whole blood was obtained by finger stick and placed directly into a glass capillary for analysis using the reagent cartridges. Participants were not expected to fast before the assessment. The assay determined both HbA1c concentration and total hemoglobin levels, and the results were expressed as the percentage of HbA1c. The DCA Vantage Analyzer automatically performs all measurements and calculations, providing results within a few minutes [33]. The adjustments to Cohort 3 are outlined in Section 5 of this paper.

#### 4.1.5. Psychosocial questionnaires

Participants completed surveys at baseline, 6- and 12-months (Table 3). These surveys collected demographic variables, socioeconomic factors (such as education, income, housing situation, etc.), use of nutrition assistance programs [34], household composition, and environmental changes (e.g. changes in neighborhood, housing, food access) [35,36]. The surveys also assessed dietary self-efficacy [37], current medications and any changes, dietary acceptability [38–40], perceived stress [41], food choice [42], and measurements of social influences on health behavior [43], perceptions of food spending [44], health-related quality of life [45], food insecurity [46], physical activity [47], and factors related to dietary restraint, disinhibition, and hunger [48], as well as appetite for palatable foods [49]. Participants completed the International Physical Activity Questionnaire – Short Form (IPAQ) [47] which provides a standardized approach for gathering physical activity data [42,43,47].

### 4.2. Data safety and monitoring

The funding organization required the establishment of a Data Safety and Monitoring Plan (DSMP) and Board (DSMB). This board, composed of three NIH-funded researchers from the same institution who are not involved in the study, was responsible for overseeing the trial. Every six months, the DSMB received a detailed report from the study team that included information on participant recruitment, the status of enrolled individuals, adherence to the intervention, and any adverse events. Serious adverse events were reported immediately to both the institution’s Institutional Review Board (IRB) and the DSMB. The DSMB’s role was to review the interim data and analyses to safeguard the safety, rights, and well-being of the participants. In addition to email reports, the DSMB held an annual in-person meeting where the chair prepared a confidential report advising the Principal Investigator (PI) on the continuation of the trial.

### 4.3. Community advisory board

A community advisory board (CAB) was assembled with members from local health, religious, and nutrition organizations. Originally composed of five members, the CAB had three active members by the end of the study. Members were selected for their community engagement experience to support recruitment and retention efforts. To improve male participation, some were chosen for their influence in the AA male community. The CAB advised on recruitment, retention, and intervention delivery, met quarterly, and received study updates. Members were compensated for their time.

### 4.4. Process evaluation

The DG3D study conducted a process evaluation to assess reach, fidelity to the intended implementation, and participants’ self-reported feedback, satisfaction, and behavior. Guided by the framework of Saunders et al. [50], the evaluation examined reach, attendance, participation, dosage, treatment fidelity, and overall compatibility and satisfaction. Data were collected before, during, and after the intervention.

An online database enabled the study team to monitor class attendance, intervention material usage, participant contact (e.g., appointment reminders), and completion of make-up classes. Treatment fidelity was periodically assessed through unannounced observations by team members who were not involved in intervention delivery. These observations evaluated aspects such as class start and end times, adherence to class structure, and execution of planned demonstrations. A standardized checklist documented fidelity metrics across diet-specific classes within each cohort.

### 4.5. Adherence outcomes

Adherence will be assessed via class attendance (intervention dose) and ASA24 dietary recalls. Methods for evaluating dietary adherence are under development. Adherence outcomes will be reported upon completion of data collection.

## 5. Changes to Cohort 3

A number of modifications were made to the DG3D intervention and its delivery for Cohort 3 based on feedback from participants in previous cohorts and observations made by study staff. In addition, while Cohort 1 met recruitment goals, Cohort 2 fell short, and recruitment for Cohort 3 was even more challenging. To assist with recruitment and retention (and based on feedback from Cohorts 1 and 2), the names of the diets were revised. “Vegetarian” was renamed “Plant-Powered” to emphasize the use of plant-based foods in this diet. “Healthy US” was changed to “Nutrient Rich” to highlight nutrient-dense foods included in this diet. “Mediterranean” remained unchanged as it was already well recognized and understood by participants. Additionally, to broaden our recruitment reach and to address challenges regarding attendance seen in Cohorts 1 and 2 (childcare issues, transportation issues, or concerns about driving after dark), all classes were delivered virtually via Zoom.

To meet the required sample size, recruitment was expanded to include individuals aged 18 to 70 years (previously 18 to 65), and the study was extended to include residents from the greater Orangeburg, Sumter, Charleston, and Greenville, SC and Augusta, GA areas. To accommodate participants outside the Columbia, SC area or those unable to schedule an appointment with the onsite CERC lab, an outside lab (Labcorp) was contracted to conduct assessments.

### 5.1. Anthropometric measures at outside laboratory

Height was measured only at baseline using a calibrated wall-mounted stadiometer. Body weight was recorded at each assessment period with a calibrated digital scale. Waist circumference was measured with a disposable paper tape measure; hip circumference was not collected by Labcorp.

### 5.2. BP at outside laboratory

Following a 5-min rest period, BP was measured with an Omron Hem 705 CP Auto Inflate Monitor. At least two readings were taken, with up to four possible, and the average of these readings was calculated. If the difference between the first and second readings exceeded 5 mmHg, additional readings were taken and averaged. BP was measured first to avoid potential inaccuracies due to stress or discomfort caused by the finger stick during HbA1c testing.

### 5.3. Blood samples and analysis (HbA1c) at outside laboratory

HbA1c was assessed using the Roche Tina-quant^®^ methodology on a cobas^®^ Analyzer [51]. Whole blood was obtained by venipuncture and placed directly into a glass tube for analysis [51]. A minimum of 0.5 ml was required to successfully conduct the test and the results were expressed as the percentage of HbA1c [51,52].

## 6. Statistics and sample size

### 6.1. Sample size justification

We selected three primary outcomes, diet quality, body weight, and HbA1c, because these metrics collectively capture behavioral, physiological, and clinical changes associated with adherence to culturally adapted USDG dietary patterns. The study was powered to ensure sufficient statistical power to analyze change in the three main outcomes of HEI score, weight loss, and HbA1c over 12 months. Considering a 20 % attrition rate, the sample size for each diet group was 65 participants, resulting in an effective sample size of 52 per group. In a previous pilot study, a SD of 4.1 kg of weight change was observed across all arms of the study [53]. For the current study, a total difference of 4.6 kg across the three diets from a minimally important difference of 2.3 kg per diet would result in a 0.56 effect size and 88 % power. For a minimum important difference in HEI score of 5.2 per diet and 10.4 across diets, the effect sizes estimated were 0.58 and 1.16 respectively. With 52 participants, power achieved was 90 % arm-to-arm and 99 % when comparing best to worst diets. Additionally, effect sizes of 0.56 and 1.12, with 88 % and 99 % power, respectively, were achieved for clinically significant changes in HbA1c per diet and across diets. These estimates were generated based on results from previous studies [38,54,55].

### 6.2. Data collection

Survey data and anthropometric data were collected via REDCap [56]. The ASA24 dietary recall data were gathered from the ASA24 website, maintained by the National Institutes of Health [22].

### 6.3. Plan for primary analysis

Primary outcome analysis will begin after all the participants complete the intervention and assessments. Data will first be examined for outliers, normality, missingness, and loss to follow-up. Repeated measures mixed models will be fitted using maximum likelihood estimation and robust standard errors in SAS^®^ PROC MIXED. Separate models will assess T2DM risk factors (weight and HbA1c) and diet quality (HEI score) at each timepoint. Models will include a three-level time effect, a three-level intervention arm effect, and a two-way interaction between time and intervention arm, with the Healthy US group as the reference. Analyses will follow an intent-to-treat approach, utilizing all available data, including participants lost to follow-up.

## 7. Retention and withdrawal

Retention strategies included raffle incentives for attendance and birthday cards to foster engagement. One-on-one sessions were offered to participants needing additional support. Monthly Lunch-and-Learn sessions were held on the third Thursday with the study interventionist and registered dietitian.

Participants received reminders via text, email, and phone regarding classes, lab assessments, dietary recalls, and surveys. Missed classes prompted follow-up messages, directing participants to the study web-site to review class content and complete a brief survey confirming make-up participation. The study database logged all contacts, including mode, frequency, and message content.

## 8. Safety

All adverse events occurring during the study were reported to the DSMB. In the event of any adverse occurrences during laboratory assessments (e.g., elevated BP), the assessment was immediately discontinued for that participant, and they were advised to consult their physician.

## 9. Baseline characteristics of the sample population

Table 4 presents the baseline sociodemographic and clinical characteristics of participants by dietary group. The sample was predominantly female, with most holding college or advanced degrees. Employment status was comparable across groups, with 38–41 % reporting no current employment. The average participant age was approximately 50 years, and most were either single or married.

Clinical and dietary measures were non-significantly different between groups. The Mediterranean group had a slightly higher BMI (37.4 ± 7 kg/m^2^) than the Vegetarian group (35.1 ± 6 kg m^2^) and all groups exhibited BMI values above the national average for AA adults in the U. S. (31.3 kg/m^2^) [57]. HbA1c levels were consistent with values for the AA population at the national level [58].

Dietary intake also varied modestly. Energy intake was highest in the Mediterranean group (1955.8 ± 682 kcal/day) and lowest in the Vegetarian group (1808.0 ± 536 kcal/day), both below national averages for AA adults (2134.0 ± 50.7 kcal/day) [59]. Carbohydrate intake was slightly higher in the Healthy US and Mediterranean groups, but all groups were below the national estimate of 260.9 g/day [60]. HEI scores ranged from 54.8 to 57.1, consistent with prior data for AA adults [61].

## 10. Implications

The DG3D trial has the potential to inform culturally responsive dietary counseling and public health strategies for AA adults at high risk for T2DM. Findings may support the integration of culturally tailored dietary patterns into clinical practice and community-based prevention programs. This work can help address health disparities and promote equity in the application of U.S. dietary guidelines.

## 11. Conclusion

The DG3D study aims to inform future dietary guidelines with a focus on culturally tailored approaches for AA individuals. The findings from this randomized controlled trial have the potential to shape public health policies and community-based programs, contributing to the prevention of chronic diseases. Additionally, this research aligns the National Nutrition Research Roadmap [62], by advancing our understanding of eating patterns and identifying effective strategies to promote healthy dietary behaviors. Together, these initiatives aim to advance public health outcomes and create a more inclusive framework for dietary guidelines that effectively serve diverse populations.

## Supplementary Material

1

2

3

## Figures and Tables

**Table 1:** Inclusion and exclusion criteria for the DG3D study.

Inclusion Criteria	Exclusion Criteria
Self-identify as Black/African American	Currently following a vegan or vegetarian diet
Age 18–65 years (expanded to 70 years for Cohort 3)	Pregnant, recently pregnant (within 6 months), currently breastfeeding, or planning pregnancy within the next 24 months
BMI between 25 and 49.9 kg/m^2^	Currently participating in a weight loss program or taking weight loss medications (participants may be trying to lose weight on their own)
≥3 risk factors for T2DM (such as a family history of T2DM, being diagnosed with high bp [≥130/80 mmHg], and not physically active)	Recent weight loss >10 lbs. in the past 6 months Type 2 diabetes controlled with medications (vs. diet and exercise)
Live within or near Columbia, SC (Cohorts 1 and 2)	
Live within or near Columbia, Orangeburg, Sumter, Charleston, or Greenville (SC), or Augusta (GA) (Cohort 3)	Uncontrolled thyroid condition
Able to attend all monitoring visits	
Willing to be randomized to either diet	
Free of major health or psychiatric disease, and no drug or alcohol dependency	
Free of an eating disorder (screened using the Eating Disorder Screen for Primary Care [ESP]) [63]	
Available on the two meeting nights (e. g., Monday or Tuesday)	

**Table 2:** DG3D intervention sessions topics with corresponding DPP content and SCT constructs.

Class	Session Topic	DPP-Based Content	SCT Construct(s) Targeted
1	Welcome to DG3D: Your Diet Guidelines	Introduction to the program	Outcome expectations
2	Grocery Store Tour and label reading	Shop to prevent T2	Observational learning Self-efficacy
3	Meal Planning	Cook to prevent T2 Track your food	Behavioral capability Self-efficacy
4	Carbohydrates, fiber & grains	More about carbohydrates SMART Goal settings	Behavioral capability Self-efficacy & Outcome expectations
5	Dairy, Soy and Calcium Your Relationship with food	Have healthy food you enjoy	Behavioral capability Reinforcement
6	Protein Dining with family	Get support	Behavioral capability Social support Reciprocal determinism
7	Physical Activity	Get active to prevent T2	Self-efficacy
8	Fats (Nuts/Seeds) Negative Talkback	Take charge of your thoughts SMART Goals	Self-regulation Self-efficacy & Outcome expectations
9	Fruits	Get support	Social support
10	Vegetables Feeding kids and teens	Eat well to prevent T2	Outcome expectations Observational learning
11	Restaurants/Travel Social Cues	Eat well away from home	Reciprocal determinism Self-regulation
12	Water	SMART Goals	Self-efficacy & Outcome expectations
13	Emotional Eating	Coping with triggers	Self-regulation
14	Pre/Probiotics		Behavioral capability
15	Movie Night		Observational learning
16	Iron Response to film	SMART Goals	Behavioral capability Self-efficacy & Outcome expectations
17	Mind the salt! – sodium and potassium levels	Slippery Slope	Self-regulation
18	Recipe Redux Shopping Triggers	Coping with triggers	Self-regulation Reinforcement
19	Heart disease/Stroke/Cholesterol Removing barriers	Keep your heart healthy	Self-efficacy
20	Added sugars, fat	SMART Goals	Self-efficacy & Outcome expectations
21	Let’s revisit getting active	Get more active	Social support Reinforcement
22	Stress eating	Manage stress	Self-regulation
23	Quick meals Healthy snacks	Shop and cook to prevent T2	Behavioral capability
24	Being part of a Community Staying motivated	Stay motivated to prevent T2 SMART Goals	Reinforcement Social support Self-efficacy & Outcome expectations
25	Diabetes	More about T2 Slippery Slope pt. 2	Behavioral capability Relapse prevention
26	Cleanses/ detoxes Balancing thoughts for the long-term	Take charge of your thoughts	Self-regulation
27	Busting through weight loss plateaus	When Weight Loss Stalls	Self-efficacy Reinforcement
28	Holiday tips	Stay active away from home Have healthy food you enjoy SMART Goals	Self-regulation Self-efficacy & Outcome expectations
29	Local restaurant menu reading	Eat well away from home	Behavioral capability
30	Religion and Diet		Outcome expectations
31	Keto Diets	Get Back on Track	Relapse prevention
32	Health through heritage Sleep, anxiety, and weight loss	Get enough sleep SMART Goals	Self-efficacy & Outcome expectations
33	Take a fitness break		Reinforcement
34	Men’s health	Preventing relapse	Behavioral capability
35	Women’s health	Check in and keep going	Behavioral capability Social support
36	Looking back Moving forward	SMART Goals for the Long-Term Prevent T2 -for life	Self-efficacy & Outcome expectations Reinforcement
37	Program close	Prevent T2 for life	Reinforcement

**Table 3:** Study Timeline: Enrollment, intervention delivery, and assessment schedule across trial phases.

Timepoint	TRIAL PERIOD				
	Enrollment		Post-randomization		Close-out
	−ti to 0	0	t1 (Months0–6)	t2 (Months7–12)	t3 (12 mo)
**Enrollment:**					
Participant fills out interest form	X				
Phone screener	X				
Eligible participant attends orientation	X				
Informed consent	X				
Baseline assessments	X	X			
Randomization	X	X			
**Intervention:**					
DG3D Intervention begins		X	X	X	
Group-based classes					
Weekly classes (in-person/Zoom)			X		
Bi-weekly classes (in-person/Zoom)				X	
Remote content					
Weekly class content			X		
Bi-weekly class content				X	
Newsletters				X	
Monthly Lunch-n-Learns				X	
Educational videos				X	
**Assessments:**					
Questionnaire measures					
Demographic		X			
Information					
Perceived Stress Scale		X		X	X
Short Form Health		X		X	X
Survey (SF-12)					
Self-Efficacy for Diet		X		X	X
Behaviors					
U.S. Adult Food Security		X		X	X
Survey Module					
Food Spending Items		X		X	X
Three-Factor Eating		X		X	X
Questionnaire					
Power of Food Scale		X		X	X
Dietary Intake (ASA-24 recalls)		X		X	X
Internation Physical		X		X	X
Activity Questionnaire					
(IPAQ) - short version					
Current Medications		X		X	X
Dietary Adherence and Acceptability		X		X	X
Lab-based assessments					
Height		X			
Weight		X		X	X
Blood Pressure		X		X	X
HbA1c		X		X	X
Waist Circumference		X		X	X
Hip Circumference^a^		X		X	X

a Hip circumference was not taken for Cohort 3 participants who had measurements taken at outside laboratory (Labcorp).

**Table 4:** Baseline characteristics of African American adult study participants in the Dietary Guidelines: 3 Diets 12-month intervention comparing a Healthy US (Nutrient Rich), Mediterranean, and Vegetarian (Plant Powered) diet.

Baseline Variables	Healthy US (Nutrient Rich)	Mediterranean	Vegetarian (Plant Powered)
n (%)	53 (32.7)	53 (32.7)	56 (34.6)
Sex			
Male	6 (35.3)	4 (23.5)	7 (41.2)
Female	47 (32.4)	49 (33.8)	49 (33.8)
Race			
AA/ African	52 (34.0)	49 (32.0)	52 (34.0)
American			
AA and other race	1 (11.1)	4 (44.4)	4 (44.4)
Education			
Some school	3 (75.0)	0 (0)	1 (25.0)
Some college	9 (25.7)	13 (37.1)	13 (37.1)
College graduate	15 (25.9)	18 (31.0)	25 (43.1)
Advanced degree	26 (40.0)	22 (33.9)	17 (26.2)
Occupation			
Employed for wages	1 (20.0)	0 (0)	4 (80.0)
No current employment	41 (34.8)	38 (32.2)	39 (33.1)
Retired	5 (25.0)	8 (40.0)	7 (35.0)
Other	6 (31.6)	7 (36.8)	6 (31.6)
Marital status			
Single	17 (27.9)	24 (39.3)	20 (32.8)
Married	21 (32.8)	19 (29.7)	24 (37.5)
Widowed	11 (40.0)	8 (29.6)	8 (29.6)
Partnered/living with someone	2 (40.0)	1 (20.0)	2 (40.0)
Divorced or separated	2 (40.0)	1 (20.0)	2 (40.0)
Means ± SD			
Mean age (y)	51.0 ± 9.3	52.1 ± 10.9	48.9 ± 10.9
BMI (kg/m^2^)	35.8 ± 5.9	37.4 ± 7.5	35.1 ± 6.5
Body weight (kg)	98.3 ± 18.9	100.6 ± 24.0	96.5 ± 21.1
HbA1c (%)	5.9 ± 1.4	6.0 ± 1.2	5.7 ± 0.4
Systolic BP (mmHg)	127.5 ± 15.1	134.6 ± 16.5	131.7 ± 17.3
Diastolic (mmHg)	82.8 ± 10.2	84.4 ± 10.8	84.9 ± 10.9
HEI score (out of 100)	57.1 ± 11.6	55.8 ± 12.1	54.8 ± 10.9
Energy intake, kcal/d	1856.5 ± 842.4	1955.8 ± 682.3	1808.0 ± 536.8
Carbohydrate intake, g/d	196.5 ± 98.7	201.7 ± 78.0	184.8 ± 63.1
Fat intake, g/d	83.5 ± 45.1	91.3 ± 34.8	84.6 ± 32.2
Protein intake, g/d	81.1 ± 29.9	82.8 ± 32.3	78.5 ± 29.1

## Data Availability

The data that has been used is confidential.

## Appendix A. Supplementary data

Supplementary data to this article can be found online at https://doi.org/10.1016/j.cct.2025.108109.
