# Supplementary material for: The Diet Guidelines: 3 Diets (DG3D) study protocol of a behavioral teaching kitchen intervention for type-2 diabetes prevention among African American adults
Source: Contemp Clin Trials. Author manuscript; Available in PMC 2026 Jun 18. (PMC13276729; doi:10.1016/j.cct.2025.108109)
Supplement: 1 [file NIHMS2184820-supplement-1.pdf]

USDA
United States Department of Agriculture

Start simple with MyPlate

Plant-Powered

MyPlate.gov

Table of Contents

Veggie Omelet in a Mug 4
Quinoa and Black Bean Salad 6
Classic Macaroni and Cheese 9
Hummus 11
Fruit and Yogurt Breakfast Shake 13
Tofu with Broccoli 15
Lentil Stew 17
Spaghetti Squash with Tomatoes, Basil, and Parmesan 19
Kale Salad with Yogurt Dressing 21
Apple Cinnamon Bars 23
Bean and Rice Burritos 25
Easiest Banana Ice Cream 27
Avocado and Corn Salsa 29
Sweet Potato Casserole 31
Apple Cranberry Salad Toss 33
Bulgur Wheat, Veggie & Chickpea Salad 35
Garden Chili 37
Simple Green Smoothie 39
Vegetable Pizza 41
Overnight Oatmeal 43
Yogurt Berry Parfait 45
Couscous with Peas and Onions 47
Rainbow Veggie Salad 49
Apple-Sage Wild Rice Stuffing 51
Minestrone Soup 53
White Bean Bruschetta 55
Banana Pudding 57
Oven-Roasted Vegetables 59
Deviled Eggs 61
Black Bean Soup 63
Scrambled Tofu 65
Oven-Baked Potato Pancakes 67
Tomato and Cucumber Salad 69
Grilled Vegetables 71
Angel Food Pastry with Fresh Berries and Whipped Cream 73
Tropical Yogurt Pops 75
Chocolate Chip Yogurt Cookies 77
Apple-Stuffed Squash 79
Grilled Fruit 81
Grilled Vegetable Packets 83
Skinny Pizza 85

Rice-Crusted Pizza 87
Curried Chickpea Salad 90
Roasted Chickpeas (Garbanzo Beans) 92
Heavenly Deviled Eggs 94
Black Bean Quesadillas 96
Broccoli Potato Soup 98
Overnight Oatmeal with Berries 100
Peppers Stuffed with Rice and Beans 102
Julia’s Sautéed & Steamed Collards 104
Bell Pepper and Apple Coleslaw 106
Slow Cooker Lentil Soup 108
Trail Mix Bars 110

Veggie Omelet in a Mug

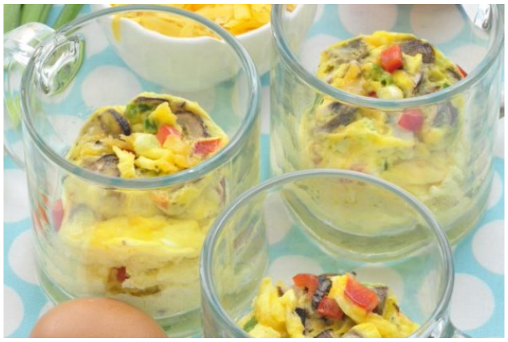

Makes: 1 Serving
Preparation Time: 10 minutes
Cook Time: 3 minutes

Making a veggie omelet has never been so easy! Combine all of your ingredients in a mug, pop it in the microwave, and breakfast is ready!

Ingredients

- 2 eggs
- 2 tablespoons fat-free (skim) milk
- 1 pinch salt
- 1 pinch black pepper
- 1/4 cup finely chopped mushrooms (or your favorite vegetables)
- 2 tablespoons low-fat cheddar cheese, shredded (or your favorite cheese)

Directions

1. Wash hands with soap and water.
2. Lightly grease the inside of a 12-ounce microwave-safe mug.
3. Use a fork to combine the eggs, milk, salt and pepper in the mug and stir well. Mix in the vegetables and cheese.
4. Microwave on HIGH for 45 seconds. Stir. Return to the microwave and cook on HIGH until the mixture has puffed and set, 60 to 90 seconds. The omelet may look wet on the top but it will dry as it cools.
5. Refrigerate leftovers within 2 hours.

Source:

Food Hero
Oregon State University Cooperative Extension Service

Nutrition Information

Serving Size: 1 recipe

Nutrients	Amount
**Total Calories**	**180**
**Total Fat**	**11 g**
Saturated Fat	4 g
Cholesterol	336 mg
**Sodium**	**397 mg**
**Carbohydrates**	**3 g**
Dietary Fiber	0 g
Total Sugars	2 g
Added Sugars included	0 g
**Protein**	**17 g**
Vitamin D	2 mcg
Calcium	216 mg
Iron	2 mg
Potassium	239 mg

Nutrients will display if the data is available

Please note: nutrient values are subject to change as data is updated

MyPlate Food Groups

Vegetables	1/4 cups
Protein Foods	2 ounces
Dairy	1/2 cups

Visit MyPlate.gov

Quinoa and Black Bean Salad

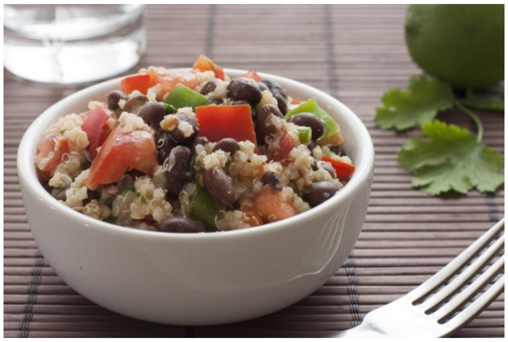

Makes: 6 servings

Quinoa (pronounced "KEEN-wah") is a whole grain with origins in South America. It is combined in this recipe with black beans, savory vegetables, and spices for a cold salad that is light and refreshing. This salad is high in protein and can be served as a main or side dish.

Ingredients

- 1/2 cup quinoa (dry)
- 1 1/2 cups water
- 1 1/2 tablespoons olive oil
- 3 teaspoons lime juice
- 1/4 teaspoon cumin
- 1/4 teaspoon coriander (ground, dried cilantro seeds)
- 2 tablespoons cilantro (chopped)
- 2 scallions (medium, minced)
- 1 can black beans, low-sodium (15.5 ounce can, rinsed and drained)
- 2 cups tomato (chopped)
- 1 red bell pepper (medium, chopped)
- 1 green bell pepper (medium, chopped)
- 2 green chiles (minced, to taste)
- black pepper (to taste)

Directions

1. Wash hands with soap and water.
2. Rinse the quinoa in cold water. Boil water in a saucepan, and then add the quinoa.
3. Return to boil, and then simmer until the water is absorbed, 10 to 15 minutes. Cool for 15 minutes.
4. While quinoa is cooking, mix olive oil, lime juice, cumin, coriander, chopped cilantro, and scallions in a small bowl, and set aside.
5. Combine chopped vegetables with the black beans in a large bowl, and set aside.
6. Once quinoa has cooled, combine all ingredients and mix well.
7. Cover and refrigerate until ready to serve.

Source:

Delicious Heart-Healthy Latino Recipes/ Platillos Latinos Sabrosos y Saludables

Nutrition Information

Serving Size: 1 cup (254g)

Nutrients	Amount
**Total Calories**	**173**
**Total Fat**	**5 g**
Saturated Fat	1 g
Cholesterol	0 mg
**Sodium**	**112 mg**
**Carbohydrates**	**27 g**
Dietary Fiber	8 g
Total Sugars	4 g
Added Sugars included	0 g
**Protein**	**7 g**
Vitamin D	0 mcg
Calcium	50 mg
Iron	3 mg
Potassium	554 mg

Nutrients will display if the data is available

Please note: nutrient values are subject to change as data is updated

MyPlate Food Groups

Vegetables	1 1/4 cups
Grains	1/2 ounces
Protein Foods	1 1/2 ounces

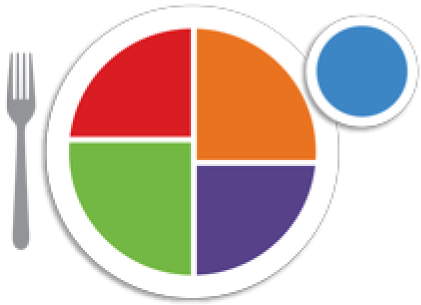

Visit MyPlate.gov

Classic Macaroni and Cheese

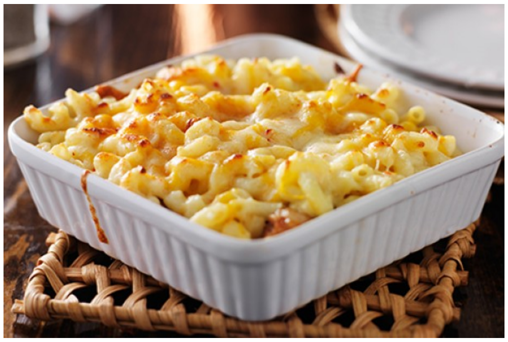

Makes: 8 servings

The ultimate comfort food! This healthier version of a family favorite uses non-fat evaporated milk and low-fat cheese to create its creamy sauce.

Ingredients

- 2 cups macaroni
- 2 sprays of cooking oil spray
- 1/2 cup onion, chopped
- 1/2 cup non-fat evaporated milk
- 1 large egg, beaten
- 1/4 teaspoon black pepper
- 1 1/4 cups finely shredded low-fat sharp cheddar cheese

Directions

1. Wash hands with soap and water.
2. Cook macaroni according to directions. (Do not add salt to the cooking water.) Drain and set aside.
3. Spray a casserole dish with nonstick cooking oil spray.
4. Preheat oven to 350 °F.
5. Lightly spray saucepan with nonstick cooking oil spray.
6. Add onions to saucepan and sauté for about 3 minutes.
7. In another bowl, combine macaroni, onions, and the remaining ingredients and mix thoroughly.
8. Transfer mixture into casserole dish.
9. Bake for 25 minutes or until bubbly. Let stand for 10 minutes before serving.

Source:

Heart Healthy Home Cooking: African American Style
US Department of Health and Human Services
National Heart, Lung, and Blood Institute

Nutrition Information

Serving Size: 1/2 cup, 1/8 of recipe

Nutrients	Amount
**Total Calories**	**170**
**Total Fat**	**3 g**
Saturated Fat	1 g
Cholesterol	28 mg
**Sodium**	**137 mg**
**Carbohydrates**	**25 g**
Dietary Fiber	1 g
Total Sugars	3 g
Added Sugars included	0 g
**Protein**	**11 g**
Vitamin D	0 mcg
Calcium	131 mg
Iron	1 mg
Potassium	121 mg

Nutrients will display if the data is available

Please note: nutrient values are subject to change as data is updated

MyPlate Food Groups

Grains 1 ounces

Dairy 1/4 cups

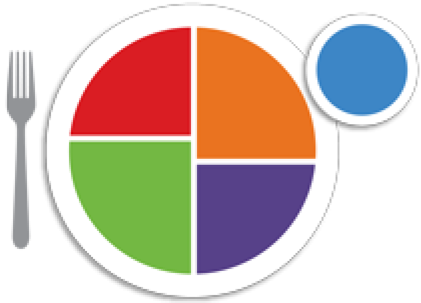

Visit MyPlate.gov

Hummus

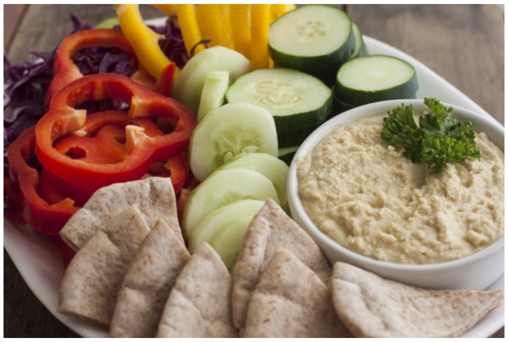

Makes: 8 Servings
Preparation Time: 20 minutes

This classic garbanzo bean dip is easy to make and a versatile dish. Serve hummus with fresh raw vegetables, on a piece of whole wheat pita bread or tortilla, on crackers, or as a sandwich filling.

Ingredients

- 2 cups garbanzo beans, cooked (also known as chickpeas)
- 2 cloves garlic (minced)
- 1/4 cup lemon juice
- 1 tablespoon sesame tahini [sesame paste] (or substitute peanut butter for a sweet taste)
- 2 tablespoons olive oil

Directions

1. Wash hands with soap and water.
2. Mash the garbanzo beans until smooth (if you have a blender, put the beans and lemon juice into it and blend).
3. Add the garlic, lemon juice, tahini and oil. Mix well.

Source:

Simple Healthy Recipes
Oklahoma Nutrition Information and Education
ONIE Project

Nutrition Information

Serving Size: 1/6 of recipe

Nutrients	Amount
**Total Calories**	**117**
**Total Fat**	**6 g**
Saturated Fat	1 g
Cholesterol	0 mg
**Sodium**	**102 mg**
**Carbohydrates**	**13 g**
Dietary Fiber	4 g
Total Sugars	2 g
Added Sugars included	0 g
**Protein**	**4 g**
Vitamin D	0 mcg
Calcium	29 mg
Iron	1 mg
Potassium	141 mg

Nutrients will display if the data is available

Please note: nutrient values are subject to change as data is updated

MyPlate Food Groups

Vegetables	1/4 cups
Protein Foods	1 ounces

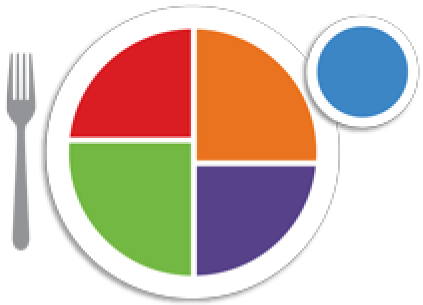

Visit MyPlate.gov

Fruit and Yogurt Breakfast Shake

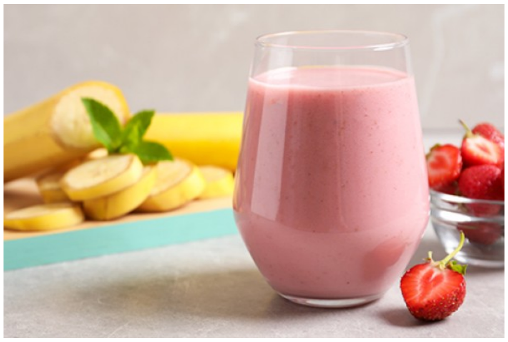

Makes: 2 servings

Shake up your day with this quick and easy breakfast. Make this recipe with any of your favorite fruits, especially when they are in season!

Ingredients

- 1 banana, medium, very ripe, (peeled)
- 3/4 cup pineapple juice
- 1/2 cup yogurt, low-fat vanilla
- 1/2 cup strawberries (remove stems and rinse)

Directions

1. Wash hands with soap and water.
2. Blend banana with pineapple juice, yogurt, and strawberries in a blender.
3. Blend until smooth.
4. Divide shake between 2 glasses and serve immediately.

Source:

Recipes to Grow On
University of Illinois Extension Service

Nutrition Information

Serving Size: 1/2 of recipe

Nutrients	Amount
**Total Calories**	**168**
**Total Fat**	**1 g**
Saturated Fat	1 g
Cholesterol	3 mg
**Sodium**	**43 mg**
**Carbohydrates**	**37 g**
Dietary Fiber	3 g
Total Sugars	27 g
Added Sugars included	4 g
**Protein**	**4 g**
Vitamin D	0 mcg
Calcium	127 mg
Iron	1 mg
Potassium	531 mg

Nutrients will display if the data is available

Please note: nutrient values are subject to change as data is updated

MyPlate Food Groups

Fruits 1 cups

Dairy 1/4 cups

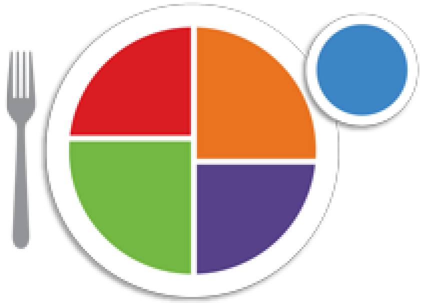

Visit MyPlate.gov

Tofu with Broccoli

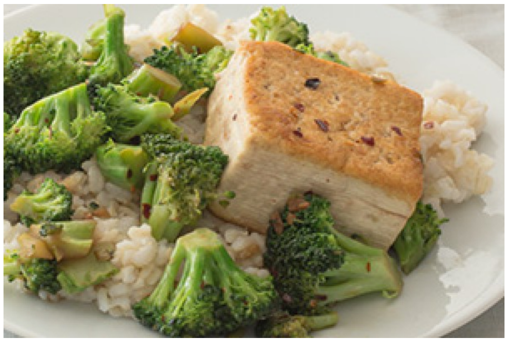

Makes: 4 Servings
Preparation Time: 20 minutes
Cook Time: 35 minutes

Tofu cooked until golden brown, and broccoli sautéed in a soy and scallion sauce make up this one-skillet meal. Serve with a side of brown rice for a quick dish.

Ingredients

- 1 pound firm tofu
- 4 teaspoons vegetable oil
- 2 cloves garlic (peeled and minced)
- 2 scallions (green and whites chopped)
- 1 head broccoli (broken into florets, stems chopped)
- 1/4 cup water
- 1 tablespoon low-sodium soy sauce
- 1/2 teaspoon crushed red pepper flakes
- 1 tablespoon chopped fresh cilantro (or basil leaves)

Directions

1. Put the tofu on a cutting board and cut it in half and then cut each half into 4 equal pieces.
2. Put 2 layers of paper towel on a cutting board and put the tofu on top. Let drain at least 20 minutes (and up to 2 hours).
3. Place a skillet over medium heat and when hot, add 2 teaspoons oil. Add the tofu, one piece at a time, and cook until golden, about 3 minutes per side. Remove the tofu to a plate and set aside.
4. Reheat the skillet and when it is hot, add remaining 2 teaspoons oil. Add garlic and scallions and cook until just golden, about 2 minutes. Add the broccoli and water and raise the heat to high. Cook until the broccoli is tender, about 4 minutes.
5. Return the tofu to the pan and add the remaining ingredients. Stir well.
6. Serve with a side of brown rice.

Source:

USDA Center for Nutrition Policy and Promotion

Nutrition Information

Serving Size: 4 ounce

Nutrients	Amount
**Total Calories**	**151**
**Total Fat**	**10 g**
Saturated Fat	1 g
Cholesterol	0 mg
**Sodium**	**173 mg**
**Carbohydrates**	**8 g**
Dietary Fiber	3 g
Total Sugars	2 g
Added Sugars included	0 g
**Protein**	**12 g**
Vitamin D	0 mcg
Calcium	269 mg
Iron	3 mg
Potassium	437 mg

Nutrients will display if the data is available

Please note: nutrient values are subject to change as data is updated

MyPlate Food Groups

Vegetables	1 cup
Protein Foods	2 ounces

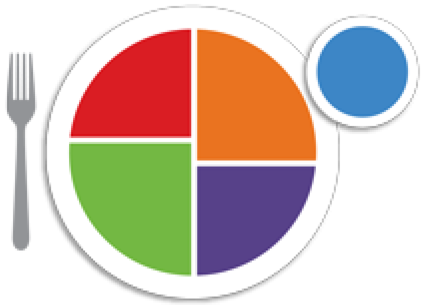

Visit MyPlate.gov

Lentil Stew

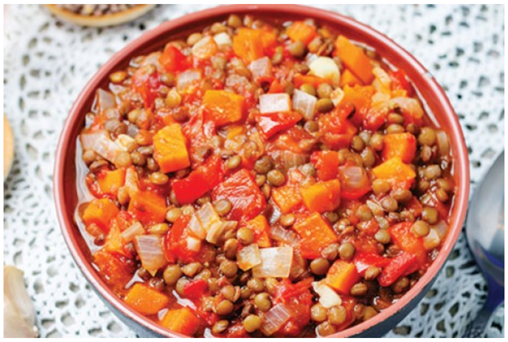

Makes: 10 servings

Lentils, carrots, and tomatoes are a savory and satisfying combination in this delicious and easy to prepare stew. Enjoy with a side of your favorite citrus fruit.

Ingredients

- 2 teaspoons olive oil (or canola oil)
- 1 onion (large, chopped)
- 1 teaspoon garlic powder
- 1 1/2 10-ounce packages of frozen sliced carrots
- 1 cup dry lentils (rinsed and drained)
- 3 cans diced tomatoes, low-sodium (14.5 ounces each)
- 3 cups water
- 1 teaspoon chili powder

Directions

1. Wash hands with soap and water.
2. Heat the oil in a large pot over medium heat.
3. Add chopped onion.
4. Cook for 3 minutes, or until tender.
5. Stir in garlic powder, carrots, lentils, tomatoes, water, and chili powder.
6. Simmer, uncovered, for about 20 minutes or until lentils are tender.

Source:

2009 Recipe Calendar
University of Maryland Extension
Food Supplement Nutrition Education Program

Nutrition Information

Serving Size: 1 cup

Nutrients	Amount
**Total Calories**	**113**
**Total Fat**	**2 g**
Saturated Fat	0 g
Cholesterol	0 mg
**Sodium**	**52 mg**
**Carbohydrates**	**20 g**
Dietary Fiber	7 g
Total Sugars	6 g
Added Sugars included	0 g
**Protein**	**6 g**
Vitamin D	0 mcg
Calcium	69 mg
Iron	3 mg
Potassium	536 mg

Nutrients will display if the data is available

Please note: nutrient values are subject to change as data is updated

MyPlate Food Groups

Vegetables	1 1/4 cups
Protein Foods	1 ounces

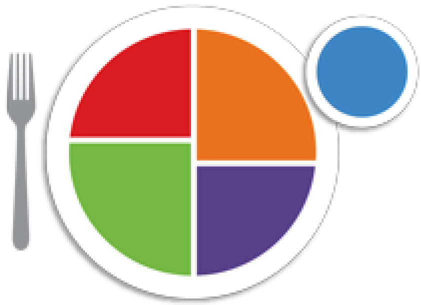

Visit MyPlate.gov

Spaghetti Squash with Tomatoes, Basil, and Parmesan

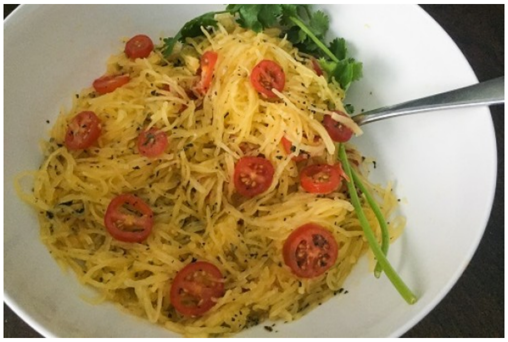

Makes: 4 Servings

Here is a delicious late summer side dish bursting with fresh flavors. This dish would go well with a grilled entrée.

Ingredients

- 1 1/2 pounds spaghetti squash (about 1 squash)
- 1 tablespoon olive oil
- 3 tablespoons Parmesan cheese
- 1/4 teaspoon dried oregano
- 2 teaspoons dried basil (or 1/2 cup fresh basil, chopped)
- 1 cup cherry tomatoes (thinly sliced)
- salt and pepper (to taste, optional)

Directions

1. Wash hands with soap and water.
2. Place the 2 squash halves, cut side down, in glass baking dish. Add about 1/4 cup water and cover with plastic wrap. Microwave on high 12 minutes or until soft when pressed. Let stand covered for 3 minutes.
3. In a large bowl, whisk oil, basil, oregano and 2 tablespoons Parmesan cheese. Stir in tomatoes and season lightly with salt and pepper to taste.
4. Scrape squash out with a fork, add strands to tomato mixture, and toss until combined. Sprinkle with remaining 1 tablespoon Parmesan cheese.

Source:

Eat Smart. Be Fit.
University of Maryland Extension

Nutrition Information

Serving Size: 1/4 of recipe

Nutrients	Amount
**Total Calories**	**77**
**Total Fat**	**5 g**
Saturated Fat	1 g
Cholesterol	3 mg
**Sodium**	**67 mg**
**Carbohydrates**	**7 g**
Dietary Fiber	2 g
Total Sugars	3 g
Added Sugars included	0 g
**Protein**	**2 g**
Vitamin D	0 mcg
Calcium	83 mg
Iron	1 mg
Potassium	195 mg

Nutrients will display if the data is available

Please note: nutrient values are subject to change as data is updated

MyPlate Food Groups

Vegetables	1 cups
Dairy	1/4 cups

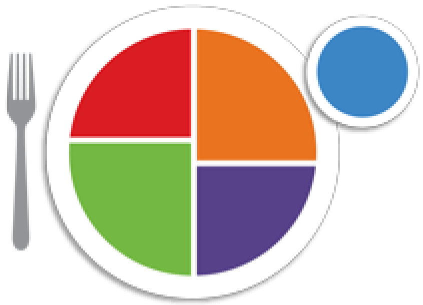

[Visit MyPlate.gov](https://www.myplate.gov)

Kale Salad with Yogurt Dressing

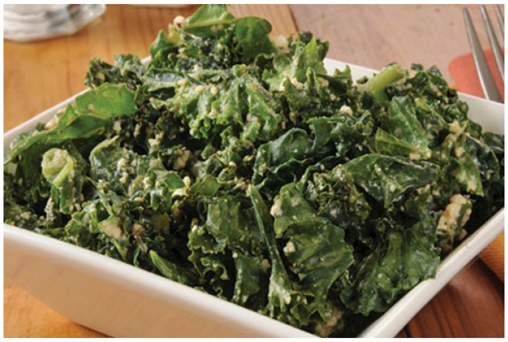

Makes: 4 Servings
Preparation Time: 15 minutes

This recipe redefines what a kale salad can be. By massaging the kale first, you break down the fibers leaving the greens tender and delicious!

Ingredients

- 4 cups kale (1 bunch)
- 2 tablespoons olive oil (or oil of your choice, divided)
- 1 lemon (zested and juiced - save zest for dressing)
- 1 lime (zested and juiced - save zest for dressing)

Dressing

- 1 cup Greek yogurt (plain, fat-free)
- salt and pepper (optional, to taste)
- 1/2 cup pumpkin seeds, toasted (pepitas)
- 1/2 cup cilantro (chopped or torn, stems included)
- 1 jalapeño chile pepper (seeds removed and diced, optional)

Directions

1. Wash hands with soap and water.
2. Wash and dry kale. Remove stems and tear into bite-sized pieces.
3. Season kale with salt, pepper, 1 tablespoon of olive oil, lemon juice, and lime juice.
4. With your hands, massage kale until the leaves just start to wilt.
5. Make the dressing - in a small bowl, add the salt and pepper to the yogurt.
6. Add the lemon and lime zest and remaining 1 tablespoon of oil.
7. Mix in cilantro and jalapeno pepper, if desired.
8. With a spoon, mix massaged kale with yogurt dressing.
9. Toss pumpkin seeds over salad.

Source:

USDA Supplemental Nutrition Assistance Program (SNAP)

Nutrition Information

Serving Size: 1/4 of recipe

Nutrients	Amount
**Total Calories**	**304**
**Total Fat**	**22 g**
Saturated Fat	3 g
Cholesterol	1 mg
**Sodium**	**129 mg**
**Carbohydrates**	**17 g**
Dietary Fiber	4 g
Total Sugars	4 g
Added Sugars included	0 g
**Protein**	**18 g**
Vitamin D	0 mcg
Calcium	200 mg
Iron	4 mg
Potassium	714 mg

Nutrients will display if the data is available

Please note: nutrient values are subject to change as data is updated

MyPlate Food Groups

Fruits	1/4 cups
Vegetables	1/2 cups
Protein Foods	2 ounces
Dairy	1/4 cups

Visit MyPlate.gov

Apple Cinnamon Bars

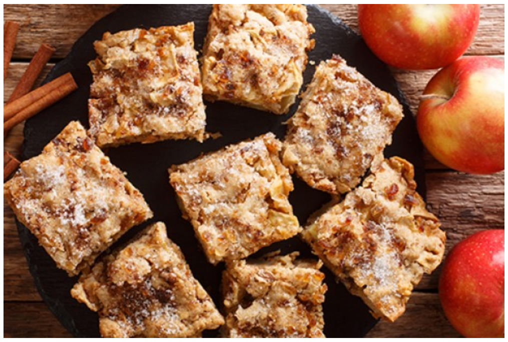

Makes: 24 servings

Apples are sandwiched between two cinnamon crumb layers for a delicious treat.

Ingredients

- 4 medium apples
- 1 cup flour
- 1/4 teaspoon salt
- 1/2 teaspoon baking soda
- 1/2 teaspoon cinnamon
- 1/2 cup brown sugar, packed
- 1 cup oats, uncooked
- 1/2 cup margarine, 1 stick (or butter)
- 4 sprays of cooking spray

Directions

1. Wash hands with soap and water.
2. Preheat the oven to 350 degrees F.
3. Put the flour, salt, baking soda, cinnamon, brown sugar, and oats in the mixing bowl. Stir together.
4. Add the margarine to the bowl. Use the 2 table knives to mix the ingredients and cut them into crumbs.
5. Lightly grease the bottom and sides of the baking dish with cooking spray.
6. Spread half of the crumb mixture in the greased baking dish.
7. Remove the core from the apples and slice them. Put the apple slices into the baking dish.
8. Top the apples with the rest of the crumb mixture.
9. Bake in the oven for 40-45 minutes.
10. Cut into squares. It will fall apart easily.

Source:

Pennsylvania Nutrition Education Network (original)
Recipe modification adapted from SNAP-Ed New York, October 2023

Nutrition Information

Serving Size: 1 bar, 1/24 of recipe

Nutrients	Amount
**Total Calories**	**82**
**Total Fat**	**4 g**
Saturated Fat	1 g
Cholesterol	0 mg
**Sodium**	**53 mg**
**Carbohydrates**	**12 g**
Dietary Fiber	1 g
Total Sugars	7 g
Added Sugars included	4 g
**Protein**	**1 g**
Vitamin D	0 mcg
Calcium	7 mg
Iron	0 mg
Potassium	37 mg

Nutrients will display if the data is available

Please note: nutrient values are subject to change as data is updated

MyPlate Food Groups

Fruits 1/4 cups

Grains 1/2 ounces

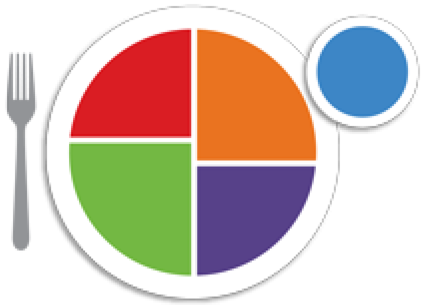

Visit MyPlate.gov

Bean and Rice Burritos

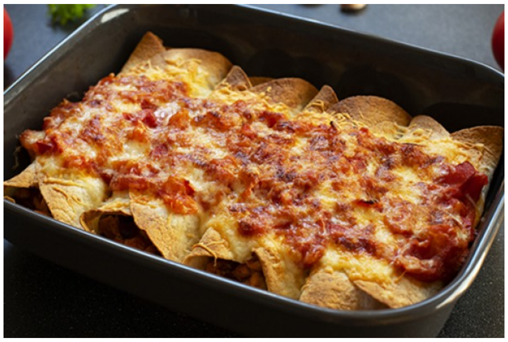

Makes: 8 servings

These baked burritos are a great way to use leftover cooked rice. Try them with brown rice for a whole grain boost.

Ingredients

- 2 cups cooked rice
- 1 small onion, chopped
- 2 cups cooked kidney beans (1 (15 ounce) can, low-sodium, drained)
- 8 (10 inch) flour tortillas
- 1/2 cup salsa
- 1/2 cup cheese, shredded

Directions

1. Wash hands with soap and water.
2. Preheat the oven to 300 °F.
3. Peel the onion, and chop it into small pieces.
4. Drain the liquid from the cooked (or canned) kidney beans.
5. Mix the rice, chopped onion, and beans in a bowl.
6. Put each tortilla on a flat surface.
7. Put 1/2 cup of the rice and bean mix in the middle of each tortilla.
8. Fold the sides of the tortilla to hold the rice and beans.
9. Put each filled tortilla (burrito) in the baking pan.
10. Bake for 15 minutes.
11. While the burritos are baking, grate 1/2 cup cheese.
12. Pour the salsa over the baked burritos. Add cheese.
13. Serve the burritos warm.

Source:

Pennsylvania Nutrition Education Network

Nutrition Information

Serving Size: 1 burrito, 1/8 of recipe

Nutrients	Amount
**Total Calories**	**341**
**Total Fat**	**8 g**
Saturated Fat	3 g
Cholesterol	7 mg
**Sodium**	**607 mg**
**Carbohydrates**	**55 g**
Dietary Fiber	5 g
Total Sugars	3 g
Added Sugars included	0 g
**Protein**	**11 g**
Vitamin D	0 mcg
Calcium	162 mg
Iron	3 mg
Potassium	285 mg

Nutrients will display if the data is available

Please note: nutrient values are subject to change as data is updated

MyPlate Food Groups

Vegetables	1/2 cups
Grains	3 ounces
Protein Foods	1 ounces
Dairy	1/4 cups

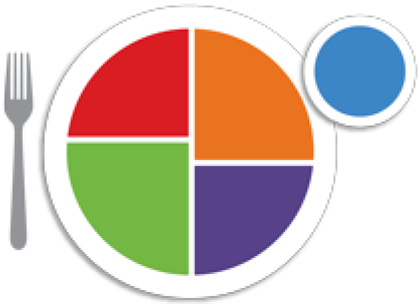

Visit MyPlate.gov

Easiest Banana Ice Cream

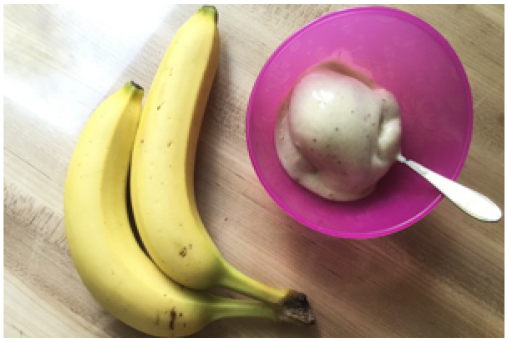

Makes: 3 Servings

What should you do with over-ripe bananas? Don't toss them! Freeze them and then throw them in the blender to make this delicious and creamy frozen dessert.

Ingredients

- 2 1/2 bananas (2-3 bananas)
- 3 tablespoons 1% milk
- chocolate syrup (optional)

Directions

1. Wash hands with soap and water.
2. When bananas have become very ripe, peel and slice them into medium size round pieces. Place on a plastic wrap covered baking sheet and put in freezer overnight.
3. Place frozen bananas in a food processor or blender with a small splash of milk (not over 1/4 cup total).
4. Pulse food processor or blender until bananas begin to break up. They will be tiny balls of bananas at this point.
5. Using a spoon or rubber spatula, scrape down the banana mixture. Continue running the food processor until the mixture is smooth and creamy. This may take a few minutes. The ice cream will look and tasted like soft serve ice cream when finished.
6. Serve in an ice cream cone or in a small bowl with a small ribbon of chocolate syrup on top (optional).

Source:

Alabama Cooperative Extension System

Nutrition Information

Serving Size: 1/3 of recipe

Nutrients	Amount
**Total Calories**	**94**
**Total Fat**	**0 g**
Saturated Fat	0 g
Cholesterol	1 mg
**Sodium**	**8 mg**
**Carbohydrates**	**23 g**
Dietary Fiber	3 g
Total Sugars	13 g
Added Sugars included	0 g
**Protein**	**2 g**
Vitamin D	0 mcg
Calcium	24 mg
Iron	0 mg
Potassium	375 mg

Nutrients will display if the data is available

Please note: nutrient values are subject to change as data is updated

MyPlate Food Groups

Fruits 3/4 cups

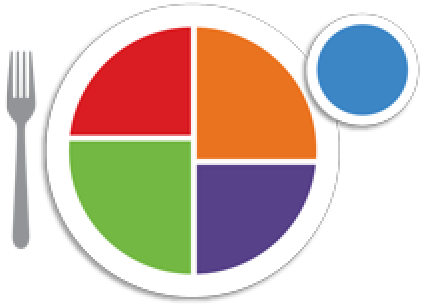

Visit MyPlate.gov

Avocado and Corn Salsa

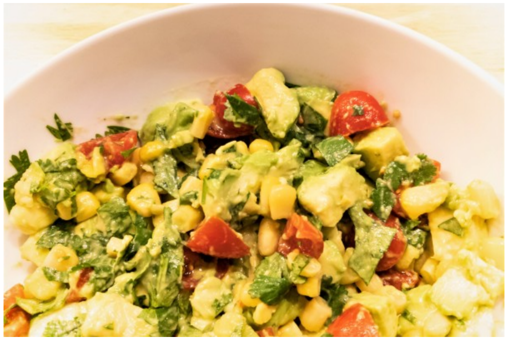

Makes: 5 Servings

Avocado and corn are given a flavor boost by fresh cilantro and lime. Serve this salsa with our Baked Chicken for an easy weekday meal.

Ingredients

- 1 avocado, diced
- 3/4 cup frozen corn kernels, thawed
- 1/2 cup grape tomatoes, quartered
- 1 tablespoon fresh cilantro, chopped
- 2 teaspoons lime juice
- 1/4 teaspoon salt

Directions

1. Wash hands with soap and water.
2. Toss avocado, corn, tomatoes, cilantro, lime juice, and salt in a medium bowl.
3. Chill one hour and then serve.

Source:

Bronson Wellness Center
Bronson Healthcare - Michigan

Nutrition Information

Serving Size: 1/2 cup

Nutrients	Amount
**Total Calories**	**67**
**Total Fat**	**4 g**
Saturated Fat	1 g
Cholesterol	0 mg
**Sodium**	**119 mg**
**Carbohydrates**	**8 g**
Dietary Fiber	3 g
Total Sugars	1 g
Added Sugars included	0 g
**Protein**	**1 g**
Vitamin D	0 mcg
Calcium	6 mg
Iron	0 mg
Potassium	235 mg

Nutrients will display if the data is available

Please note: nutrient values are subject to change as data is updated

MyPlate Food Groups

Vegetables 1/2 cups

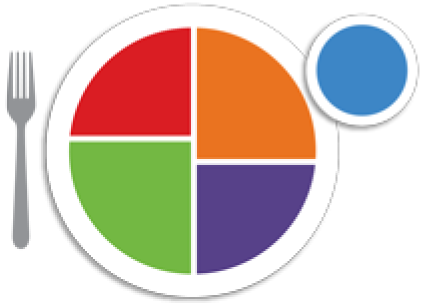

Visit MyPlate.gov

Sweet Potato Casserole

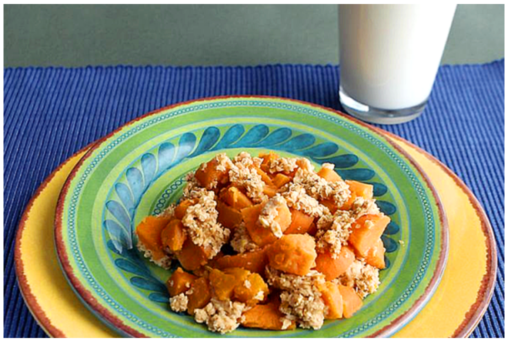

Makes: 6 Servings

Sweet potato casserole is rich is flavor and low in fat.

Ingredients

- 2 tablespoons 1% low fat milk
- 1 1/2 teaspoons brown sugar
- 1 teaspoon ground cinnamon
- 1/4 cup quick cooking oats, dry
- 2 3/4 cups low-sodium sweet potatoes, drained and chopped (about 15 ounces)

Directions

1. Preheat oven to 350 °F.
2. In a small bowl, combine milk, brown sugar, cinnamon, and oatmeal. Mix well and set aside.
3. In a medium-sized baking pan, add the sweet potatoes so that they cover the bottom of the pan.
4. Add the oatmeal mixture on top of the sweet potatoes.
5. Bake for 20 minutes. Serve hot or refrigerate and serve cold.

Source:

A Harvest of Recipes with USDA Foods
USDA Food Distribution Program on Indian Reservations

Nutrition Information

Serving Size: 1/4 cup

Nutrients	Amount
**Total Calories**	**94**
**Total Fat**	**1 g**
Saturated Fat	0 g
Cholesterol	0 mg
**Sodium**	**30 mg**
**Carbohydrates**	**21 g**
Dietary Fiber	3 g
Total Sugars	5 g
Added Sugars included	1 g
**Protein**	**2 g**
Vitamin D	0 mcg
Calcium	33 mg
Iron	1 mg
Potassium	309 mg

Nutrients will display if the data is available

Please note: nutrient values are subject to change as data is updated

MyPlate Food Groups

Vegetables 1/2 cups

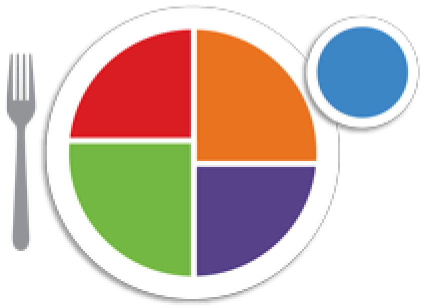

[Visit MyPlate.gov](https://www.myplate.gov)

Apple Cranberry Salad Toss

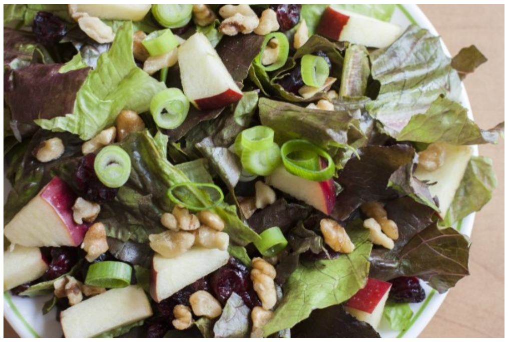

Makes: 8 servings

Enjoy the best of fall flavors with this sweet and tart green salad. Light yet crisp, it’s a perfect dish for the autumnal change of weather.

Ingredients

- 1 medium head of lettuce (about 10 cups)
- 2 medium apples, sliced
- 1/2 cup chopped walnuts
- 1 cup dried cranberries
- 1/2 cup green onions, sliced
- 3/4 cup vinaigrette dressing

Directions

1. Wash hands with soap and water.
2. Toss lettuce, apples, walnuts, cranberries, and onions in a large bowl.
3. Add dressing; toss to coat. Serve immediately.

Source:

Creative Recipes for Less Familiar USDA Commodities Used by Household Programs
USDA, Food and Nutrition Service, Food Distribution Service

Nutrition Information

Serving Size: 1/8 of recipe

**Nutrients**	**Amount**
**Total Calories**	**174**
**Total Fat**	**10 g**
Saturated Fat	1 g
Cholesterol	0 mg
**Sodium**	**227 mg**
**Carbohydrates**	**22 g**
Dietary Fiber	3 g
Total Sugars	17 g
Added Sugars included	9 g
**Protein**	**2 g**
Vitamin D	0 mcg
Calcium	30 mg
Iron	1 mg
Potassium	206 mg

Nutrients will display if the data is available

Please note: nutrient values are subject to change as data is updated

MyPlate Food Groups

Fruits	1/2 cups
Vegetables	3/4 cups
Protein Foods	1/2 ounces

Visit MyPlate.gov

Bulgur Wheat, Veggie & Chickpea Salad

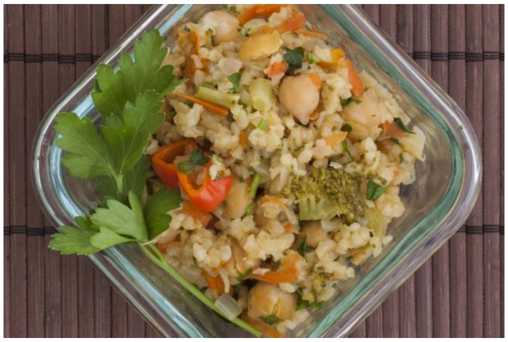

Makes: 8 servings

Bulgur is the star whole grain of this simple and colorful salad.

Ingredients

- 1 medium onion, chopped
- 1 cup broccoli, chopped
- 1 cup carrots, shredded
- 1 small green pepper, chopped (or red or yellow pepper)
- 1/3 cup fresh parsley, chopped (or 2 tablespoons dried)
- 1 teaspoon canola oil (or cooking oil of choice)
- 1 1/2 cups dry bulgur
- 2 cups low-sodium chicken broth
- 1 cup low-sodium canned chickpeas, drained (garbanzo beans)

Directions

1. Wash hands with soap and water.
2. Wash and chop fresh onion, broccoli, carrots, pepper and parsley (if using fresh parsley).
3. Heat canola oil in a large skillet over medium-high heat. Add onions and cook until soft.
4. Add bulgur and stir to coat. Add 2 cups chicken broth to the skillet, bring to a boil.
5. Lower the heat, add remaining vegetables and chickpeas. Cook for 10 minutes or until the liquid is absorbed.
6. Add parsley and stir. Serve warm or cold.

Source:

Recipe Adapted from: 5 A Day Bulger Wheat
Choices: Steps Toward Health
University of Massachusetts Extension Nutrition Education Program

Nutrition Information

Serving Size: 1/8 of recipe

Nutrients	Amount
**Total Calories**	**140**
**Total Fat**	**2 g**
Saturated Fat	0 g
Cholesterol	0 mg
**Sodium**	**66 mg**
**Carbohydrates**	**27 g**
Dietary Fiber	7 g
Total Sugars	2 g
Added Sugars included	0 g
**Protein**	**6 g**
Vitamin D	0 mcg
Calcium	38 mg
Iron	2 mg
Potassium	283 mg

Nutrients will display if the data is available

Please note: nutrient values are subject to change as data is updated

MyPlate Food Groups

Vegetables	1/2 cups
Grains	1 1/2 ounces
Protein Foods	1/2 ounces

Visit MyPlate.gov

Garden Chili

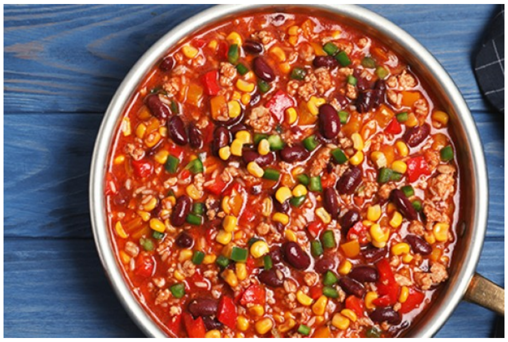

Makes: 4 servings

This quick and easy one pan dish is full of veggies, spices, and flavor. Get your kids involved by letting them wash the vegetables.

Ingredients

- 3/4 pound ground beef (lean, 10% fat)
- 1/2 cup green pepper (chopped)
- 1/2 large onion (chopped)
- 1/2 cup celery (chopped)
- 2/3 cup kidney beans, canned, low-sodium (drained and rinsed)
- 1/2 cup corn (sweet)
- 1 can tomato sauce, low-sodium (8 oz)
- 1 cup fresh tomatoes (chopped)
- 1 dash black pepper
- 1/2 teaspoon garlic powder
- 2 teaspoons chili powder

Directions

1. Wash your hands with soap and water.
2. Place ground beef in a skillet sprayed with non-stick cooking spray. Brown meat over medium-high heat, stirring occasionally to break it into pieces. Drain fat and blot meat with paper towels. Transfer beef into a colander and rinse with very hot water to further remove fat.
3. Add green pepper, onion, and celery. Cook until softened.
4. Add beans, corn, tomato sauce, chopped tomatoes, pepper, garlic powder and chili powder.
5. Cook mixture over low heat for 20 minutes.
6. Serve hot in bowls. Or serve as a dip with baked tortilla chips or on a bun.
7. Cover and refrigerate leftovers within 2 hours.

Source:

Fix it Fresh! Recipe Series
Kansas State University Research and Extension

Nutrition Information

Serving Size: 1/4 of recipe

Nutrients	Amount
**Total Calories**	**224**
**Total Fat**	**8 g**
Saturated Fat	3 g
Cholesterol	54 mg
**Sodium**	**169 mg**
**Carbohydrates**	**17 g**
Dietary Fiber	5 g
Total Sugars	7 g
Added Sugars included	3 g
**Protein**	**20 g**
Vitamin D	0 mcg
Calcium	53 mg
Iron	3 mg
Potassium	758 mg

Nutrients will display if the data is available

Please note: nutrient values are subject to change as data is updated

MyPlate Food Groups

Vegetables	1 1/4 cups
Protein Foods	3 ounces

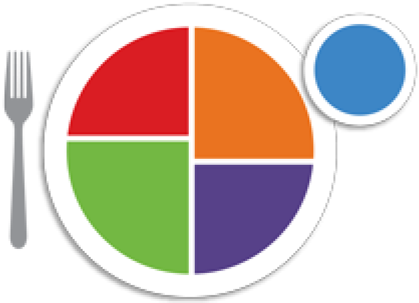

Visit MyPlate.gov

Simple Green Smoothie

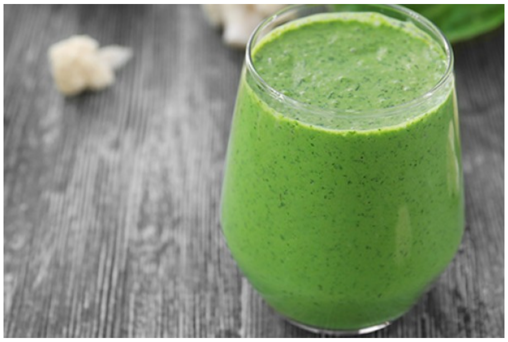

Makes: 2 Servings

Add kale or spinach to this fruit smoothie for a quick and delicious breakfast or snack.

Ingredients

- 1 cup kale or spinach
- 1 medium banana
- 1 cup low-fat milk (or fortified soy, coconut, or almond milk)
- 1 cup plain low-fat yogurt
- 1 medium apple (cored and sliced)
- 1 cup frozen fruit (all one fruit or a combination of mixed frozen fruit)
- flax seeds, 1 tablespoon (optional)
- chia seeds, 1 tablespoon (optional)

Directions

1. Wash hands with soap and water.
2. Add the kale or spinach and the liquid of your choice to a blender. Blend.
3. Add in the rest of the ingredients, blending after each item.
4. Serve and enjoy, cold.
5. Reserve the leftover smoothie in the refrigerator for later in the day or the next day.

Source:

Northern Valley Catholic Social Service

Nutrition Information

Nutrients	Amount
**Total Calories**	**299**
**Total Fat**	**4 g**
Saturated Fat	2 g
Cholesterol	N/A
**Sodium**	**156 mg**
**Carbohydrates**	**56 g**
Dietary Fiber	7 g
Total Sugars	38 g
Added Sugars included	0 g
**Protein**	**13 g**
Vitamin D	1 mcg
Calcium	454 mg
Iron	2 mg
Potassium	1119 mg

Nutrients will display if the data is available

Please note: nutrient values are subject to change as data is updated

MyPlate Food Groups

Fruits	2 cups
Vegetables	1/4 cups
Dairy	1 cup

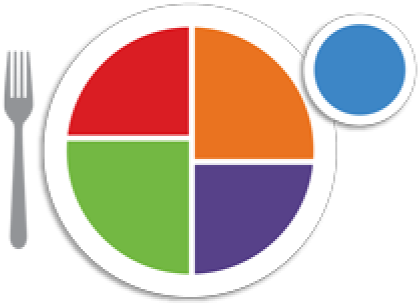

Visit MyPlate.gov

Vegetable Pizza

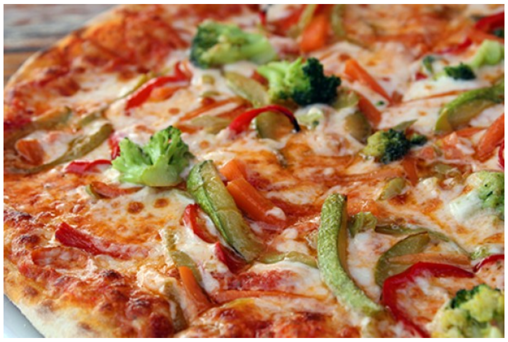

Makes: 8 servings

Enjoy fresh, delicious pizza made at home. You can spice this up with red pepper flakes or garlic powder. Try with whatever vegetables you have on hand.

Ingredients

- 3/4 cup low-sodium pizza sauce
- 1 large (12-inch) Italian pizza shell
- 1 cup broccoli, chopped
- 1 cup carrots, shredded
- 1/2 cup green bell pepper, sliced (or red bell pepper)
- 5 ounces part-skim mozzarella cheese, shredded

Directions

1. Wash hands with soap and water.
2. Preheat the oven to 450 degrees F.
3. Spoon pizza sauce on pizza shell.
4. Put pizza shell on a cookie sheet. Arrange vegetables over sauce. Sprinkle on the cheese.
5. Bake for 10 minutes.
6. When baked, cool pizza for 3 minutes before slicing. Cut into 8 wedges.

Source:

Adapted from Spunky Vegetable Pizza, California 5 A Day, It's So Easy
Contra Costa Health Services

Nutrition Information

Serving Size: 1 slice, 1/8 of recipe

Nutrients	Amount
**Total Calories**	**213**
**Total Fat**	**6 g**
Saturated Fat	3 g
Cholesterol	10 mg
**Sodium**	**285 mg**
**Carbohydrates**	**29 g**
Dietary Fiber	2 g
Total Sugars	3 g
Added Sugars included	0 g
**Protein**	**10 g**
Vitamin D	N/A
Calcium	198 mg
Iron	2 mg
Potassium	61 mg

Nutrients will display if the data is available

Please note: nutrient values are subject to change as data is updated

MyPlate Food Groups

Vegetables	1/2 cups
Grains	2 ounces
Dairy	1/2 cups

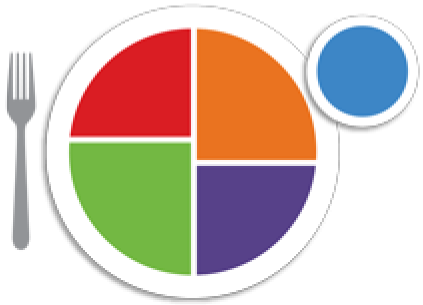

Visit MyPlate.gov

Overnight Oatmeal

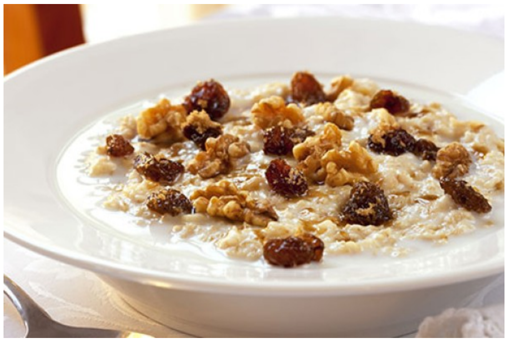

Makes: 2 Servings

Overnight soaking cuts down on prep time in the morning, so you can enjoy a healthy and hearty, cooked breakfast, even after hitting the snooze button.

Ingredients

- 1 cup dry oats
- 2 1/2 cups water
- 1/2 cup dried fruit (raisins, cranberries, or apricots)
- 1/4 cup walnuts, lightly chopped (or pecans)

Directions

1. Wash hands with soap and water.
2. Put all ingredients in a cooking pot and cover. Let sit overnight in refrigerator.
3. In the morning, put on a stove burner and cook on medium heat until simmering, then turn on low and heat for 10 to 15 minutes more or less depending on type of oats -- quick (5 minutes), regular, or steel cut.
4. Serve warm with a little milk (or milk alternative), a drizzle of maple syrup, and a side of fresh fruit, if desired.

Source:

Lake Family Resource Center
Be-Fresh Program

Nutrition Information

Serving Size: 1/2 of recipe

Nutrients	Amount
**Total Calories**	**333**
**Total Fat**	**13 g**
Saturated Fat	2 g
Cholesterol	0 mg
**Sodium**	**27 mg**
**Carbohydrates**	**50 g**
Dietary Fiber	7 g
Total Sugars	17 g
Added Sugars included	0 g
**Protein**	**8 g**
Vitamin D	0 mcg
Calcium	57 mg
Iron	3 mg
Potassium	470 mg

Nutrients will display if the data is available

Please note: nutrient values are subject to change as data is updated

MyPlate Food Groups

Fruits	1/2 cups
Grains	2 ounces
Protein Foods	1 ounces

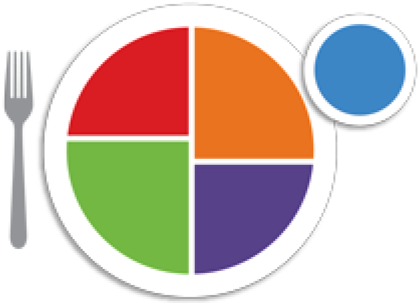

Visit MyPlate.gov

Yogurt Berry Parfait

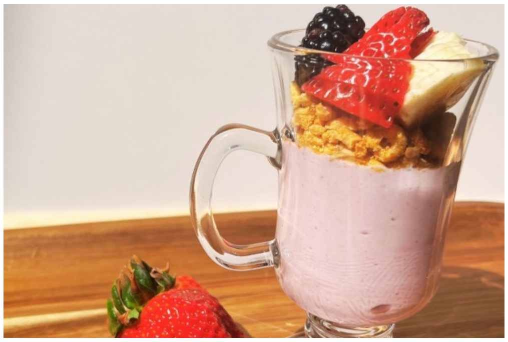

Makes: 4 servings
Preparation Time: 5 minutes

Enjoy this hearty snack or dessert, abounding with fresh fruit, granola, and any flavor of low-fat yogurt.

Ingredients

- 2 cups low fat yogurt (or fat-free, any flavor*)
- 1 cup banana (sliced)
- 1/2 cup blueberries (fresh)
- 1/2 cup strawberries (fresh, sliced)
- other optional fruit (raspberries, peaches, pineapple and/or mangos)
- 1 cup granola

Directions

1. Wash hands with soap and water.
2. Line up 4 parfait or other tall glasses.
3. Spoon 1/2 cup of yogurt into each glass.
4. Sprinkle with granola.
5. Top with about 1/4 cup of fruit.

Source:

USDA Food and Nutrition Information Center

Nutrition Information

Serving Size: 1 parfait, 1/4 of recipe (214g)

Nutrients	Amount
**Total Calories**	**304**
**Total Fat**	**9 g**
Saturated Fat	2 g
Cholesterol	6 mg
**Sodium**	**89 mg**
**Carbohydrates**	**46 g**
Dietary Fiber	5 g
Total Sugars	30 g
Added Sugars included	11 g
**Protein**	**11 g**
Vitamin D	1 mcg
Calcium	240 mg
Iron	2 mg
Potassium	613 mg

Nutrients will display if the data is available

Please note: nutrient values are subject to change as data is updated

MyPlate Food Groups

Fruits	1/2 cups
Grains	1/2 ounces
Protein Foods	1/2 ounces
Dairy	1/2 cups

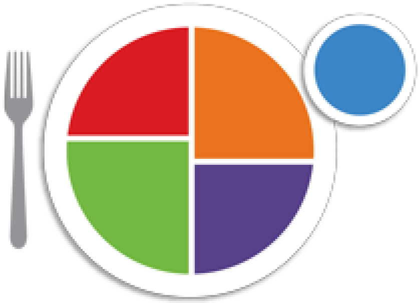

Visit MyPlate.gov

Couscous with Peas and Onions

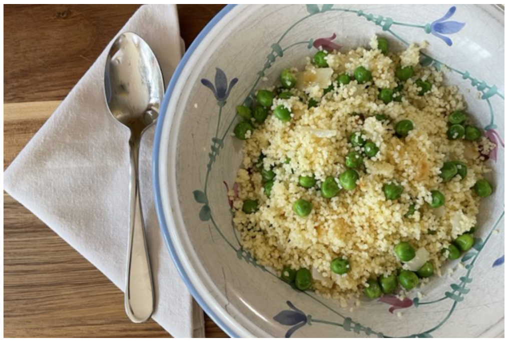

Makes: 4 servings

Couscous is cooked with sage, peas, and onions for a savory side dish. Look for couscous or whole grain couscous in the aisle near the rice.

Ingredients

- 1 teaspoon olive oil
- 1 cup onion, finely chopped
- 1 cup frozen green peas
- 1/2 teaspoon ground sage
- 1 1/3 cups water
- 1 cup couscous
- salt (1/2 teaspoon, optional)

Directions

1. Wash hands with soap and water.
2. Combine oil and onions in heavy skillet.
3. Sauté for 5 to 10 minutes until lightly browned.
4. Add the peas, sage, water, couscous, and salt if desired.
5. Cover and cook on medium heat for about 5 minutes or until peas are tender but still bright green and all of the water is absorbed.
6. Fluff with fork.

Source:

CHOICES: Steps Toward Health
UMass Extension Nutrition Education Program

Nutrition Information

Serving Size: 1 cup

Nutrients	Amount
**Total Calories**	**205**
**Total Fat**	**1 g**
Saturated Fat	0 g
Cholesterol	0 mg
**Sodium**	**40 mg**
**Carbohydrates**	**40 g**
Dietary Fiber	5 g
Total Sugars	4 g
Added Sugars included	0 g
**Protein**	**8 g**
Vitamin D	0 mcg
Calcium	33 mg
Iron	1 mg
Potassium	179 mg

Nutrients will display if the data is available

Please note: nutrient values are subject to change as data is updated

MyPlate Food Groups

Vegetables	1/2 cups
Grains	1 1/2 ounces

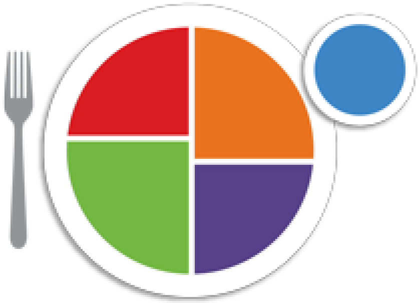

Visit MyPlate.gov

Rainbow Veggie Salad

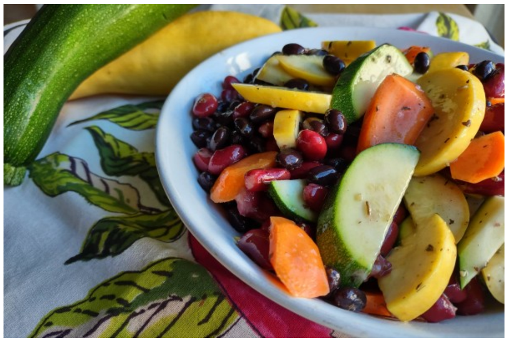

Makes: 10 servings

This easy and colorful salad tastes best after marinating it overnight. Enjoy it as a side dish at lunch or dinner.

Ingredients

- 1 can (15.5 ounces) red kidney beans, low-sodium (drained and rinsed)
- 1 can (15.5 ounces) black beans, low-sodium (drained and rinsed)
- 3 carrots (scrubbed and sliced)
- 1 medium yellow squash (washed and sliced)
- 1 medium zucchini (washed and sliced)
- 1/2 cup light Italian dressing
- 1/2 teaspoon black pepper

Directions

1. Wash hands with soap and water.
2. Mix all the vegetables together in a large bowl.
3. Pour dressing over vegetables.
4. Sprinkle with pepper.
5. Stir gently, coating all vegetables.
6. Cover and refrigerate at least 8 hours.

Source:

2009 Recipe Calendar
University of Maryland Extension
Food Supplement Nutrition Education Program

Nutrition Information

Serving Size: 2/3 cup (106g)

Nutrients	Amount
**Total Calories**	**95**
**Total Fat**	**1 g**
Saturated Fat	0 g
Cholesterol	0 mg
**Sodium**	**257 mg**
**Carbohydrates**	**18 g**
Dietary Fiber	6 g
Total Sugars	4 g
Added Sugars included	0 g
**Protein**	**6 g**
Vitamin D	0 mcg
Calcium	44 mg
Iron	2 mg
Potassium	424 mg

Nutrients will display if the data is available

Please note: nutrient values are subject to change as data is updated

MyPlate Food Groups

Vegetables	1 cups
Protein Foods	2 ounces

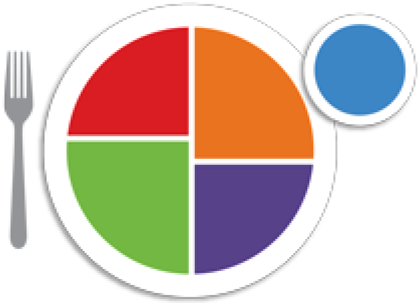

Visit MyPlate.gov

Apple-Sage Wild Rice Stuffing

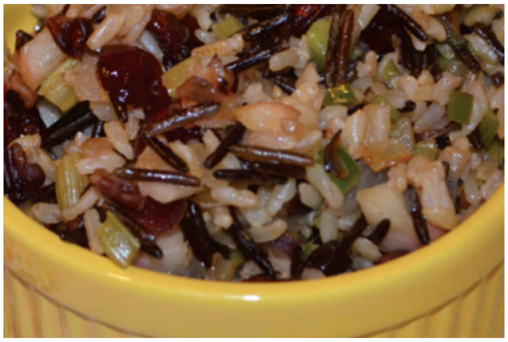

Makes: 12 Servings
Preparation Time: 25 minutes
Cook Time: 20 minutes

This tasty wild rice dish starts with sautéed celery and onions mixed with sweet apples and cranberries. This combination, topped with toasted pecans, sage and a hint of heat makes a flavorful addition to any meal.

Ingredients

- 4 ounces chopped pecans
- 4 teaspoons canola oil (divided)
- 1 1/2 cups diced celery
- 1 1/2 cups diced onions
- 1 medium red apple, such as Jonathan or Gala (halved, cored, and diced, about 1 1/2 cup total)
- 2 cups hot cooked brown rice
- 1 cup hot cooked wild rice
- 1/2 cup dried cranberries
- 1 medium jalapeno pepper (seeded (if desired) and finely chopped OR 1/4 tsp dried pepper flakes)
- 1 1/2 tablespoons chopped fresh sage (or 1 1/2 tsp dried rubbed sage)
- 3/4 teaspoon salt

Directions

1. Heat a large skillet over medium-high heat. Add the pecans and cook 2-3 minutes or until beginning to brown, stirring frequently. Set aside on separate plate.
2. Heat 1 teaspoon of the oil over medium heat. Cook the celery and onion 8 minutes or until beginning to lightly brown on edge, stirring occasionally.
3. Add the apples and cook 4 minutes or until tender crisp.
4. Stir in the pecans and the remaining ingredients and cook 3-4 minutes or until the rice mixture is heated, stirring occasionally.

Source:

Meeting Your MyPlate Goals on a Budget
MyPlate National Strategic Partners Toolkit

Nutrition Information

Nutrients	Amount
**Total Calories**	**160**
**Total Fat**	**9 g**
Saturated Fat	1 g
Cholesterol	0 mg
**Sodium**	**160 mg**
**Carbohydrates**	**20 g**
Dietary Fiber	3 g
Total Sugars	7 g
Added Sugars included	3 g
**Protein**	**3 g**
Vitamin D	0 mcg
Calcium	20 mg
Iron	1 mg
Potassium	174 mg

Nutrients will display if the data is available

Please note: nutrient values are subject to change as data is updated

MyPlate Food Groups

Fruits	1/4 cups
Vegetables	1/4 cups
Grains	1 ounce
Protein Foods	1/2 ounces

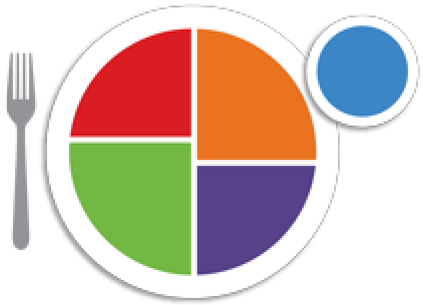

Visit MyPlate.gov

Minestrone Soup

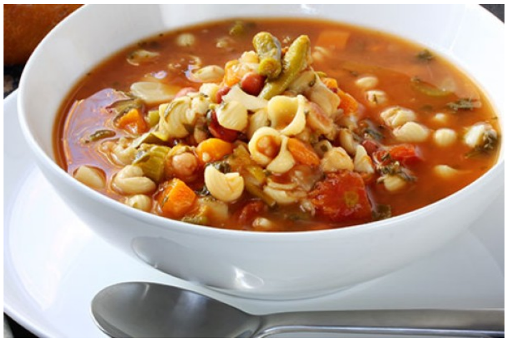

Makes: 6 servings

Prepare delicious soup in a snap! Canned beans and frozen veggies make this dish perfect for busy weeknights.

Ingredients

- 1 package (10 ounces) frozen vegetables, any type
- 2 cans (14.5 ounces each) low-sodium stewed tomatoes
- 2 cans (14.5 ounces each) low-sodium broth, any flavor
- 1 can (15.5 ounces) low-sodium beans, any type
- 1 cup dry pasta, any type

Directions

This recipe is developed for a child to help an adult in the kitchen. Directions are written to different audiences.

1. **Adult and child**: Wash hands with soap and water.
2. **Child**: In a large pot, combine frozen vegetables, tomatoes, broth and beans.
3. **Adult**: Bring the soup to a boil and add the pasta. Then reduce to low heat. Let simmer for 6 to 8 minutes or until the pasta and vegetables are tender.

Source:

Food Wise Learn at Home Print Materials
Rutgers Cooperative Extension

Nutrition Information

Serving Size: 1 cup, 1/6 of recipe

Nutrients	Amount
**Total Calories**	**144**
**Total Fat**	**1 g**
Saturated Fat	0 g
Cholesterol	0 mg
**Sodium**	**172 mg**
**Carbohydrates**	**29 g**
Dietary Fiber	9 g
Total Sugars	6 g
Added Sugars included	1 g
**Protein**	**7 g**
Vitamin D	N/A
Calcium	80 mg
Iron	3 mg
Potassium	163 mg

Nutrients will display if the data is available

Please note: nutrient values are subject to change as data is updated

MyPlate Food Groups

Vegetables	1 1/4 cups
Protein Foods	1 1/2 ounces

Visit MyPlate.gov

White Bean Bruschetta

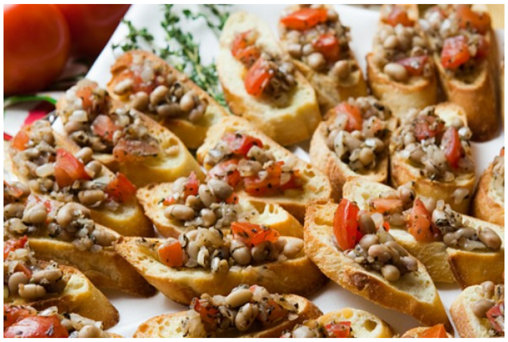

Makes: 4 Servings

Try this tasty bruschetta with crispy bread topped with white beans, tomatoes, and spices. Served as a side dish or an appetizer, it is sure to be a crowd pleaser!

Ingredients

- 1 whole-wheat French baguette, cut into 12 thin slices along the bias
- 1/4 cup olive oil, divided
- 1 cup white onion, chopped
- 4 cloves garlic, minced
- 1 teaspoon dried basil
- 1 teaspoon dried oregano
- 1 cup canned navy beans, rinsed and drained
- 2 tomatoes, cored and cubed
- 2 tablespoons balsamic vinegar

Directions

1. Slice the baguette on the diagonal into thin slices (about 12 slices for a baguette).
2. In a large sauté pan, heat 2 tablespoons of the olive oil over medium heat.
3. Place the bread slices in the pan and cook on medium high heat until sizzling and golden. Before flipping the bread, add an additional tablespoon of olive oil to the pan and cook the second side until golden.
4. For the topping, cook the onions and the remaining tablespoon of olive oil over medium heat until the onions are soft, about 7 minutes.
5. Add the garlic, basil, and oregano and cook another minute or two, until fragrant.
6. Add beans and continue cooking for another five minutes on low heat. Add the tomatoes and turn off the heat, allowing tomatoes to warm without cooking.
7. Drizzle the balsamic vinegar into the pan and gently stir.
8. Scoop heaping spoonsful of the tomato-bean mixture onto the grilled bread and enjoy.

Source:

MyPlate National Strategic Partners
The Grain Chain

Nutrition Information

Nutrients	Amount
**Total Calories**	**342**
**Total Fat**	**15 g**
Saturated Fat	2 g
Cholesterol	0 mg
**Sodium**	**332 mg**
**Carbohydrates**	**43 g**
Dietary Fiber	7 g
Total Sugars	6 g
Added Sugars included	1 g
**Protein**	**10 g**
Vitamin D	0 mcg
Calcium	95 mg
Iron	4 mg
Potassium	574 mg

Nutrients will display if the data is available

Please note: nutrient values are subject to change as data is updated

MyPlate Food Groups

Vegetables	3/4 cups
Grains	1 1/2 ounces

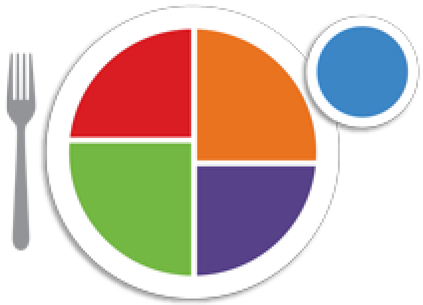

Visit MyPlate.gov

Banana Pudding

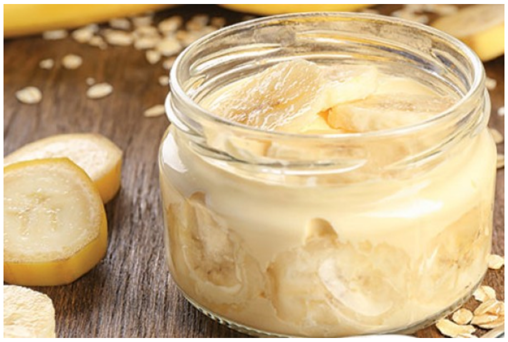

Makes: 8 servings

Dress up instant pudding mix with this dessert that combines low fat dairy and fruit for a tasty, nourishing treat.

Ingredients

- 1 (3.4 ounce) box instant pudding mix, banana or vanilla
- 2 cups fat-free (skim) milk (or 1% milk)
- 1 (8 ounce) container of fat-free plain yogurt (or non-dairy whipped topping)
- 2 medium bananas, sliced (or other sliced fruit)

Directions

1. Wash hands with soap and water.
2. In a medium bowl, combine pudding and milk. Beat with wooden spoon, wire whisk, or electric mixer on lowest speed for 2 minutes
3. Gently mix yogurt (or whipped topping) with pudding mixture. Refrigerate for 30 minutes.
4. Layer fruit slices in the bottom of 8 dessert cups.
5. Pour the pudding mixture over sliced fruit. Top with more fruit.
6. Refrigerate until ready to serve, at least 5 minutes. The taste is better if it's refrigerated longer.

Source:

Choices: Steps Toward Health, Adapted from: Lisa's Famous Dessert
University of Massachusetts
Extension Nutrition Education Program

Nutrition Information

Serving Size: 1/8 of recipe

Nutrients	Amount
**Total Calories**	**110**
**Total Fat**	**1 g**
Saturated Fat	0 g
Cholesterol	3 mg
**Sodium**	**219 mg**
**Carbohydrates**	**23 g**
Dietary Fiber	1 g
Total Sugars	20 g
Added Sugars included	11 g
**Protein**	**4 g**
Vitamin D	1 mcg
Calcium	130 mg
Iron	0 mg
Potassium	269 mg

Nutrients will display if the data is available

Please note: nutrient values are subject to change as data is updated

MyPlate Food Groups

Fruits 1/4 cups

Dairy 1/4 cups

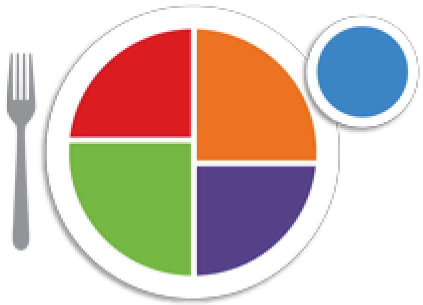

[Visit MyPlate.gov](https://www.myplate.gov)

Oven-Roasted Vegetables

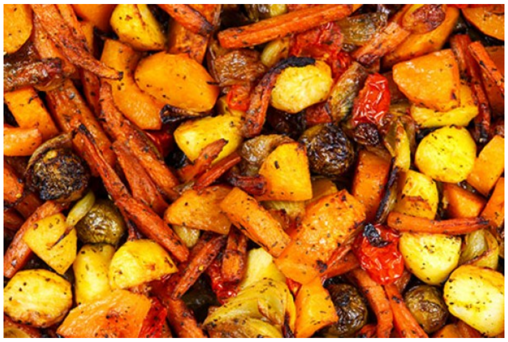

Makes: 6 servings

This simple and delicious vegetable dish is perfect with your favorite entrée.

Ingredients

- 2 tablespoons vegetable oil (or cooking oil of choice)
- 1 tablespoon lemon juice
- 1/2 teaspoon Italian seasoning
- 1/4 teaspoon salt
- 1/4 teaspoon black ground pepper
- 3 cups fresh vegetables (potatoes, broccoli, carrots, cauliflower, or red peppers)

Directions

1. Wash hands with soap and water.
2. Preheat the oven to 450 degrees F.
3. In a small bowl, mix the oil, lemon juice, Italian seasoning, salt, and pepper.
4. Wash, peel, and cut the fresh vegetables to get 3 cups cut-up vegetables.
5. Spread vegetables on baking sheet pan.
6. Pour the oil mixture over the vegetables and mix together.
7. Bake for 20 minutes. Stir after the first 10 minutes of baking.
8. Serve the vegetables while they are still hot.

Source:

Pennsylvania Nutrition Education Network

Nutrition Information

Serving Size: 1/2 cup prepared vegetables, 1/6 of recipe

Nutrients	Amount
**Total Calories**	**100**
**Total Fat**	**5 g**
Saturated Fat	1 g
Cholesterol	0 mg
**Sodium**	**129 mg**
**Carbohydrates**	**12 g**
Dietary Fiber	4 g
Total Sugars	3 g
Added Sugars included	0 g
**Protein**	**3 g**
Vitamin D	0 mcg
Calcium	25 mg
Iron	1 mg
Potassium	158 mg

Nutrients will display if the data is available

Please note: nutrient values are subject to change as data is updated

MyPlate Food Groups

Vegetables 1/2 cups

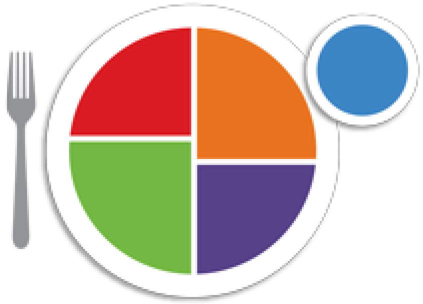

Visit MyPlate.gov

Deviled Eggs

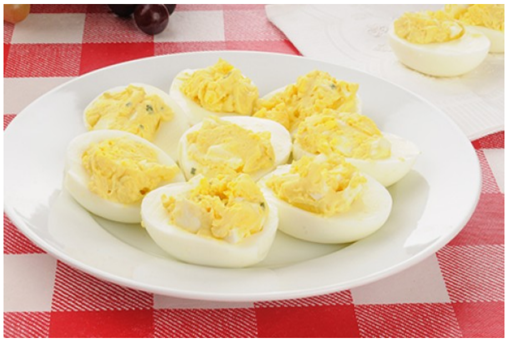

Makes: 6 servings

A favorite dish at parties and potlucks, Deviled Eggs also make a great, protein-filled anytime snack!

Ingredients

- 6 large eggs, hard-boiled and peeled
- 1/4 cup mayonnaise
- 1/8 teaspoon salt
- 1/8 teaspoon black pepper

Directions

1. Wash hands with soap and water.
2. Hard boil eggs by placing eggs in a saucepan and covering them with water. Bring to a boil.
3. Reduce heat to simmer; cook for 15 minutes.
4. Immediately rinse under cold water to stop cooking and to make it easy to peel off shells. Refrigerate peeled eggs (without shells) until ready for use.
5. Slice eggs into halves lengthwise. Remove yellow yolks and save whites.
6. Place yolks in a one-quart zip lock style bag along with the remaining ingredients (except the egg whites). Press out air.
7. Close bag and knead (mush together) until ingredients are well-blended. (Note: you could also put yolks in a bowl with other ingredients [except the egg whites] and mix together well until they look like a paste).
8. Push contents toward one corner of the bag. Cut about 1/2 inch off the corner of the bag. Squeezing the bag gently, fill reserved egg white hollows with the yolk mixture. (Note if you used a bowl, spoon the yolk mixture into the egg whites).
9. Chill to blend flavors.

Source:

Kids a Cookin'
Kansas Family Nutrition Program

Nutrition Information

Serving Size: 2 prepared egg halves, 1/6 of recipe

Nutrients	Amount
**Total Calories**	**134**
**Total Fat**	**12 g**
Saturated Fat	3 g
Cholesterol	190 mg
**Sodium**	**180 mg**
**Carbohydrates**	**0 g**
Dietary Fiber	0 g
Total Sugars	0 g
Added Sugars included	0 g
**Protein**	**6 g**
Vitamin D	1 mcg
Calcium	29 mg
Iron	1 mg
Potassium	72 mg

Nutrients will display if the data is available

Please note: nutrient values are subject to change as data is updated

MyPlate Food Groups

Protein Foods 1 ounces

Visit MyPlate.gov

Black Bean Soup

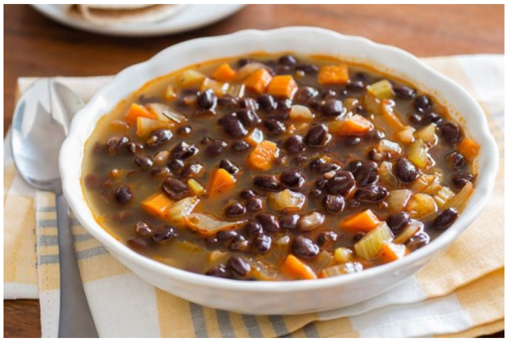

Makes: 5 Servings
Preparation Time: 20 minutes
Cook Time: 3 hours

Black beans, carrots, celery, and seasonings are simmered into this hearty soup. Bring out the delicious flavors with a splash of lime juice and spoonful of yogurt at serving.

Ingredients

- 2 tablespoons vegetable oil
- 1 Spanish onion
- 2 carrots (diced)
- 2 celery sticks (diced)
- 4 garlic cloves (peeled and minced)
- 1 teaspoon dried basil
- 1 teaspoon dried oregano
- 2 teaspoons chili powder (or more to taste)
- 3 cans (15.5 ounce) low-sodium black beans (drained and rinsed in cold water, or use 6 cups cooked (dried) black beans)
- 8 cups water
- 1 cube low sodium chicken bouillon
- 1 lime (juiced)
- plain low-fat yogurt (optional)

Directions

1. Place a soup or stock pot on the stove over medium heat and when it is hot, add the oil. Add an onion, carrots, celery, garlic, basil, oregano, and chili powder and cook about 10 minutes until the onion is soft.
2. Add the beans, water, and bouillon cube and raise the heat to high and bring to a boil. Turn the heat down to low and cook about 2 1/2 hours until the beans are very tender and the mixture is uniform in color.
3. If you want a pureed soup, set the soup aside to cool for about 20 minutes. Put 2 cups soup in a blender and put the top on, halfway (this will allow the soup to expand and any steam to escape) and blend until smooth. Transfer to a large container. Repeat using the rest of the soup.
4. Just before serving, squeeze the juice of 1/4 lime on each serving and a tablespoon of yogurt.
5. Serve right away, or cover and refrigerate up to 5 days.

Nutrition Information

Serving Size: 2.5 cups

Nutrients	Amount
**Total Calories**	**322**
**Total Fat**	**6 g**
Saturated Fat	0 g
Cholesterol	0 mg
**Sodium**	**455 mg**
**Carbohydrates**	**51 g**
Dietary Fiber	20 g
Total Sugars	4 g
Added Sugars included	0 g
**Protein**	**17 g**
Vitamin D	0 mcg
Calcium	147 mg
Iron	6 mg
Potassium	1044 mg

Nutrients will display if the data is available

Please note: nutrient values are subject to change as data is updated

MyPlate Food Groups

Vegetables 2 cups

Visit MyPlate.gov

Scrambled Tofu

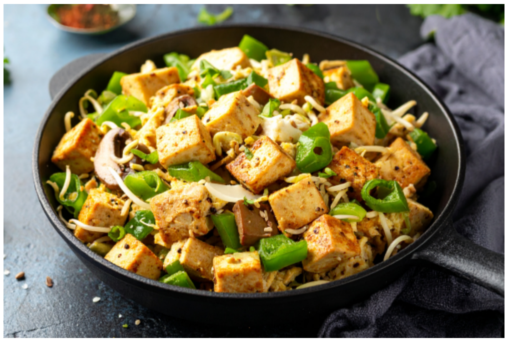

Makes: 4 servings

Scrambled tofu is a powerful protein packed dish for any meal of the day. Add your favorite veggies for increased flavor and crunch.

Ingredients

- 1 package tofu (14 ounces)
- 1 tablespoon butter
- 2 cups bean sprouts (10 ounces)
- 2 eggs
- salt and pepper (optional, to taste)

Directions

1. Wash hands with soap and water.
2. Drain tofu.
3. In small bowl, dice or mash tofu. For optional ingredients, crush garlic and/or slice watercress, mushrooms, cheese, bell peppers, and green onions.
4. In a pan, melt butter. Add tofu. Add bean sprouts and optional ingredients. Saute over medium heat until lightly browned.
5. Beat eggs and add to tofu mixture. Cook until firm.
6. Sprinkle with salt and pepper to taste.
7. Stir and cook until firm.

Source:

University of Hawaii at Manoa Cooperative Extension

Nutrition Information

Serving Size: 1/4 of recipe

Nutrients	Amount
**Total Calories**	**142**
**Total Fat**	**9 g**
Saturated Fat	3 g
Cholesterol	89 mg
**Sodium**	**47 mg**
**Carbohydrates**	**5 g**
Dietary Fiber	2 g
Total Sugars	3 g
Added Sugars included	0 g
**Protein**	**12 g**
Vitamin D	0 mcg
Calcium	218 mg
Iron	2 mg
Potassium	255 mg

Nutrients will display if the data is available

Please note: nutrient values are subject to change as data is updated

MyPlate Food Groups

Vegetables	1/2 cups
Protein Foods	2 ounces

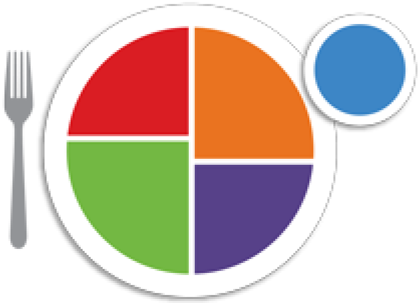

Visit MyPlate.gov

Oven-Baked Potato Pancakes

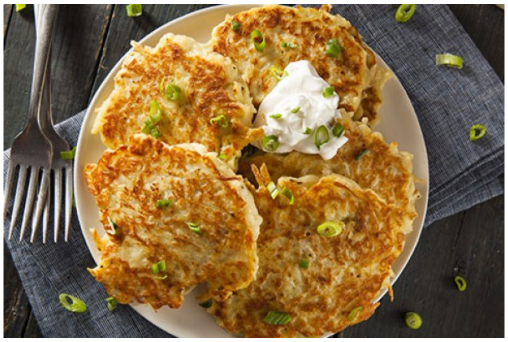

Makes: 12 Servings

These potato pancakes are easily baked in the oven and offer a healthier version of a traditional Hanukkah recipe.

Ingredients

- 2 tablespoons olive oil (or cooking oil of your choice)
- 3 tablespoons whole wheat flour (or all-purpose flour)
- 1 teaspoon baking powder
- 3/4 teaspoon salt
- 2 large russet potatoes (or Yukon Gold potatoes)
- 1 onion, small peeled
- 1 large egg
- applesauce and yogurt (plain low-fat or Greek) (optional)

Directions

1. Wash hands with soap and water.
2. Preheat the oven to 425 °F. Lightly oil the baking sheets by spreading the oil around with your clean hand or a paper towel.
3. Mix the flour, baking powder, and salt in a small bowl.
4. Use the large holes on your grater to grate the potato, then grate the onion too.
5. Put the potato and onion mixture in the colander inside the sink. Using a paper towel, press the potato mixture down to squeeze out and blot up some of the extra moisture. Stir it and blot again.
6. Put the potato mixture in the large bowl, add the egg, and stir well. Add the flour mixture and stir it very well.
7. Use a spoon or your fingers to pluck a clump of the potato mixture from the bowl and spread it into a round, flat nest on the oiled baking sheet: it should make a circle that's about 3 inches wide and 1/4 inch thick. Repeat to fill the sheet. (You don't need to leave space between them.)
8. Bake until the bottoms are deeply golden, 15 to 20 minutes, then turn the pancakes over and put them back in the over for 10 more minutes.
9. Serve the latkes with applesauce and yogurt (optional).

Source:

ChopChop Family

Nutrition Information

Serving Size: 1 latke, 1/12 of recipe (76g)

Nutrients	Amount
**Total Calories**	**83**
**Total Fat**	**3 g**
Saturated Fat	0 g
Cholesterol	16 mg
**Sodium**	**196 mg**
**Carbohydrates**	**13 g**
Dietary Fiber	2 g
Total Sugars	1 g
Added Sugars included	0 g
**Protein**	**2 g**
Vitamin D	0 mcg
Calcium	35 mg
Iron	1 mg
Potassium	285 mg

Nutrients will display if the data is available

Please note: nutrient values are subject to change as data is updated

MyPlate Food Groups

Vegetables 1/2 cups

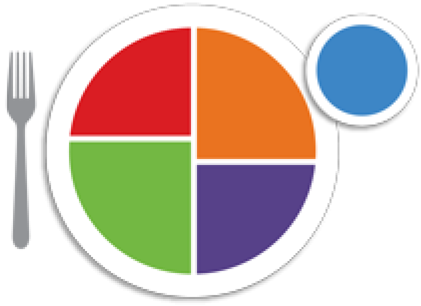

[Visit MyPlate.gov](https://www.myplate.gov)

Tomato and Cucumber Salad

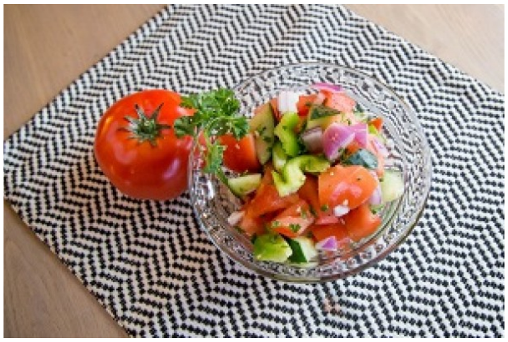

Makes: 12 Servings

This refreshing salad is an excellent source of vitamin C and a great way to take advantage of the vegetables' summer growing season.

Ingredients

Salad

- 4 large tomatoes (cubed)
- 1 large cucumber (chopped)
- 1 cup red onion (chopped)
- 1 cup green pepper (chopped)
- 1/3 cup parsley (chopped)

Dressing

- 1/3 cup apple cider vinegar
- 1 tablespoon olive oil (or oil of your choice)
- 2 cloves garlic (minced)
- 1/2 teaspoon salt
- 1/2 teaspoon ground black pepper
- 1/2 teaspoon sugar

Directions

1. Wash hands with soap and water.
2. In a large bowl, combine the salad ingredients.
3. In a small bowl, mix the dressing ingredients together.
4. Pour the dressing over the salad. Mix well.
5. Refrigerate for at least 1 hour before serving.

Source:

University of Nebraska at Lincoln
Nebraska Nutrition Education Program

Nutrition Information

Serving Size: 3/4 cup

Nutrients	Amount
**Total Calories**	**34**
**Total Fat**	**1 g**
Saturated Fat	0 g
Cholesterol	0 mg
**Sodium**	**103 mg**
**Carbohydrates**	**5 g**
Dietary Fiber	1 g
Total Sugars	3 g
Added Sugars included	0 g
**Protein**	**1 g**
Vitamin D	0 mcg
Calcium	17 mg
Iron	0 mg
Potassium	225 mg

Nutrients will display if the data is available

Please note: nutrient values are subject to change as data is updated

MyPlate Food Groups

Vegetables 3/4 cups

Visit MyPlate.gov

Grilled Vegetables

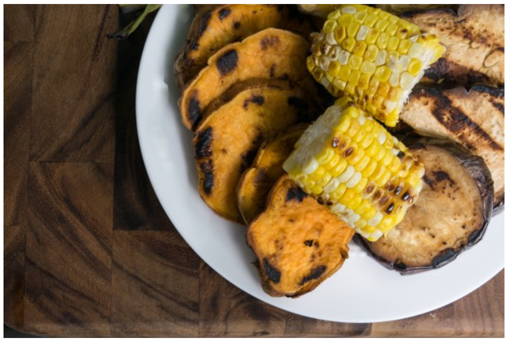

Makes: 6 servings

Cooking out tonight? Try cooking vegetables coated in oil and garlic on the grill for a delicious side dish. If you don't have a grill, you can make these vegetables in the oven.

Ingredients

- 2 tablespoons vegetable oil
- 2 garlic cloves (finely chopped)
- 3 sweet potatoes (cut into 1-inch slices)
- 3 corn cobs (cut into 2-inch sections)
- 1 eggplant (cut into 1/2-inch slices)
- 12 green onions (trimmed)

Directions

1. Wash hands with soap and water.
2. Mix oil and garlic in a large bowl. Add vegetables and toss.
3. Place vegetable on broiler pan or grill. Cook 10 minutes, turning twice until vegetables are tender.
4. Place vegetables on platter. Serve.

Source:

US Department of Health and Human Services
Centers for Disease Control and Prevention (CDC)

Nutrition Information

Serving Size: 1/6 of recipe (254g)

**Nutrients**	**Amount**
**Total Calories**	**149**
**Total Fat**	**5 g**
Saturated Fat	1 g
Cholesterol	0 mg
**Sodium**	**48 mg**
**Carbohydrates**	**25 g**
Dietary Fiber	4 g
Total Sugars	7 g
Added Sugars included	0 g
**Protein**	**3 g**
Vitamin D	0 mcg
Calcium	45 mg
Iron	1 mg
Potassium	459 mg

Nutrients will display if the data is available

Please note: nutrient values are subject to change as data is updated

MyPlate Food Groups

Vegetables 1 cups

Visit MyPlate.gov

Angel Food Pastry with Fresh Berries and Whipped Cream

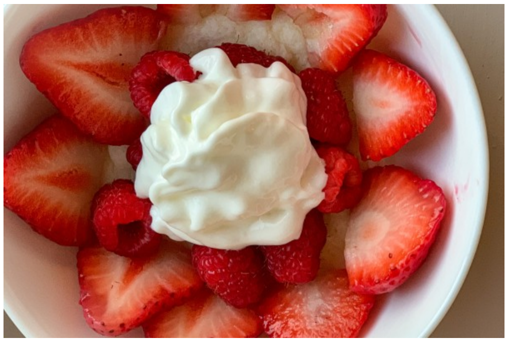

Makes: 1 Servings

Light and fluffy angel food cake is mixed with yogurt and a sprinkle of berries on top.

Ingredients

- 1 ounce angel food cake mix (3 tablespoons)
- 1/8 cup low-fat vanilla yogurt (2 tablespoons)
- 1/4 cup raspberries
- 2 strawberries, sliced
- 1 1/2 tablespoons lite whipped topping

Directions

1. Wash hands with soap and water.
2. Scoop 3 tablespoons of angel food cake mix into a microwave-safe cup or mug.
3. Add 2 tablespoons of vanilla yogurt and stir.
4. Place in the center of the microwave oven and microwave for 1 minute.
5. Take out of microwave and let cool for 1 minute.
6. Using a knife, cut around the sides of the cup to loosen the cake and "dump" it on a plate.
7. Dole 1 tablespoon of whipped topping on top of the cake.
8. Sprinkle raspberries around the angel cake and add sliced strawberries to top.
9. Enjoy.

Source:

Community Services for Autistic Adults and Children (CSAAC)

Nutrition Information

Serving Size: 1 Angel Food Pastry

Nutrients	Amount
**Total Calories**	**174**
**Total Fat**	**2 g**
Saturated Fat	1 g
Cholesterol	2 mg
**Sodium**	**260 mg**
**Carbohydrates**	**37 g**
Dietary Fiber	3 g
Total Sugars	27 g
Added Sugars included	10 g
**Protein**	**4 g**
Vitamin D	0 mcg
Calcium	116 mg
Iron	0 mg
Potassium	204 mg

Nutrients will display if the data is available

Please note: nutrient values are subject to change as data is updated

MyPlate Food Groups

Fruits 1/2 cups

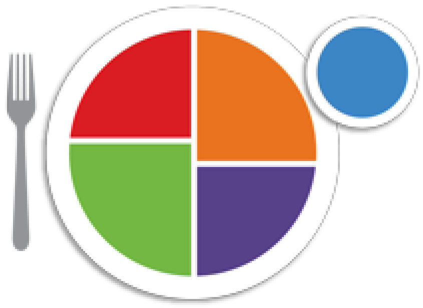

Visit MyPlate.gov

Tropical Yogurt Pops

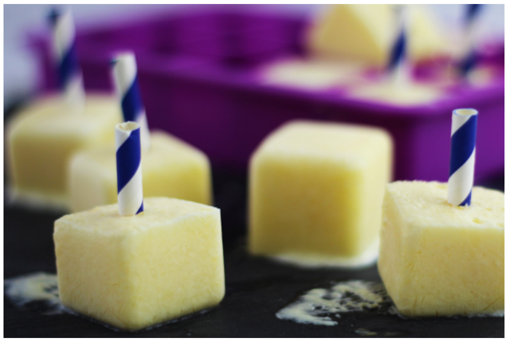

Makes: 4 Servings

Enjoy a tropical treat with these frozen yogurt pops! They are fun to make and have 1/4 cup of fruit per serving!

Ingredients

- 1 cup low-fat vanilla yogurt
- 1/2 cup mango pieces (fresh or frozen)
- 1/2 cup pineapple pieces (fresh, canned, or frozen)
- 1/4 cup cup coconut water
- 1 ice cube tray (or paper cups)

Directions

1. Place all ingredients into blender.
2. Blend on high until smooth.
3. Pour into 4 small paper cups (or 8 ice cubes) and place in popsicle sticks (or cut paper straws).
4. Freeze.
5. Enjoy as a frozen treat!

Source:

USDA Center for Nutrition Policy and Promotion

Nutrition Information

Nutrients	Amount
**Total Calories**	**65**
**Total Fat**	**1 g**
Saturated Fat	1 g
Cholesterol	4 mg
**Sodium**	**43 mg**
**Carbohydrates**	**11 g**
Dietary Fiber	1 g
Total Sugars	10 g
Added Sugars included	0 g
**Protein**	**4 g**
Vitamin D	0 mcg
Calcium	N/A
Iron	N/A
Potassium	N/A

Nutrients will display if the data is available

Please note: nutrient values are subject to change as data is updated

MyPlate Food Groups

Fruits 1/4 cups
Dairy 1/4 cups

Visit MyPlate.gov

Chocolate Chip Yogurt Cookies

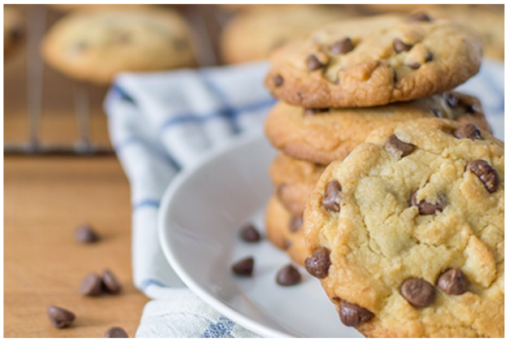

Makes: 36 servings

Add extra calcium to your cookies with yogurt. These cookies also use whole wheat flour which adds fiber and other nutrients.

Ingredients

- 1/2 cup sugar
- 1/2 cup brown sugar, firmly packed
- 1/2 cup margarine (1 stick)
- 1/2 cup plain yogurt, non-fat
- 1 1/2 teaspoons vanilla
- 3/4 cup all-purpose flour
- 1 cup whole wheat flour
- 1/2 teaspoon baking soda
- 1/2 cup chocolate chips (miniature, or carob chips)

Directions

1. Wash hands with soap and water.
2. Heat oven to 375 °F.
3. In a large bowl combine sugar, brown sugar and margarine; beat until light and fluffy.
4. Add yogurt and vanilla; blend well. Stir in flour and baking soda; mix well. Stir in chocolate chips.
5. Drop dough by rounded teaspoonfuls 2 inches apart onto un-greased cookie sheets. Bake at 375 °F for 8 to 12 minutes or until light and golden brown.
6. Cool 1 minute, remove from cookie sheets.

Source:

Eat Smart New York!
Cornell University Cooperative Extension

Nutrition Information

Serving Size: 1 cookie

Nutrients	Amount
**Total Calories**	**79**
**Total Fat**	**3 g**
Saturated Fat	1 g
Cholesterol	0 mg
**Sodium**	**42 mg**
**Carbohydrates**	**12 g**
Dietary Fiber	1 g
Total Sugars	7 g
Added Sugars included	7 g
**Protein**	**1 g**
Vitamin D	0 mcg
Calcium	12 mg
Iron	0 mg
Potassium	37 mg

Nutrients will display if the data is available

Please note: nutrient values are subject to change as data is updated

MyPlate Food Groups

Grains 1/2 ounces

Visit MyPlate.gov

Apple-Stuffed Squash

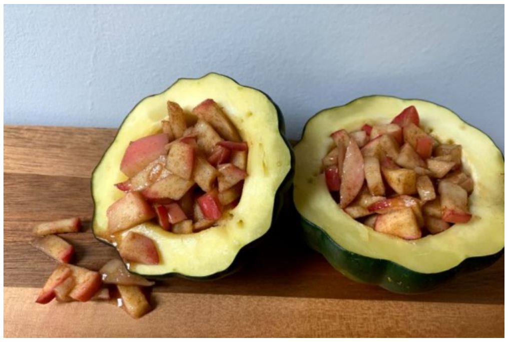

Makes: 8 Servings

All you need is a microwave to cook this delicious and seasonal side dish.

Ingredients

- 4 acorn squashes, washed
- 1 tablespoon butter (or margarine)
- 2 apples, chopped
- 2 teaspoons brown sugar, packed
- 1/2 teaspoon cinnamon

Directions

1. Wash hands with soap and water.
2. Cut squash into halves and remove seeds. Place in a glass dish, cover with plastic wrap and microwave on high for 5 minutes.
3. Melt butter in a separate bowl in the microwave. Mix in apples, sugar, and cinnamon. Microwave for 1 1/2 minutes.
4. Spoon apple filling into each squash half. Cover and microwave on high for 3-5 minutes until squash and apples are tender.
5. Serve warm.

Source:

University of Maryland Cooperative Extension
SNAP-Ed Program

Nutrition Information

Serving Size: 1/2 squash with filling, 1/8 of recipe

Nutrients	Amount
**Total Calories**	**122**
**Total Fat**	**2 g**
Saturated Fat	1 g
Cholesterol	4 mg
**Sodium**	**7 mg**
**Carbohydrates**	**28 g**
Dietary Fiber	4 g
Total Sugars	5 g
Added Sugars included	1 g
**Protein**	**2 g**
Vitamin D	0 mcg
Calcium	76 mg
Iron	2 mg
Potassium	787 mg

Nutrients will display if the data is available

Please note: nutrient values are subject to change as data is updated

MyPlate Food Groups

Fruits	1/4 cups
Vegetables	2 cups

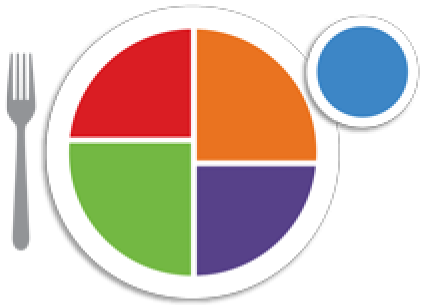

Visit MyPlate.gov

Grilled Fruit

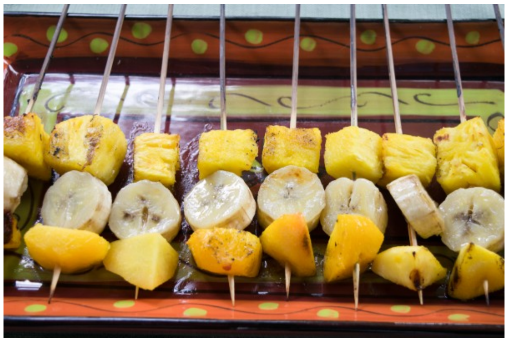

Makes: 3 Servings

Fruit kabobs cooked on the grill make a tasty and healthy dessert.

Ingredients

- 1 cup pineapple chunks (fresh or canned)
- 1 peach (cubed)
- 1 banana (sliced)

Directions

1. Wash hands with soap and water.
2. Place fruit chunks on a skewer to make kabobs.
3. Grill or broil on low heat until the fruit is hot and slightly golden.

Source:

University of Wyoming Extension
Cent$ible Nutrition Program

Nutrition Information

Serving Size: 1/3 of recipe

Nutrients	Amount
**Total Calories**	**104**
**Total Fat**	**0 g**
Saturated Fat	0 g
Cholesterol	0 mg
**Sodium**	**1 mg**
**Carbohydrates**	**27 g**
Dietary Fiber	2 g
Total Sugars	21 g
Added Sugars included	0 g
**Protein**	**1 g**
Vitamin D	0 mcg
Calcium	17 mg
Iron	0 mg
Potassium	337 mg

Nutrients will display if the data is available

Please note: nutrient values are subject to change as data is updated

MyPlate Food Groups

Fruits 1 cups

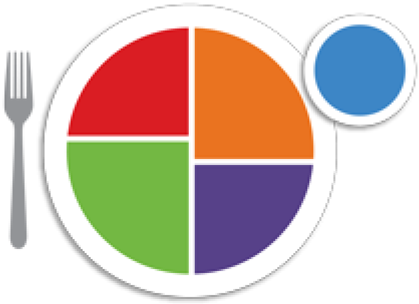

Visit MyPlate.gov

Grilled Vegetable Packets

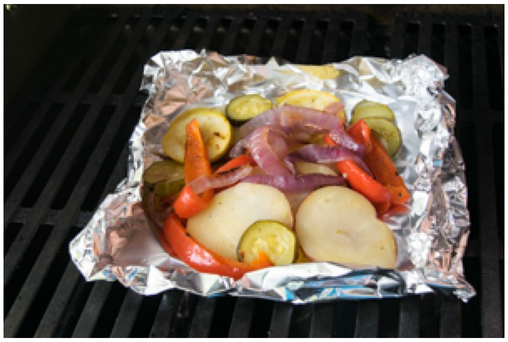

Makes: 5 servings

Veggies are wrapped in foil and cooked on the grill for an easy side dish to your cookout. Don't have a grill? This recipe can also be made in the oven.

Ingredients

- 2 small zucchinis (sliced)
- 2 small yellow squashes (sliced)
- 4 small red potatoes (scrubbed well and sliced)
- 1/2 red onion (sliced)
- 1/2 red or green bell pepper (sliced)
- 1/4 cup light Italian salad dressing
- salt and pepper (optional, to taste)

Directions

1. Wash hands with soap and water.
2. Heat grill to medium heat or 350 °F.
3. Wash vegetables and slice.
4. Toss in a large bowl. Add dressing and toss until all vegetables are coated.
5. Tear 2 large squares of aluminum foil and place half of the vegetable mixture on each piece. Place an equal piece of foil over the top of vegetable mixture and fold bottom piece with top sheet to form a packet.
6. Place on heated grill for 20 to 30 minutes or until the potatoes are tender. If you don't have a grill, bake Veggie Packets in the oven at 400 °F for 20 to 30 minutes.
7. Before you open the packets, poke holes in the foil with a fork. Be very careful opening the foil as the steam will be very hot and could burn you!
8. Empty vegetables onto serving plate or serve from foil packets.

Source:

Connecticut Food Policy Council

Nutrition Information

Serving Size: 3/4 cup (316g)

Nutrients	Amount
**Total Calories**	**133**
**Total Fat**	**0 g**
Saturated Fat	0 g
Cholesterol	0 mg
**Sodium**	**144 mg**
**Carbohydrates**	**29 g**
Dietary Fiber	4 g
Total Sugars	5 g
Added Sugars included	0 g
**Protein**	**4 g**
Vitamin D	0 mcg
Calcium	38 mg
Iron	2 mg
Potassium	868 mg

Nutrients will display if the data is available

Please note: nutrient values are subject to change as data is updated

MyPlate Food Groups

Vegetables 2 cups

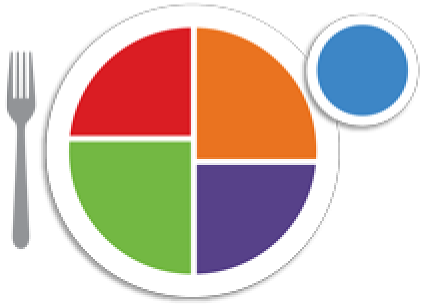

Visit MyPlate.gov

Skinny Pizza

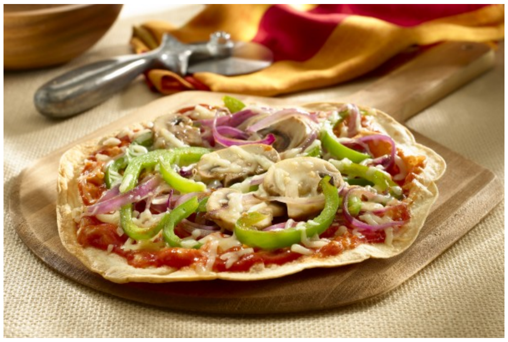

Makes: 4 Servings
Preparation Time: 30 minutes

Next time they ask for pizza, you can feel good about saying "yes!" Flour tortillas make for a crispy crust, perfect for loading with low-sodium tomato sauce, low-fat cheese, and lots of fresh veggies.

Ingredients

- 4 6" flour tortillas
- 1/2 teaspoon extra virgin olive oil
- 2 cups sliced mushrooms (white button or baby Portobello)
- 1 green bell pepper (thinly sliced, about 1 cup)
- 1 red onion (thinly sliced, about 1 cup)
- 2 teaspoons minced garlic
- 1/2 cup low-sodium tomato sauce
- 1/2 cup shredded fat-free mozzarella cheese
- 2 teaspoons grated reduced-fat Parmesan cheese

Directions

1. Heat oven to 400 °F.
2. Place tortillas on 2 large baking sheets.
3. Cook, flipping once, until crisp (about 10 minutes). Set aside.
4. Meanwhile, heat oil in large skillet over medium heat. Add mushrooms, peppers, onions, and garlic.
5. Cook until all vegetables are soft and tender (about 10 minutes). Set aside.
6. Spread tortilla crust with 2 tablespoons tomato sauce, 1/4 cup vegetable mixture, 2 tablespoons mozzarella cheese, and 1/2 teaspoon of Parmesan cheese. Repeat with remaining crusts and topping ingredients.
7. Transfer pizzas to same baking sheets.
8. Cook until cheese is melted and edges of tortillas are golden brown (about 10 minutes).

Source:

The Best of La Cocina GOYA: Healthy, Tasty, Affordable Latin Cooking/ Lo Mejor de la Cocina GOYA: Cocina Latina Saludable, Rica y Económica
GOYA Foods

Nutrition Information

Nutrients	Amount
**Total Calories**	**190**
**Total Fat**	**5 g**
Saturated Fat	1 g
Cholesterol	5 mg
**Sodium**	**450 mg**
**Carbohydrates**	**26 g**
Dietary Fiber	3 g
Total Sugars	6 g
Added Sugars included	2 g
**Protein**	**9 g**
Vitamin D	0 mcg
Calcium	200 mg
Iron	2 mg
Potassium	375 mg

Nutrients will display if the data is available

Please note: nutrient values are subject to change as data is updated

MyPlate Food Groups

Vegetables	1 1/4 cups
Grains	1 1/2 ounces
Dairy	1/2 cups

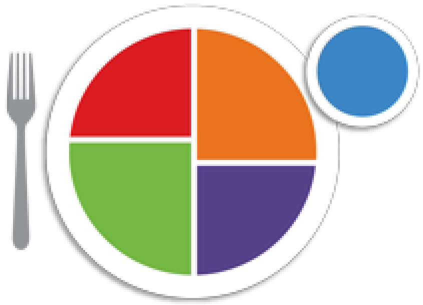

Visit MyPlate.gov

Rice-Crusted Pizza

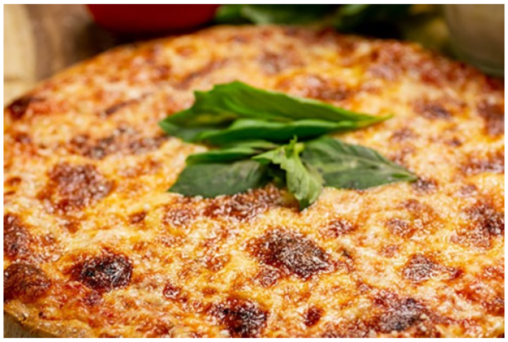

Makes: 12 servings

A fun and unique twist on pizza using rice as the crust. Enjoy this main dish with all of your favorite toppings!

Ingredients

- 2 cups rice (cooked)
- 16 ounces mozzarella cheese, part skim
- 1 egg
- 1/4 teaspoon salt
- 2 cups tomato purée, low-sodium

Directions

1. Wash hands with soap and water.
2. Heat oven to 350 °F. Grease a 12-inch pizza pan or baking sheet.
3. Cook rice following directions on the package; set aside.
4. Grate cheese; set aside.
5. Crack egg and place in mixing bowl, stirring to blend. Measure and add 2 cups cooked rice, 1 cup grated cheese, and salt. Mix well to combine ingredients.
6. Spread rice mixture in prepared pan, pressing firmly and making outer edge slightly raised.
7. Spread tomato purée evenly over rice mixture.
8. Place pan in oven. Bake for 25 minutes.
9. Remove pan from oven sprinkle pizza with remaining cheese. Bake 5 minutes longer or until cheese melts.
10. Remove pizza from oven. Cut into 12 slices.

Create-a-Flavor Changes:

- Try different kinds of rice.
- Try different kinds of cheese.
- Use sliced or chopped green peppers, onions, mushrooms, or other vegetables as toppings.

Source:

Cooking Up Fun - A Pyramid of Snacks
Cornell University Cooperative Extension

Nutrition Information

Serving Size: 1 slice, 1/12 of recipe (110g)

Nutrients	Amount
**Total Calories**	**175**
**Total Fat**	**9 g**
Saturated Fat	4 g
Cholesterol	37 mg
**Sodium**	**313 mg**
**Carbohydrates**	**12 g**
Dietary Fiber	1 g
Total Sugars	2 g
Added Sugars included	3 g
**Protein**	**12 g**
Vitamin D	0 mcg
Calcium	292 mg
Iron	1 mg
Potassium	181 mg

Nutrients will display if the data is available

Please note: nutrient values are subject to change as data is updated

MyPlate Food Groups

Vegetables	1/4 cups
Grains	1/2 ounces
Dairy	1 cups

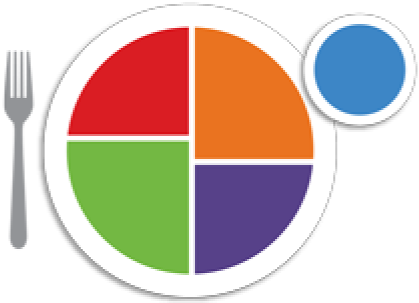

Visit MyPlate.gov

Curried Chickpea Salad

Makes: 6 Servings
Preparation Time: 10 minutes

This dish can be served on whole wheat bread, on a bed of lettuce, or as a dip with your favorite veggies!

Ingredients

- 2 cans (15 ounces each) chickpeas, low-sodium, rinsed and drained
- 1/2 cup celery, diced
- 1/2 cup apple, diced
- 1/4 cup red onion, diced
- 1/4 cup cranberries, dried
- 1/4 cup mayonnaise, low fat
- 1 teaspoon dijon mustard
- 2 teaspoons curry powder
- 1/4 teaspoon thyme, dried
- 1/8 teaspoon black pepper, ground
- salt (1/2 teaspoon, optional)

Directions

1. Wash hands with soap and water.
2. Add the chickpeas to a large bowl and mash with a potato masher. You can mash them to be as smooth or coarse as you desire.
3. Add all of the remaining ingredients to the bowl and mix until everything is combined.

Source:

Penn State Extension
Nutrition Links Program

Nutrition Information

Serving Size: 1/2 cup

Nutrients	Amount
**Total Calories**	**181**
**Total Fat**	**6 g**
Saturated Fat	1 g
Cholesterol	N/A
**Sodium**	**282 mg**
**Carbohydrates**	**26 g**
Dietary Fiber	7 g
Total Sugars	8 g
Added Sugars included	3 g
**Protein**	**7 g**
Vitamin D	N/A
Calcium	59 mg
Iron	2 mg
Potassium	N/A

Nutrients will display if the data is available

Please note: nutrient values are subject to change as data is updated

MyPlate Food Groups

Fruits	1/4 cups
Vegetables	1 cups
Protein Foods	3 ounces

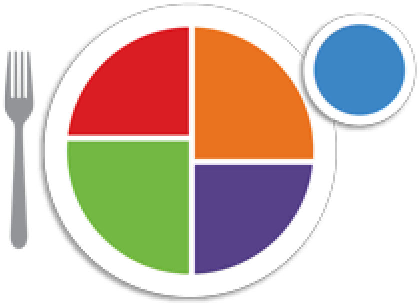

Visit MyPlate.gov

Roasted Chickpeas (Garbanzo Beans)

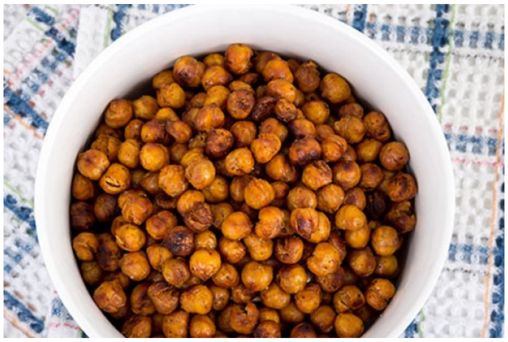

Makes: 4 Servings
Cook Time: 45 minutes

These crunchy chickpeas make a great on-the-go snack or a healthy topping to your favorite salad. Experiment with different spices to see which ones you like best.

Ingredients

- 1 can (15 ounces) low-sodium garbanzo beans, drained and rinsed
- 1 tablespoon canola oil (or cooking oil of choice)
- 1/8 teaspoon salt
- spices of your choice, optional (try garlic, paprika, sage, etc.)

Directions

1. Wash hands with soap and water.
2. Preheat oven to 400 degrees F.
3. Drain and rinse the beans until there is no foamy residue. Pick out the skins and discard. Wrap the drained beans in a clean towel to dry thoroughly.
4. Measure oil into a 9x13-inch baking dish or foil lined pan. Pour beans into dish, sprinkle with salt and spices. Mix oil and spices well to coat all the beans.
5. Bake for 45 to 60 minutes, stirring or shaking every 15 minutes. Longer makes more crunch, but watch to prevent burning!

Source:

USDA Supplemental Nutrition Assistance Program (SNAP)

Nutrition Information

Serving Size: 1/4 cup

Nutrients	Amount
**Total Calories**	**124**
**Total Fat**	**5 g**
Saturated Fat	0 g
Cholesterol	0 mg
**Sodium**	**216 mg**
**Carbohydrates**	**14 g**
Dietary Fiber	5 g
Total Sugars	3 g
Added Sugars included	0 g
**Protein**	**5 g**
Vitamin D	0 mcg
Calcium	37 mg
Iron	1 mg
Potassium	153 mg

Nutrients will display if the data is available

Please note: nutrient values are subject to change as data is updated

MyPlate Food Groups

Vegetables	1/2 cups
Protein Foods	2 1/2 ounces

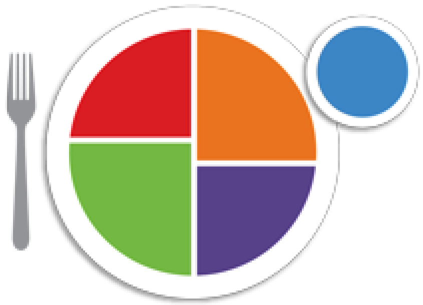

Visit MyPlate.gov

Heavenly Deviled Eggs

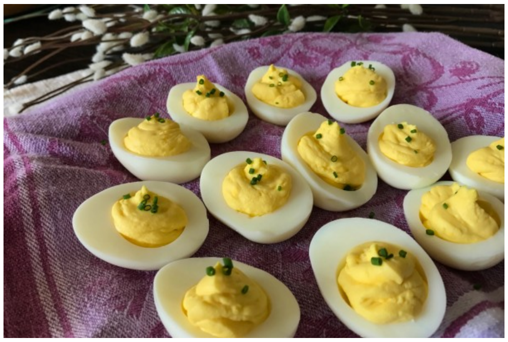

Makes: 6 Servings

Versatile and easy to prepare, these deviled eggs are great as an appetizer, side dish, or snack.

Ingredients

- 6 eggs (in shell)
- 2 tablespoons light mayonnaise
- 1 teaspoon mustard

Directions

1. Wash hands with soap and water.
2. Put eggs into a saucepan. Cover with cold water.
3. Bring eggs to a simmer (small bubbles) and cook for 12 minutes.
4. Remove from the heat and drain.
5. Crack eggs under cold water and allow to cool. Remove shells.
6. Split eggs in half, lengthwise and remove yolks.
7. Put yolks, dressing and mustard into a small zipper-lock plastic bag to mix.
8. Cut a small hole in a lower corner of the bag. Squeeze mixture into egg white halves. Garnish as desired.

Source:

Simple Healthy Recipes
Oklahoma Nutrition Information and Education
ONIE Project

Nutrition Information

Serving Size: 2 prepared egg halves, 1/6 of recipe

Nutrients	Amount
**Total Calories**	**90**
**Total Fat**	**6 g**
Saturated Fat	2 g
Cholesterol	212 mg
**Sodium**	**174 mg**
**Carbohydrates**	**1 g**
Dietary Fiber	0 g
Total Sugars	1 g
Added Sugars included	0 g
**Protein**	**6 g**
Vitamin D	40 mcg
Calcium	25 mg
Iron	1 mg
Potassium	65 mg

Nutrients will display if the data is available

Please note: nutrient values are subject to change as data is updated

MyPlate Food Groups

Protein Foods 1 ounce

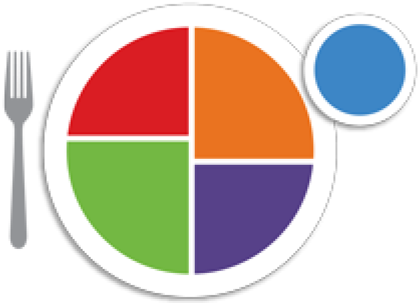

Visit MyPlate.gov

Black Bean Quesadillas

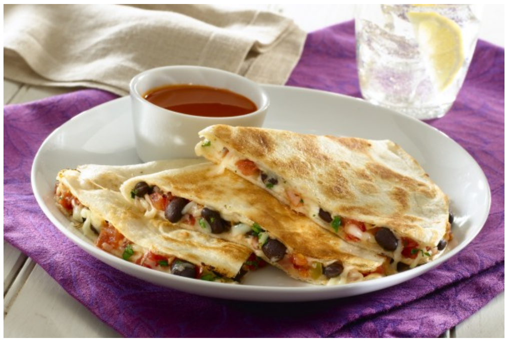

Makes: 8 Servings
Preparation Time: 15 minutes

Enjoy a quesadilla any night of the week! This quesadilla recipe can be served as a tasty lunch, dinner, or side dish.

Ingredients

- 3/4 cup chunky salsa (or pico de gallo)
- 1 can (15.5 oz) low-sodium black beans (drained and rinsed)
- 2 cups shredded reduced-fat Colby & Monterey Jack cheese
- 2 tablespoons fresh cilantro (finely chopped)
- 4 8 inch flour tortillas
- 1/2 teaspoon extra virgin olive oil

Directions

1. Using a small-hole strainer, drain liquid from salsa; discard liquid.
2. Transfer leftover tomato mixture to a medium bowl.
3. Mix in black beans, cheese, and cilantro until combined.
4. Divide black bean mixture evenly over half of each tortilla (about 1/2 cup each).
5. Fold tortillas in half.
6. Heat large griddle or skillet over medium-high heat.
7. Brush with oil.
8. Place filled tortillas on a griddle.
9. Cook, carefully flipping once, until tortillas are gold brown and crisp and cheese filling melts, about 5 minutes.
10. Cut quesadillas into wedges.

Source:

The Best of La Cocina GOYA: Healthy, Tasty, Affordable Latin Cooking/ Lo Mejor de la Cocina GOYA: Cocina Latina Saludable, Rica y Económica
GOYA Foods

Nutrition Information

Nutrients	Amount
**Total Calories**	**160**
**Total Fat**	**5 g**
Saturated Fat	2 g
Cholesterol	5 mg
**Sodium**	**490 mg**
**Carbohydrates**	**25 g**
Dietary Fiber	4 g
Total Sugars	1 g
Added Sugars included	0 g
**Protein**	**7 g**
Vitamin D	0 mcg
Calcium	96 mg
Iron	2 mg
Potassium	287 mg

Nutrients will display if the data is available

Please note: nutrient values are subject to change as data is updated

MyPlate Food Groups

Vegetables	1/4 cups
Grains	1 ounce

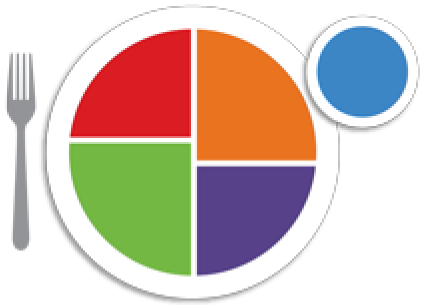

Visit MyPlate.gov

Broccoli Potato Soup

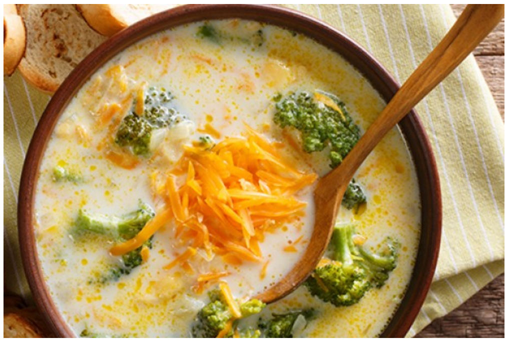

Makes: 4 servings

A tasty and made-from-scratch soup that comes together quickly with the help of instant mashed potatoes and powdered milk.

Ingredients

- 4 cups broccoli, chopped
- 1 small onion, chopped
- 4 cups chicken or vegetable broth, low-sodium
- 1 cup non-fat evaporated milk
- 1/2 cup instant mashed potatoes, prepare with water for 1 cup potatoes (or 1 cup leftover mashed potatoes.)
- salt and pepper (to taste, optional)
- 1/4 cup cheddar cheese, shredded

Directions

1. Wash hands with soap and water.
2. Combine broccoli, onion, and broth in large sauce pan.
3. Bring to a boil.
4. Reduce heat. Cover and simmer about 10 minutes or until vegetables are tender.
5. Add milk to soup. Slowly stir in potatoes.
6. Cook, stirring constantly, until bubbly and thickened.
7. Season with salt and pepper; stir in a little more milk or water if soup starts to become too thick.
8. Ladle into serving bowls.
9. Sprinkle about 1 tablespoon cheese over each serving.

Source:

Don't Play With Your Food: Fall and Winter Cookbook
Arizona Nutrition Network

Nutrition Information

Serving Size: 1/4 of recipe

Nutrients	Amount
**Total Calories**	**178**
**Total Fat**	**4 g**
Saturated Fat	2 g
Cholesterol	10 mg
**Sodium**	**225 mg**
**Carbohydrates**	**23 g**
Dietary Fiber	3 g
Total Sugars	10 g
Added Sugars included	0 g
**Protein**	**15 g**
Vitamin D	1 mcg
Calcium	295 mg
Iron	2 mg
Potassium	806 mg

Nutrients will display if the data is available

Please note: nutrient values are subject to change as data is updated

MyPlate Food Groups

Vegetables	1 1/2 cups
Dairy	3/4 cups

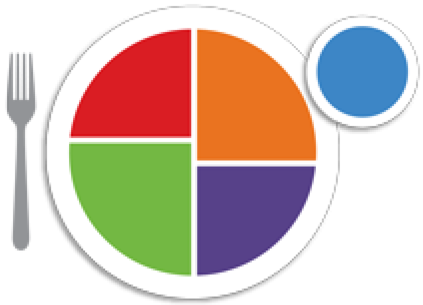

Visit MyPlate.gov

Overnight Oatmeal with Berries

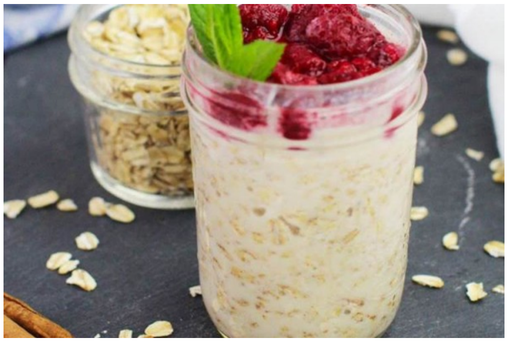

Makes: 1 Serving
Preparation Time: 15 minutes

A mixture of milk, yogurt, and raspberries combine with dry oats to become a smooth make-ahead breakfast. Just mix and refrigerate overnight for the next day.

Ingredients

- 1/2 cup low-fat milk (or less for thicker oatmeal)
- 1/4 cup Greek yogurt, fat-free
- 2 teaspoons honey
- 1/4 teaspoon cinnamon
- 1/4 teaspoon vanilla extract
- 1/2 cup uncooked rolled oats
- 1/4 cup raspberries, frozen

Directions

1. Combine milk, Greek yogurt, sugar, cinnamon, and vanilla extract in a container or jar with a lid.
2. Add oats and mix well.
3. Gently fold in raspberries.
4. Cover and refrigerate 8 hours to overnight.
5. Enjoy cold or heat as desired.

Source:

USDA Center for Nutrition Policy and Promotion

Nutrition Information

Serving Size: 1 serving

Nutrients	Amount
**Total Calories**	**311**
**Total Fat**	**4 g**
Saturated Fat	1 g
Cholesterol	7 mg
**Sodium**	**86 mg**
**Carbohydrates**	**53 g**
Dietary Fiber	9 g
Total Sugars	21 g
Added Sugars included	11 g
**Protein**	**17 g**
Vitamin D	1 mcg
Calcium	268 mg
Iron	2 mg
Potassium	513 mg

Nutrients will display if the data is available

Please note: nutrient values are subject to change as data is updated

MyPlate Food Groups

Fruits	1/4 cups
Grains	1 1/2 ounces
Dairy	3/4 cups

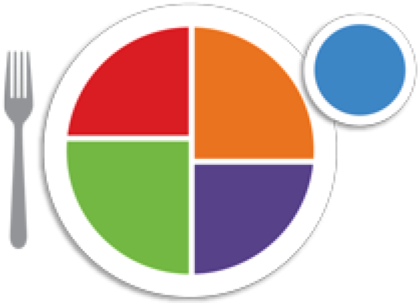

Visit MyPlate.gov

Peppers Stuffed with Rice and Beans

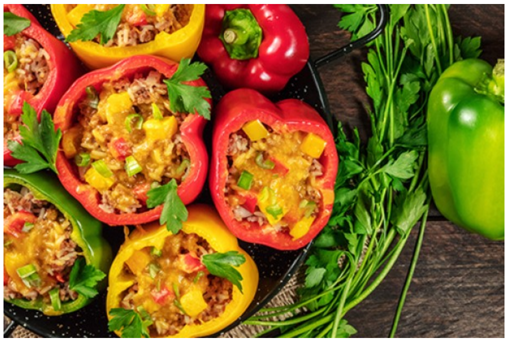

Makes: 4 Servings

These simple stuffed peppers are fun and flavorful. Use yellow, red, green, and orange peppers for a colorful dish.

Ingredients

- 1 cup brown rice, uncooked
- 1 can black beans, low sodium (15 ounces)
- 4 bell peppers (any color)
- 1 cup cheddar cheese, shredded (reduced fat)
- 1 tomato, sliced
- 1 cup salsa
- salt (to taste, optional)

Directions

1. Wash hands with soap and water.
2. Preheat the oven to 400 °F.
3. Cook brown rice according to package directions.
4. Wash the peppers under running water.
5. Cut the tops off the peppers and spoon out the seeds.
6. Drain and rinse the black beans.
7. Combine the beans, rice, salsa, and salt (optional).
8. Spoon about 3 tablespoons of the mixture into the bottom of each pepper.
9. Place a slice of tomato on top of the mixture and sprinkle with 2 tablespoons of cheese.
10. Repeat steps 8 and 9 to fill the pepper completely, but do not top with cheese.
11. Bake peppers for 30 minutes.
12. Top each pepper with 2 tablespoons of cheese and continue baking for 15 minutes more.

Source:

Adapted from Simple Stuffed Peppers, Simple Healthy Recipes
Oklahoma Nutrition Information and Education
ONIE Project

Nutrition Information

Serving Size: 1/4 of recipe

Nutrients	Amount
**Total Calories**	**388**
**Total Fat**	**7 g**
Saturated Fat	4 g
Cholesterol	16 mg
**Sodium**	**631 mg**
**Carbohydrates**	**63 g**
Dietary Fiber	13 g
Total Sugars	6 g
Added Sugars included	0 g
**Protein**	**20 g**
Vitamin D	0 mcg
Calcium	330 mg
Iron	3 mg
Potassium	795 mg

Nutrients will display if the data is available

Please note: nutrient values are subject to change as data is updated

MyPlate Food Groups

Vegetables	2 cups
Grains	1 1/2 ounces
Protein Foods	2 1/2 ounces
Dairy	1 cup

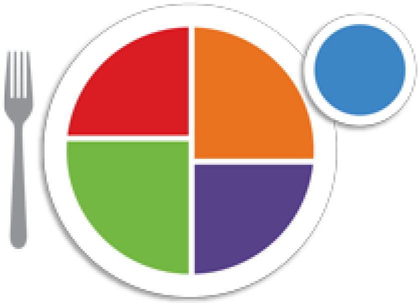

Visit MyPlate.gov

Julia's Sautéed & Steamed Collards

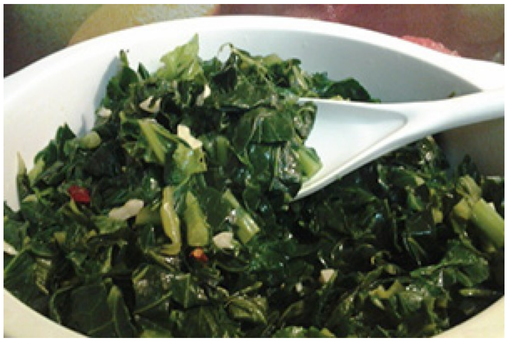

Makes: 8 Servings

Collards are an all-time favorite side to any meal. This recipe starts with tender collards and adds a hint of "heat" from hot pepper flakes. Try this easy and tasty recipe at your next family gathering.
- Julia, CNPP

Ingredients

- 16 cups chopped collards (small leaves)
- 2 tablespoons olive oil
- 4 large garlic cloves
- 1/2 teaspoon hot pepper flakes
- 1 teaspoon salt-free seasoning

Directions

1. Cut bottom stems from collards.
2. Wash and cut collards into bite-sized pieces. Place in colander to drain.
3. Chop garlic cloves into small pieces.
4. Place olive oil into large skillet. Place on high heat to start.
5. Place collards in skillet. Keep on high heat, turning collards as they saute, about 1 minute in the uncovered skillet. (You will not need to add water because of the water already on the washed collards.)
6. Add garlic cloves and red pepper flakes.
7. Place cover on the skillet to allow collards to steam until they are half their original volume.
8. Turn heat to low and continue steaming until the collards are tender.

Source:

USDA Center for Nutrition Policy and Promotion
Recipe submitted by Julia

Nutrition Information

Serving Size: 1 portion of Julia's Sauteed & Steamed Collards

Nutrients	Amount
**Total Calories**	**54**
**Total Fat**	**4 g**
Saturated Fat	1 g
Cholesterol	0 mg
**Sodium**	**15 mg**
**Carbohydrates**	**5 g**
Dietary Fiber	3 g
Total Sugars	0 g
Added Sugars included	0 g
**Protein**	**2 g**
Vitamin D	0 mcg
Calcium	111 mg
Iron	0 mg
Potassium	135 mg

Nutrients will display if the data is available

Please note: nutrient values are subject to change as data is updated

MyPlate Food Groups

Vegetables 1 cup

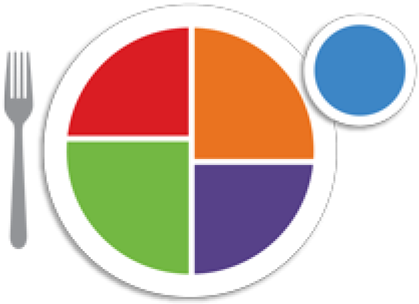

Visit MyPlate.gov

Bell Pepper and Apple Coleslaw

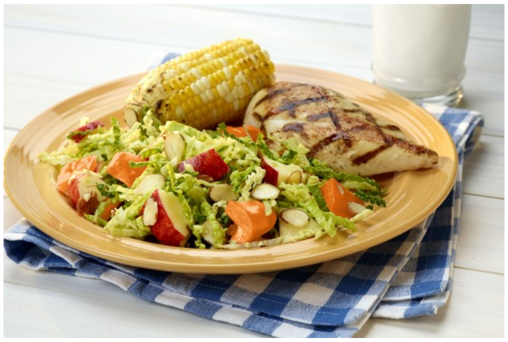

Makes: 4 Servings
Preparation Time: 20 minutes

Apples and bell peppers liven up this coleslaw made with an easy vinegar mustard dressing. Add more color with red, orange or yellow bell peppers. Use your favorite sweet and crisp apple, such as Gala or Fuji.

Ingredients

For the Dressing:

- 1 tablespoon vegetable oil
- 1/4 cup apple juice
- 2 tablespoons cider vinegar
- 2 teaspoons Dijon mustard
- 1/4 teaspoon salt
- 1 dash black pepper

For the Salad:

- 1 small head green or Napa cabbage
- 1 orange or red bell pepper
- 1 Gala or Fuji apple
- 1/4 cup sliced almonds

Directions

1. Whisk together dressing ingredients.
2. Shred or thinly slice cabbage.
3. Cut apple and pepper into small chunks.
4. Place cabbage, apples, pepper and almonds in large bowl.
5. Drizzle with dressing and toss.

Source:

Produce for Better Health Foundation

Nutrition Information

Nutrients	Amount
**Total Calories**	**154**
**Total Fat**	**9 g**
Saturated Fat	1 g
Cholesterol	0 mg
**Sodium**	**229 mg**
**Carbohydrates**	**17 g**
Dietary Fiber	5 g
Total Sugars	9 g
Added Sugars included	0 g
**Protein**	**4 g**
Vitamin D	0 mcg
Calcium	83 mg
Iron	1 mg
Potassium	386 mg

Nutrients will display if the data is available

Please note: nutrient values are subject to change as data is updated

MyPlate Food Groups

Fruits	1/2 cups
Vegetables	1 1/2 cups
Protein Foods	1/2 ounces

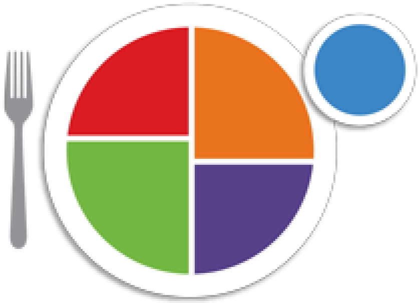

Visit MyPlate.gov

Slow Cooker Lentil Soup

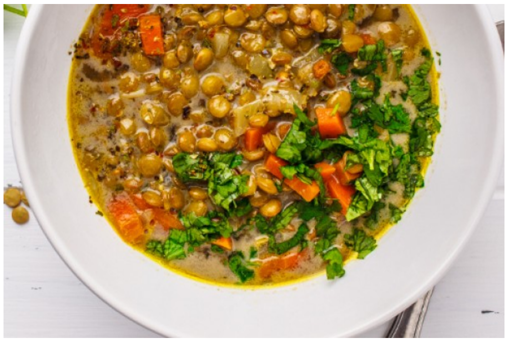

Makes: 6 servings

Quick to make but rich and delicious flavors of carrots, onion, and celery flavor in this slow cooker lentil soup. Add your favorite spices if you want to add a little kick.

Ingredients

- 6 cups water
- 1/4 cup fresh parsley, chopped (or 2 tablespoons dried)
- 2 teaspoons beef bouillon (or 2 cubes beef bouillon)
- 1 1/2 cups dry lentils
- 2 medium carrots, sliced
- 1 medium onion, chopped
- 2 medium celery stalks, sliced

Directions

1. Wash hands with soap and water.
2. Mix all ingredients together in slow cooker.
3. Cook on low for 8 to 10 hours or high for 4 to 5 hours.
4. Serve hot with crackers or bread.

Source:

Montana State University Extension Service
Nutrition Education Program

Nutrition Information

Serving Size: 1/6 of recipe

Nutrients	Amount
**Total Calories**	**177**
**Total Fat**	**1 g**
Saturated Fat	0 g
Cholesterol	0 mg
**Sodium**	**186 mg**
**Carbohydrates**	**32 g**
Dietary Fiber	12 g
Total Sugars	5 g
Added Sugars included	0 g
**Protein**	**13 g**
Vitamin D	0 mcg
Calcium	53 mg
Iron	5 mg
Potassium	642 mg

Nutrients will display if the data is available

Please note: nutrient values are subject to change as data is updated

MyPlate Food Groups

Vegetables	1 cups
Protein Foods	3 ounces

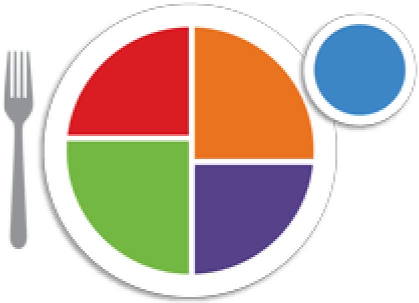

Visit MyPlate.gov

Trail Mix Bars

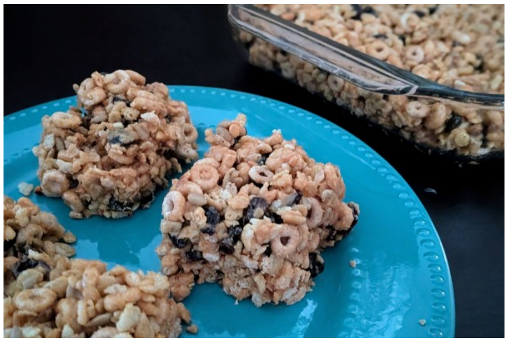

Makes: 28 Bars

You can adjust this recipe to accommodate food allergies. Try with sunflower seed butter or almond butter if you are working with a peanut allergy.

Ingredients

- 3 cups crispy rice cereal
- 3 cups toasted oat cereal
- 1 1/2 cups raisins
- 1/2 cup sunflower seeds, no salt
- 1 cup honey*
- 3/4 cup sugar
- 1 (16 ounce) jar chunky peanut butter
- 1 teaspoon vanilla

Directions

1. Wash hands with soap and water.
2. Combine dry ingredients in bowl.
3. Combine honey and sugar in pan and bring to a boil.
4. Add peanut butter and vanilla, stir until peanut butter melts.
5. Pour mixture over cereal and mix well.
6. Press into a 13x9 inch pan and cool.

Source:

University of Wisconsin Cooperative Extension Service

Nutrition Information

Serving Size: 1 bar, 1/28 of the recipe (50g)

Nutrients	Amount
**Total Calories**	**208**
**Total Fat**	**10 g**
Saturated Fat	2 g
Cholesterol	0 mg
**Sodium**	**83 mg**
**Carbohydrates**	**28 g**
Dietary Fiber	2 g
Total Sugars	22 g
Added Sugars included	15 g
**Protein**	**5 g**
Vitamin D	0 mcg
Calcium	23 mg
Iron	4 mg
Potassium	207 mg

Nutrients will display if the data is available

Please note: nutrient values are subject to change as data is updated

MyPlate Food Groups

Protein Foods 1 ounces

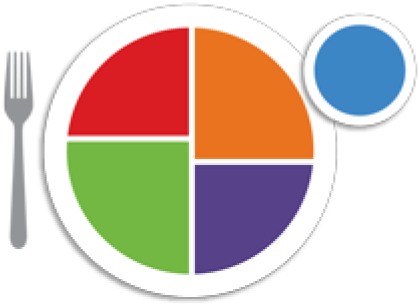

Visit MyPlate.gov
