# Supplementary material for: The Diet Guidelines: 3 Diets (DG3D) study protocol of a behavioral teaching kitchen intervention for type-2 diabetes prevention among African American adults
Source: Contemp Clin Trials. Author manuscript; Available in PMC 2026 Jun 18. (PMC13276729; doi:10.1016/j.cct.2025.108109)
Supplement: 2 [file NIHMS2184820-supplement-2.pdf]

USDA
United States Department of Agriculture

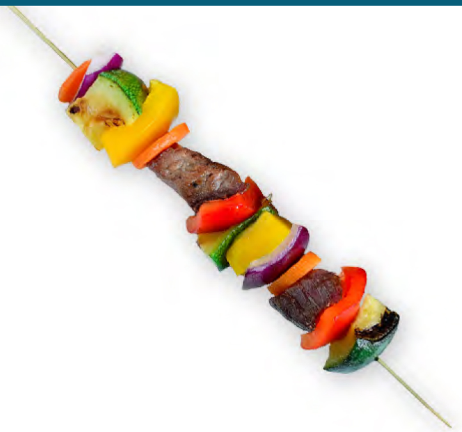
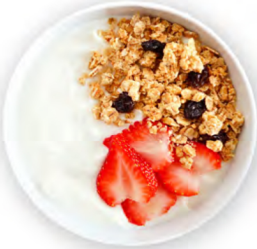
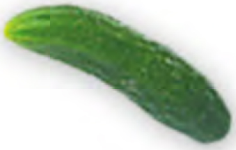
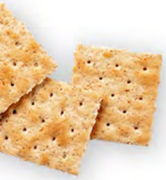

Start simple
with MyPlate

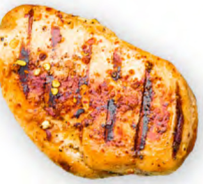
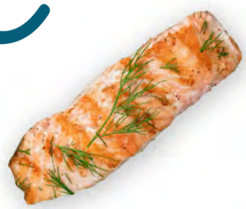

Nutrient-Rich

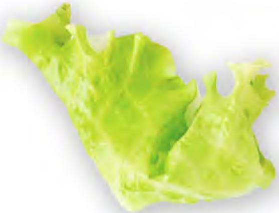
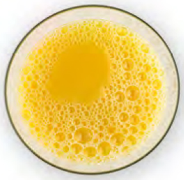
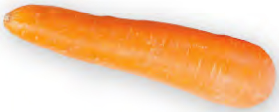
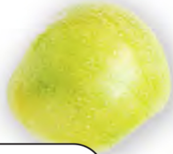

Table of Contents

Veggie Omelet in a Mug 4
Avocado and Corn Salsa 6
Very Berry Smoothie 8
Candied Yams 10
Easiest Banana Ice Cream 12
Overnight Oatmeal 14
Lentil Stew 16
Apple Cinnamon Bars 18
Simple Green Smoothie 20
Yogurt Berry Parfait 22
Couscous with Peas and Onions 24
Minestrone Soup 26
White Bean Bruschetta 28
Banana Pudding 30
Deviled Eggs 32
Oven-Baked Potato Pancakes 34
Tomato and Cucumber Salad 36
Gingery Quinoa with Green Beans 38
Hummus 40
Fish with Spinach 42
Apple Cranberry Salad Toss 44
Classic Macaroni and Cheese 46
Curried Chicken Salad 48
Basic Quiche 50
Grilled Asparagus and Shrimp Quinoa Salad with Lemon Vinaigrette 52
Tuna Salad on Greens 55
20-Minute Chicken Creole 57
Zingy Lemon Chicken Pasta 60
Salmon Patties 63
Black Bean Quesadillas 66
Garden Chili 68
Grilled Vegetables 70
Angel Food Pastry with Fresh Berries and Whipped Cream 72
Tropical Yogurt Pops 74
Chocolate Chip Yogurt Cookies 76
Apple-Stuffed Squash 78
Rainbow Veggie Salad 80
Grilled Fruit 82
Grilled Vegetable Packets 84
Skinny Pizza 86
Rice-Crusted Pizza 88

Curried Chickpea Salad 91
Roasted Chickpeas (Garbanzo Beans) 93
Heavenly Deviled Eggs 95
Broccoli Potato Soup 97
Marinated Beef 99
Overnight Oatmeal with Berries 101
Quinoa and Black Bean Salad 103
Stuffed Bell Peppers 106
Julia’s Sautéed & Steamed Collards 108
Healthy Black Bean Soup 110
Sweet Potato Casserole 112
Bell Pepper and Apple Coleslaw 114
Apple & Chicken Salad 116
Slow Cooker Lentil Soup 118
Cinnamon-Raisin Breakfast Mix 120

Veggie Omelet in a Mug

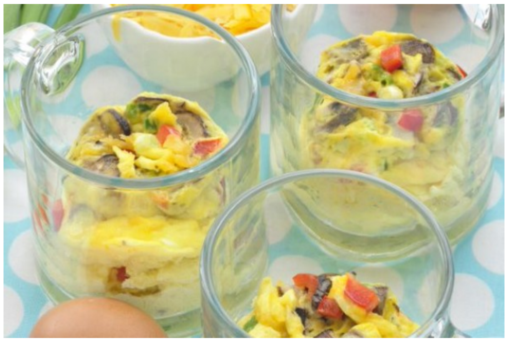

Makes: 1 Serving
Preparation Time: 10 minutes
Cook Time: 3 minutes

Making a veggie omelet has never been so easy! Combine all of your ingredients in a mug, pop it in the microwave, and breakfast is ready!

Ingredients

- 2 eggs
- 2 tablespoons fat-free (skim) milk
- 1 pinch salt
- 1 pinch black pepper
- 1/4 cup finely chopped mushrooms (or your favorite vegetables)
- 2 tablespoons low-fat cheddar cheese, shredded (or your favorite cheese)

Directions

1. Wash hands with soap and water.
2. Lightly grease the inside of a 12-ounce microwave-safe mug.
3. Use a fork to combine the eggs, milk, salt and pepper in the mug and stir well. Mix in the vegetables and cheese.
4. Microwave on HIGH for 45 seconds. Stir. Return to the microwave and cook on HIGH until the mixture has puffed and set, 60 to 90 seconds. The omelet may look wet on the top but it will dry as it cools.
5. Refrigerate leftovers within 2 hours.

Source:

Food Hero
Oregon State University Cooperative Extension Service

Nutrition Information

Serving Size: 1 recipe

Nutrients	Amount
**Total Calories**	**180**
**Total Fat**	**11 g**
Saturated Fat	4 g
Cholesterol	336 mg
**Sodium**	**397 mg**
**Carbohydrates**	**3 g**
Dietary Fiber	0 g
Total Sugars	2 g
Added Sugars included	0 g
**Protein**	**17 g**
Vitamin D	2 mcg
Calcium	216 mg
Iron	2 mg
Potassium	239 mg

Nutrients will display if the data is available

Please note: nutrient values are subject to change as data is updated

MyPlate Food Groups

Vegetables	1/4 cups
Protein Foods	2 ounces
Dairy	1/2 cups

Visit MyPlate.gov

Avocado and Corn Salsa

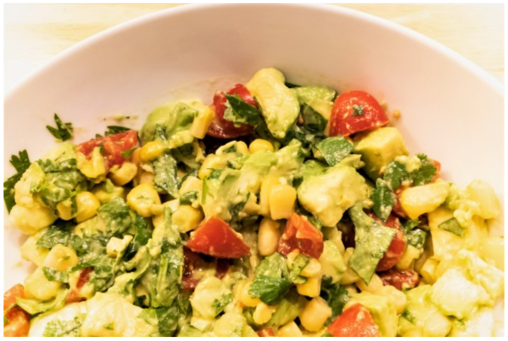

Makes: 5 Servings

Avocado and corn are given a flavor boost by fresh cilantro and lime. Serve this salsa with our Baked Chicken for an easy weekday meal.

Ingredients

- 1 avocado, diced
- 3/4 cup frozen corn kernels, thawed
- 1/2 cup grape tomatoes, quartered
- 1 tablespoon fresh cilantro, chopped
- 2 teaspoons lime juice
- 1/4 teaspoon salt

Directions

1. Wash hands with soap and water.
2. Toss avocado, corn, tomatoes, cilantro, lime juice, and salt in a medium bowl.
3. Chill one hour and then serve.

Source:

Bronson Wellness Center
Bronson Healthcare - Michigan

Nutrition Information

Serving Size: 1/2 cup

Nutrients	Amount
**Total Calories**	**67**
**Total Fat**	**4 g**
Saturated Fat	1 g
Cholesterol	0 mg
**Sodium**	**119 mg**
**Carbohydrates**	**8 g**
Dietary Fiber	3 g
Total Sugars	1 g
Added Sugars included	0 g
**Protein**	**1 g**
Vitamin D	0 mcg
Calcium	6 mg
Iron	0 mg
Potassium	235 mg

Nutrients will display if the data is available

Please note: nutrient values are subject to change as data is updated

MyPlate Food Groups

Vegetables 1/2 cups

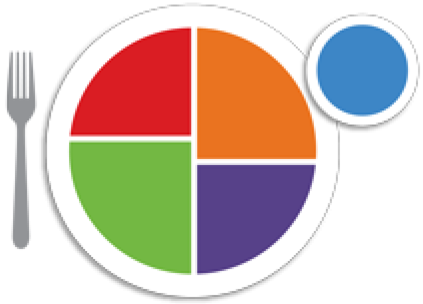

Visit MyPlate.gov

Very Berry Smoothie

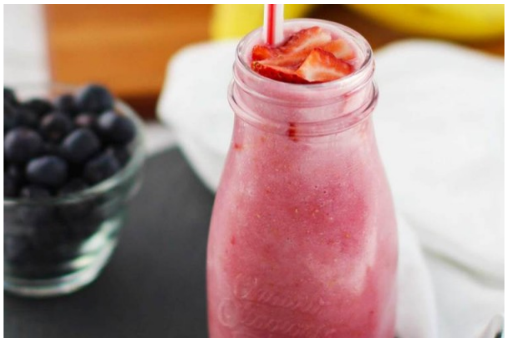

Makes: 2 Servings

This refreshing pineapple, banana, and berry blend smoothie has 2 and 1/2 cups of fruit per serving.

Ingredients

- 1 cup frozen strawberries
- 1 cup frozen raspberries
- 1 cup pineapple chunks
- 1 banana
- 1 cup skim milk (or almond milk)
- 1 1/2 cups ice

Directions

1. Peel and slice the banana.
2. Gather all ingredients and combine in a blender.
3. Cover and blend until smooth.
4. Serve right away or pour into ice cube trays and freeze. During the week, pop out the cubes for a quick on-the-go breakfast.

Source:

USDA Center for Nutrition Policy and Promotion

Nutrition Information

Nutrients	Amount
**Total Calories**	**252**
**Total Fat**	**1 g**
Saturated Fat	0 g
Cholesterol	2 mg
**Sodium**	**56 mg**
**Carbohydrates**	**59 g**
Dietary Fiber	13 g
Total Sugars	37 g
Added Sugars included	0 g
**Protein**	**7 g**
Vitamin D	1 mcg
Calcium	N/A
Iron	N/A
Potassium	N/A

Nutrients will display if the data is available

Please note: nutrient values are subject to change as data is updated

MyPlate Food Groups

Fruits 2 1/2 cups
Dairy 1/2 cups

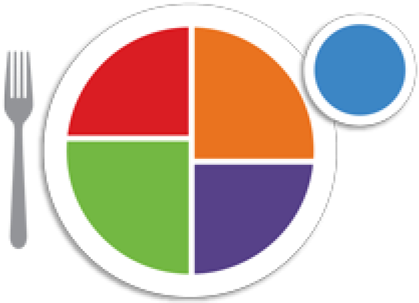

Visit MyPlate.gov

Candied Yams

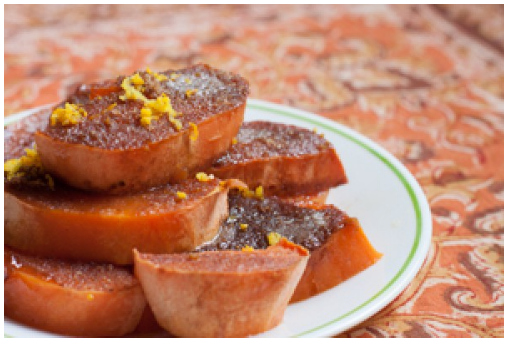

Makes: 6 servings

Enjoy this side dish for a Thanksgiving treat or anytime yams are in season.

Ingredients

- 1 1/2 cups yams
- 1/4 cup brown sugar, packed
- 1 teaspoon flour, sifted
- 1/4 teaspoon salt
- 1/4 teaspoon ground cinnamon
- 1/4 teaspoon ground nutmeg
- 1/4 teaspoon orange peel
- 1 teaspoon unsalted margarine
- 1/2 cup orange juice

Directions

1. Wash hands with soap and water.
2. Preheat oven to 350 °F.
3. Cut yams in half and boil until tender, but firm (about 20 minutes). When cool enough to handle, peel and slice into 1/4 inch thickness.
4. Combine sugar, flour, salt, cinnamon, nutmeg, and grated orange peel.
5. Place half of sliced yams in medium-sized casserole dish. Sprinkle with spiced sugar mixture.
6. Dot with half the amount of margarine.
7. Add second layer of yams, using the rest of the ingredients in the order above. Add orange juice.
8. Bake uncovered for 20 minutes.

Source:

Heart Healthy Home Cooking: African American Style
US Department of Health and Human Services
National Heart, Lung, and Blood Institute

Nutrition Information

Serving Size: 1/4 Cup (66g)

Nutrients	Amount
**Total Calories**	**81**
**Total Fat**	**1 g**
Saturated Fat	0 g
Cholesterol	0 mg
**Sodium**	**118 mg**
**Carbohydrates**	**18 g**
Dietary Fiber	1 g
Total Sugars	12 g
Added Sugars included	8 g
**Protein**	**1 g**
Vitamin D	0 mcg
Calcium	21 mg
Iron	0 mg
Potassium	167 mg

Nutrients will display if the data is available

Please note: nutrient values are subject to change as data is updated

MyPlate Food Groups

Vegetables 1/4 cups

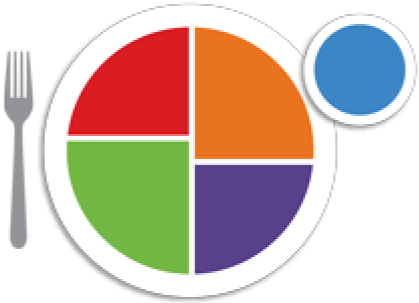

Visit MyPlate.gov

Easiest Banana Ice Cream

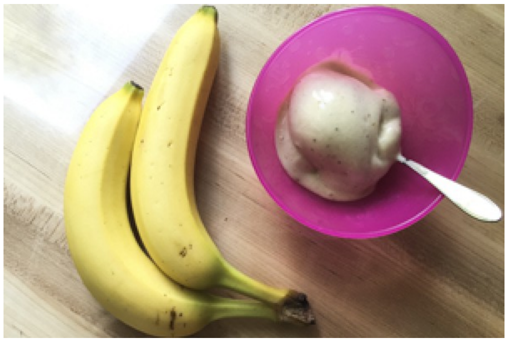

Makes: 3 Servings

What should you do with over-ripe bananas? Don't toss them! Freeze them and then throw them in the blender to make this delicious and creamy frozen dessert.

Ingredients

- 2 1/2 bananas (2-3 bananas)
- 3 tablespoons 1% milk
- chocolate syrup (optional)

Directions

1. Wash hands with soap and water.
2. When bananas have become very ripe, peel and slice them into medium size round pieces. Place on a plastic wrap covered baking sheet and put in freezer overnight.
3. Place frozen bananas in a food processor or blender with a small splash of milk (not over 1/4 cup total).
4. Pulse food processor or blender until bananas begin to break up. They will be tiny balls of bananas at this point.
5. Using a spoon or rubber spatula, scrape down the banana mixture. Continue running the food processor until the mixture is smooth and creamy. This may take a few minutes. The ice cream will look and tasted like soft serve ice cream when finished.
6. Serve in an ice cream cone or in a small bowl with a small ribbon of chocolate syrup on top (optional).

Source:

Alabama Cooperative Extension System

Nutrition Information

Serving Size: 1/3 of recipe

Nutrients	Amount
**Total Calories**	**94**
**Total Fat**	**0 g**
Saturated Fat	0 g
Cholesterol	1 mg
**Sodium**	**8 mg**
**Carbohydrates**	**23 g**
Dietary Fiber	3 g
Total Sugars	13 g
Added Sugars included	0 g
**Protein**	**2 g**
Vitamin D	0 mcg
Calcium	24 mg
Iron	0 mg
Potassium	375 mg

Nutrients will display if the data is available

Please note: nutrient values are subject to change as data is updated

MyPlate Food Groups

Fruits 3/4 cups

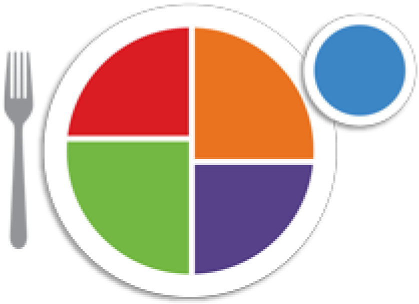

Visit MyPlate.gov

Overnight Oatmeal

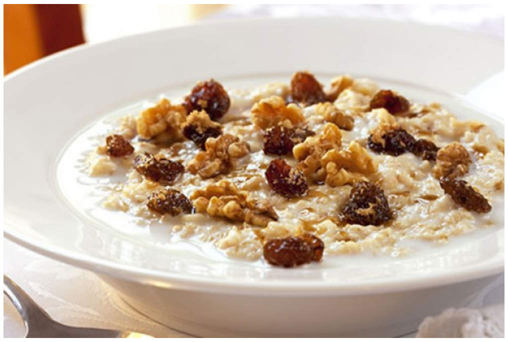

Makes: 2 Servings

Overnight soaking cuts down on prep time in the morning, so you can enjoy a healthy and hearty, cooked breakfast, even after hitting the snooze button.

Ingredients

- 1 cup dry oats
- 2 1/2 cups water
- 1/2 cup dried fruit (raisins, cranberries, or apricots)
- 1/4 cup walnuts, lightly chopped (or pecans)

Directions

1. Wash hands with soap and water.
2. Put all ingredients in a cooking pot and cover. Let sit overnight in refrigerator.
3. In the morning, put on a stove burner and cook on medium heat until simmering, then turn on low and heat for 10 to 15 minutes more or less depending on type of oats -- quick (5 minutes), regular, or steel cut.
4. Serve warm with a little milk (or milk alternative), a drizzle of maple syrup, and a side of fresh fruit, if desired.

Source:

Lake Family Resource Center
Be-Fresh Program

Nutrition Information

Serving Size: 1/2 of recipe

Nutrients	Amount
**Total Calories**	**333**
**Total Fat**	**13 g**
Saturated Fat	2 g
Cholesterol	0 mg
**Sodium**	**27 mg**
**Carbohydrates**	**50 g**
Dietary Fiber	7 g
Total Sugars	17 g
Added Sugars included	0 g
**Protein**	**8 g**
Vitamin D	0 mcg
Calcium	57 mg
Iron	3 mg
Potassium	470 mg

Nutrients will display if the data is available

Please note: nutrient values are subject to change as data is updated

MyPlate Food Groups

Fruits	1/2 cups
Grains	2 ounces
Protein Foods	1 ounces

Visit MyPlate.gov

Lentil Stew

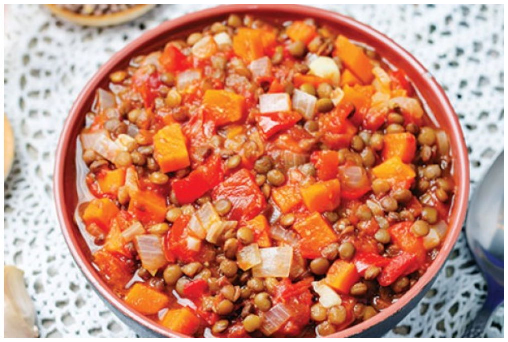

Makes: 10 servings

Lentils, carrots, and tomatoes are a savory and satisfying combination in this delicious and easy to prepare stew. Enjoy with a side of your favorite citrus fruit.

Ingredients

- 2 teaspoons olive oil (or canola oil)
- 1 onion (large, chopped)
- 1 teaspoon garlic powder
- 1 1/2 10-ounce packages of frozen sliced carrots
- 1 cup dry lentils (rinsed and drained)
- 3 cans diced tomatoes, low-sodium (14.5 ounces each)
- 3 cups water
- 1 teaspoon chili powder

Directions

1. Wash hands with soap and water.
2. Heat the oil in a large pot over medium heat.
3. Add chopped onion.
4. Cook for 3 minutes, or until tender.
5. Stir in garlic powder, carrots, lentils, tomatoes, water, and chili powder.
6. Simmer, uncovered, for about 20 minutes or until lentils are tender.

Source:

2009 Recipe Calendar
University of Maryland Extension
Food Supplement Nutrition Education Program

Nutrition Information

Serving Size: 1 cup

Nutrients	Amount
**Total Calories**	**113**
**Total Fat**	**2 g**
Saturated Fat	0 g
Cholesterol	0 mg
**Sodium**	**52 mg**
**Carbohydrates**	**20 g**
Dietary Fiber	7 g
Total Sugars	6 g
Added Sugars included	0 g
**Protein**	**6 g**
Vitamin D	0 mcg
Calcium	69 mg
Iron	3 mg
Potassium	536 mg

Nutrients will display if the data is available

Please note: nutrient values are subject to change as data is updated

MyPlate Food Groups

Vegetables	1 1/4 cups
Protein Foods	1 ounces

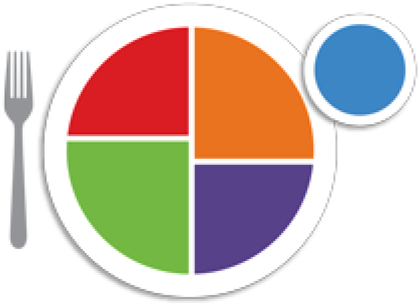

Visit MyPlate.gov

Apple Cinnamon Bars

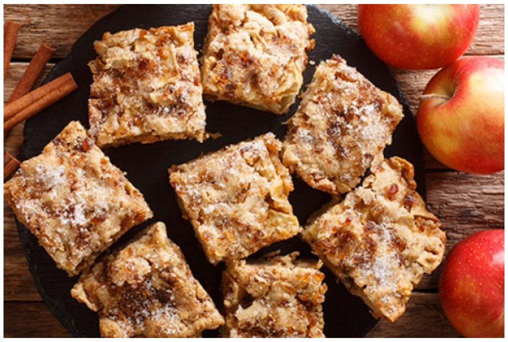

Makes: 24 servings

Apples are sandwiched between two cinnamon crumb layers for a delicious treat.

Ingredients

- 4 medium apples
- 1 cup flour
- 1/4 teaspoon salt
- 1/2 teaspoon baking soda
- 1/2 teaspoon cinnamon
- 1/2 cup brown sugar, packed
- 1 cup oats, uncooked
- 1/2 cup margarine, 1 stick (or butter)
- 4 sprays of cooking spray

Directions

1. Wash hands with soap and water.
2. Preheat the oven to 350 degrees F.
3. Put the flour, salt, baking soda, cinnamon, brown sugar, and oats in the mixing bowl. Stir together.
4. Add the margarine to the bowl. Use the 2 table knives to mix the ingredients and cut them into crumbs.
5. Lightly grease the bottom and sides of the baking dish with cooking spray.
6. Spread half of the crumb mixture in the greased baking dish.
7. Remove the core from the apples and slice them. Put the apple slices into the baking dish.
8. Top the apples with the rest of the crumb mixture.
9. Bake in the oven for 40-45 minutes.
10. Cut into squares. It will fall apart easily.

Source:

Pennsylvania Nutrition Education Network (original)
Recipe modification adapted from SNAP-Ed New York, October 2023

Nutrition Information

Serving Size: 1 bar, 1/24 of recipe

Nutrients	Amount
**Total Calories**	**82**
**Total Fat**	**4 g**
Saturated Fat	1 g
Cholesterol	0 mg
**Sodium**	**53 mg**
**Carbohydrates**	**12 g**
Dietary Fiber	1 g
Total Sugars	7 g
Added Sugars included	4 g
**Protein**	**1 g**
Vitamin D	0 mcg
Calcium	7 mg
Iron	0 mg
Potassium	37 mg

Nutrients will display if the data is available

Please note: nutrient values are subject to change as data is updated

MyPlate Food Groups

Fruits 1/4 cups

Grains 1/2 ounces

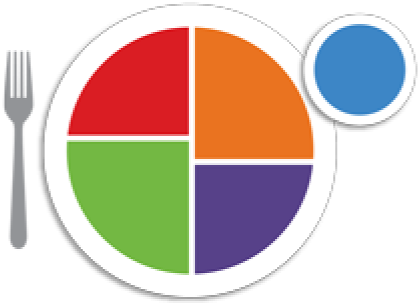

Visit MyPlate.gov

Simple Green Smoothie

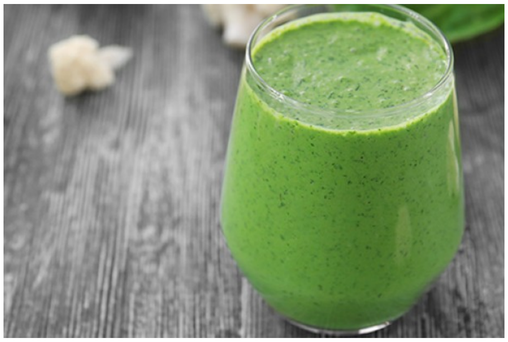

Makes: 2 Servings

Add kale or spinach to this fruit smoothie for a quick and delicious breakfast or snack.

Ingredients

- 1 cup kale or spinach
- 1 medium banana
- 1 cup low-fat milk (or fortified soy, coconut, or almond milk)
- 1 cup plain low-fat yogurt
- 1 medium apple (cored and sliced)
- 1 cup frozen fruit (all one fruit or a combination of mixed frozen fruit)
- flax seeds, 1 tablespoon (optional)
- chia seeds, 1 tablespoon (optional)

Directions

1. Wash hands with soap and water.
2. Add the kale or spinach and the liquid of your choice to a blender. Blend.
3. Add in the rest of the ingredients, blending after each item.
4. Serve and enjoy, cold.
5. Reserve the leftover smoothie in the refrigerator for later in the day or the next day.

Source:

Northern Valley Catholic Social Service

Nutrition Information

Nutrients	Amount
**Total Calories**	**299**
**Total Fat**	**4 g**
Saturated Fat	2 g
Cholesterol	N/A
**Sodium**	**156 mg**
**Carbohydrates**	**56 g**
Dietary Fiber	7 g
Total Sugars	38 g
Added Sugars included	0 g
**Protein**	**13 g**
Vitamin D	1 mcg
Calcium	454 mg
Iron	2 mg
Potassium	1119 mg

Nutrients will display if the data is available

Please note: nutrient values are subject to change as data is updated

MyPlate Food Groups

Fruits	2 cups
Vegetables	1/4 cups
Dairy	1 cup

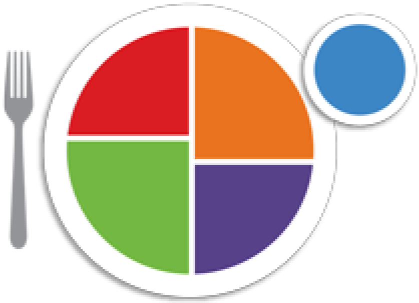

Visit MyPlate.gov

Yogurt Berry Parfait

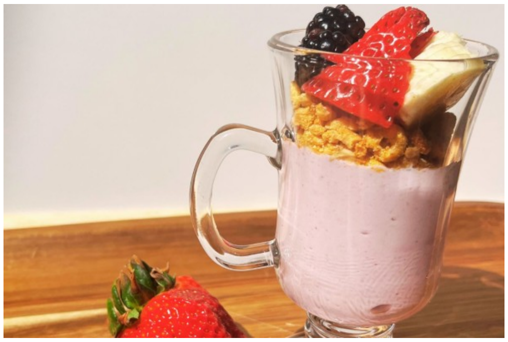

Makes: 4 servings
Preparation Time: 5 minutes

Enjoy this hearty snack or dessert, abounding with fresh fruit, granola, and any flavor of low-fat yogurt.

Ingredients

- 2 cups low fat yogurt (or fat-free, any flavor*)
- 1 cup banana (sliced)
- 1/2 cup blueberries (fresh)
- 1/2 cup strawberries (fresh, sliced)
- other optional fruit (raspberries, peaches, pineapple and/or mangos)
- 1 cup granola

Directions

1. Wash hands with soap and water.
2. Line up 4 parfait or other tall glasses.
3. Spoon 1/2 cup of yogurt into each glass.
4. Sprinkle with granola.
5. Top with about 1/4 cup of fruit.

Source:

USDA Food and Nutrition Information Center

Nutrition Information

Serving Size: 1 parfait, 1/4 of recipe (214g)

Nutrients	Amount
**Total Calories**	**304**
**Total Fat**	**9 g**
Saturated Fat	2 g
Cholesterol	6 mg
**Sodium**	**89 mg**
**Carbohydrates**	**46 g**
Dietary Fiber	5 g
Total Sugars	30 g
Added Sugars included	11 g
**Protein**	**11 g**
Vitamin D	1 mcg
Calcium	240 mg
Iron	2 mg
Potassium	613 mg

Nutrients will display if the data is available

Please note: nutrient values are subject to change as data is updated

MyPlate Food Groups

Fruits	1/2 cups
Grains	1/2 ounces
Protein Foods	1/2 ounces
Dairy	1/2 cups

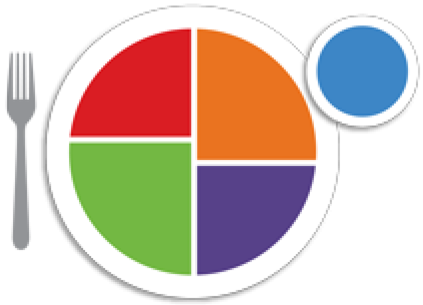

Visit MyPlate.gov

Couscous with Peas and Onions

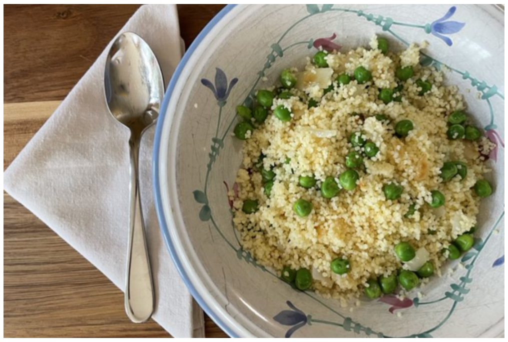

Makes: 4 servings

Couscous is cooked with sage, peas, and onions for a savory side dish. Look for couscous or whole grain couscous in the aisle near the rice.

Ingredients

- 1 teaspoon olive oil
- 1 cup onion, finely chopped
- 1 cup frozen green peas
- 1/2 teaspoon ground sage
- 1 1/3 cups water
- 1 cup couscous
- salt (1/2 teaspoon, optional)

Directions

1. Wash hands with soap and water.
2. Combine oil and onions in heavy skillet.
3. Sauté for 5 to 10 minutes until lightly browned.
4. Add the peas, sage, water, couscous, and salt if desired.
5. Cover and cook on medium heat for about 5 minutes or until peas are tender but still bright green and all of the water is absorbed.
6. Fluff with fork.

Source:

CHOICES: Steps Toward Health
UMass Extension Nutrition Education Program

Nutrition Information

Serving Size: 1 cup

Nutrients	Amount
**Total Calories**	**205**
**Total Fat**	**1 g**
Saturated Fat	0 g
Cholesterol	0 mg
**Sodium**	**40 mg**
**Carbohydrates**	**40 g**
Dietary Fiber	5 g
Total Sugars	4 g
Added Sugars included	0 g
**Protein**	**8 g**
Vitamin D	0 mcg
Calcium	33 mg
Iron	1 mg
Potassium	179 mg

Nutrients will display if the data is available

Please note: nutrient values are subject to change as data is updated

MyPlate Food Groups

Vegetables	1/2 cups
Grains	1 1/2 ounces

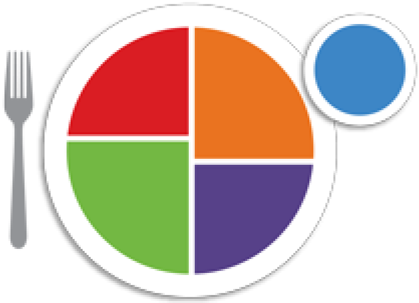

Visit MyPlate.gov

Minestrone Soup

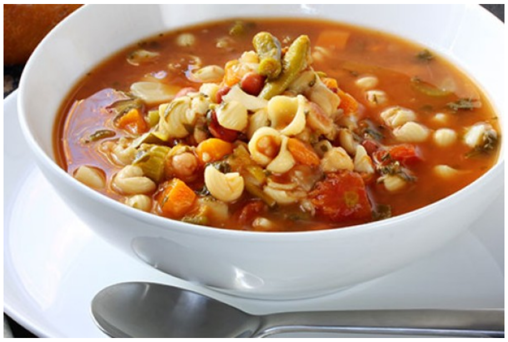

Makes: 6 servings

Prepare delicious soup in a snap! Canned beans and frozen veggies make this dish perfect for busy weeknights.

Ingredients

- 1 package (10 ounces) frozen vegetables, any type
- 2 cans (14.5 ounces each) low-sodium stewed tomatoes
- 2 cans (14.5 ounces each) low-sodium broth, any flavor
- 1 can (15.5 ounces) low-sodium beans, any type
- 1 cup dry pasta, any type

Directions

This recipe is developed for a child to help an adult in the kitchen. Directions are written to different audiences.

1. **Adult and child**: Wash hands with soap and water.
2. **Child**: In a large pot, combine frozen vegetables, tomatoes, broth and beans.
3. **Adult**: Bring the soup to a boil and add the pasta. Then reduce to low heat. Let simmer for 6 to 8 minutes or until the pasta and vegetables are tender.

Source:

Food Wise Learn at Home Print Materials
Rutgers Cooperative Extension

Nutrition Information

Serving Size: 1 cup, 1/6 of recipe

Nutrients	Amount
**Total Calories**	**144**
**Total Fat**	**1 g**
Saturated Fat	0 g
Cholesterol	0 mg
**Sodium**	**172 mg**
**Carbohydrates**	**29 g**
Dietary Fiber	9 g
Total Sugars	6 g
Added Sugars included	1 g
**Protein**	**7 g**
Vitamin D	N/A
Calcium	80 mg
Iron	3 mg
Potassium	163 mg

Nutrients will display if the data is available

Please note: nutrient values are subject to change as data is updated

MyPlate Food Groups

Vegetables	1 1/4 cups
Protein Foods	1 1/2 ounces

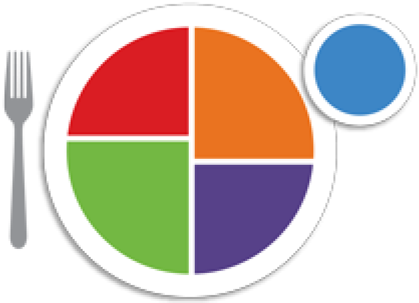

Visit MyPlate.gov

White Bean Bruschetta

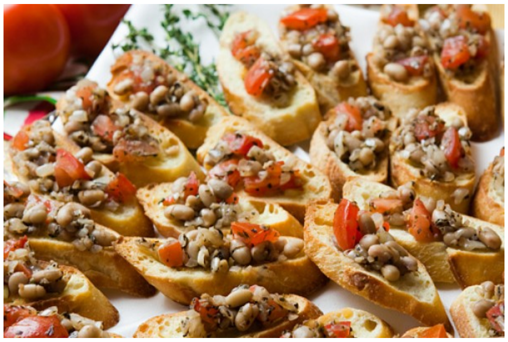

Makes: 4 Servings

Try this tasty bruschetta with crispy bread topped with white beans, tomatoes, and spices. Served as a side dish or an appetizer, it is sure to be a crowd pleaser!

Ingredients

- 1 whole-wheat French baguette, cut into 12 thin slices along the bias
- 1/4 cup olive oil, divided
- 1 cup white onion, chopped
- 4 cloves garlic, minced
- 1 teaspoon dried basil
- 1 teaspoon dried oregano
- 1 cup canned navy beans, rinsed and drained
- 2 tomatoes, cored and cubed
- 2 tablespoons balsamic vinegar

Directions

1. Slice the baguette on the diagonal into thin slices (about 12 slices for a baguette).
2. In a large sauté pan, heat 2 tablespoons of the olive oil over medium heat.
3. Place the bread slices in the pan and cook on medium high heat until sizzling and golden. Before flipping the bread, add an additional tablespoon of olive oil to the pan and cook the second side until golden.
4. For the topping, cook the onions and the remaining tablespoon of olive oil over medium heat until the onions are soft, about 7 minutes.
5. Add the garlic, basil, and oregano and cook another minute or two, until fragrant.
6. Add beans and continue cooking for another five minutes on low heat. Add the tomatoes and turn off the heat, allowing tomatoes to warm without cooking.
7. Drizzle the balsamic vinegar into the pan and gently stir.
8. Scoop heaping spoonsful of the tomato-bean mixture onto the grilled bread and enjoy.

Source:

MyPlate National Strategic Partners
The Grain Chain

Nutrition Information

Nutrients	Amount
**Total Calories**	**342**
**Total Fat**	**15 g**
Saturated Fat	2 g
Cholesterol	0 mg
**Sodium**	**332 mg**
**Carbohydrates**	**43 g**
Dietary Fiber	7 g
Total Sugars	6 g
Added Sugars included	1 g
**Protein**	**10 g**
Vitamin D	0 mcg
Calcium	95 mg
Iron	4 mg
Potassium	574 mg

Nutrients will display if the data is available

Please note: nutrient values are subject to change as data is updated

MyPlate Food Groups

Vegetables	3/4 cups
Grains	1 1/2 ounces

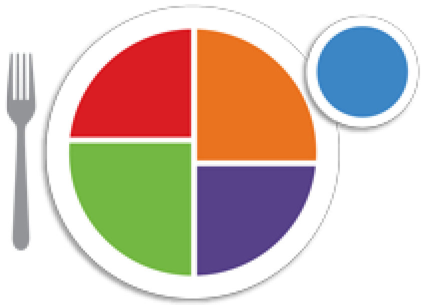

Visit MyPlate.gov

Banana Pudding

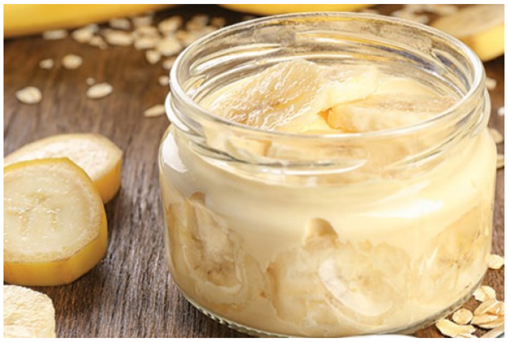

Makes: 8 servings

Dress up instant pudding mix with this dessert that combines low fat dairy and fruit for a tasty, nourishing treat.

Ingredients

- 1 (3.4 ounce) box instant pudding mix, banana or vanilla
- 2 cups fat-free (skim) milk (or 1% milk)
- 1 (8 ounce) container of fat-free plain yogurt (or non-dairy whipped topping)
- 2 medium bananas, sliced (or other sliced fruit)

Directions

1. Wash hands with soap and water.
2. In a medium bowl, combine pudding and milk. Beat with wooden spoon, wire whisk, or electric mixer on lowest speed for 2 minutes
3. Gently mix yogurt (or whipped topping) with pudding mixture. Refrigerate for 30 minutes.
4. Layer fruit slices in the bottom of 8 dessert cups.
5. Pour the pudding mixture over sliced fruit. Top with more fruit.
6. Refrigerate until ready to serve, at least 5 minutes. The taste is better if it's refrigerated longer.

Source:

Choices: Steps Toward Health, Adapted from: Lisa's Famous Dessert
University of Massachusetts
Extension Nutrition Education Program

Nutrition Information

Serving Size: 1/8 of recipe

Nutrients	Amount
**Total Calories**	**110**
**Total Fat**	**1 g**
Saturated Fat	0 g
Cholesterol	3 mg
**Sodium**	**219 mg**
**Carbohydrates**	**23 g**
Dietary Fiber	1 g
Total Sugars	20 g
Added Sugars included	11 g
**Protein**	**4 g**
Vitamin D	1 mcg
Calcium	130 mg
Iron	0 mg
Potassium	269 mg

Nutrients will display if the data is available

Please note: nutrient values are subject to change as data is updated

MyPlate Food Groups

Fruits 1/4 cups

Dairy 1/4 cups

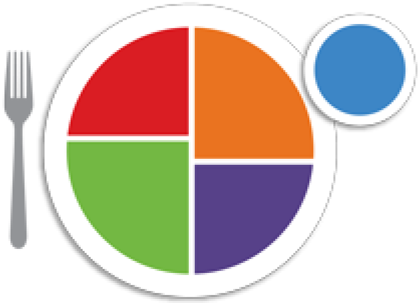

Visit MyPlate.gov

Deviled Eggs

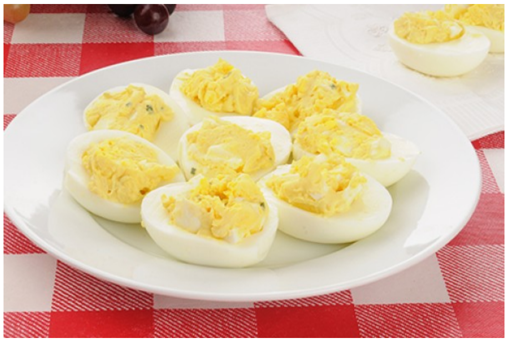

Makes: 6 servings

A favorite dish at parties and potlucks, Deviled Eggs also make a great, protein-filled anytime snack!

Ingredients

- 6 large eggs, hard-boiled and peeled
- 1/4 cup mayonnaise
- 1/8 teaspoon salt
- 1/8 teaspoon black pepper

Directions

1. Wash hands with soap and water.
2. Hard boil eggs by placing eggs in a saucepan and covering them with water. Bring to a boil.
3. Reduce heat to simmer; cook for 15 minutes.
4. Immediately rinse under cold water to stop cooking and to make it easy to peel off shells. Refrigerate peeled eggs (without shells) until ready for use.
5. Slice eggs into halves lengthwise. Remove yellow yolks and save whites.
6. Place yolks in a one-quart zip lock style bag along with the remaining ingredients (except the egg whites). Press out air.
7. Close bag and knead (mush together) until ingredients are well-blended. (Note: you could also put yolks in a bowl with other ingredients [except the egg whites] and mix together well until they look like a paste).
8. Push contents toward one corner of the bag. Cut about 1/2 inch off the corner of the bag. Squeezing the bag gently, fill reserved egg white hollows with the yolk mixture. (Note if you used a bowl, spoon the yolk mixture into the egg whites).
9. Chill to blend flavors.

Source:

Kids a Cookin'
Kansas Family Nutrition Program

Nutrition Information

Serving Size: 2 prepared egg halves, 1/6 of recipe

Nutrients	Amount
**Total Calories**	**134**
**Total Fat**	**12 g**
Saturated Fat	3 g
Cholesterol	190 mg
**Sodium**	**180 mg**
**Carbohydrates**	**0 g**
Dietary Fiber	0 g
Total Sugars	0 g
Added Sugars included	0 g
**Protein**	**6 g**
Vitamin D	1 mcg
Calcium	29 mg
Iron	1 mg
Potassium	72 mg

Nutrients will display if the data is available

Please note: nutrient values are subject to change as data is updated

MyPlate Food Groups

Protein Foods 1 ounces

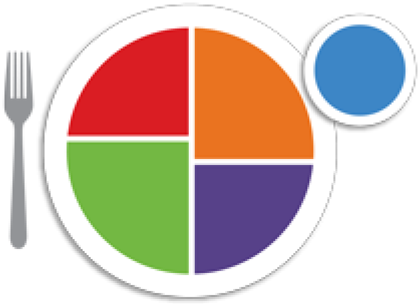

Visit MyPlate.gov

Oven-Baked Potato Pancakes

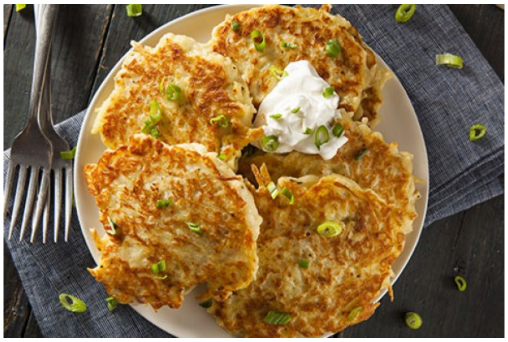

Makes: 12 Servings

These potato pancakes are easily baked in the oven and offer a healthier version of a traditional Hanukkah recipe.

Ingredients

- 2 tablespoons olive oil (or cooking oil of your choice)
- 3 tablespoons whole wheat flour (or all-purpose flour)
- 1 teaspoon baking powder
- 3/4 teaspoon salt
- 2 large russet potatoes (or Yukon Gold potatoes)
- 1 onion, small peeled
- 1 large egg
- applesauce and yogurt (plain low-fat or Greek) (optional)

Directions

1. Wash hands with soap and water.
2. Preheat the oven to 425 °F. Lightly oil the baking sheets by spreading the oil around with your clean hand or a paper towel.
3. Mix the flour, baking powder, and salt in a small bowl.
4. Use the large holes on your grater to grate the potato, then grate the onion too.
5. Put the potato and onion mixture in the colander inside the sink. Using a paper towel, press the potato mixture down to squeeze out and blot up some of the extra moisture. Stir it and blot again.
6. Put the potato mixture in the large bowl, add the egg, and stir well. Add the flour mixture and stir it very well.
7. Use a spoon or your fingers to pluck a clump of the potato mixture from the bowl and spread it into a round, flat nest on the oiled baking sheet: it should make a circle that's about 3 inches wide and 1/4 inch thick. Repeat to fill the sheet. (You don't need to leave space between them.)
8. Bake until the bottoms are deeply golden, 15 to 20 minutes, then turn the pancakes over and put them back in the over for 10 more minutes.
9. Serve the latkes with applesauce and yogurt (optional).

Source:

ChopChop Family

Nutrition Information

Serving Size: 1 latke, 1/12 of recipe (76g)

Nutrients	Amount
**Total Calories**	**83**
**Total Fat**	**3 g**
Saturated Fat	0 g
Cholesterol	16 mg
**Sodium**	**196 mg**
**Carbohydrates**	**13 g**
Dietary Fiber	2 g
Total Sugars	1 g
Added Sugars included	0 g
**Protein**	**2 g**
Vitamin D	0 mcg
Calcium	35 mg
Iron	1 mg
Potassium	285 mg

Nutrients will display if the data is available

Please note: nutrient values are subject to change as data is updated

MyPlate Food Groups

Vegetables 1/2 cups

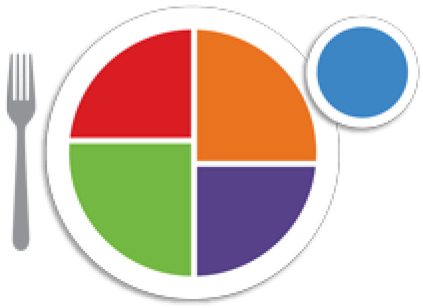

Visit MyPlate.gov

Tomato and Cucumber Salad

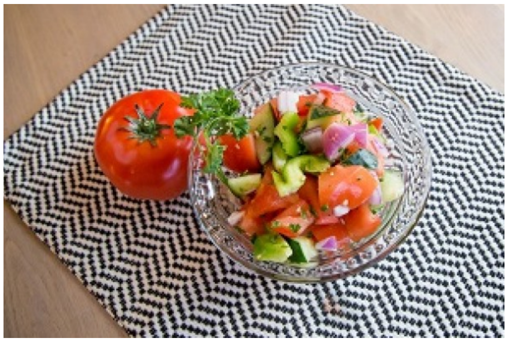

Makes: 12 Servings

This refreshing salad is an excellent source of vitamin C and a great way to take advantage of the vegetables' summer growing season.

Ingredients

Salad

- 4 large tomatoes (cubed)
- 1 large cucumber (chopped)
- 1 cup red onion (chopped)
- 1 cup green pepper (chopped)
- 1/3 cup parsley (chopped)

Dressing

- 1/3 cup apple cider vinegar
- 1 tablespoon olive oil (or oil of your choice)
- 2 cloves garlic (minced)
- 1/2 teaspoon salt
- 1/2 teaspoon ground black pepper
- 1/2 teaspoon sugar

Directions

1. Wash hands with soap and water.
2. In a large bowl, combine the salad ingredients.
3. In a small bowl, mix the dressing ingredients together.
4. Pour the dressing over the salad. Mix well.
5. Refrigerate for at least 1 hour before serving.

Source:

University of Nebraska at Lincoln
Nebraska Nutrition Education Program

Nutrition Information

Serving Size: 3/4 cup

Nutrients	Amount
**Total Calories**	**34**
**Total Fat**	**1 g**
Saturated Fat	0 g
Cholesterol	0 mg
**Sodium**	**103 mg**
**Carbohydrates**	**5 g**
Dietary Fiber	1 g
Total Sugars	3 g
Added Sugars included	0 g
**Protein**	**1 g**
Vitamin D	0 mcg
Calcium	17 mg
Iron	0 mg
Potassium	225 mg

Nutrients will display if the data is available

Please note: nutrient values are subject to change as data is updated

MyPlate Food Groups

Vegetables 3/4 cups

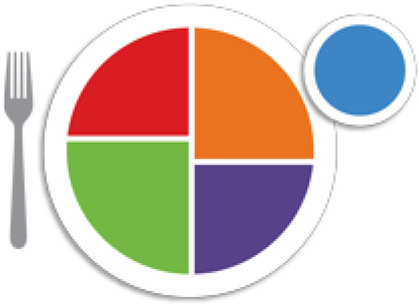

Visit MyPlate.gov

Gingery Quinoa with Green Beans

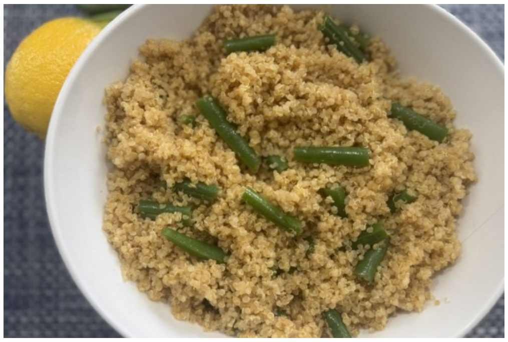

Makes: 4 Servings

Fresh green beans work well when they’re in season. If they’re not, try frozen in this recipe that combines a whole grains and veggies into one satisfying and eye-catching dish.

Ingredients

- 1 cup quinoa (uncooked)
- 1 1/2 cups water or low-sodium broth
- 2 cups green beans (trimmed into 1" pieces)
- 1 tablespoon olive oil or cooking oil
- 1 tablespoon sesame oil
- 2 tablespoons reduced sodium soy sauce
- 2 1/2 tablespoons lemon juice
- 1/4 teaspoon ground ginger

Directions

1. Wash hands with soap and water.
2. Toast quinoa in a dry skillet over medium heat, stirring constantly for about 3 minutes.
3. Transfer toasted quinoa to a medium pot and add water or broth.
4. Bring to a boil, then let simmer until all of the water is absorbed and quinoa is tender (10 to 15 minutes).
5. Set aside and leave uncovered to cool.
6. While quinoa is cooling, place green beans in a microwave-safe dish. Add a small amount of water, cover, and microwave for 4-6 minutes or until just tender.
7. Rinse green beans in cold water to stop the cooking process.
8. In a large bowl, combine the quinoa, green beans, olive oil or cooking oil, sesame oil, soy sauce, lemon juice, and ground ginger. Stir well.
9. Season with salt and pepper, as needed.
10. Serve warm or at room temperature.

Source:

Bronson Wellness Center
Bronson Healthcare - Michigan

Nutrition Information

Serving Size: 1 Cup

Nutrients	Amount
**Total Calories**	**239**
**Total Fat**	**9 g**
Saturated Fat	1 g
Cholesterol	0 mg
**Sodium**	**301 mg**
**Carbohydrates**	**33 g**
Dietary Fiber	6 g
Total Sugars	2 g
Added Sugars included	0 g
**Protein**	**7 g**
Vitamin D	0 mcg
Calcium	56 mg
Iron	3 mg
Potassium	365 mg

Nutrients will display if the data is available

Please note: nutrient values are subject to change as data is updated

MyPlate Food Groups

Vegetables	1/2 cups
Grains	1 1/2 ounces

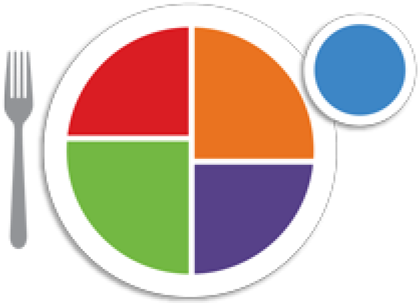

Visit MyPlate.gov

Hummus

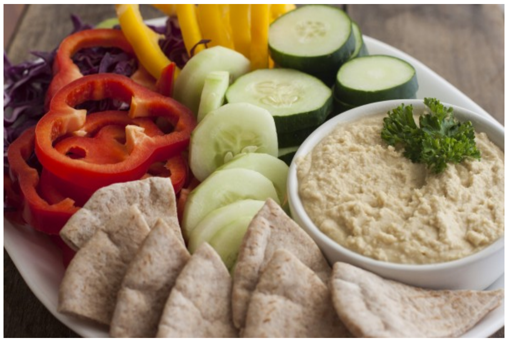

Makes: 8 Servings
Preparation Time: 20 minutes

This classic garbanzo bean dip is easy to make and a versatile dish. Serve hummus with fresh raw vegetables, on a piece of whole wheat pita bread or tortilla, on crackers, or as a sandwich filling.

Ingredients

- 2 cups garbanzo beans, cooked (also known as chickpeas)
- 2 cloves garlic (minced)
- 1/4 cup lemon juice
- 1 tablespoon sesame tahini [sesame paste] (or substitute peanut butter for a sweet taste)
- 2 tablespoons olive oil

Directions

1. Wash hands with soap and water.
2. Mash the garbanzo beans until smooth (if you have a blender, put the beans and lemon juice into it and blend).
3. Add the garlic, lemon juice, tahini and oil. Mix well.

Source:

Simple Healthy Recipes
Oklahoma Nutrition Information and Education
ONIE Project

Nutrition Information

Serving Size: 1/6 of recipe

Nutrients	Amount
**Total Calories**	**117**
**Total Fat**	**6 g**
Saturated Fat	1 g
Cholesterol	0 mg
**Sodium**	**102 mg**
**Carbohydrates**	**13 g**
Dietary Fiber	4 g
Total Sugars	2 g
Added Sugars included	0 g
**Protein**	**4 g**
Vitamin D	0 mcg
Calcium	29 mg
Iron	1 mg
Potassium	141 mg

Nutrients will display if the data is available

Please note: nutrient values are subject to change as data is updated

MyPlate Food Groups

Vegetables	1/4 cups
Protein Foods	1 ounces

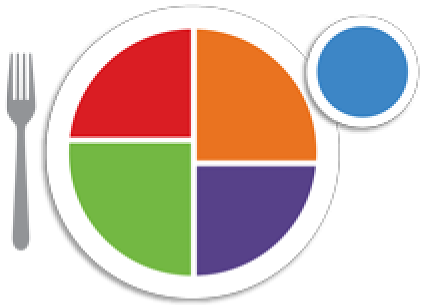

Visit MyPlate.gov

Fish with Spinach

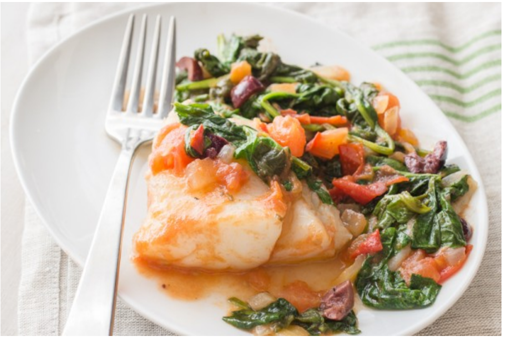

Makes: 4 Servings
Cook Time: 30 minutes

Cod fillets are pan-fried until golden brown and mixed with a rich tomato and spinach sauce for an easy seafood dish. Top with olives and serve with a favorite side for a quick meal.

Ingredients

- 1 tablespoon vegetable oil
- 1 pound skinless cod fillets
- 1 yellow onion (peeled and chopped into 1/4 inch pieces)
- 2 cloves garlic (peeled and minced)
- 2 cups canned low-sodium diced tomatoes (or fresh tomatoes)
- 1/2 cup water
- 2 cups frozen spinach (coarsely chopped)
- 1/4 cup Kalamata olives (or other Greek olives pitted and coarsely chopped)

Directions

1. Put the skillet on the stove over high heat. When it is hot, add 1 1/2 teaspoons oil.
2. Add fish. Cook about 5 minutes per side, until deeply browned. Remove the fish to the plate and cover.
3. Reheat the skillet to medium heat. Add the remaining 1 1/2 teaspoons oil, onion, and garlic. Cook about 7 minutes. Add tomatoes and water. Cook about 10 minutes, until the mixture thickens and turns from bright red to an orange color.
4. Return fish to skillet with the tomato mixture. Cover with spinach and sprinkle with olives. Cover skillet. Cook about 2 minutes over low heat until the spinach is steamed. Serve right away.

Source:

USDA Center for Nutrition Policy and Promotion

Nutrition Information

Serving Size: 4 ounces

Nutrients	Amount
**Total Calories**	**194**
**Total Fat**	**6 g**
Saturated Fat	1 g
Cholesterol	47 mg
**Sodium**	**255 mg**
**Carbohydrates**	**12 g**
Dietary Fiber	6 g
Total Sugars	4 g
Added Sugars included	0 g
**Protein**	**25 g**
Vitamin D	1 mcg
Calcium	220 mg
Iron	4 mg
Potassium	777 mg

Nutrients will display if the data is available

Please note: nutrient values are subject to change as data is updated

MyPlate Food Groups

Vegetables	1 1/4 cups
Protein Foods	3 ounces

Visit MyPlate.gov

Apple Cranberry Salad Toss

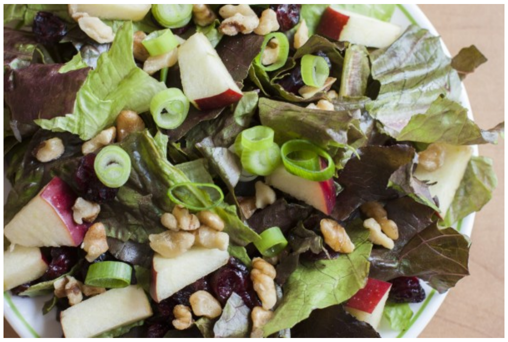

Makes: 8 servings

Enjoy the best of fall flavors with this sweet and tart green salad. Light yet crisp, it's a perfect dish for the autumnal change of weather.

Ingredients

- 1 medium head of lettuce (about 10 cups)
- 2 medium apples, sliced
- 1/2 cup chopped walnuts
- 1 cup dried cranberries
- 1/2 cup green onions, sliced
- 3/4 cup vinaigrette dressing

Directions

1. Wash hands with soap and water.
2. Toss lettuce, apples, walnuts, cranberries, and onions in a large bowl.
3. Add dressing; toss to coat. Serve immediately.

Source:

Creative Recipes for Less Familiar USDA Commodities Used by Household Programs
USDA, Food and Nutrition Service, Food Distribution Service

Nutrition Information

Serving Size: 1/8 of recipe

Nutrients	Amount
**Total Calories**	**174**
**Total Fat**	**10 g**
Saturated Fat	1 g
Cholesterol	0 mg
**Sodium**	**227 mg**
**Carbohydrates**	**22 g**
Dietary Fiber	3 g
Total Sugars	17 g
Added Sugars included	9 g
**Protein**	**2 g**
Vitamin D	0 mcg
Calcium	30 mg
Iron	1 mg
Potassium	206 mg

Nutrients will display if the data is available

Please note: nutrient values are subject to change as data is updated

MyPlate Food Groups

Fruits	1/2 cups
Vegetables	3/4 cups
Protein Foods	1/2 ounces

Visit MyPlate.gov

Classic Macaroni and Cheese

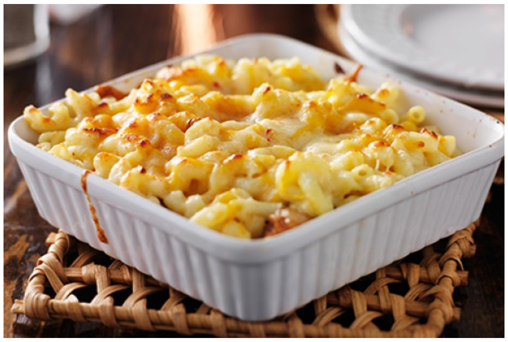

Makes: 8 servings

The ultimate comfort food! This healthier version of a family favorite uses non-fat evaporated milk and low-fat cheese to create its creamy sauce.

Ingredients

- 2 cups macaroni
- 2 sprays of cooking oil spray
- 1/2 cup onion, chopped
- 1/2 cup non-fat evaporated milk
- 1 large egg, beaten
- 1/4 teaspoon black pepper
- 1 1/4 cups finely shredded low-fat sharp cheddar cheese

Directions

1. Wash hands with soap and water.
2. Cook macaroni according to directions. (Do not add salt to the cooking water.) Drain and set aside.
3. Spray a casserole dish with nonstick cooking oil spray.
4. Preheat oven to 350 °F.
5. Lightly spray saucepan with nonstick cooking oil spray.
6. Add onions to saucepan and sauté for about 3 minutes.
7. In another bowl, combine macaroni, onions, and the remaining ingredients and mix thoroughly.
8. Transfer mixture into casserole dish.
9. Bake for 25 minutes or until bubbly. Let stand for 10 minutes before serving.

Source:

Heart Healthy Home Cooking: African American Style
US Department of Health and Human Services
National Heart, Lung, and Blood Institute

Nutrition Information

Serving Size: 1/2 cup, 1/8 of recipe

Nutrients	Amount
**Total Calories**	**170**
**Total Fat**	**3 g**
Saturated Fat	1 g
Cholesterol	28 mg
**Sodium**	**137 mg**
**Carbohydrates**	**25 g**
Dietary Fiber	1 g
Total Sugars	3 g
Added Sugars included	0 g
**Protein**	**11 g**
Vitamin D	0 mcg
Calcium	131 mg
Iron	1 mg
Potassium	121 mg

Nutrients will display if the data is available

Please note: nutrient values are subject to change as data is updated

MyPlate Food Groups

Grains 1 ounces

Dairy 1/4 cups

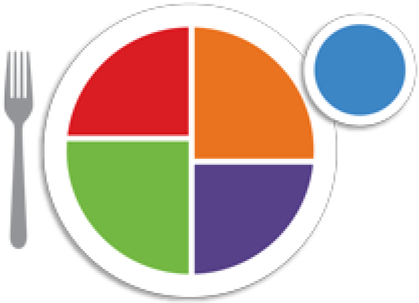

Visit MyPlate.gov

Curried Chicken Salad

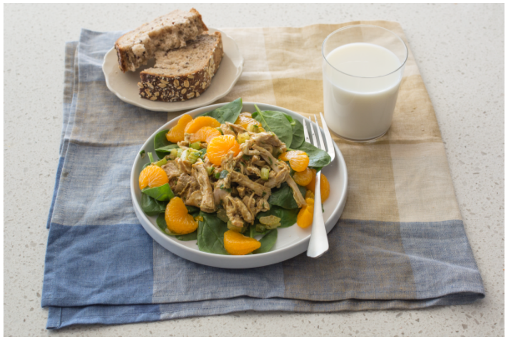

Makes: 4 Servings
Preparation Time: 40 minutes

Shredded chicken is coated with yogurt, curry powder, and cilantro and combined with crisp celery and apples give this salad delicious flavor with a refreshing "crunch." Chill and serve with crackers or use as a sandwich filling.

Ingredients

- 4 cooked cooled chicken thighs (about 3 cups shredded)
- 2 tablespoons vegetable oil
- 1/8 cup low-fat yogurt
- 1 tablespoon curry powder (or more to taste)
- 2 celery stalks (diced or sliced)
- 1 apple (cored and diced)
- 1/4 cup mandarin oranges, canned, drained
- 1/4 cup chopped fresh cilantro (and/or basil leaves)

Directions

1. Put chicken in a bowl and add remaining ingredients. Mix to combine.
2. Cover and refrigerate at least one hour and up three days.

Source:

USDA Center for Nutrition Policy and Promotion

Nutrition Information

Serving Size: 1 cup

Nutrients	Amount
**Total Calories**	**301**
**Total Fat**	**18 g**
Saturated Fat	4 g
Cholesterol	96 mg
**Sodium**	**438 mg**
**Carbohydrates**	**7 g**
Dietary Fiber	2 g
Total Sugars	5 g
Added Sugars included	0 g
**Protein**	**27 g**
Vitamin D	0 mcg
Calcium	48 mg
Iron	2 mg
Potassium	377 mg

Nutrients will display if the data is available

Please note: nutrient values are subject to change as data is updated

MyPlate Food Groups

Fruits	1/4 cups
Vegetables	1/4 cups
Protein Foods	3 1/2 ounces

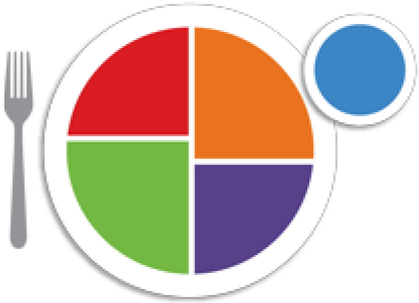

Visit MyPlate.gov

Basic Quiche

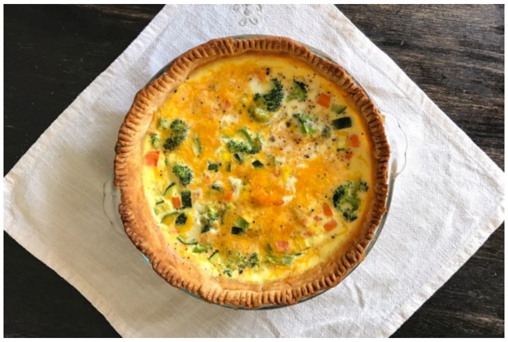

Makes: 6 servings

You can vary this recipe by using whatever vegetables you have on hand (fresh, frozen, or canned)!

Ingredients

- 1 (9-inch) pie crust, baked
- 1 cup vegetables, chopped (broccoli, zucchini, or mushrooms)
- 1/2 cup cheese, shredded
- 3 large eggs, beaten
- 1 cup milk. fat-free
- 1/2 teaspoon salt
- 1/2 teaspoon ground black pepper
- 1/2 teaspoon garlic powder

Directions

1. Wash hands with soap and water.
2. Preheat the oven to 375 °F.
3. Shred the cheese with a grater. Put it in a small bowl for now.
4. Chop the vegetables until you have 1 cup of chopped vegetables.
5. Cook the vegetables until they are cooked, but still crisp.
6. Put the cooked vegetables and shredded cheese into a pie shell.
7. Mix the eggs, milk, salt, pepper, and garlic powder in a bowl.
8. Pour the egg mix over the cheese and vegetables
9. Bake for 30-40 minutes, or until a knife inserted near the center comes out clean.
10. Let the quiche cool for 5 minutes before serving.

Source:

Pennsylvania Nutrition Education Network

Nutrition Information

Serving Size: 1 slice, 1/6 of recipe (128g)

Nutrients	Amount
**Total Calories**	**133**
**Total Fat**	**7 g**
Saturated Fat	3 g
Cholesterol	104 mg
**Sodium**	**338 mg**
**Carbohydrates**	**9 g**
Dietary Fiber	2 g
Total Sugars	3 g
Added Sugars included	0 g
**Protein**	**8 g**
Vitamin D	1 mcg
Calcium	141 mg
Iron	1 mg
Potassium	170 mg

Nutrients will display if the data is available

Please note: nutrient values are subject to change as data is updated

MyPlate Food Groups

Vegetables	1/4 cups
Protein Foods	1/2 ounces
Dairy	1/2 cups

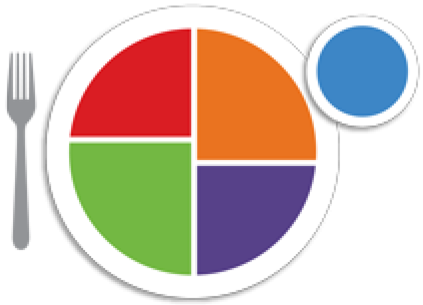

[Visit MyPlate.gov](https://www.myplate.gov)

Grilled Asparagus and Shrimp Quinoa Salad with Lemon Vinaigrette

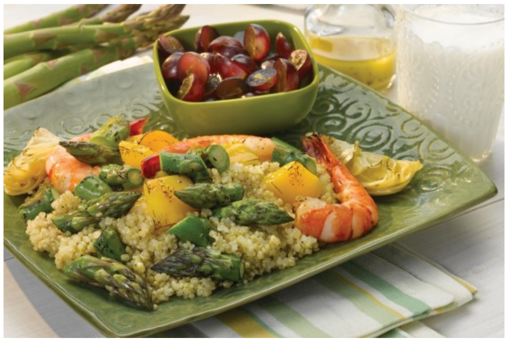

Makes: 4 Servings
Preparation Time: 45 minutes

Grilling brings out the flavor in fresh vegetables like asparagus. Add shrimp, quinoa, and refreshing lemon vinaigrette, and you have a delicious and quick spring or summer salad.

Ingredients

- 2 cups fresh asparagus, large spears (cut into 1" pieces)
- 1/2 yellow or red bell pepper (cut into 1/2" pieces)
- 1 clove garlic (minced)
- 1 14 ounce can quartered artichoke hearts (drained)
- 12 ounces fresh or frozen large raw shrimp (peeled and deveined)
- 1 1/2 cups dry quinoa (cooked according to package directions)

For the Lemon Vinaigrette:

- 1 teaspoon grated lemon peel (optional)
- 3 tablespoons fresh or bottled lemon juice
- 3 tablespoons olive oil
- 1 teaspoon Dijon mustard
- 1/2 teaspoon dried thyme leaves
- 1/2 teaspoon ground black pepper

Directions

1. Place vinaigrette ingredients in a small bowl and whisk; set aside.
2. Cut vegetables as directed.
3. Heat grill and grilling tray.
4. Place vegetables and shrimp in a large bowl; add about 1/3 of the vinaigrette (about 3 tablespoons) and toss.
5. Spread shrimp-vegetable mixture over hot grilling tray.
6. Grill, turning shrimp and vegetables, until the flesh of the shrimp is an opaque color (about 5 to 6 minutes); remove from grill.
7. Serve grill mixture over cooked quinoa, and drizzle with vinaigrette.

Source:

Nutrition Information

Serving Size: 1/4 of the recipe

Nutrients	Amount
**Total Calories**	**460**
**Total Fat**	**16 g**
Saturated Fat	2 g
Cholesterol	115 mg
**Sodium**	**420 mg**
**Carbohydrates**	**51 g**
Dietary Fiber	7 g
Total Sugars	6 g
Added Sugars included	0 g
**Protein**	**29 g**
Vitamin D	0 mcg
Calcium	200 mg
Iron	6 mg
Potassium	860 mg

Nutrients will display if the data is available

Please note: nutrient values are subject to change as data is updated

MyPlate Food Groups

Vegetables	1 1/4 cups
Grains	2 1/2 ounces
Protein Foods	2 1/2 ounces

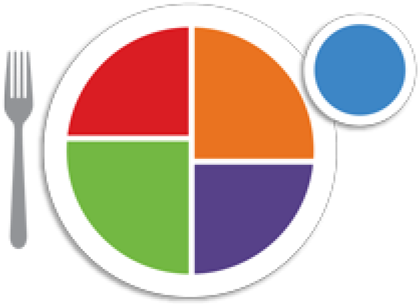

Visit MyPlate.gov

Tuna Salad on Greens

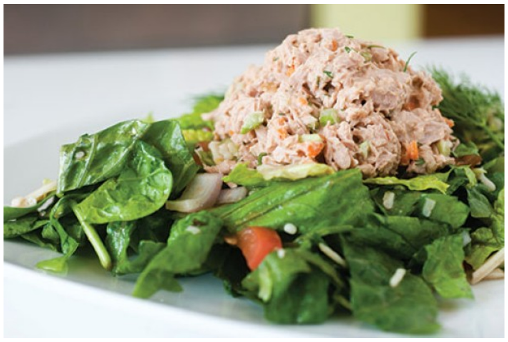

Makes: 4 servings

Tuna salad served on a bed of lettuce and veggies with a whole grain crackers on the side makes this an easy and delicious lunch. Have a piece of fruit as a tasty side dish or dessert.

Ingredients

- 4 whole grain crackers
- 4 cups romaine lettuce (chopped)
- 1 tomato (chopped)
- 1/4 red onion (chopped)
- 1 tablespoon olive oil
- 3 tablespoons vinegar (red wine)
- 1 teaspoon Italian seasoning
- 1 dash black pepper
- 6 ounces tuna, canned in water (drained)
- 1/3 cup mayonnaise, low-fat
- 1/4 cup celery (chopped)
- 1 carrot (peeled and grated)
- 1/4 cup green onion (chopped)

Directions

1. Wash hands with soap and water.
2. Toss lettuce, vegetables, oil, vinegar and herbs in a large salad bowl.
3. Mix ingredients for tuna salad in another medium sized mixing bowl.
4. Assemble salad by placing greens on a large plate. Top with a scoop of tuna salad.
5. Serve crackers on the side.

Source:

Cooking Demo II
Food and Health Communications, Inc.

Nutrition Information

Serving Size: 2 cups, 1/4 of recipe (203g)

Nutrients	Amount
**Total Calories**	**175**
**Total Fat**	**12 g**
Saturated Fat	2 g
Cholesterol	22 mg
**Sodium**	**316 mg**
**Carbohydrates**	**8 g**
Dietary Fiber	2 g
Total Sugars	2 g
Added Sugars included	0 g
**Protein**	**10 g**
Vitamin D	1 mcg
Calcium	50 mg
Iron	2 mg
Potassium	365 mg

Nutrients will display if the data is available

Please note: nutrient values are subject to change as data is updated

MyPlate Food Groups

Vegetables	3/4 cups
Protein Foods	1 1/2 ounces

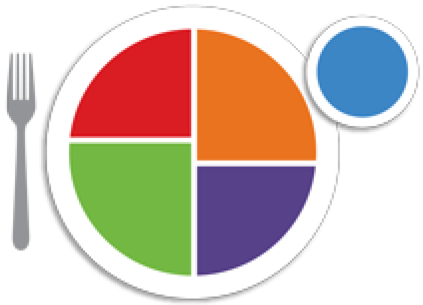

Visit MyPlate.gov

20-Minute Chicken Creole

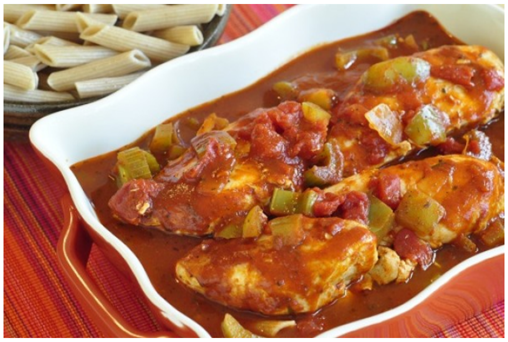

Makes: 8 servings
Cook Time: 20 minutes

This Creole-inspired dish uses chili sauce and cayenne pepper to spice it up. Tomatoes, green pepper, celery, onions and garlic spices also surround the chicken with delicious color. This main dish can be cooked on the stovetop or with an electric skillet.

Ingredients

- 1 tablespoon vegetable oil (or cooking oil of choice)
- 1 pound skinless, boneless chicken breasts
- 1 can (14.5 ounces) low-sodium diced tomatoes
- 1 cup chili sauce
- 1 large green pepper, chopped
- 2 medium celery stalks, chopped
- 1 medium onion, chopped
- 2 garlic cloves, minced
- 1 teaspoon dried basil
- 1 teaspoon dried parsley
- 1/4 teaspoon cayenne pepper
- 1/4 teaspoon salt

Directions

1. Wash hands with soap and water.
2. Heat pan over medium-high heat (350 degrees F in an electric skillet). Add vegetable oil and chicken and cook until the chicken reaches an internal temperature of 165 degrees F (3-5 minutes).
3. Reduce heat to medium (300 degrees F in electric skillet).
4. Add tomatoes with juice, chili sauce, green pepper, celery, onion, garlic, basil, parsley, cayenne pepper, and salt.
5. Bring to a boil; reduce heat to low and simmer, covered for 10-15 minutes.
6. Serve over hot, cooked rice or whole wheat pasta.
7. Refrigerate leftovers within 2 hours.

Source:

Recipe Adapted from: *Food Hero*

Nutrition Information

Serving Size: 1 cup

Nutrients	Amount
**Total Calories**	**112**
**Total Fat**	**4 g**
Saturated Fat	1 g
Cholesterol	35 mg
**Sodium**	**126 mg**
**Carbohydrates**	**6 g**
Dietary Fiber	2 g
Total Sugars	3 g
Added Sugars included	0 g
**Protein**	**14 g**
Vitamin D	0 mcg
Calcium	40 mg
Iron	1 mg
Potassium	468 mg

Nutrients will display if the data is available

Please note: nutrient values are subject to change as data is updated

MyPlate Food Groups

Vegetables	1/2 cups
Protein Foods	1 1/2 ounces

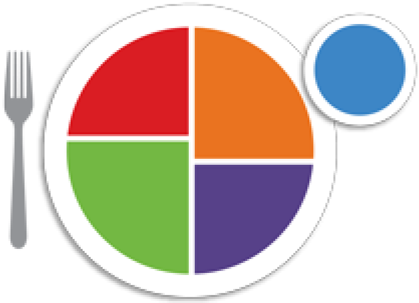

Visit MyPlate.gov

Zingy Lemon Chicken Pasta

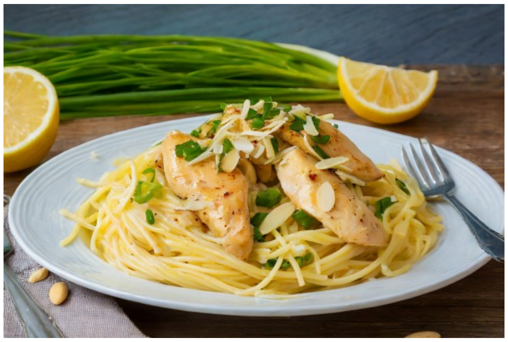

Makes: 4 Servings

This creamy pasta dish with a hint of lemon is sure to be a family favorite. Packed with chicken and topped with crunchy almonds, it can be baked and ready to serve any night of the week.

Ingredients

- 8 ounces whole wheat spaghetti, uncooked
- 1 tablespoon tub margarine
- 1 tablespoon olive oil
- 20 ounces boneless, skinless chicken breast (cut into 3/4-inch pieces)
- 5 green onions (scallions), sliced
- 1 clove large garlic, minced
- 1/4 cup all-purpose flour
- 1/4 teaspoon salt
- 1/8 teaspoon black pepper
- 1/8 teaspoon cayenne pepper
- 1 1/3 cups chicken broth
- 2/3 cup skim milk
- 2 teaspoons prepared mustard
- 1/4 cup freshly squeezed lemon juice

For Topping:

- 1/4 cup chopped almonds
- 2 tablespoons chopped chives or green onions (scallions)
- 1/4 teaspoon paprika (optional)

Directions

1. Combine flour, salt, pepper, and cayenne in a saucepan; gradually add chicken broth and skim milk, stirring with a wire whisk until smooth.
2. Place over medium heat and cook, stirring constantly, until thickened. Add mustard and lemon juice. Allow to cool.
3. Heat margarine and oil in a skillet. Add chicken pieces, green onions, and garlic. Sauté until chicken is just firm and cooked through, about 10 minutes.
4. Prepare pasta according to package directions and drain. Combine sauce, chicken, and pasta mixture.
5. Put into a 2 1/2 quart casserole dish. Sprinkle top with almonds, chopped chives, and paprika (if using).

6. Bake at 375 °F for 10 minutes or until heated through.

Source:

MyPlate National Strategic Partners
The Grain Chain

Nutrition Information

Serving Size: 1 serving

Nutrients	Amount
**Total Calories**	**540**
**Total Fat**	**15 g**
Saturated Fat	3 g
Cholesterol	59 mg
**Sodium**	**324 mg**
**Carbohydrates**	**56 g**
Dietary Fiber	9 g
Total Sugars	4 g
Added Sugars included	0 g
**Protein**	**35 g**
Vitamin D	1 mcg
Calcium	100 mg
Iron	4 mg
Potassium	505 mg

Nutrients will display if the data is available

Please note: nutrient values are subject to change as data is updated

MyPlate Food Groups

Vegetables	1/4 cups
Grains	3 ounces
Protein Foods	3 ounces
Dairy	1/4 cups

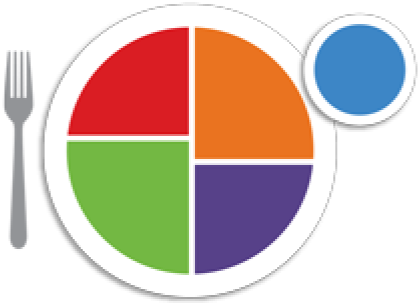

Visit MyPlate.gov

Salmon Patties

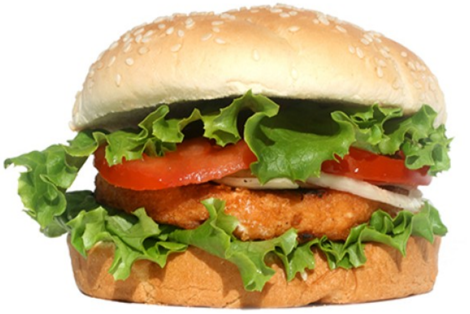

Makes: 6 servings

Tasty patties cooked in a skillet that are perfect topped with fresh lettuce, tomato, and a side of fresh or canned pineapple.

Ingredients

- 1 can salmon, 16 oz
- 1 tablespoon lemon juice
- cold water
- minced celery (1/4 cup, optional)
- green pepper (1 tablespoon, optional)
- 1/2 medium onion
- 2 large eggs
- 1/3 cup bread crumbs or cracker crumbs
- 2 tablespoons all-purpose flour
- 1/8 teaspoon black pepper
- 1 tablespoon vegetable oil

Directions

1. Wash hands with soap and water.
2. Wash fresh vegetables (if using).
3. Collect, mince, and measure all ingredients before starting to prepare the recipe.
4. Open salmon, and drain liquid into a liquid measuring cup. Add lemon juice and cold water to the salmon liquid to make 1/2 cup liquid total, and set aside.
5. Put the salmon in a separate mixing bowl. Mix in the celery (if using), green pepper (if using), and onion.
6. In another small bowl, beat the eggs. Then, add them to the salmon mixture.
7. Add the bread or cracker crumbs, flour, pepper, and the salmon liquid mixture to the salmon mixture, and stir until all ingredients are mixed together.
8. Use 1/3 cup measuring cup to measure salmon mixture. Shape into a 1/2 inch thick patty, and place on a plate. Repeat to make 6 patties.
9. Heat the oil in a skillet over medium heat, then add 3 patties.
10. Cook for about 2 to 3 minutes (or until golden brown) on each side.
11. Remove patties from skillet, and place on a clean paper towel-lined plate to drain. Cook remaining 3 patties, then serve immediately. Refrigerate leftovers within 2 hours. Eat within 3 to 5 days.

Source:

Eat for Health Toolkit
Recipe by Missouri Nutrition Network
Optional additions from ONIE Project & Colorado State University Extension

Nutrition Information

Serving Size: 1 patty

Nutrients	Amount
**Total Calories**	**165**
**Total Fat**	**8 g**
Saturated Fat	1 g
Cholesterol	98 mg
**Sodium**	**316 mg**
**Carbohydrates**	**7 g**
Dietary Fiber	1 g
Total Sugars	1 g
Added Sugars included	0 g
**Protein**	**16 g**
Vitamin D	10 mcg
Calcium	159 mg
Iron	1 mg
Potassium	261 mg

Nutrients will display if the data is available

Please note: nutrient values are subject to change as data is updated

MyPlate Food Groups

Grains	1/2 ounces
Protein Foods	2 1/2 ounces

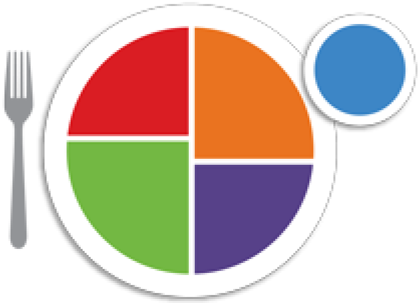

Visit MyPlate.gov

Black Bean Quesadillas

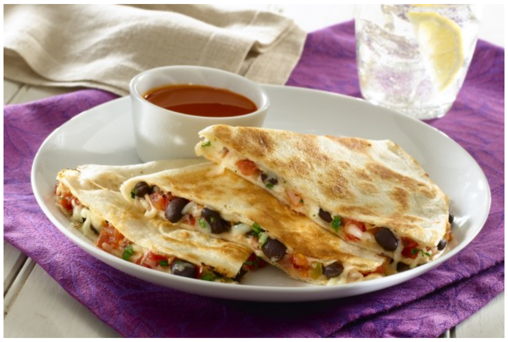

Makes: 8 Servings
Preparation Time: 15 minutes

Enjoy a quesadilla any night of the week! This quesadilla recipe can be served as a tasty lunch, dinner, or side dish.

Ingredients

- 3/4 cup chunky salsa (or pico de gallo)
- 1 can (15.5 oz) low-sodium black beans (drained and rinsed)
- 2 cups shredded reduced-fat Colby & Monterey Jack cheese
- 2 tablespoons fresh cilantro (finely chopped)
- 4 8 inch flour tortillas
- 1/2 teaspoon extra virgin olive oil

Directions

1. Using a small-hole strainer, drain liquid from salsa; discard liquid.
2. Transfer leftover tomato mixture to a medium bowl.
3. Mix in black beans, cheese, and cilantro until combined.
4. Divide black bean mixture evenly over half of each tortilla (about 1/2 cup each).
5. Fold tortillas in half.
6. Heat large griddle or skillet over medium-high heat.
7. Brush with oil.
8. Place filled tortillas on a griddle.
9. Cook, carefully flipping once, until tortillas are gold brown and crisp and cheese filling melts, about 5 minutes.
10. Cut quesadillas into wedges.

Source:

The Best of La Cocina GOYA: Healthy, Tasty, Affordable Latin Cooking/ Lo Mejor de la Cocina GOYA: Cocina Latina Saludable, Rica y Económica
GOYA Foods

Nutrition Information

Nutrients	Amount
**Total Calories**	**160**
**Total Fat**	**5 g**
Saturated Fat	2 g
Cholesterol	5 mg
**Sodium**	**490 mg**
**Carbohydrates**	**25 g**
Dietary Fiber	4 g
Total Sugars	1 g
Added Sugars included	0 g
**Protein**	**7 g**
Vitamin D	0 mcg
Calcium	96 mg
Iron	2 mg
Potassium	287 mg

Nutrients will display if the data is available

Please note: nutrient values are subject to change as data is updated

MyPlate Food Groups

Vegetables	1/4 cups
Grains	1 ounce

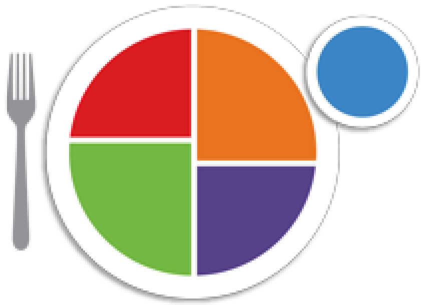

Visit MyPlate.gov

Garden Chili

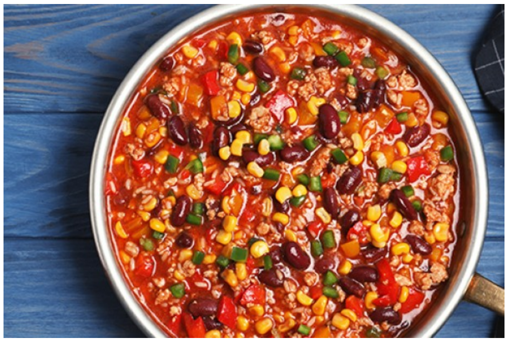

Makes: 4 servings

This quick and easy one pan dish is full of veggies, spices, and flavor. Get your kids involved by letting them wash the vegetables.

Ingredients

- 3/4 pound ground beef (lean, 10% fat)
- 1/2 cup green pepper (chopped)
- 1/2 large onion (chopped)
- 1/2 cup celery (chopped)
- 2/3 cup kidney beans, canned, low-sodium (drained and rinsed)
- 1/2 cup corn (sweet)
- 1 can tomato sauce, low-sodium (8 oz)
- 1 cup fresh tomatoes (chopped)
- 1 dash black pepper
- 1/2 teaspoon garlic powder
- 2 teaspoons chili powder

Directions

1. Wash your hands with soap and water.
2. Place ground beef in a skillet sprayed with non-stick cooking spray. Brown meat over medium-high heat, stirring occasionally to break it into pieces. Drain fat and blot meat with paper towels. Transfer beef into a colander and rinse with very hot water to further remove fat.
3. Add green pepper, onion, and celery. Cook until softened.
4. Add beans, corn, tomato sauce, chopped tomatoes, pepper, garlic powder and chili powder.
5. Cook mixture over low heat for 20 minutes.
6. Serve hot in bowls. Or serve as a dip with baked tortilla chips or on a bun.
7. Cover and refrigerate leftovers within 2 hours.

Source:

Fix it Fresh! Recipe Series
Kansas State University Research and Extension

Nutrition Information

Serving Size: 1/4 of recipe

Nutrients	Amount
**Total Calories**	**224**
**Total Fat**	**8 g**
Saturated Fat	3 g
Cholesterol	54 mg
**Sodium**	**169 mg**
**Carbohydrates**	**17 g**
Dietary Fiber	5 g
Total Sugars	7 g
Added Sugars included	3 g
**Protein**	**20 g**
Vitamin D	0 mcg
Calcium	53 mg
Iron	3 mg
Potassium	758 mg

Nutrients will display if the data is available

Please note: nutrient values are subject to change as data is updated

MyPlate Food Groups

Vegetables	1 1/4 cups
Protein Foods	3 ounces

Visit MyPlate.gov

Grilled Vegetables

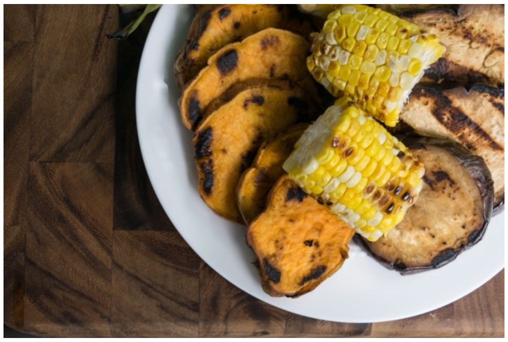

Makes: 6 servings

Cooking out tonight? Try cooking vegetables coated in oil and garlic on the grill for a delicious side dish. If you don't have a grill, you can make these vegetables in the oven.

Ingredients

- 2 tablespoons vegetable oil
- 2 garlic cloves (finely chopped)
- 3 sweet potatoes (cut into 1-inch slices)
- 3 corn cobs (cut into 2-inch sections)
- 1 eggplant (cut into 1/2-inch slices)
- 12 green onions (trimmed)

Directions

1. Wash hands with soap and water.
2. Mix oil and garlic in a large bowl. Add vegetables and toss.
3. Place vegetable on broiler pan or grill. Cook 10 minutes, turning twice until vegetables are tender.
4. Place vegetables on platter. Serve.

Source:

US Department of Health and Human Services
Centers for Disease Control and Prevention (CDC)

Nutrition Information

Serving Size: 1/6 of recipe (254g)

Nutrients	Amount
**Total Calories**	**149**
**Total Fat**	**5 g**
Saturated Fat	1 g
Cholesterol	0 mg
**Sodium**	**48 mg**
**Carbohydrates**	**25 g**
Dietary Fiber	4 g
Total Sugars	7 g
Added Sugars included	0 g
**Protein**	**3 g**
Vitamin D	0 mcg
Calcium	45 mg
Iron	1 mg
Potassium	459 mg

Nutrients will display if the data is available

Please note: nutrient values are subject to change as data is updated

MyPlate Food Groups

Vegetables 1 cups

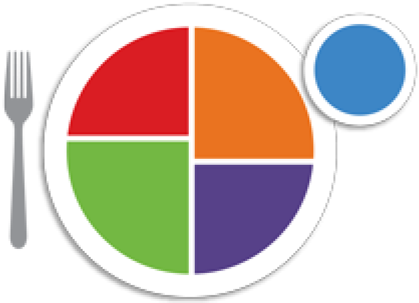

Visit MyPlate.gov

Angel Food Pastry with Fresh Berries and Whipped Cream

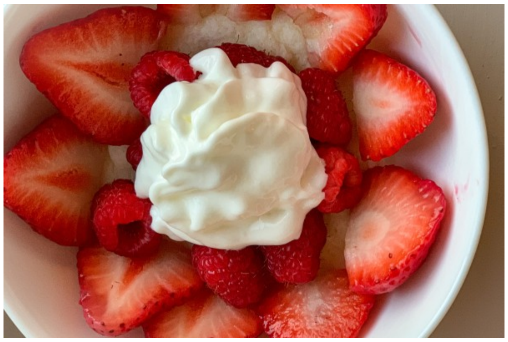

Makes: 1 Servings

Light and fluffy angel food cake is mixed with yogurt and a sprinkle of berries on top.

Ingredients

- 1 ounce angel food cake mix (3 tablespoons)
- 1/8 cup low-fat vanilla yogurt (2 tablespoons)
- 1/4 cup raspberries
- 2 strawberries, sliced
- 1 1/2 tablespoons lite whipped topping

Directions

1. Wash hands with soap and water.
2. Scoop 3 tablespoons of angel food cake mix into a microwave-safe cup or mug.
3. Add 2 tablespoons of vanilla yogurt and stir.
4. Place in the center of the microwave oven and microwave for 1 minute.
5. Take out of microwave and let cool for 1 minute.
6. Using a knife, cut around the sides of the cup to loosen the cake and "dump" it on a plate.
7. Dole 1 tablespoon of whipped topping on top of the cake.
8. Sprinkle raspberries around the angel cake and add sliced strawberries to top.
9. Enjoy.

Source:

Community Services for Autistic Adults and Children (CSAAC)

Nutrition Information

Serving Size: 1 Angel Food Pastry

Nutrients	Amount
**Total Calories**	**174**
**Total Fat**	**2 g**
Saturated Fat	1 g
Cholesterol	2 mg
**Sodium**	**260 mg**
**Carbohydrates**	**37 g**
Dietary Fiber	3 g
Total Sugars	27 g
Added Sugars included	10 g
**Protein**	**4 g**
Vitamin D	0 mcg
Calcium	116 mg
Iron	0 mg
Potassium	204 mg

Nutrients will display if the data is available

Please note: nutrient values are subject to change as data is updated

MyPlate Food Groups

Fruits 1/2 cups

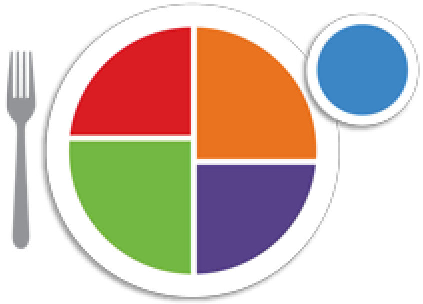

Visit MyPlate.gov

Tropical Yogurt Pops

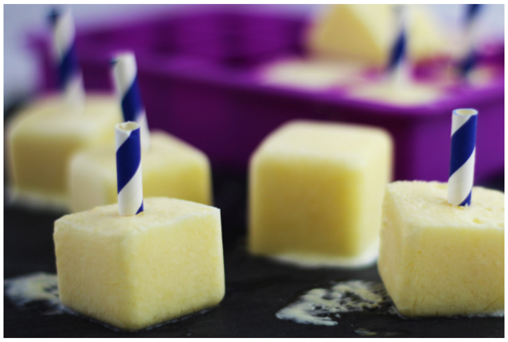

Makes: 4 Servings

Enjoy a tropical treat with these frozen yogurt pops! They are fun to make and have 1/4 cup of fruit per serving!

Ingredients

- 1 cup low-fat vanilla yogurt
- 1/2 cup mango pieces (fresh or frozen)
- 1/2 cup pineapple pieces (fresh, canned, or frozen)
- 1/4 cup cup coconut water
- 1 ice cube tray (or paper cups)

Directions

1. Place all ingredients into blender.
2. Blend on high until smooth.
3. Pour into 4 small paper cups (or 8 ice cubes) and place in popsicle sticks (or cut paper straws).
4. Freeze.
5. Enjoy as a frozen treat!

Source:

USDA Center for Nutrition Policy and Promotion

Nutrition Information

Nutrients	Amount
**Total Calories**	**65**
**Total Fat**	**1 g**
Saturated Fat	1 g
Cholesterol	4 mg
**Sodium**	**43 mg**
**Carbohydrates**	**11 g**
Dietary Fiber	1 g
Total Sugars	10 g
Added Sugars included	0 g
**Protein**	**4 g**
Vitamin D	0 mcg
Calcium	N/A
Iron	N/A
Potassium	N/A

Nutrients will display if the data is available

Please note: nutrient values are subject to change as data is updated

MyPlate Food Groups

Fruits 1/4 cups
Dairy 1/4 cups

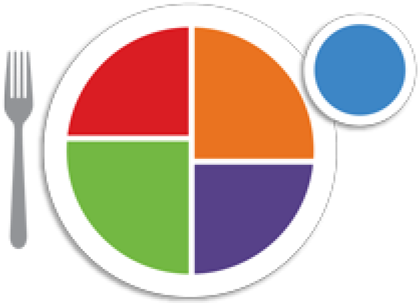

[Visit MyPlate.gov]

Chocolate Chip Yogurt Cookies

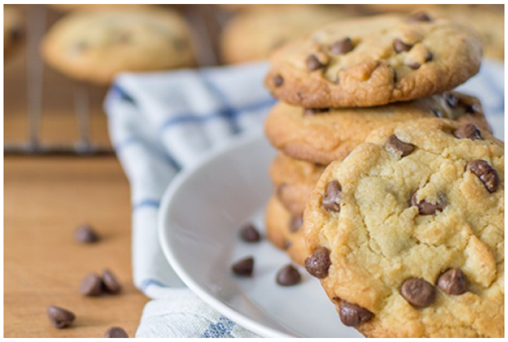

Makes: 36 servings

Add extra calcium to your cookies with yogurt. These cookies also use whole wheat flour which adds fiber and other nutrients.

Ingredients

- 1/2 cup sugar
- 1/2 cup brown sugar, firmly packed
- 1/2 cup margarine (1 stick)
- 1/2 cup plain yogurt, non-fat
- 1 1/2 teaspoons vanilla
- 3/4 cup all-purpose flour
- 1 cup whole wheat flour
- 1/2 teaspoon baking soda
- 1/2 cup chocolate chips (miniature, or carob chips)

Directions

1. Wash hands with soap and water.
2. Heat oven to 375 °F.
3. In a large bowl combine sugar, brown sugar and margarine; beat until light and fluffy.
4. Add yogurt and vanilla; blend well. Stir in flour and baking soda; mix well. Stir in chocolate chips.
5. Drop dough by rounded teaspoonfuls 2 inches apart onto un-greased cookie sheets. Bake at 375 °F for 8 to 12 minutes or until light and golden brown.
6. Cool 1 minute, remove from cookie sheets.

Source:

Eat Smart New York!
Cornell University Cooperative Extension

Nutrition Information

Serving Size: 1 cookie

Nutrients	Amount
**Total Calories**	**79**
**Total Fat**	**3 g**
Saturated Fat	1 g
Cholesterol	0 mg
**Sodium**	**42 mg**
**Carbohydrates**	**12 g**
Dietary Fiber	1 g
Total Sugars	7 g
Added Sugars included	7 g
**Protein**	**1 g**
Vitamin D	0 mcg
Calcium	12 mg
Iron	0 mg
Potassium	37 mg

Nutrients will display if the data is available

Please note: nutrient values are subject to change as data is updated

MyPlate Food Groups

Grains 1/2 ounces

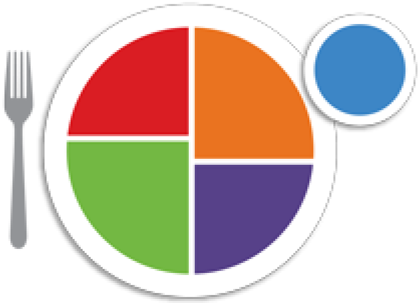

Visit MyPlate.gov

Apple-Stuffed Squash

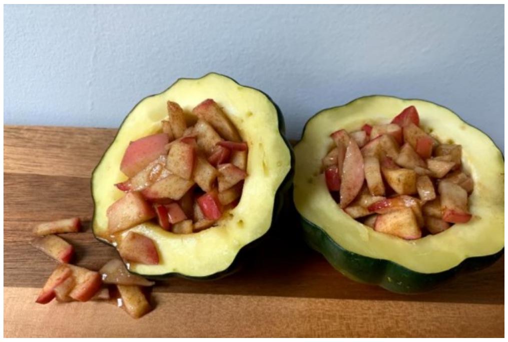

Makes: 8 Servings

All you need is a microwave to cook this delicious and seasonal side dish.

Ingredients

- 4 acorn squashes, washed
- 1 tablespoon butter (or margarine)
- 2 apples, chopped
- 2 teaspoons brown sugar, packed
- 1/2 teaspoon cinnamon

Directions

1. Wash hands with soap and water.
2. Cut squash into halves and remove seeds. Place in a glass dish, cover with plastic wrap and microwave on high for 5 minutes.
3. Melt butter in a separate bowl in the microwave. Mix in apples, sugar, and cinnamon. Microwave for 1 1/2 minutes.
4. Spoon apple filling into each squash half. Cover and microwave on high for 3-5 minutes until squash and apples are tender.
5. Serve warm.

Source:

University of Maryland Cooperative Extension
SNAP-Ed Program

Nutrition Information

Serving Size: 1/2 squash with filling, 1/8 of recipe

Nutrients	Amount
**Total Calories**	**122**
**Total Fat**	**2 g**
Saturated Fat	1 g
Cholesterol	4 mg
**Sodium**	**7 mg**
**Carbohydrates**	**28 g**
Dietary Fiber	4 g
Total Sugars	5 g
Added Sugars included	1 g
**Protein**	**2 g**
Vitamin D	0 mcg
Calcium	76 mg
Iron	2 mg
Potassium	787 mg

Nutrients will display if the data is available

Please note: nutrient values are subject to change as data is updated

MyPlate Food Groups

Fruits	1/4 cups
Vegetables	2 cups

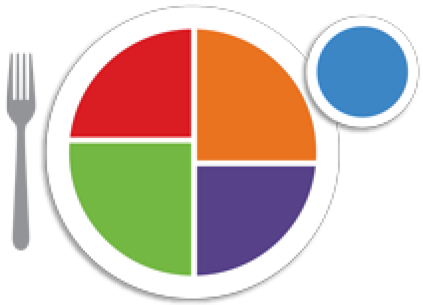

Visit MyPlate.gov

Rainbow Veggie Salad

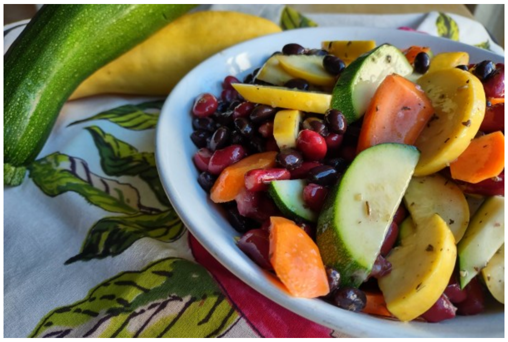

Makes: 10 servings

This easy and colorful salad tastes best after marinating it overnight. Enjoy it as a side dish at lunch or dinner.

Ingredients

- 1 can (15.5 ounces) red kidney beans, low-sodium (drained and rinsed)
- 1 can (15.5 ounces) black beans, low-sodium (drained and rinsed)
- 3 carrots (scrubbed and sliced)
- 1 medium yellow squash (washed and sliced)
- 1 medium zucchini (washed and sliced)
- 1/2 cup light Italian dressing
- 1/2 teaspoon black pepper

Directions

1. Wash hands with soap and water.
2. Mix all the vegetables together in a large bowl.
3. Pour dressing over vegetables.
4. Sprinkle with pepper.
5. Stir gently, coating all vegetables.
6. Cover and refrigerate at least 8 hours.

Source:

2009 Recipe Calendar
University of Maryland Extension
Food Supplement Nutrition Education Program

Nutrition Information

Serving Size: 2/3 cup (106g)

Nutrients	Amount
**Total Calories**	**95**
**Total Fat**	**1 g**
Saturated Fat	0 g
Cholesterol	0 mg
**Sodium**	**257 mg**
**Carbohydrates**	**18 g**
Dietary Fiber	6 g
Total Sugars	4 g
Added Sugars included	0 g
**Protein**	**6 g**
Vitamin D	0 mcg
Calcium	44 mg
Iron	2 mg
Potassium	424 mg

Nutrients will display if the data is available

Please note: nutrient values are subject to change as data is updated

MyPlate Food Groups

Vegetables	1 cups
Protein Foods	2 ounces

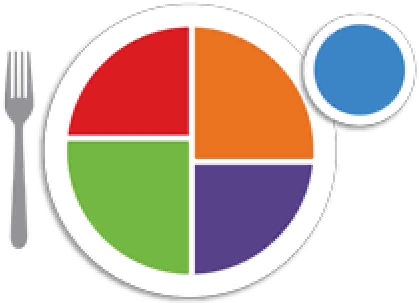

Visit MyPlate.gov

Grilled Fruit

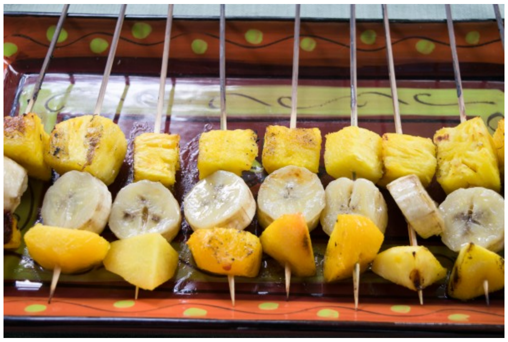

Makes: 3 Servings

Fruit kabobs cooked on the grill make a tasty and healthy dessert.

Ingredients

- 1 cup pineapple chunks (fresh or canned)
- 1 peach (cubed)
- 1 banana (sliced)

Directions

1. Wash hands with soap and water.
2. Place fruit chunks on a skewer to make kabobs.
3. Grill or broil on low heat until the fruit is hot and slightly golden.

Source:

University of Wyoming Extension
Cent$ible Nutrition Program

Nutrition Information

Serving Size: 1/3 of recipe

Nutrients	Amount
**Total Calories**	**104**
**Total Fat**	**0 g**
Saturated Fat	0 g
Cholesterol	0 mg
**Sodium**	**1 mg**
**Carbohydrates**	**27 g**
Dietary Fiber	2 g
Total Sugars	21 g
Added Sugars included	0 g
**Protein**	**1 g**
Vitamin D	0 mcg
Calcium	17 mg
Iron	0 mg
Potassium	337 mg

Nutrients will display if the data is available

Please note: nutrient values are subject to change as data is updated

MyPlate Food Groups

Fruits 1 cups

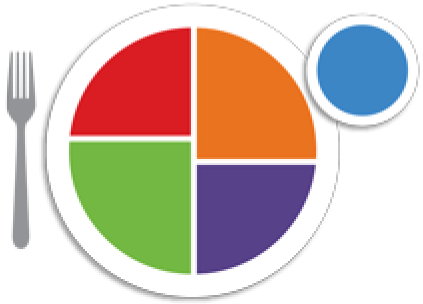

Visit MyPlate.gov

Grilled Vegetable Packets

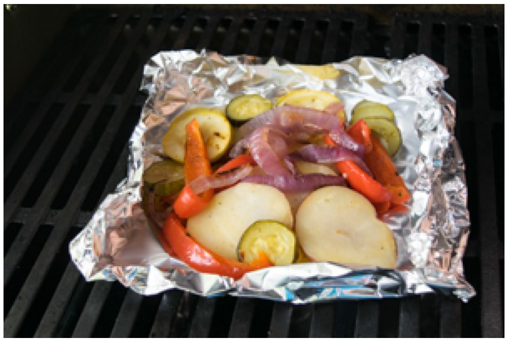

Makes: 5 servings

Veggies are wrapped in foil and cooked on the grill for an easy side dish to your cookout. Don't have a grill? This recipe can also be made in the oven.

Ingredients

- 2 small zucchinis (sliced)
- 2 small yellow squashes (sliced)
- 4 small red potatoes (scrubbed well and sliced)
- 1/2 red onion (sliced)
- 1/2 red or green bell pepper (sliced)
- 1/4 cup light Italian salad dressing
- salt and pepper (optional, to taste)

Directions

1. Wash hands with soap and water.
2. Heat grill to medium heat or 350 °F.
3. Wash vegetables and slice.
4. Toss in a large bowl. Add dressing and toss until all vegetables are coated.
5. Tear 2 large squares of aluminum foil and place half of the vegetable mixture on each piece. Place an equal piece of foil over the top of vegetable mixture and fold bottom piece with top sheet to form a packet.
6. Place on heated grill for 20 to 30 minutes or until the potatoes are tender. If you don't have a grill, bake Veggie Packets in the oven at 400 °F for 20 to 30 minutes.
7. Before you open the packets, poke holes in the foil with a fork. Be very careful opening the foil as the steam will be very hot and could burn you!
8. Empty vegetables onto serving plate or serve from foil packets.

Source:

Connecticut Food Policy Council

Nutrition Information

Serving Size: 3/4 cup (316g)

Nutrients	Amount
**Total Calories**	**133**
**Total Fat**	**0 g**
Saturated Fat	0 g
Cholesterol	0 mg
**Sodium**	**144 mg**
**Carbohydrates**	**29 g**
Dietary Fiber	4 g
Total Sugars	5 g
Added Sugars included	0 g
**Protein**	**4 g**
Vitamin D	0 mcg
Calcium	38 mg
Iron	2 mg
Potassium	868 mg

Nutrients will display if the data is available

Please note: nutrient values are subject to change as data is updated

MyPlate Food Groups

Vegetables 2 cups

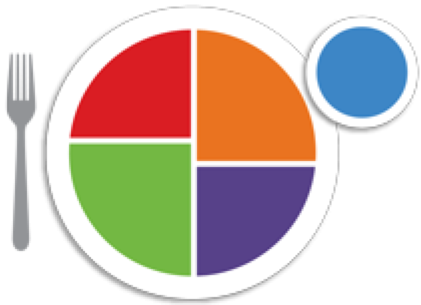

[Visit MyPlate.gov]

Skinny Pizza

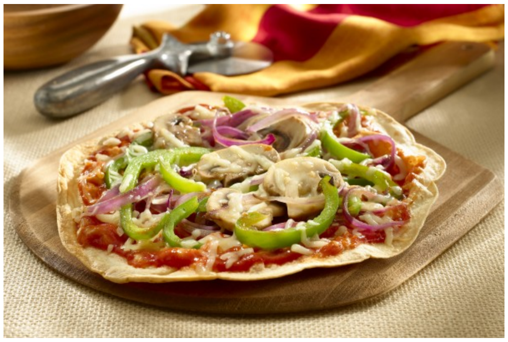

Makes: 4 Servings
Preparation Time: 30 minutes

Next time they ask for pizza, you can feel good about saying "yes!" Flour tortillas make for a crispy crust, perfect for loading with low-sodium tomato sauce, low-fat cheese, and lots of fresh veggies.

Ingredients

- 4 6" flour tortillas
- 1/2 teaspoon extra virgin olive oil
- 2 cups sliced mushrooms (white button or baby Portobello)
- 1 green bell pepper (thinly sliced, about 1 cup)
- 1 red onion (thinly sliced, about 1 cup)
- 2 teaspoons minced garlic
- 1/2 cup low-sodium tomato sauce
- 1/2 cup shredded fat-free mozzarella cheese
- 2 teaspoons grated reduced-fat Parmesan cheese

Directions

1. Heat oven to 400 °F.
2. Place tortillas on 2 large baking sheets.
3. Cook, flipping once, until crisp (about 10 minutes). Set aside.
4. Meanwhile, heat oil in large skillet over medium heat. Add mushrooms, peppers, onions, and garlic.
5. Cook until all vegetables are soft and tender (about 10 minutes). Set aside.
6. Spread tortilla crust with 2 tablespoons tomato sauce, 1/4 cup vegetable mixture, 2 tablespoons mozzarella cheese, and 1/2 teaspoon of Parmesan cheese. Repeat with remaining crusts and topping ingredients.
7. Transfer pizzas to same baking sheets.
8. Cook until cheese is melted and edges of tortillas are golden brown (about 10 minutes).

Source:

The Best of La Cocina GOYA: Healthy, Tasty, Affordable Latin Cooking/ Lo Mejor de la Cocina GOYA: Cocina Latina Saludable, Rica y Económica
GOYA Foods

Nutrition Information

Nutrients	Amount
**Total Calories**	**190**
**Total Fat**	**5 g**
Saturated Fat	1 g
Cholesterol	5 mg
**Sodium**	**450 mg**
**Carbohydrates**	**26 g**
Dietary Fiber	3 g
Total Sugars	6 g
Added Sugars included	2 g
**Protein**	**9 g**
Vitamin D	0 mcg
Calcium	200 mg
Iron	2 mg
Potassium	375 mg

Nutrients will display if the data is available

Please note: nutrient values are subject to change as data is updated

MyPlate Food Groups

Vegetables	1 1/4 cups
Grains	1 1/2 ounces
Dairy	1/2 cups

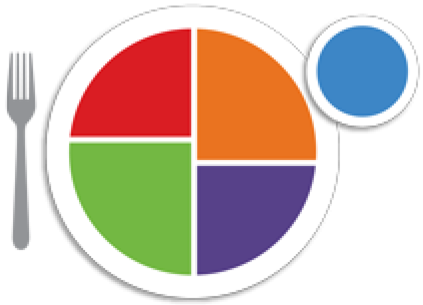

Visit MyPlate.gov

Rice-Crusted Pizza

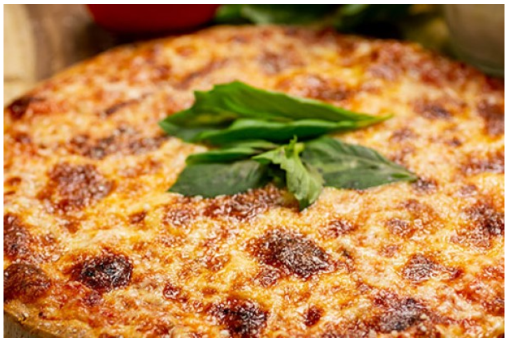

Makes: 12 servings

A fun and unique twist on pizza using rice as the crust. Enjoy this main dish with all of your favorite toppings!

Ingredients

- 2 cups rice (cooked)
- 16 ounces mozzarella cheese, part skim
- 1 egg
- 1/4 teaspoon salt
- 2 cups tomato purée, low-sodium

Directions

1. Wash hands with soap and water.
2. Heat oven to 350 °F. Grease a 12-inch pizza pan or baking sheet.
3. Cook rice following directions on the package; set aside.
4. Grate cheese; set aside.
5. Crack egg and place in mixing bowl, stirring to blend. Measure and add 2 cups cooked rice, 1 cup grated cheese, and salt. Mix well to combine ingredients.
6. Spread rice mixture in prepared pan, pressing firmly and making outer edge slightly raised.
7. Spread tomato purée evenly over rice mixture.
8. Place pan in oven. Bake for 25 minutes.
9. Remove pan from oven sprinkle pizza with remaining cheese. Bake 5 minutes longer or until cheese melts.
10. Remove pizza from oven. Cut into 12 slices.

Create-a-Flavor Changes:

- Try different kinds of rice.
- Try different kinds of cheese.
- Use sliced or chopped green peppers, onions, mushrooms, or other vegetables as toppings.

Source:

Cooking Up Fun - A Pyramid of Snacks
Cornell University Cooperative Extension

Nutrition Information

Serving Size: 1 slice, 1/12 of recipe (110g)

Nutrients	Amount
**Total Calories**	**175**
**Total Fat**	**9 g**
Saturated Fat	4 g
Cholesterol	37 mg
**Sodium**	**313 mg**
**Carbohydrates**	**12 g**
Dietary Fiber	1 g
Total Sugars	2 g
Added Sugars included	3 g
**Protein**	**12 g**
Vitamin D	0 mcg
Calcium	292 mg
Iron	1 mg
Potassium	181 mg

Nutrients will display if the data is available

Please note: nutrient values are subject to change as data is updated

MyPlate Food Groups

Vegetables	1/4 cups
Grains	1/2 ounces
Dairy	1 cups

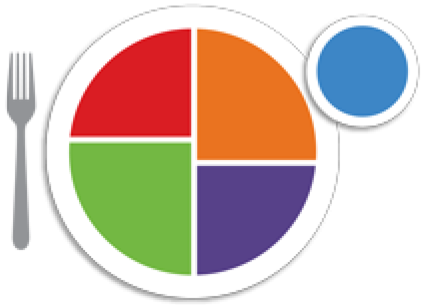

Visit MyPlate.gov

Curried Chickpea Salad

Makes: 6 Servings
Preparation Time: 10 minutes

This dish can be served on whole wheat bread, on a bed of lettuce, or as a dip with your favorite veggies!

Ingredients

- 2 cans (15 ounces each) chickpeas, low-sodium, rinsed and drained
- 1/2 cup celery, diced
- 1/2 cup apple, diced
- 1/4 cup red onion, diced
- 1/4 cup cranberries, dried
- 1/4 cup mayonnaise, low fat
- 1 teaspoon dijon mustard
- 2 teaspoons curry powder
- 1/4 teaspoon thyme, dried
- 1/8 teaspoon black pepper, ground
- salt (1/2 teaspoon, optional)

Directions

1. Wash hands with soap and water.
2. Add the chickpeas to a large bowl and mash with a potato masher. You can mash them to be as smooth or coarse as you desire.
3. Add all of the remaining ingredients to the bowl and mix until everything is combined.

Source:

Penn State Extension
Nutrition Links Program

Nutrition Information

Serving Size: 1/2 cup

Nutrients	Amount
**Total Calories**	**181**
**Total Fat**	**6 g**
Saturated Fat	1 g
Cholesterol	N/A
**Sodium**	**282 mg**
**Carbohydrates**	**26 g**
Dietary Fiber	7 g
Total Sugars	8 g
Added Sugars included	3 g
**Protein**	**7 g**
Vitamin D	N/A
Calcium	59 mg
Iron	2 mg
Potassium	N/A

Nutrients will display if the data is available

Please note: nutrient values are subject to change as data is updated

MyPlate Food Groups

Fruits	1/4 cups
Vegetables	1 cups
Protein Foods	3 ounces

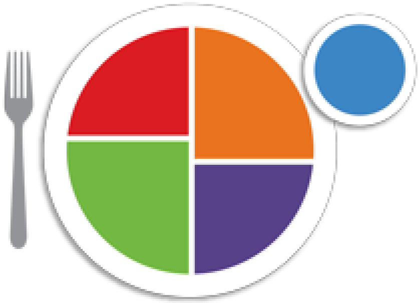

Visit MyPlate.gov

Roasted Chickpeas (Garbanzo Beans)

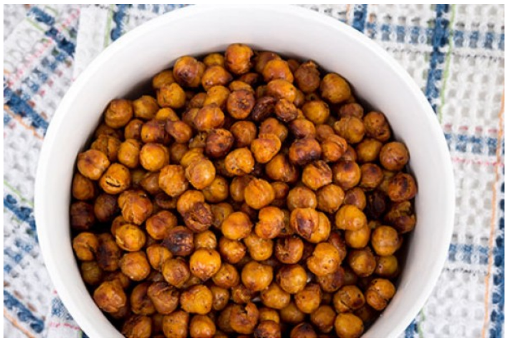

Makes: 4 Servings
Cook Time: 45 minutes

These crunchy chickpeas make a great on-the-go snack or a healthy topping to your favorite salad. Experiment with different spices to see which ones you like best.

Ingredients

- 1 can (15 ounces) low-sodium garbanzo beans, drained and rinsed
- 1 tablespoon canola oil (or cooking oil of choice)
- 1/8 teaspoon salt
- spices of your choice, optional (try garlic, paprika, sage, etc.)

Directions

1. Wash hands with soap and water.
2. Preheat oven to 400 degrees F.
3. Drain and rinse the beans until there is no foamy residue. Pick out the skins and discard. Wrap the drained beans in a clean towel to dry thoroughly.
4. Measure oil into a 9x13-inch baking dish or foil lined pan. Pour beans into dish, sprinkle with salt and spices. Mix oil and spices well to coat all the beans.
5. Bake for 45 to 60 minutes, stirring or shaking every 15 minutes. Longer makes more crunch, but watch to prevent burning!

Source:

USDA Supplemental Nutrition Assistance Program (SNAP)

Nutrition Information

Serving Size: 1/4 cup

Nutrients	Amount
**Total Calories**	**124**
**Total Fat**	**5 g**
Saturated Fat	0 g
Cholesterol	0 mg
**Sodium**	**216 mg**
**Carbohydrates**	**14 g**
Dietary Fiber	5 g
Total Sugars	3 g
Added Sugars included	0 g
**Protein**	**5 g**
Vitamin D	0 mcg
Calcium	37 mg
Iron	1 mg
Potassium	153 mg

Nutrients will display if the data is available

Please note: nutrient values are subject to change as data is updated

MyPlate Food Groups

Vegetables	1/2 cups
Protein Foods	2 1/2 ounces

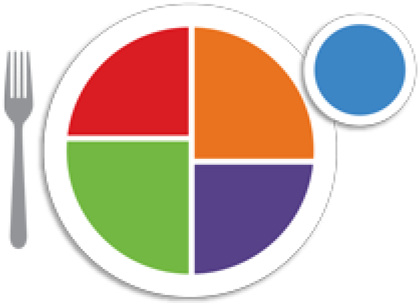

Visit MyPlate.gov

Heavenly Deviled Eggs

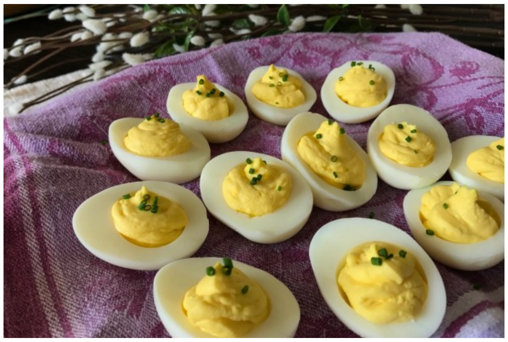

Makes: 6 Servings

Versatile and easy to prepare, these deviled eggs are great as an appetizer, side dish, or snack.

Ingredients

- 6 eggs (in shell)
- 2 tablespoons light mayonnaise
- 1 teaspoon mustard

Directions

1. Wash hands with soap and water.
2. Put eggs into a saucepan. Cover with cold water.
3. Bring eggs to a simmer (small bubbles) and cook for 12 minutes.
4. Remove from the heat and drain.
5. Crack eggs under cold water and allow to cool. Remove shells.
6. Split eggs in half, lengthwise and remove yolks.
7. Put yolks, dressing and mustard into a small zipper-lock plastic bag to mix.
8. Cut a small hole in a lower corner of the bag. Squeeze mixture into egg white halves. Garnish as desired.

Source:

Simple Healthy Recipes
Oklahoma Nutrition Information and Education
ONIE Project

Nutrition Information

Serving Size: 2 prepared egg halves, 1/6 of recipe

Nutrients	Amount
**Total Calories**	**90**
**Total Fat**	**6 g**
Saturated Fat	2 g
Cholesterol	212 mg
**Sodium**	**174 mg**
**Carbohydrates**	**1 g**
Dietary Fiber	0 g
Total Sugars	1 g
Added Sugars included	0 g
**Protein**	**6 g**
Vitamin D	40 mcg
Calcium	25 mg
Iron	1 mg
Potassium	65 mg

Nutrients will display if the data is available

Please note: nutrient values are subject to change as data is updated

MyPlate Food Groups

Protein Foods 1 ounce

[Visit MyPlate.gov](https://www.myplate.gov)

Broccoli Potato Soup

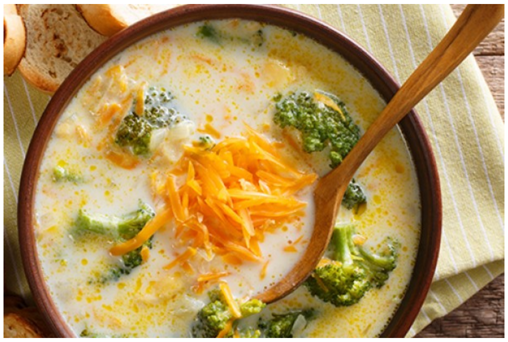

Makes: 4 servings

A tasty and made-from-scratch soup that comes together quickly with the help of instant mashed potatoes and powdered milk.

Ingredients

- 4 cups broccoli, chopped
- 1 small onion, chopped
- 4 cups chicken or vegetable broth, low-sodium
- 1 cup non-fat evaporated milk
- 1/2 cup instant mashed potatoes, prepare with water for 1 cup potatoes (or 1 cup leftover mashed potatoes.)
- salt and pepper (to taste, optional)
- 1/4 cup cheddar cheese, shredded

Directions

1. Wash hands with soap and water.
2. Combine broccoli, onion, and broth in large sauce pan.
3. Bring to a boil.
4. Reduce heat. Cover and simmer about 10 minutes or until vegetables are tender.
5. Add milk to soup. Slowly stir in potatoes.
6. Cook, stirring constantly, until bubbly and thickened.
7. Season with salt and pepper; stir in a little more milk or water if soup starts to become too thick.
8. Ladle into serving bowls.
9. Sprinkle about 1 tablespoon cheese over each serving.

Source:

Don't Play With Your Food: Fall and Winter Cookbook
Arizona Nutrition Network

Nutrition Information

Serving Size: 1/4 of recipe

Nutrients	Amount
**Total Calories**	**178**
**Total Fat**	**4 g**
Saturated Fat	2 g
Cholesterol	10 mg
**Sodium**	**225 mg**
**Carbohydrates**	**23 g**
Dietary Fiber	3 g
Total Sugars	10 g
Added Sugars included	0 g
**Protein**	**15 g**
Vitamin D	1 mcg
Calcium	295 mg
Iron	2 mg
Potassium	806 mg

Nutrients will display if the data is available

Please note: nutrient values are subject to change as data is updated

MyPlate Food Groups

Vegetables	1 1/2 cups
Dairy	3/4 cups

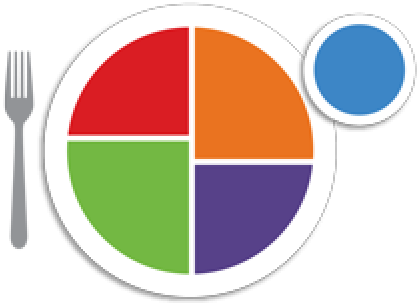

[Visit MyPlate.gov](https://www.myplate.gov)

Marinated Beef

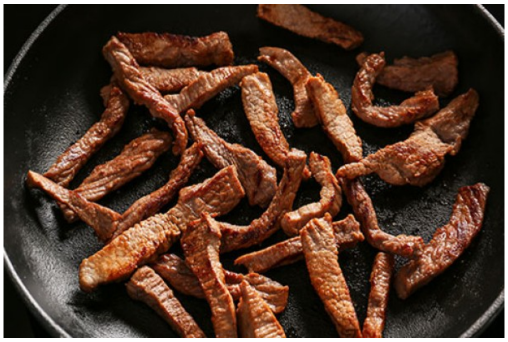

Makes: 4 servings

Tangy, marinated beef is full of flavor and pairs well with roasted potatoes, steamed broccoli, and fresh, sliced tomatoes.

Ingredients

- 12 ounces beef round steak
- 1 garlic clove
- 2 tablespoons lemon juice
- 4 tablespoons vegetable oil, divided (or cooking oil of choice)
- 1/2 teaspoon salt
- 1/2 teaspoon black pepper

Directions

1. Wash hands with soap and water.
2. Using a cutting board and sharp knife, cut round steak across the grain into thin strips about 1/2 inch wide and 2 to 3 inches long.
3. In a medium glass mixing bowl, combine garlic, lemon juice, 2 tablespoons of vegetable oil, salt, and pepper.
4. Add beef strips and stir to coat with the oil mixture (marinade sauce). Cover bowl with plastic wrap and refrigerate for about 2 hours.
5. In a medium skillet over medium-high heat, heat 2 tablespoons of the oil for 1-2 minutes until hot.
6. Drain marinade from beef. Put beef in skillet, stir and cook for 5 to 7 minutes or until meat is thoroughly browned.

Source:

Kids a Cookin'
Kansas Family Nutrition Program

Nutrition Information

Serving Size: 1/4 of recipe

Nutrients	Amount
**Total Calories**	**219**
**Total Fat**	**18 g**
Saturated Fat	3 g
Cholesterol	42 mg
**Sodium**	**310 mg**
**Carbohydrates**	**1 g**
Dietary Fiber	0 g
Total Sugars	0 g
Added Sugars included	0 g
**Protein**	**14 g**
Vitamin D	0 mcg
Calcium	6 mg
Iron	1 mg
Potassium	131 mg

Nutrients will display if the data is available

Please note: nutrient values are subject to change as data is updated

MyPlate Food Groups

Protein Foods 2 ounces

Visit MyPlate.gov

Overnight Oatmeal with Berries

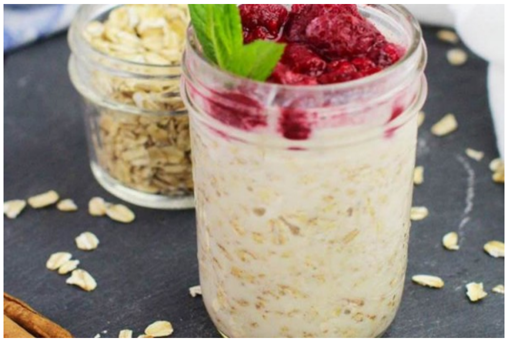

Makes: 1 Serving
Preparation Time: 15 minutes

A mixture of milk, yogurt, and raspberries combine with dry oats to become a smooth make-ahead breakfast. Just mix and refrigerate overnight for the next day.

Ingredients

- 1/2 cup low-fat milk (or less for thicker oatmeal)
- 1/4 cup Greek yogurt, fat-free
- 2 teaspoons honey
- 1/4 teaspoon cinnamon
- 1/4 teaspoon vanilla extract
- 1/2 cup uncooked rolled oats
- 1/4 cup raspberries, frozen

Directions

1. Combine milk, Greek yogurt, sugar, cinnamon, and vanilla extract in a container or jar with a lid.
2. Add oats and mix well.
3. Gently fold in raspberries.
4. Cover and refrigerate 8 hours to overnight.
5. Enjoy cold or heat as desired.

Source:

USDA Center for Nutrition Policy and Promotion

Nutrition Information

Serving Size: 1 serving

Nutrients	Amount
**Total Calories**	**311**
**Total Fat**	**4 g**
Saturated Fat	1 g
Cholesterol	7 mg
**Sodium**	**86 mg**
**Carbohydrates**	**53 g**
Dietary Fiber	9 g
Total Sugars	21 g
Added Sugars included	11 g
**Protein**	**17 g**
Vitamin D	1 mcg
Calcium	268 mg
Iron	2 mg
Potassium	513 mg

Nutrients will display if the data is available

Please note: nutrient values are subject to change as data is updated

MyPlate Food Groups

Fruits 1/4 cups
Grains 1 1/2 ounces
Dairy 3/4 cups

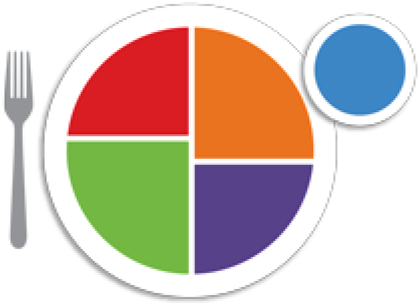

[Visit MyPlate.gov]

Quinoa and Black Bean Salad

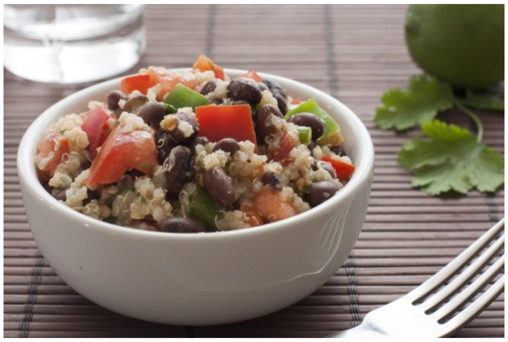

Makes: 6 servings

Quinoa (pronounced "KEEN-wah") is a whole grain with origins in South America. It is combined in this recipe with black beans, savory vegetables, and spices for a cold salad that is light and refreshing. This salad is high in protein and can be served as a main or side dish.

Ingredients

- 1/2 cup quinoa (dry)
- 1 1/2 cups water
- 1 1/2 tablespoons olive oil
- 3 teaspoons lime juice
- 1/4 teaspoon cumin
- 1/4 teaspoon coriander (ground, dried cilantro seeds)
- 2 tablespoons cilantro (chopped)
- 2 scallions (medium, minced)
- 1 can black beans, low-sodium (15.5 ounce can, rinsed and drained)
- 2 cups tomato (chopped)
- 1 red bell pepper (medium, chopped)
- 1 green bell pepper (medium, chopped)
- 2 green chiles (minced, to taste)
- black pepper (to taste)

Directions

1. Wash hands with soap and water.
2. Rinse the quinoa in cold water. Boil water in a saucepan, and then add the quinoa.
3. Return to boil, and then simmer until the water is absorbed, 10 to 15 minutes. Cool for 15 minutes.
4. While quinoa is cooking, mix olive oil, lime juice, cumin, coriander, chopped cilantro, and scallions in a small bowl, and set aside.
5. Combine chopped vegetables with the black beans in a large bowl, and set aside.
6. Once quinoa has cooled, combine all ingredients and mix well.
7. Cover and refrigerate until ready to serve.

Source:

Delicious Heart-Healthy Latino Recipes/ Platillos Latinos Sabrosos y Saludables

Nutrition Information

Serving Size: 1 cup (254g)

Nutrients	Amount
**Total Calories**	**173**
**Total Fat**	**5 g**
Saturated Fat	1 g
Cholesterol	0 mg
**Sodium**	**112 mg**
**Carbohydrates**	**27 g**
Dietary Fiber	8 g
Total Sugars	4 g
Added Sugars included	0 g
**Protein**	**7 g**
Vitamin D	0 mcg
Calcium	50 mg
Iron	3 mg
Potassium	554 mg

Nutrients will display if the data is available

Please note: nutrient values are subject to change as data is updated

MyPlate Food Groups

Vegetables	1 1/4 cups
Grains	1/2 ounces
Protein Foods	1 1/2 ounces

Visit MyPlate.gov

Stuffed Bell Peppers

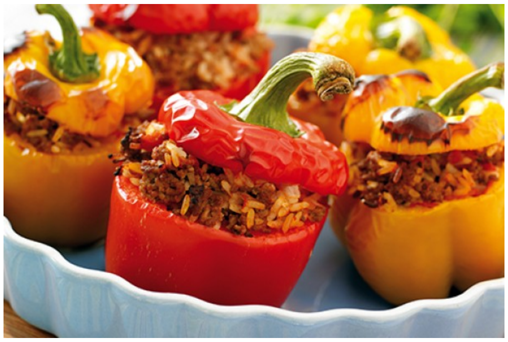

Makes: 5 Servings

Peppers stuffed with a combination of ground beef, brown rice, veggies, and spices create a filling dish. Try using red, green, yellow, and orange bell peppers for a colorful meal. Enjoy with a side of low-fat cottage cheese and canned pineapple.

Ingredients

- 5 bell peppers (red, orange, yellow, or green)
- 1 pound 90% lean ground beef
- 3/4 cup brown rice
- 1/2 can (14.5 ounces) low-sodium diced tomatoes
- 3 tablespoons lemon juice
- 1/4 teaspoon cinnamon
- 1/4 teaspoon allspice
- 1/4 teaspoon ground black pepper

Directions

1. Wash hands with soap and watr.
2. Cut a circular hole in the tops of the bell peppers. Remove seeds and membrane and set aside.
3. In a large bowl, combine remaining ingredients and stir until completely mixed. Fill the bell peppers with meat mixture.
4. Place the stuffed bell peppers in large stock pot on stove, with the tops facing up. Add 1-inch water to bottom of pot and cover.
5. Place heat on medium, keeping covered for 30 to 40 minutes until rice is done. Serve.

Source:

Simple Healthy Recipes
Oklahoma Nutrition Information and Education
ONIE Project

Nutrition Information

Serving Size: 1 stuffed pepper, 1/5 of recipe

Nutrients	Amount
**Total Calories**	**277**
**Total Fat**	**8 g**
Saturated Fat	3 g
Cholesterol	57 mg
**Sodium**	**66 mg**
**Carbohydrates**	**29 g**
Dietary Fiber	4 g
Total Sugars	4 g
Added Sugars included	0 g
**Protein**	**21 g**
Vitamin D	0 mcg
Calcium	48 mg
Iron	3 mg
Potassium	588 mg

Nutrients will display if the data is available

Please note: nutrient values are subject to change as data is updated

MyPlate Food Groups

Vegetables	1 1/4 cups
Grains	1 ounces
Protein Foods	2 1/2 ounces

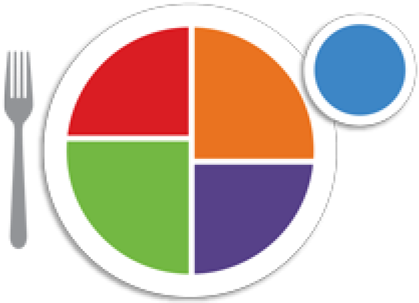

Visit MyPlate.gov

Julia’s Sautéed & Steamed Collards

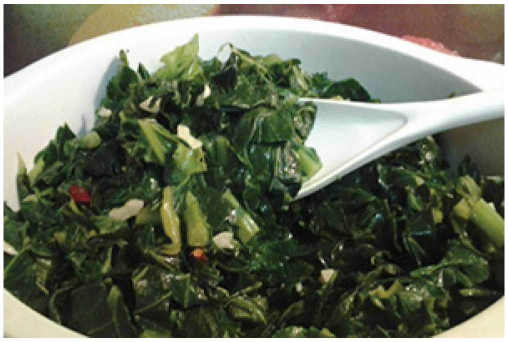

Makes: 8 Servings

Collards are an all-time favorite side to any meal. This recipe starts with tender collards and adds a hint of “heat” from hot pepper flakes. Try this easy and tasty recipe at your next family gathering.
- Julia, CNPP

Ingredients

- 16 cups chopped collards (small leaves)
- 2 tablespoons olive oil
- 4 large garlic cloves
- 1/2 teaspoon hot pepper flakes
- 1 teaspoon salt-free seasoning

Directions

1. Cut bottom stems from collards.
2. Wash and cut collards into bite-sized pieces. Place in colander to drain.
3. Chop garlic cloves into small pieces.
4. Place olive oil into large skillet. Place on high heat to start.
5. Place collards in skillet. Keep on high heat, turning collards as they saute, about 1 minute in the uncovered skillet. (You will not need to add water because of the water already on the washed collards.)
6. Add garlic cloves and red pepper flakes.
7. Place cover on the skillet to allow collards to steam until they are half their original volume.
8. Turn heat to low and continue steaming until the collards are tender.

Source:

USDA Center for Nutrition Policy and Promotion
Recipe submitted by Julia

Nutrition Information

Serving Size: 1 portion of Julia's Sauteed & Steamed Collards

Nutrients	Amount
**Total Calories**	**54**
**Total Fat**	**4 g**
Saturated Fat	1 g
Cholesterol	0 mg
**Sodium**	**15 mg**
**Carbohydrates**	**5 g**
Dietary Fiber	3 g
Total Sugars	0 g
Added Sugars included	0 g
**Protein**	**2 g**
Vitamin D	0 mcg
Calcium	111 mg
Iron	0 mg
Potassium	135 mg

Nutrients will display if the data is available

Please note: nutrient values are subject to change as data is updated

MyPlate Food Groups

Vegetables 1 cup

Visit MyPlate.gov

Healthy Black Bean Soup

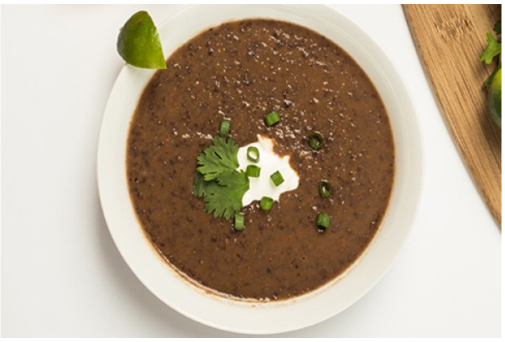

Makes: 4 Servings

This soup is full of flavor and is so easy to prepare. Using canned beans and salsa, along with cumin and cilantro, this soup is a great way to throw dinner together in minutes.

Ingredients

- 2 cans (15.5 ounces each) low-sodium black beans, drained and rinsed
- 2 cups low-sodium chicken broth (or low-sodium vegetable broth)
- 1 cup fresh salsa
- 1 teaspoon ground cumin
- 4 tablespoons low-fat sour cream (or fat-free)
- 2 tablespoons fresh cilantro, chopped

Directions

1. Wash hands with soap and water.
2. Lightly purée one can of black beans with broth, salsa, and cumin in a blender.
3. In a medium saucepan, combine puréed bean mixture with remaining beans and heat through.
4. To serve, ladle soup into 4 bowls.
5. Top each bowl with one tablespoon of sour cream and garnish with the cilantro.

Source:

Adapted from "Easy and Healthy Black Bean Soup"
Bronson Wellness Center
Bronson Healthcare - Michigan

Nutrition Information

Serving Size: 1 1/2 cups

Nutrients	Amount
**Total Calories**	**247**
**Total Fat**	**3 g**
Saturated Fat	1 g
Cholesterol	5 mg
**Sodium**	**390 mg**
**Carbohydrates**	**40 g**
Dietary Fiber	16 g
Total Sugars	1 g
Added Sugars included	0 g
**Protein**	**16 g**
Vitamin D	0 mcg
Calcium	111 mg
Iron	5 mg
Potassium	876 mg

Nutrients will display if the data is available

Please note: nutrient values are subject to change as data is updated

MyPlate Food Groups

Vegetables	1 1/2 cups
Protein Foods	5 ounces

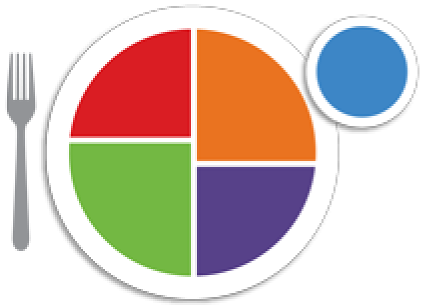

Visit MyPlate.gov

Sweet Potato Casserole

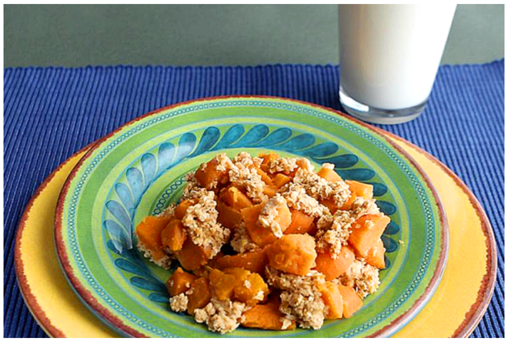

Makes: 6 Servings

Sweet potato casserole is rich is flavor and low in fat.

Ingredients

- 2 tablespoons 1% low fat milk
- 1 1/2 teaspoons brown sugar
- 1 teaspoon ground cinnamon
- 1/4 cup quick cooking oats, dry
- 2 3/4 cups low-sodium sweet potatoes, drained and chopped (about 15 ounces)

Directions

1. Preheat oven to 350 °F.
2. In a small bowl, combine milk, brown sugar, cinnamon, and oatmeal. Mix well and set aside.
3. In a medium-sized baking pan, add the sweet potatoes so that they cover the bottom of the pan.
4. Add the oatmeal mixture on top of the sweet potatoes.
5. Bake for 20 minutes. Serve hot or refrigerate and serve cold.

Source:

A Harvest of Recipes with USDA Foods
USDA Food Distribution Program on Indian Reservations

Nutrition Information

Serving Size: 1/4 cup

Nutrients	Amount
**Total Calories**	**94**
**Total Fat**	**1 g**
Saturated Fat	0 g
Cholesterol	0 mg
**Sodium**	**30 mg**
**Carbohydrates**	**21 g**
Dietary Fiber	3 g
Total Sugars	5 g
Added Sugars included	1 g
**Protein**	**2 g**
Vitamin D	0 mcg
Calcium	33 mg
Iron	1 mg
Potassium	309 mg

Nutrients will display if the data is available

Please note: nutrient values are subject to change as data is updated

MyPlate Food Groups

Vegetables 1/2 cups

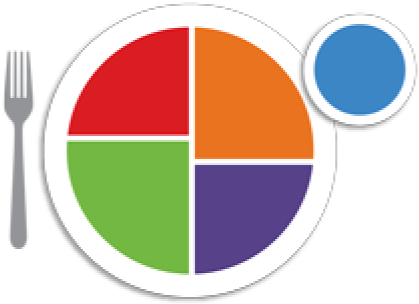

Visit MyPlate.gov

Bell Pepper and Apple Coleslaw

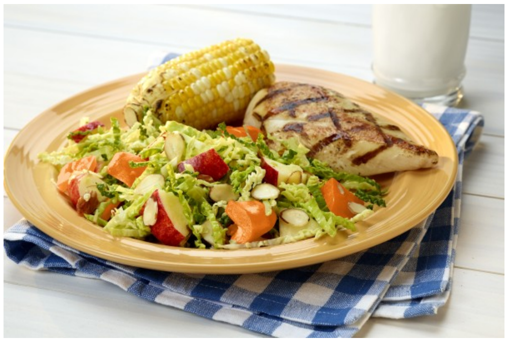

Makes: 4 Servings
Preparation Time: 20 minutes

Apples and bell peppers liven up this coleslaw made with an easy vinegar mustard dressing. Add more color with red, orange or yellow bell peppers. Use your favorite sweet and crisp apple, such as Gala or Fuji.

Ingredients

For the Dressing:

- 1 tablespoon vegetable oil
- 1/4 cup apple juice
- 2 tablespoons cider vinegar
- 2 teaspoons Dijon mustard
- 1/4 teaspoon salt
- 1 dash black pepper

For the Salad:

- 1 small head green or Napa cabbage
- 1 orange or red bell pepper
- 1 Gala or Fuji apple
- 1/4 cup sliced almonds

Directions

1. Whisk together dressing ingredients.
2. Shred or thinly slice cabbage.
3. Cut apple and pepper into small chunks.
4. Place cabbage, apples, pepper and almonds in large bowl.
5. Drizzle with dressing and toss.

Source:

Produce for Better Health Foundation

Nutrition Information

Nutrients	Amount
**Total Calories**	**154**
**Total Fat**	**9 g**
Saturated Fat	1 g
Cholesterol	0 mg
**Sodium**	**229 mg**
**Carbohydrates**	**17 g**
Dietary Fiber	5 g
Total Sugars	9 g
Added Sugars included	0 g
**Protein**	**4 g**
Vitamin D	0 mcg
Calcium	83 mg
Iron	1 mg
Potassium	386 mg

Nutrients will display if the data is available

Please note: nutrient values are subject to change as data is updated

MyPlate Food Groups

Fruits	1/2 cups
Vegetables	1 1/2 cups
Protein Foods	1/2 ounces

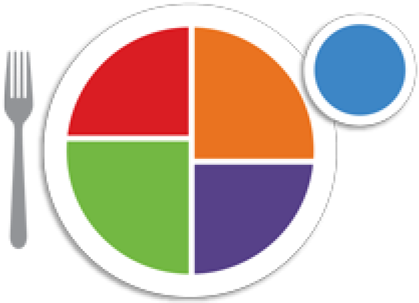

Visit MyPlate.gov

Apple & Chicken Salad

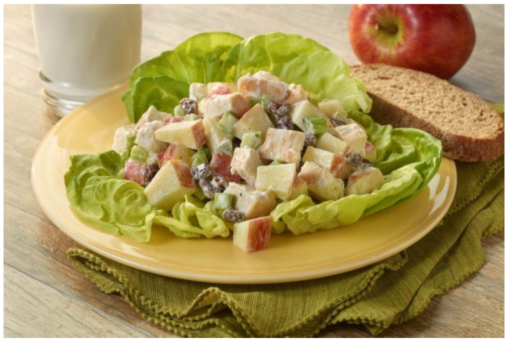

Makes: 4 Servings

A deliciously balanced blend of crisp Cameo apples, celery, raisins, chicken breast and Greek yogurt-based dressing, served over a bed of lettuce.

Ingredients

- 2 red apples (such as Cameo Apples)
- 2 celery stalks (diced)
- 2 chicken breasts (skinless, cooked, diced, about 2 cups)
- 1/4 cup plain non-fat Greek yogurt
- 1/2 cup raisins
- 1/4 cup mayonnaise
- 1/4 teaspoon salt
- 1/8 teaspoon ground black pepper
- 16 lettuce leaves (Bibb, Romaine, green or red leaf)

Directions

1. Cut apples in quarters; remove core and chop.
2. In a medium size bowl, mix all ingredients, except lettuce.
3. To serve, arrange lettuce on serving plates top with apple and chicken salad.

Source:

Produce for Better Health Foundation

Nutrition Information

Nutrients	Amount
**Total Calories**	**290**
**Total Fat**	**8 g**
Saturated Fat	2 g
Cholesterol	65 mg
**Sodium**	**330 mg**
**Carbohydrates**	**34 g**
Dietary Fiber	4 g
Total Sugars	12 g
Added Sugars included	0 g
**Protein**	**25 g**
Vitamin D	0 mcg
Calcium	56 mg
Iron	2 mg
Potassium	553 mg

Nutrients will display if the data is available

Please note: nutrient values are subject to change as data is updated

MyPlate Food Groups

Fruits	1 cup
Vegetables	1/2 cups
Protein Foods	2 1/2 ounces

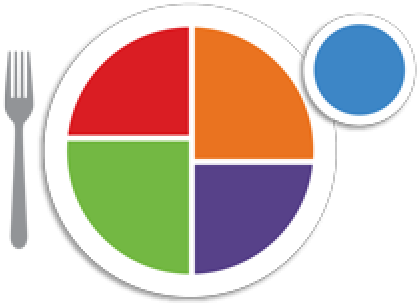

Visit MyPlate.gov

Slow Cooker Lentil Soup

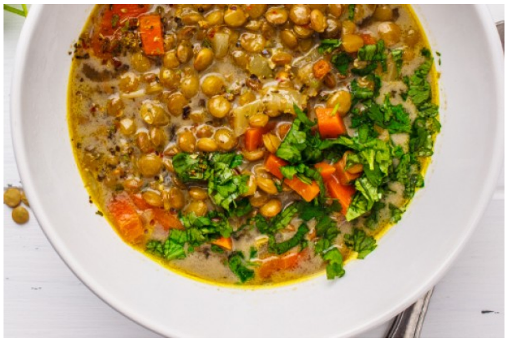

Makes: 6 servings

Quick to make but rich and delicious flavors of carrots, onion, and celery flavor in this slow cooker lentil soup. Add your favorite spices if you want to add a little kick.

Ingredients

- 6 cups water
- 1/4 cup fresh parsley, chopped (or 2 tablespoons dried)
- 2 teaspoons beef bouillon (or 2 cubes beef bouillon)
- 1 1/2 cups dry lentils
- 2 medium carrots, sliced
- 1 medium onion, chopped
- 2 medium celery stalks, sliced

Directions

1. Wash hands with soap and water.
2. Mix all ingredients together in slow cooker.
3. Cook on low for 8 to 10 hours or high for 4 to 5 hours.
4. Serve hot with crackers or bread.

Source:

Montana State University Extension Service
Nutrition Education Program

Nutrition Information

Serving Size: 1/6 of recipe

Nutrients	Amount
**Total Calories**	**177**
**Total Fat**	**1 g**
Saturated Fat	0 g
Cholesterol	0 mg
**Sodium**	**186 mg**
**Carbohydrates**	**32 g**
Dietary Fiber	12 g
Total Sugars	5 g
Added Sugars included	0 g
**Protein**	**13 g**
Vitamin D	0 mcg
Calcium	53 mg
Iron	5 mg
Potassium	642 mg

Nutrients will display if the data is available

Please note: nutrient values are subject to change as data is updated

MyPlate Food Groups

Vegetables	1 cups
Protein Foods	3 ounces

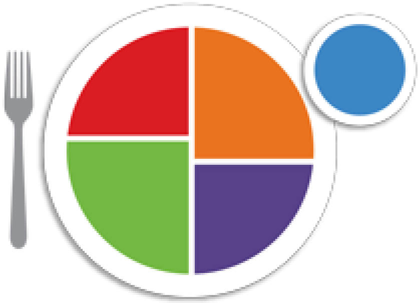

Visit MyPlate.gov

Cinnamon-Raisin Breakfast Mix

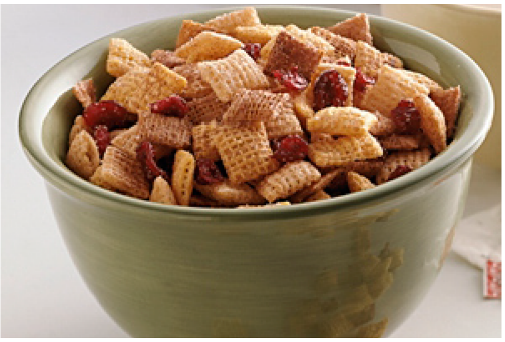

Makes: 10 Servings

Try this quick and easy breakfast mix, made in the microwave. Scoop portions into small bags and serve with yogurt and a banana for a nutritious, on-the-go breakfast!

Ingredients

- 1/4 cup sugar
- 1 teaspoon ground cinnamon
- 1/4 cup margarine, tub-style
- 1 1/2 cups square, whole-grain corn cereal
- 1 1/2 cups square, whole-grain rice cereal
- 1 1/2 cups square, whole-grain wheat cereal
- 1/2 cup raisins or dried cranberries

Directions

1. In small bowl, mix sugar and cinnamon; set aside.
2. In large microwavable bowl, microwave butter uncovered on HIGH about 40 seconds, or until melted.
3. Stir in cereals until evenly coated. Microwave uncovered 2 minutes, stirring after 1 minute.
4. Sprinkle half of the sugar mixture evenly over cereals; stir. Sprinkle with remaining sugar mixture; stir.
5. Microwave uncovered 1 minute.
6. Stir in raisins or dried cranberries. Spread on paper towels to cool.

Source:

MyPlate National Strategic Partners
The Grain Chain (members include American Bakers Association, Wheat Food Council, Grains Food Foundation, National Pasta Association, USA Rice Federation, General Mills, and Kellogg's)

Nutrition Information

Serving Size: one serving: 1/2 cup

Nutrients	Amount
**Total Calories**	**165**
**Total Fat**	**4 g**
Saturated Fat	1 g
Cholesterol	0 mg
**Sodium**	**210 mg**
**Carbohydrates**	**25 g**
Dietary Fiber	2 g
Total Sugars	11 g
Added Sugars included	6 g
**Protein**	**2 g**
Vitamin D	0 mcg
Calcium	55 milliliter
Iron	5 mg
Potassium	101 mg

Nutrients will display if the data is available

Please note: nutrient values are subject to change as data is updated

MyPlate Food Groups

Grains 1/2 ounces

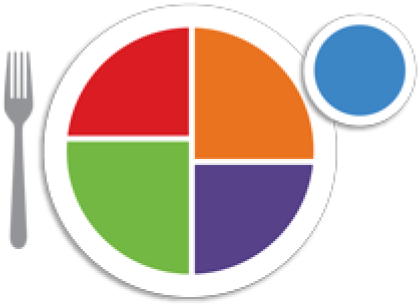

Visit MyPlate.gov
