# Supplementary material for: The Diet Guidelines: 3 Diets (DG3D) study protocol of a behavioral teaching kitchen intervention for type-2 diabetes prevention among African American adults
Source: Contemp Clin Trials. Author manuscript; Available in PMC 2026 Jun 18. (PMC13276729; doi:10.1016/j.cct.2025.108109)
Supplement: 3 [file NIHMS2184820-supplement-3.pdf]

USDA
United States Department of Agriculture

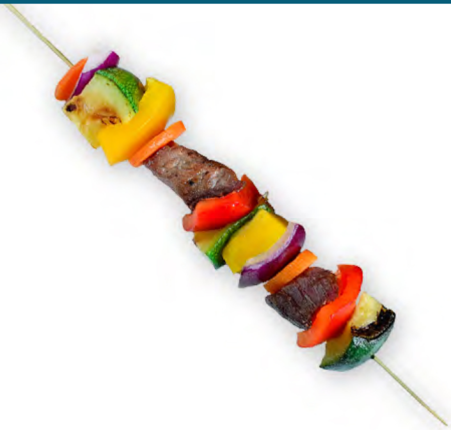

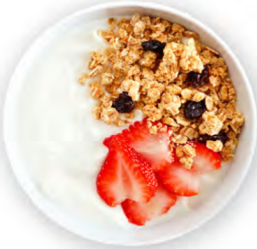

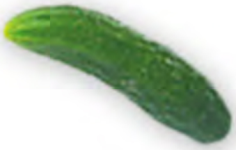

Start simple with MyPlate

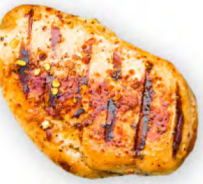

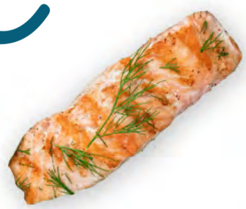

Mediterranean

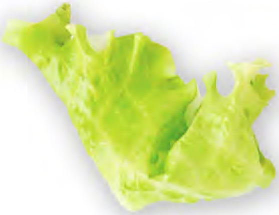

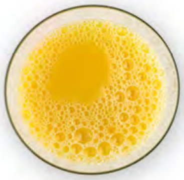

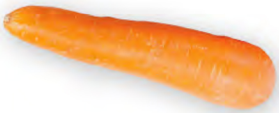

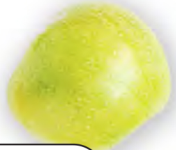

MyPlate.gov

Table of Contents

Veggie Omelet in a Mug 4
Greek Salad with Chicken 6
White Bean Bruschetta 9
Banana Pudding 11
Tomato and Cucumber Salad 13
Salmon Patties 15
Apple Cinnamon Bars 18
Easiest Banana Ice Cream 20
Avocado and Corn Salsa 22
Broccoli Alfredo 24
Very Berry Smoothie 26
Cornbread Dressing 28
Apple Cranberry Salad Toss 30
Overnight Oatmeal 32
Lentil Stew 34
Simple Green Smoothie 36
Yogurt Berry Parfait 38
Couscous with Peas and Onions 40
Minestrone Soup 42
Deviled Eggs 44
Oven-Baked Potato Pancakes 46
Falafel with Yogurt Sandwich 48
Gingery Quinoa with Green Beans 51
Smoky Mustard-Maple Salmon 53
Mixed Grain Bread 55
Hummus 57
Mediterranean Chicken and White Bean Salad 59
Broccoli-Cheddar Frittata 61
Grilled Asparagus and Shrimp Quinoa Salad with Lemon Vinaigrette 63
Grilled Vegetables 66
Angel Food Pastry with Fresh Berries and Whipped Cream 68
Tropical Yogurt Pops 70
Chocolate Chip Yogurt Cookies 72
Apple-Stuffed Squash 74
Rainbow Veggie Salad 76
Grilled Steak and Peppers Salad with Pears 78
Grilled Lamb Salad 81
Grilled Fruit 84
Grilled Vegetable Packets 86
Skinny Pizza 88
Rice-Crusted Pizza 90

Curried Chickpea Salad 93
Roasted Chickpeas (Garbanzo Beans) 95
Heavenly Deviled Eggs 97
Black Bean Quesadillas 99
Broccoli Potato Soup 101
Marinated Beef 103
Overnight Oatmeal with Berries 105
Quinoa and Black Bean Salad 107
Stuffed Bell Peppers 110
Julia’s Sautéed & Steamed Collards 112
Peanutty Stew 114
Bell Pepper and Apple Coleslaw 116
Fabulous Fig Bars 118
Slow Cooker Lentil Soup 120
Argentinean Grilled Steak with Salsa Criolla 122

Veggie Omelet in a Mug

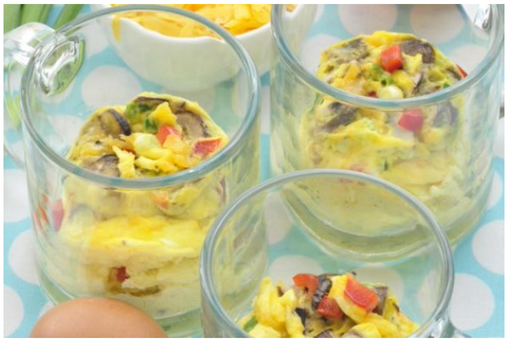

Makes: 1 Serving
Preparation Time: 10 minutes
Cook Time: 3 minutes

Making a veggie omelet has never been so easy! Combine all of your ingredients in a mug, pop it in the microwave, and breakfast is ready!

Ingredients

- 2 eggs
- 2 tablespoons fat-free (skim) milk
- 1 pinch salt
- 1 pinch black pepper
- 1/4 cup finely chopped mushrooms (or your favorite vegetables)
- 2 tablespoons low-fat cheddar cheese, shredded (or your favorite cheese)

Directions

1. Wash hands with soap and water.
2. Lightly grease the inside of a 12-ounce microwave-safe mug.
3. Use a fork to combine the eggs, milk, salt and pepper in the mug and stir well. Mix in the vegetables and cheese.
4. Microwave on HIGH for 45 seconds. Stir. Return to the microwave and cook on HIGH until the mixture has puffed and set, 60 to 90 seconds. The omelet may look wet on the top but it will dry as it cools.
5. Refrigerate leftovers within 2 hours.

Source:

Food Hero
Oregon State University Cooperative Extension Service

Nutrition Information

Serving Size: 1 recipe

Nutrients	Amount
**Total Calories**	**180**
**Total Fat**	**11 g**
Saturated Fat	4 g
Cholesterol	336 mg
**Sodium**	**397 mg**
**Carbohydrates**	**3 g**
Dietary Fiber	0 g
Total Sugars	2 g
Added Sugars included	0 g
**Protein**	**17 g**
Vitamin D	2 mcg
Calcium	216 mg
Iron	2 mg
Potassium	239 mg

Nutrients will display if the data is available

Please note: nutrient values are subject to change as data is updated

MyPlate Food Groups

Vegetables	1/4 cups
Protein Foods	2 ounces
Dairy	1/2 cups

Visit MyPlate.gov

Greek Salad with Chicken

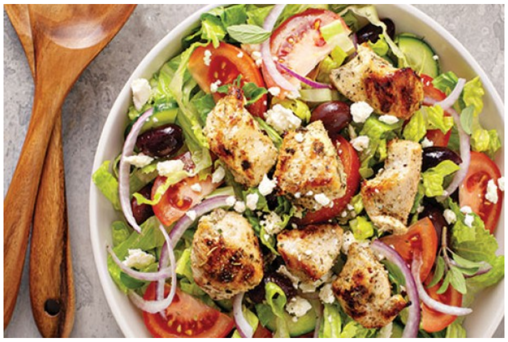

Makes: 4 Servings

A colorful salad loaded with lettuce, cucumbers, tomatoes, onions, and olives topped with chicken and a creamy yogurt dressing.

Ingredients

For the Dressing:

- 1/2 cup Greek yogurt, fat-free
- 2 teaspoons lemon juice
- 2 teaspoons olive oil
- 1 clove garlic
- 1 teaspoon oregano
- 1/4 teaspoon salt
- 1/4 teaspoon pepper

For the Salad:

- 1 head romaine lettuce
- 1/2 cup cherry tomatoes, cut in half
- 1 cucumber, diced
- 1/4 cup red onion, sliced
- 1/4 cup black olives, cut in half
- 1 cup cooked chicken breast, cubed
- 1/4 cup feta cheese, crumbled

Directions

1. To make the dressing, add Greek yogurt, lemon juice, olive oil, garlic, oregano, salt, and pepper to a small bowl. Mix well to combine.
2. Cover dressing and refrigerate for at least 1 hour.
3. Wash lettuce and tear into bite-sized pieces. Place in large salad bowl and add tomatoes, cucumbers, red onion, olives, and chicken.
4. Sprinkle with feta cheese.
5. Top with yogurt dressing and enjoy.

Source:

USDA Center for Nutrition Policy and Promotion

Nutrition Information

Nutrients	Amount
**Total Calories**	**173**
**Total Fat**	**7 g**
Saturated Fat	2 g
Cholesterol	42 mg
**Sodium**	**522 mg**
**Carbohydrates**	**12 g**
Dietary Fiber	6 g
Total Sugars	5 g
Added Sugars included	0 g
**Protein**	**18 g**
Vitamin D	0 mcg
Calcium	208 mg
Iron	2 mg
Potassium	836 mg

Nutrients will display if the data is available

Please note: nutrient values are subject to change as data is updated

MyPlate Food Groups

Vegetables	2 1/4 cups
Protein Foods	1 ounces
Dairy	1/4 cups

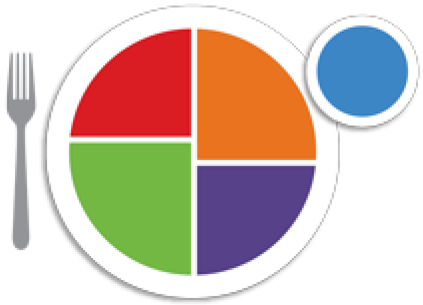

Visit MyPlate.gov

White Bean Bruschetta

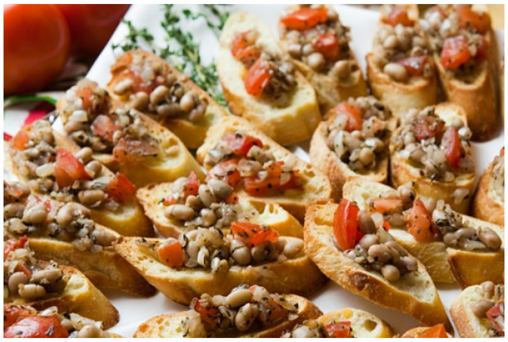

Makes: 4 Servings

Try this tasty bruschetta with crispy bread topped with white beans, tomatoes, and spices. Served as a side dish or an appetizer, it is sure to be a crowd pleaser!

Ingredients

- 1 whole-wheat French baguette, cut into 12 thin slices along the bias
- 1/4 cup olive oil, divided
- 1 cup white onion, chopped
- 4 cloves garlic, minced
- 1 teaspoon dried basil
- 1 teaspoon dried oregano
- 1 cup canned navy beans, rinsed and drained
- 2 tomatoes, cored and cubed
- 2 tablespoons balsamic vinegar

Directions

1. Slice the baguette on the diagonal into thin slices (about 12 slices for a baguette).
2. In a large sauté pan, heat 2 tablespoons of the olive oil over medium heat.
3. Place the bread slices in the pan and cook on medium high heat until sizzling and golden. Before flipping the bread, add an additional tablespoon of olive oil to the pan and cook the second side until golden.
4. For the topping, cook the onions and the remaining tablespoon of olive oil over medium heat until the onions are soft, about 7 minutes.
5. Add the garlic, basil, and oregano and cook another minute or two, until fragrant.
6. Add beans and continue cooking for another five minutes on low heat. Add the tomatoes and turn off the heat, allowing tomatoes to warm without cooking.
7. Drizzle the balsamic vinegar into the pan and gently stir.
8. Scoop heaping spoonsful of the tomato-bean mixture onto the grilled bread and enjoy.

Source:

MyPlate National Strategic Partners
The Grain Chain

Nutrition Information

Nutrients	Amount
**Total Calories**	**342**
**Total Fat**	**15 g**
Saturated Fat	2 g
Cholesterol	0 mg
**Sodium**	**332 mg**
**Carbohydrates**	**43 g**
Dietary Fiber	7 g
Total Sugars	6 g
Added Sugars included	1 g
**Protein**	**10 g**
Vitamin D	0 mcg
Calcium	95 mg
Iron	4 mg
Potassium	574 mg

Nutrients will display if the data is available

Please note: nutrient values are subject to change as data is updated

MyPlate Food Groups

Vegetables	3/4 cups
Grains	1 1/2 ounces

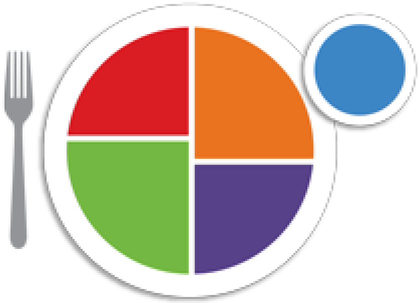

Visit MyPlate.gov

Banana Pudding

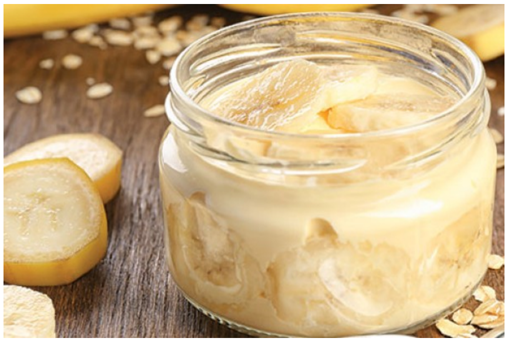

Makes: 8 servings

Dress up instant pudding mix with this dessert that combines low fat dairy and fruit for a tasty, nourishing treat.

Ingredients

- 1 (3.4 ounce) box instant pudding mix, banana or vanilla
- 2 cups fat-free (skim) milk (or 1% milk)
- 1 (8 ounce) container of fat-free plain yogurt (or non-dairy whipped topping)
- 2 medium bananas, sliced (or other sliced fruit)

Directions

1. Wash hands with soap and water.
2. In a medium bowl, combine pudding and milk. Beat with wooden spoon, wire whisk, or electric mixer on lowest speed for 2 minutes
3. Gently mix yogurt (or whipped topping) with pudding mixture. Refrigerate for 30 minutes.
4. Layer fruit slices in the bottom of 8 dessert cups.
5. Pour the pudding mixture over sliced fruit. Top with more fruit.
6. Refrigerate until ready to serve, at least 5 minutes. The taste is better if it's refrigerated longer.

Source:

Choices: Steps Toward Health, Adapted from: Lisa's Famous Dessert
University of Massachusetts
Extension Nutrition Education Program

Nutrition Information

Serving Size: 1/8 of recipe

Nutrients	Amount
**Total Calories**	**110**
**Total Fat**	**1 g**
Saturated Fat	0 g
Cholesterol	3 mg
**Sodium**	**219 mg**
**Carbohydrates**	**23 g**
Dietary Fiber	1 g
Total Sugars	20 g
Added Sugars included	11 g
**Protein**	**4 g**
Vitamin D	1 mcg
Calcium	130 mg
Iron	0 mg
Potassium	269 mg

Nutrients will display if the data is available

Please note: nutrient values are subject to change as data is updated

MyPlate Food Groups

Fruits 1/4 cups

Dairy 1/4 cups

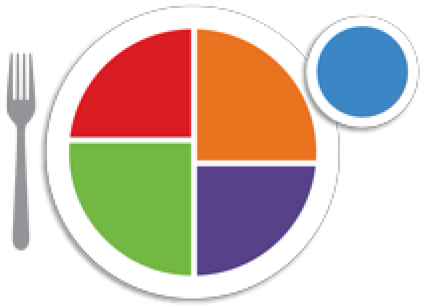

Visit MyPlate.gov

Tomato and Cucumber Salad

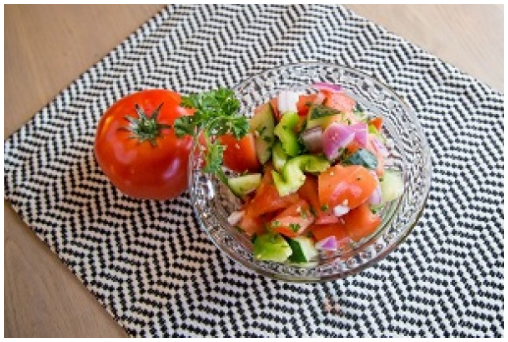

Makes: 12 Servings

This refreshing salad is an excellent source of vitamin C and a great way to take advantage of the vegetables' summer growing season.

Ingredients

Salad

- 4 large tomatoes (cubed)
- 1 large cucumber (chopped)
- 1 cup red onion (chopped)
- 1 cup green pepper (chopped)
- 1/3 cup parsley (chopped)

Dressing

- 1/3 cup apple cider vinegar
- 1 tablespoon olive oil (or oil of your choice)
- 2 cloves garlic (minced)
- 1/2 teaspoon salt
- 1/2 teaspoon ground black pepper
- 1/2 teaspoon sugar

Directions

1. Wash hands with soap and water.
2. In a large bowl, combine the salad ingredients.
3. In a small bowl, mix the dressing ingredients together.
4. Pour the dressing over the salad. Mix well.
5. Refrigerate for at least 1 hour before serving.

Source:

University of Nebraska at Lincoln
Nebraska Nutrition Education Program

Nutrition Information

Serving Size: 3/4 cup

Nutrients	Amount
**Total Calories**	**34**
**Total Fat**	**1 g**
Saturated Fat	0 g
Cholesterol	0 mg
**Sodium**	**103 mg**
**Carbohydrates**	**5 g**
Dietary Fiber	1 g
Total Sugars	3 g
Added Sugars included	0 g
**Protein**	**1 g**
Vitamin D	0 mcg
Calcium	17 mg
Iron	0 mg
Potassium	225 mg

Nutrients will display if the data is available

Please note: nutrient values are subject to change as data is updated

MyPlate Food Groups

Vegetables 3/4 cups

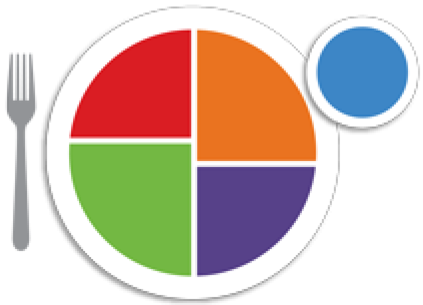

[Visit MyPlate.gov]

Salmon Patties

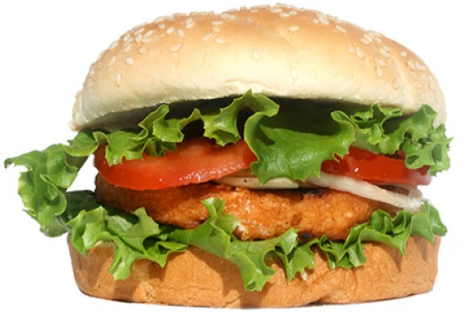

Makes: 6 servings

Tasty patties cooked in a skillet that are perfect topped with fresh lettuce, tomato, and a side of fresh or canned pineapple.

Ingredients

- 1 can salmon, 16 oz
- 1 tablespoon lemon juice
- cold water
- minced celery (1/4 cup, optional)
- green pepper (1 tablespoon, optional)
- 1/2 medium onion
- 2 large eggs
- 1/3 cup bread crumbs or cracker crumbs
- 2 tablespoons all-purpose flour
- 1/8 teaspoon black pepper
- 1 tablespoon vegetable oil

Directions

1. Wash hands with soap and water.
2. Wash fresh vegetables (if using).
3. Collect, mince, and measure all ingredients before starting to prepare the recipe.
4. Open salmon, and drain liquid into a liquid measuring cup. Add lemon juice and cold water to the salmon liquid to make 1/2 cup liquid total, and set aside.
5. Put the salmon in a separate mixing bowl. Mix in the celery (if using), green pepper (if using), and onion.
6. In another small bowl, beat the eggs. Then, add them to the salmon mixture.
7. Add the bread or cracker crumbs, flour, pepper, and the salmon liquid mixture to the salmon mixture, and stir until all ingredients are mixed together.
8. Use 1/3 cup measuring cup to measure salmon mixture. Shape into a 1/2 inch thick patty, and place on a plate. Repeat to make 6 patties.
9. Heat the oil in a skillet over medium heat, then add 3 patties.
10. Cook for about 2 to 3 minutes (or until golden brown) on each side.
11. Remove patties from skillet, and place on a clean paper towel-lined plate to drain. Cook remaining 3 patties, then serve immediately. Refrigerate leftovers within 2 hours. Eat within 3 to 5 days.

Source:

Eat for Health Toolkit
Recipe by Missouri Nutrition Network
Optional additions from ONIE Project & Colorado State University Extension

Nutrition Information

Serving Size: 1 patty

Nutrients	Amount
**Total Calories**	**165**
**Total Fat**	**8 g**
Saturated Fat	1 g
Cholesterol	98 mg
**Sodium**	**316 mg**
**Carbohydrates**	**7 g**
Dietary Fiber	1 g
Total Sugars	1 g
Added Sugars included	0 g
**Protein**	**16 g**
Vitamin D	10 mcg
Calcium	159 mg
Iron	1 mg
Potassium	261 mg

Nutrients will display if the data is available

Please note: nutrient values are subject to change as data is updated

MyPlate Food Groups

Grains	1/2 ounces
Protein Foods	2 1/2 ounces

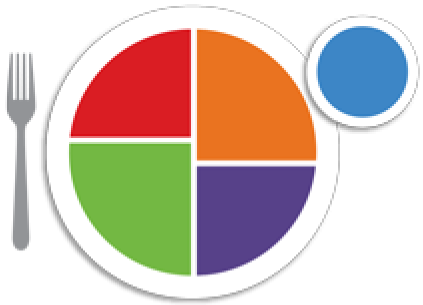

Visit MyPlate.gov

Apple Cinnamon Bars

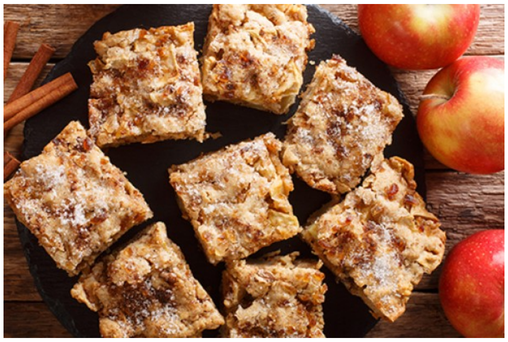

Makes: 24 servings

Apples are sandwiched between two cinnamon crumb layers for a delicious treat.

Ingredients

- 4 medium apples
- 1 cup flour
- 1/4 teaspoon salt
- 1/2 teaspoon baking soda
- 1/2 teaspoon cinnamon
- 1/2 cup brown sugar, packed
- 1 cup oats, uncooked
- 1/2 cup margarine, 1 stick (or butter)
- 4 sprays of cooking spray

Directions

1. Wash hands with soap and water.
2. Preheat the oven to 350 degrees F.
3. Put the flour, salt, baking soda, cinnamon, brown sugar, and oats in the mixing bowl. Stir together.
4. Add the margarine to the bowl. Use the 2 table knives to mix the ingredients and cut them into crumbs.
5. Lightly grease the bottom and sides of the baking dish with cooking spray.
6. Spread half of the crumb mixture in the greased baking dish.
7. Remove the core from the apples and slice them. Put the apple slices into the baking dish.
8. Top the apples with the rest of the crumb mixture.
9. Bake in the oven for 40-45 minutes.
10. Cut into squares. It will fall apart easily.

Source:

Pennsylvania Nutrition Education Network (original)
Recipe modification adapted from SNAP-Ed New York, October 2023

Nutrition Information

Serving Size: 1 bar, 1/24 of recipe

Nutrients	Amount
**Total Calories**	**82**
**Total Fat**	**4 g**
Saturated Fat	1 g
Cholesterol	0 mg
**Sodium**	**53 mg**
**Carbohydrates**	**12 g**
Dietary Fiber	1 g
Total Sugars	7 g
Added Sugars included	4 g
**Protein**	**1 g**
Vitamin D	0 mcg
Calcium	7 mg
Iron	0 mg
Potassium	37 mg

Nutrients will display if the data is available

Please note: nutrient values are subject to change as data is updated

MyPlate Food Groups

Fruits 1/4 cups

Grains 1/2 ounces

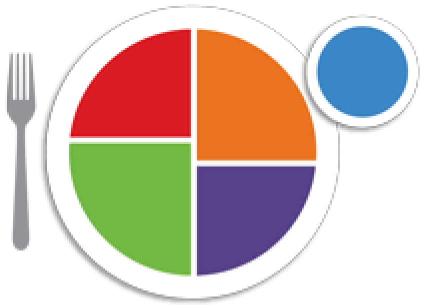

Visit MyPlate.gov

Easiest Banana Ice Cream

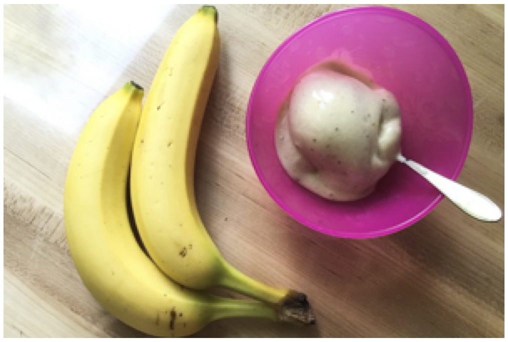

Makes: 3 Servings

What should you do with over-ripe bananas? Don't toss them! Freeze them and then throw them in the blender to make this delicious and creamy frozen dessert.

Ingredients

- 2 1/2 bananas (2-3 bananas)
- 3 tablespoons 1% milk
- chocolate syrup (optional)

Directions

1. Wash hands with soap and water.
2. When bananas have become very ripe, peel and slice them into medium size round pieces. Place on a plastic wrap covered baking sheet and put in freezer overnight.
3. Place frozen bananas in a food processor or blender with a small splash of milk (not over 1/4 cup total).
4. Pulse food processor or blender until bananas begin to break up. They will be tiny balls of bananas at this point.
5. Using a spoon or rubber spatula, scrape down the banana mixture. Continue running the food processor until the mixture is smooth and creamy. This may take a few minutes. The ice cream will look and tasted like soft serve ice cream when finished.
6. Serve in an ice cream cone or in a small bowl with a small ribbon of chocolate syrup on top (optional).

Source:

Alabama Cooperative Extension System

Nutrition Information

Serving Size: 1/3 of recipe

Nutrients	Amount
**Total Calories**	**94**
**Total Fat**	**0 g**
Saturated Fat	0 g
Cholesterol	1 mg
**Sodium**	**8 mg**
**Carbohydrates**	**23 g**
Dietary Fiber	3 g
Total Sugars	13 g
Added Sugars included	0 g
**Protein**	**2 g**
Vitamin D	0 mcg
Calcium	24 mg
Iron	0 mg
Potassium	375 mg

Nutrients will display if the data is available

Please note: nutrient values are subject to change as data is updated

MyPlate Food Groups

Fruits 3/4 cups

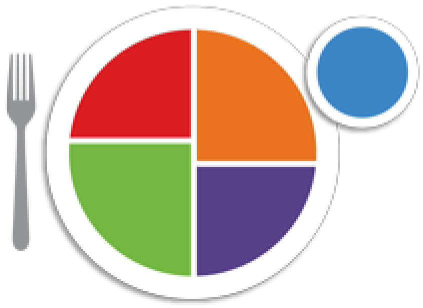

[Visit MyPlate.gov](https://www.myplate.gov)

Avocado and Corn Salsa

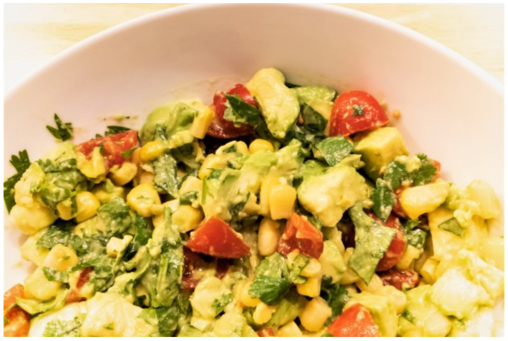

Makes: 5 Servings

Avocado and corn are given a flavor boost by fresh cilantro and lime. Serve this salsa with our Baked Chicken for an easy weekday meal.

Ingredients

- 1 avocado, diced
- 3/4 cup frozen corn kernels, thawed
- 1/2 cup grape tomatoes, quartered
- 1 tablespoon fresh cilantro, chopped
- 2 teaspoons lime juice
- 1/4 teaspoon salt

Directions

1. Wash hands with soap and water.
2. Toss avocado, corn, tomatoes, cilantro, lime juice, and salt in a medium bowl.
3. Chill one hour and then serve.

Source:

Bronson Wellness Center
Bronson Healthcare - Michigan

Nutrition Information

Serving Size: 1/2 cup

Nutrients	Amount
**Total Calories**	**67**
**Total Fat**	**4 g**
Saturated Fat	1 g
Cholesterol	0 mg
**Sodium**	**119 mg**
**Carbohydrates**	**8 g**
Dietary Fiber	3 g
Total Sugars	1 g
Added Sugars included	0 g
**Protein**	**1 g**
Vitamin D	0 mcg
Calcium	6 mg
Iron	0 mg
Potassium	235 mg

Nutrients will display if the data is available

Please note: nutrient values are subject to change as data is updated

MyPlate Food Groups

Vegetables 1/2 cups

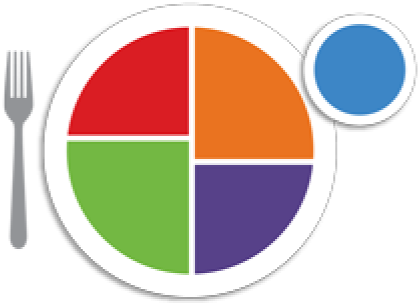

Visit MyPlate.gov

Broccoli Alfredo

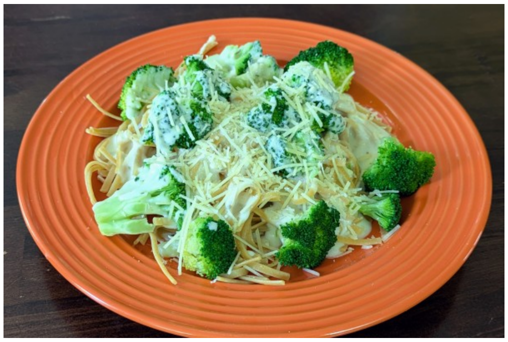

Makes: 4 Servings

This dish features whole wheat pasta and fat-free Parmesan cheese.

Ingredients

- 4 cups broccoli, cooked
- 4 cups whole wheat pasta, cooked
- 2 1/8 cups 1% milk, divided (1/8 cup or 2 tablespoons is used later in the recipe)
- 1 teaspoon dried basil
- 1/2 teaspoon garlic powder
- 1 cup Parmesan cheese, reduced fat
- 2 tablespoons cornstarch
- black pepper (to taste, optional)

Directions

1. Wash hands with soap and water.
2. Placed cooked broccoli and pasta in a bowl.
3. Heat milk over medium heat and then add basil and garlic powder. When hot, add Parmesan cheese.
4. Mix cornstarch with 2 or 3 tablespoons of milk and add to hot mixture. Heat until thickened.
5. Pour mixture over pasta and broccoli. Serve.

Source:

Simple Healthy Recipes
Oklahoma Nutrition Information and Education
ONIE Project

Nutrition Information

Serving Size: 1/4 of recipe

Nutrients	Amount
**Total Calories**	**349**
**Total Fat**	**6 g**
Saturated Fat	4 g
Cholesterol	24 mg
**Sodium**	**388 mg**
**Carbohydrates**	**58 g**
Dietary Fiber	12 g
Total Sugars	11 g
Added Sugars included	0 g
**Protein**	**22 g**
Vitamin D	2 mcg
Calcium	491 mg
Iron	3 mg
Potassium	590 mg

Nutrients will display if the data is available

Please note: nutrient values are subject to change as data is updated

MyPlate Food Groups

Vegetables	1 cup
Grains	2 ounces
Dairy	1 1/4 cups

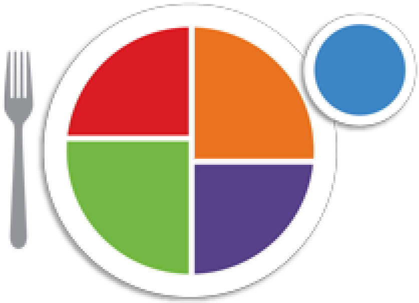

Visit MyPlate.gov

Very Berry Smoothie

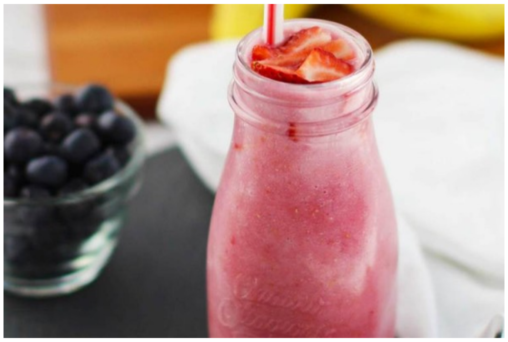

Makes: 2 Servings

This refreshing pineapple, banana, and berry blend smoothie has 2 and 1/2 cups of fruit per serving.

Ingredients

- 1 cup frozen strawberries
- 1 cup frozen raspberries
- 1 cup pineapple chunks
- 1 banana
- 1 cup skim milk (or almond milk)
- 1 1/2 cups ice

Directions

1. Peel and slice the banana.
2. Gather all ingredients and combine in a blender.
3. Cover and blend until smooth.
4. Serve right away or pour into ice cube trays and freeze. During the week, pop out the cubes for a quick on-the-go breakfast.

Source:

USDA Center for Nutrition Policy and Promotion

Nutrition Information

Nutrients	Amount
**Total Calories**	**252**
**Total Fat**	**1 g**
Saturated Fat	0 g
Cholesterol	2 mg
**Sodium**	**56 mg**
**Carbohydrates**	**59 g**
Dietary Fiber	13 g
Total Sugars	37 g
Added Sugars included	0 g
**Protein**	**7 g**
Vitamin D	1 mcg
Calcium	N/A
Iron	N/A
Potassium	N/A

Nutrients will display if the data is available

Please note: nutrient values are subject to change as data is updated

MyPlate Food Groups

Fruits 2 1/2 cups
Dairy 1/2 cups

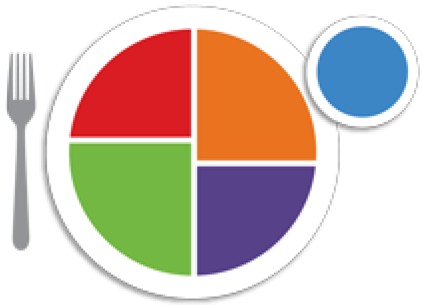

Visit MyPlate.gov

Cornbread Dressing

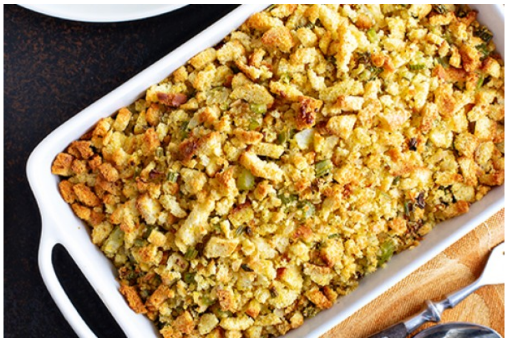

Makes: 8 Servings

Some families call this "dressing" while others call it "stuffing." Whatever your family prefers, there's no denying that cornbread, broth, and savory herbs come together beautifully in this traditional dish.

Ingredients

- 1 teaspoon olive oil (or cooking oil of choice)
- 4 green onions, thinly sliced
- 1 celery stalk
- 1 1/2 cups chicken broth, low-sodium
- 1 teaspoon dried sage
- 1/4 teaspoon black pepper
- 4 cups crumbled cornbread
- 1/2 cup egg substitute
- 1/4 cup chopped pecans

Directions

1. Wash hands with soap and water.
2. Preheat the oven to 350 °F. Lightly spray an 8-inch square baking pan with vegetable oil spray.
3. In a large skillet, heat the oil over medium heat, swirling to coat the bottom. Cook the green onions and celery for 4 to 5 minutes, or until tender, stirring occasionally. Stir in the broth, sage, and pepper.
4. Put the cornbread in a large bowl. Stir in the vegetable mixture, egg substitute, and pecans (the mixture will be moist). Transfer to the baking pan.
5. Bake for 30 minutes, or until cooked through and golden brown on top. Transfer to a serving bowl.

Source:

Power to End Stroke: 46 Healthy Soul Food Recipes Cookbook
American Heart Association

Nutrition Information

Serving Size: 1 serving, 1/8 of recipe

Nutrients	Amount
**Total Calories**	**172**
**Total Fat**	**8 g**
Saturated Fat	1 g
Cholesterol	18 mg
**Sodium**	**266 mg**
**Carbohydrates**	**21 g**
Dietary Fiber	1 g
Total Sugars	4 g
Added Sugars included	2 g
**Protein**	**6 g**
Vitamin D	1 mcg
Calcium	113 mg
Iron	2 mg
Potassium	179 mg

Nutrients will display if the data is available

Please note: nutrient values are subject to change as data is updated

MyPlate Food Groups

Grains	1 ounces
Protein Foods	1/2 ounces

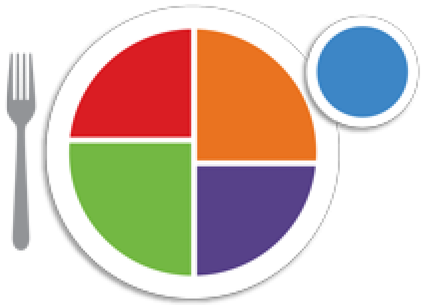

Visit MyPlate.gov

Apple Cranberry Salad Toss

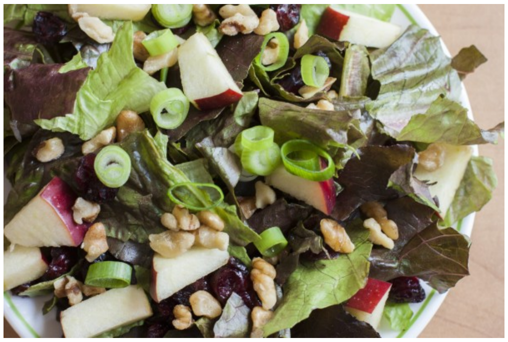

Makes: 8 servings

Enjoy the best of fall flavors with this sweet and tart green salad. Light yet crisp, it’s a perfect dish for the autumnal change of weather.

Ingredients

- 1 medium head of lettuce (about 10 cups)
- 2 medium apples, sliced
- 1/2 cup chopped walnuts
- 1 cup dried cranberries
- 1/2 cup green onions, sliced
- 3/4 cup vinaigrette dressing

Directions

1. Wash hands with soap and water.
2. Toss lettuce, apples, walnuts, cranberries, and onions in a large bowl.
3. Add dressing; toss to coat. Serve immediately.

Source:

Creative Recipes for Less Familiar USDA Commodities Used by Household Programs
USDA, Food and Nutrition Service, Food Distribution Service

Nutrition Information

Serving Size: 1/8 of recipe

Nutrients	Amount
**Total Calories**	**174**
**Total Fat**	**10 g**
Saturated Fat	1 g
Cholesterol	0 mg
**Sodium**	**227 mg**
**Carbohydrates**	**22 g**
Dietary Fiber	3 g
Total Sugars	17 g
Added Sugars included	9 g
**Protein**	**2 g**
Vitamin D	0 mcg
Calcium	30 mg
Iron	1 mg
Potassium	206 mg

Nutrients will display if the data is available

Please note: nutrient values are subject to change as data is updated

MyPlate Food Groups

Fruits	1/2 cups
Vegetables	3/4 cups
Protein Foods	1/2 ounces

Visit MyPlate.gov

Overnight Oatmeal

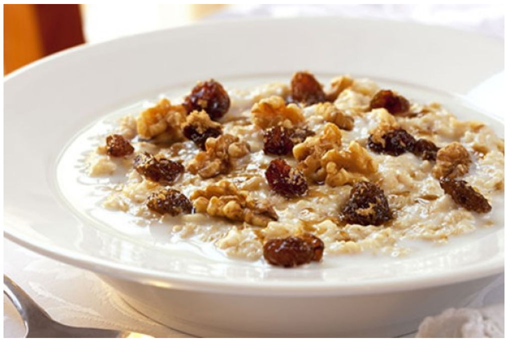

Makes: 2 Servings

Overnight soaking cuts down on prep time in the morning, so you can enjoy a healthy and hearty, cooked breakfast, even after hitting the snooze button.

Ingredients

- 1 cup dry oats
- 2 1/2 cups water
- 1/2 cup dried fruit (raisins, cranberries, or apricots)
- 1/4 cup walnuts, lightly chopped (or pecans)

Directions

1. Wash hands with soap and water.
2. Put all ingredients in a cooking pot and cover. Let sit overnight in refrigerator.
3. In the morning, put on a stove burner and cook on medium heat until simmering, then turn on low and heat for 10 to 15 minutes more or less depending on type of oats -- quick (5 minutes), regular, or steel cut.
4. Serve warm with a little milk (or milk alternative), a drizzle of maple syrup, and a side of fresh fruit, if desired.

Source:

Lake Family Resource Center
Be-Fresh Program

Nutrition Information

Serving Size: 1/2 of recipe

Nutrients	Amount
**Total Calories**	**333**
**Total Fat**	**13 g**
Saturated Fat	2 g
Cholesterol	0 mg
**Sodium**	**27 mg**
**Carbohydrates**	**50 g**
Dietary Fiber	7 g
Total Sugars	17 g
Added Sugars included	0 g
**Protein**	**8 g**
Vitamin D	0 mcg
Calcium	57 mg
Iron	3 mg
Potassium	470 mg

Nutrients will display if the data is available

Please note: nutrient values are subject to change as data is updated

MyPlate Food Groups

Fruits	1/2 cups
Grains	2 ounces
Protein Foods	1 ounces

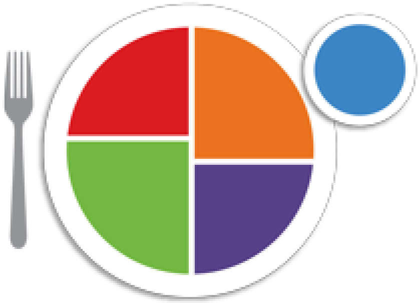

Visit MyPlate.gov

Lentil Stew

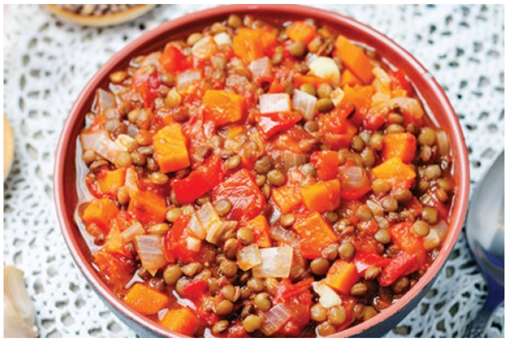

Makes: 10 servings

Lentils, carrots, and tomatoes are a savory and satisfying combination in this delicious and easy to prepare stew. Enjoy with a side of your favorite citrus fruit.

Ingredients

- 2 teaspoons olive oil (or canola oil)
- 1 onion (large, chopped)
- 1 teaspoon garlic powder
- 1 1/2 10-ounce packages of frozen sliced carrots
- 1 cup dry lentils (rinsed and drained)
- 3 cans diced tomatoes, low-sodium (14.5 ounces each)
- 3 cups water
- 1 teaspoon chili powder

Directions

1. Wash hands with soap and water.
2. Heat the oil in a large pot over medium heat.
3. Add chopped onion.
4. Cook for 3 minutes, or until tender.
5. Stir in garlic powder, carrots, lentils, tomatoes, water, and chili powder.
6. Simmer, uncovered, for about 20 minutes or until lentils are tender.

Source:

2009 Recipe Calendar
University of Maryland Extension
Food Supplement Nutrition Education Program

Nutrition Information

Serving Size: 1 cup

Nutrients	Amount
**Total Calories**	**113**
**Total Fat**	**2 g**
Saturated Fat	0 g
Cholesterol	0 mg
**Sodium**	**52 mg**
**Carbohydrates**	**20 g**
Dietary Fiber	7 g
Total Sugars	6 g
Added Sugars included	0 g
**Protein**	**6 g**
Vitamin D	0 mcg
Calcium	69 mg
Iron	3 mg
Potassium	536 mg

Nutrients will display if the data is available

Please note: nutrient values are subject to change as data is updated

MyPlate Food Groups

Vegetables	1 1/4 cups
Protein Foods	1 ounces

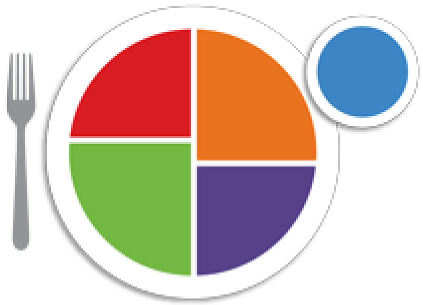

Visit MyPlate.gov

Simple Green Smoothie

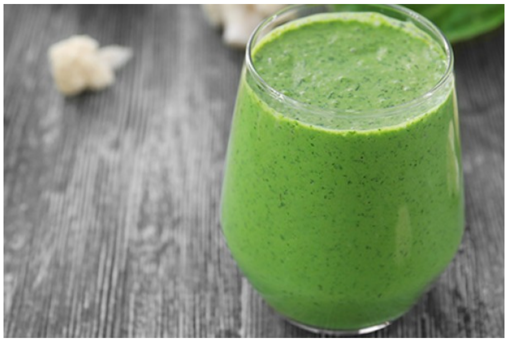

Makes: 2 Servings

Add kale or spinach to this fruit smoothie for a quick and delicious breakfast or snack.

Ingredients

- 1 cup kale or spinach
- 1 medium banana
- 1 cup low-fat milk (or fortified soy, coconut, or almond milk)
- 1 cup plain low-fat yogurt
- 1 medium apple (cored and sliced)
- 1 cup frozen fruit (all one fruit or a combination of mixed frozen fruit)
- flax seeds, 1 tablespoon (optional)
- chia seeds, 1 tablespoon (optional)

Directions

1. Wash hands with soap and water.
2. Add the kale or spinach and the liquid of your choice to a blender. Blend.
3. Add in the rest of the ingredients, blending after each item.
4. Serve and enjoy, cold.
5. Reserve the leftover smoothie in the refrigerator for later in the day or the next day.

Source:

Northern Valley Catholic Social Service

Nutrition Information

Nutrients	Amount
**Total Calories**	**299**
**Total Fat**	**4 g**
Saturated Fat	2 g
Cholesterol	N/A
**Sodium**	**156 mg**
**Carbohydrates**	**56 g**
Dietary Fiber	7 g
Total Sugars	38 g
Added Sugars included	0 g
**Protein**	**13 g**
Vitamin D	1 mcg
Calcium	454 mg
Iron	2 mg
Potassium	1119 mg

Nutrients will display if the data is available

Please note: nutrient values are subject to change as data is updated

MyPlate Food Groups

Fruits	2 cups
Vegetables	1/4 cups
Dairy	1 cup

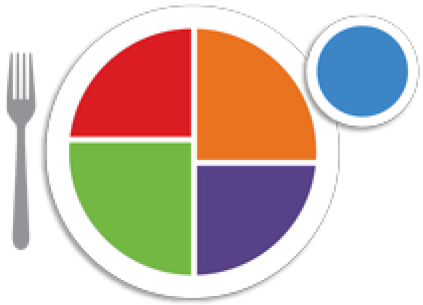

Visit MyPlate.gov

Yogurt Berry Parfait

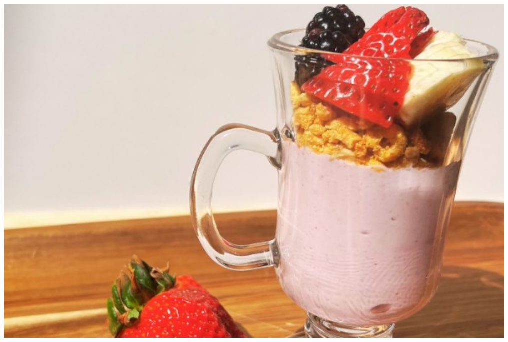

Makes: 4 servings
Preparation Time: 5 minutes

Enjoy this hearty snack or dessert, abounding with fresh fruit, granola, and any flavor of low-fat yogurt.

Ingredients

- 2 cups low fat yogurt (or fat-free, any flavor*)
- 1 cup banana (sliced)
- 1/2 cup blueberries (fresh)
- 1/2 cup strawberries (fresh, sliced)
- other optional fruit (raspberries, peaches, pineapple and/or mangos)
- 1 cup granola

Directions

1. Wash hands with soap and water.
2. Line up 4 parfait or other tall glasses.
3. Spoon 1/2 cup of yogurt into each glass.
4. Sprinkle with granola.
5. Top with about 1/4 cup of fruit.

Source:

USDA Food and Nutrition Information Center

Nutrition Information

Serving Size: 1 parfait, 1/4 of recipe (214g)

Nutrients	Amount
**Total Calories**	**304**
**Total Fat**	**9 g**
Saturated Fat	2 g
Cholesterol	6 mg
**Sodium**	**89 mg**
**Carbohydrates**	**46 g**
Dietary Fiber	5 g
Total Sugars	30 g
Added Sugars included	11 g
**Protein**	**11 g**
Vitamin D	1 mcg
Calcium	240 mg
Iron	2 mg
Potassium	613 mg

Nutrients will display if the data is available

Please note: nutrient values are subject to change as data is updated

MyPlate Food Groups

Fruits	1/2 cups
Grains	1/2 ounces
Protein Foods	1/2 ounces
Dairy	1/2 cups

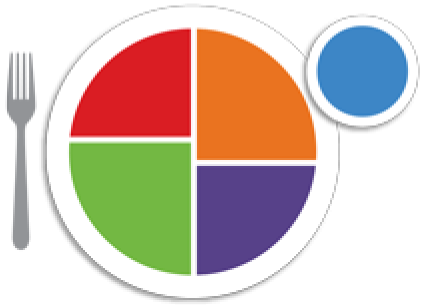

Visit MyPlate.gov

Couscous with Peas and Onions

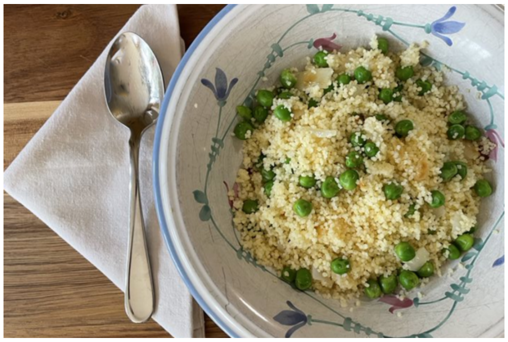

Makes: 4 servings

Couscous is cooked with sage, peas, and onions for a savory side dish. Look for couscous or whole grain couscous in the aisle near the rice.

Ingredients

- 1 teaspoon olive oil
- 1 cup onion, finely chopped
- 1 cup frozen green peas
- 1/2 teaspoon ground sage
- 1 1/3 cups water
- 1 cup couscous
- salt (1/2 teaspoon, optional)

Directions

1. Wash hands with soap and water.
2. Combine oil and onions in heavy skillet.
3. Sauté for 5 to 10 minutes until lightly browned.
4. Add the peas, sage, water, couscous, and salt if desired.
5. Cover and cook on medium heat for about 5 minutes or until peas are tender but still bright green and all of the water is absorbed.
6. Fluff with fork.

Source:

CHOICES: Steps Toward Health
UMass Extension Nutrition Education Program

Nutrition Information

Serving Size: 1 cup

Nutrients	Amount
**Total Calories**	**205**
**Total Fat**	**1 g**
Saturated Fat	0 g
Cholesterol	0 mg
**Sodium**	**40 mg**
**Carbohydrates**	**40 g**
Dietary Fiber	5 g
Total Sugars	4 g
Added Sugars included	0 g
**Protein**	**8 g**
Vitamin D	0 mcg
Calcium	33 mg
Iron	1 mg
Potassium	179 mg

Nutrients will display if the data is available

Please note: nutrient values are subject to change as data is updated

MyPlate Food Groups

Vegetables	1/2 cups
Grains	1 1/2 ounces

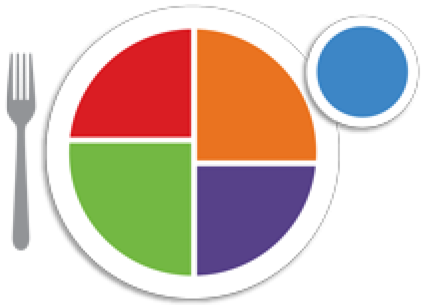

[Visit MyPlate.gov]

Minestrone Soup

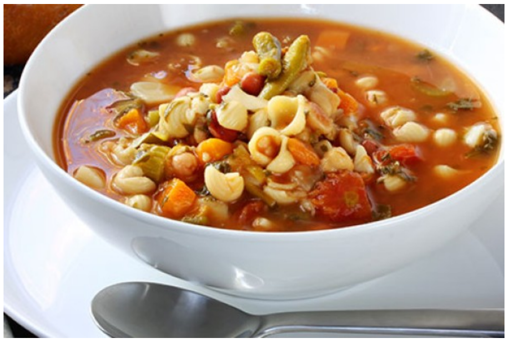

Makes: 6 servings

Prepare delicious soup in a snap! Canned beans and frozen veggies make this dish perfect for busy weeknights.

Ingredients

- 1 package (10 ounces) frozen vegetables, any type
- 2 cans (14.5 ounces each) low-sodium stewed tomatoes
- 2 cans (14.5 ounces each) low-sodium broth, any flavor
- 1 can (15.5 ounces) low-sodium beans, any type
- 1 cup dry pasta, any type

Directions

This recipe is developed for a child to help an adult in the kitchen. Directions are written to different audiences.

1. **Adult and child**: Wash hands with soap and water.
2. **Child**: In a large pot, combine frozen vegetables, tomatoes, broth and beans.
3. **Adult**: Bring the soup to a boil and add the pasta. Then reduce to low heat. Let simmer for 6 to 8 minutes or until the pasta and vegetables are tender.

Source:

Food Wise Learn at Home Print Materials
Rutgers Cooperative Extension

Nutrition Information

Serving Size: 1 cup, 1/6 of recipe

Nutrients	Amount
**Total Calories**	**144**
**Total Fat**	**1 g**
Saturated Fat	0 g
Cholesterol	0 mg
**Sodium**	**172 mg**
**Carbohydrates**	**29 g**
Dietary Fiber	9 g
Total Sugars	6 g
Added Sugars included	1 g
**Protein**	**7 g**
Vitamin D	N/A
Calcium	80 mg
Iron	3 mg
Potassium	163 mg

Nutrients will display if the data is available

Please note: nutrient values are subject to change as data is updated

MyPlate Food Groups

Vegetables	1 1/4 cups
Protein Foods	1 1/2 ounces

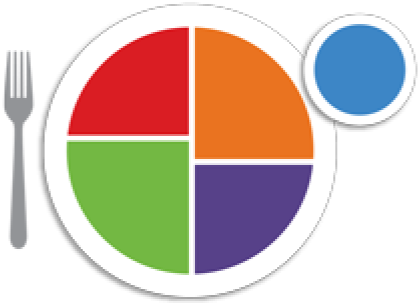

Visit MyPlate.gov

Deviled Eggs

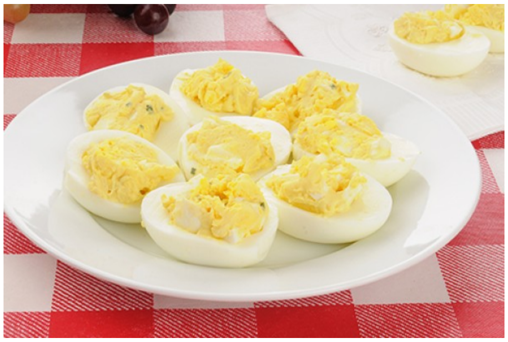

Makes: 6 servings

A favorite dish at parties and potlucks, Deviled Eggs also make a great, protein-filled anytime snack!

Ingredients

- 6 large eggs, hard-boiled and peeled
- 1/4 cup mayonnaise
- 1/8 teaspoon salt
- 1/8 teaspoon black pepper

Directions

1. Wash hands with soap and water.
2. Hard boil eggs by placing eggs in a saucepan and covering them with water. Bring to a boil.
3. Reduce heat to simmer; cook for 15 minutes.
4. Immediately rinse under cold water to stop cooking and to make it easy to peel off shells. Refrigerate peeled eggs (without shells) until ready for use.
5. Slice eggs into halves lengthwise. Remove yellow yolks and save whites.
6. Place yolks in a one-quart zip lock style bag along with the remaining ingredients (except the egg whites). Press out air.
7. Close bag and knead (mush together) until ingredients are well-blended. (Note: you could also put yolks in a bowl with other ingredients [except the egg whites] and mix together well until they look like a paste).
8. Push contents toward one corner of the bag. Cut about 1/2 inch off the corner of the bag. Squeezing the bag gently, fill reserved egg white hollows with the yolk mixture. (Note if you used a bowl, spoon the yolk mixture into the egg whites).
9. Chill to blend flavors.

Source:

Kids a Cookin'
Kansas Family Nutrition Program

Nutrition Information

Serving Size: 2 prepared egg halves, 1/6 of recipe

Nutrients	Amount
**Total Calories**	**134**
**Total Fat**	**12 g**
Saturated Fat	3 g
Cholesterol	190 mg
**Sodium**	**180 mg**
**Carbohydrates**	**0 g**
Dietary Fiber	0 g
Total Sugars	0 g
Added Sugars included	0 g
**Protein**	**6 g**
Vitamin D	1 mcg
Calcium	29 mg
Iron	1 mg
Potassium	72 mg

Nutrients will display if the data is available

Please note: nutrient values are subject to change as data is updated

MyPlate Food Groups

Protein Foods 1 ounces

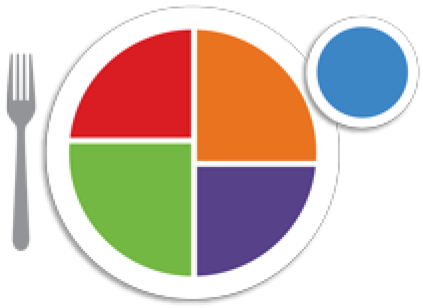

Visit MyPlate.gov

Oven-Baked Potato Pancakes

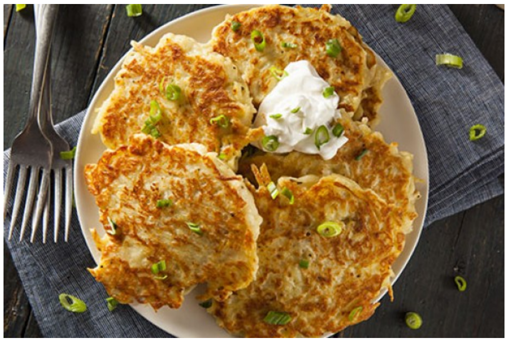

Makes: 12 Servings

These potato pancakes are easily baked in the oven and offer a healthier version of a traditional Hanukkah recipe.

Ingredients

- 2 tablespoons olive oil (or cooking oil of your choice)
- 3 tablespoons whole wheat flour (or all-purpose flour)
- 1 teaspoon baking powder
- 3/4 teaspoon salt
- 2 large russet potatoes (or Yukon Gold potatoes)
- 1 onion, small peeled
- 1 large egg
- applesauce and yogurt (plain low-fat or Greek) (optional)

Directions

1. Wash hands with soap and water.
2. Preheat the oven to 425 °F. Lightly oil the baking sheets by spreading the oil around with your clean hand or a paper towel.
3. Mix the flour, baking powder, and salt in a small bowl.
4. Use the large holes on your grater to grate the potato, then grate the onion too.
5. Put the potato and onion mixture in the colander inside the sink. Using a paper towel, press the potato mixture down to squeeze out and blot up some of the extra moisture. Stir it and blot again.
6. Put the potato mixture in the large bowl, add the egg, and stir well. Add the flour mixture and stir it very well.
7. Use a spoon or your fingers to pluck a clump of the potato mixture from the bowl and spread it into a round, flat nest on the oiled baking sheet: it should make a circle that's about 3 inches wide and 1/4 inch thick. Repeat to fill the sheet. (You don't need to leave space between them.)
8. Bake until the bottoms are deeply golden, 15 to 20 minutes, then turn the pancakes over and put them back in the over for 10 more minutes.
9. Serve the latkes with applesauce and yogurt (optional).

Source:

ChopChop Family

Nutrition Information

Serving Size: 1 latke, 1/12 of recipe (76g)

Nutrients	Amount
**Total Calories**	**83**
**Total Fat**	**3 g**
Saturated Fat	0 g
Cholesterol	16 mg
**Sodium**	**196 mg**
**Carbohydrates**	**13 g**
Dietary Fiber	2 g
Total Sugars	1 g
Added Sugars included	0 g
**Protein**	**2 g**
Vitamin D	0 mcg
Calcium	35 mg
Iron	1 mg
Potassium	285 mg

Nutrients will display if the data is available

Please note: nutrient values are subject to change as data is updated

MyPlate Food Groups

Vegetables 1/2 cups

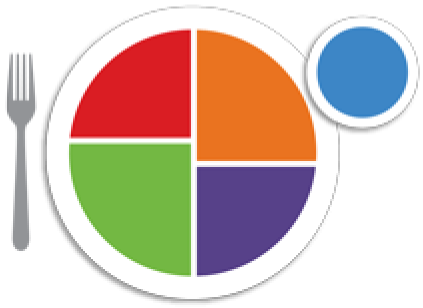

Visit MyPlate.gov

Falafel with Yogurt Sandwich

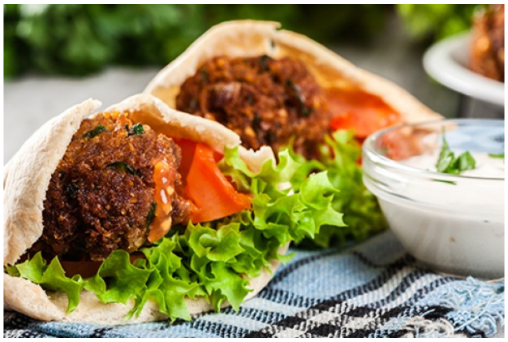

Makes: 4 servings

Falafel is a pan-fried patty made from ground garbanzo beans (chickpeas) and Middle Eastern spices. It makes a healthy and delicious sandwich when served with a seasoned yogurt dressing and fresh onions on whole wheat pita bread. Try this recipe for lunch or dinner.

Ingredients

- 1 cup dry garbanzo beans/chickpeas (sorted and rinsed)
- 3 cups water
- 1/4 cup oil
- 1 garlic clove (crushed)
- 1 medium onion (chopped)
- 1/3 tablespoon parsley (1 sprig or about 1 teaspoon, chopped)
- 1/4 teaspoon salt
- 2 teaspoons lemon juice
- 1/3 teaspoon hot pepper sauce
- 1 cup plain yogurt, low-fat
- 1 medium onion (chopped)
- 4 whole wheat pita bread (pockets)
- 1/4 cup flour (as needed)
- tomatoes (sliced, optional)
- lettuce (optional)
- bread crumbs (optional - see note)

Directions

1. Wash hands with soap and water.
2. Put beans and water in large pot and soak by the overnight or quick-soak method.
3. Cook until tender, about 2 hours. Add more water if necessary. Drain.
4. Slowly heat oil and sauté garlic and 1 onion until tender (5 to 7 minutes).
5. Mash cooked beans, sautéed vegetables, parsley, salt, lemon juice and hot pepper sauce until smooth.
6. With floured hands, form ovals with bean mixture (about 1/4 cup each). Roll in flour.
7. Fry falafel, with the remaining oil in skillet, until golden brown. Drain on paper towel.
8. Combine yogurt with remaining onion.
9. Serve falafel in pocket bread topped with yogurt.

Source:

The Bold and Beautiful Book of Bean Recipes
Washington State WIC Program

Nutrition Information

Serving Size: 1 sandwich (480g)

Nutrients	Amount
**Total Calories**	**503**
**Total Fat**	**19 g**
Saturated Fat	3 g
Cholesterol	4 mg
**Sodium**	**408 mg**
**Carbohydrates**	**69 g**
Dietary Fiber	12 g
Total Sugars	12 g
Added Sugars included	0 g
**Protein**	**18 g**
Vitamin D	0 mcg
Calcium	192 mg
Iron	5 mg
Potassium	622 mg

Nutrients will display if the data is available

Please note: nutrient values are subject to change as data is updated

MyPlate Food Groups

Vegetables	1 cups
Grains	2 1/2 ounces
Protein Foods	2 1/2 ounces
Dairy	1/4 cups

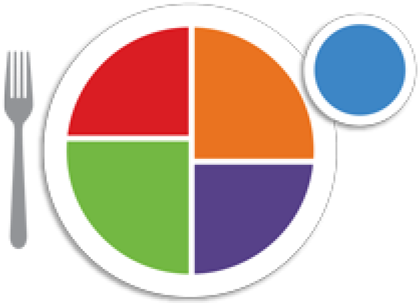

Visit MyPlate.gov

Gingery Quinoa with Green Beans

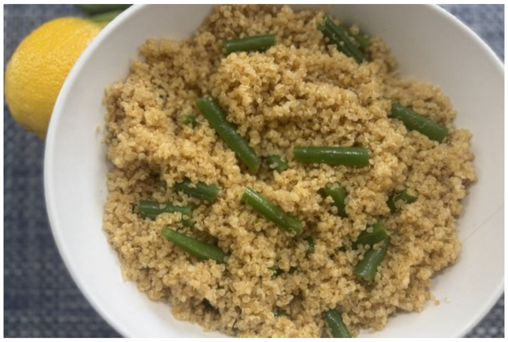

Makes: 4 Servings

Fresh green beans work well when they’re in season. If they’re not, try frozen in this recipe that combines a whole grains and veggies into one satisfying and eye-catching dish.

Ingredients

- 1 cup quinoa (uncooked)
- 1 1/2 cups water or low-sodium broth
- 2 cups green beans (trimmed into 1" pieces)
- 1 tablespoon olive oil or cooking oil
- 1 tablespoon sesame oil
- 2 tablespoons reduced sodium soy sauce
- 2 1/2 tablespoons lemon juice
- 1/4 teaspoon ground ginger

Directions

1. Wash hands with soap and water.
2. Toast quinoa in a dry skillet over medium heat, stirring constantly for about 3 minutes.
3. Transfer toasted quinoa to a medium pot and add water or broth.
4. Bring to a boil, then let simmer until all of the water is absorbed and quinoa is tender (10 to 15 minutes).
5. Set aside and leave uncovered to cool.
6. While quinoa is cooling, place green beans in a microwave-safe dish. Add a small amount of water, cover, and microwave for 4-6 minutes or until just tender.
7. Rinse green beans in cold water to stop the cooking process.
8. In a large bowl, combine the quinoa, green beans, olive oil or cooking oil, sesame oil, soy sauce, lemon juice, and ground ginger. Stir well.
9. Season with salt and pepper, as needed.
10. Serve warm or at room temperature.

Source:

Bronson Wellness Center
Bronson Healthcare - Michigan

Nutrition Information

Serving Size: 1 Cup

Nutrients	Amount
**Total Calories**	**239**
**Total Fat**	**9 g**
Saturated Fat	1 g
Cholesterol	0 mg
**Sodium**	**301 mg**
**Carbohydrates**	**33 g**
Dietary Fiber	6 g
Total Sugars	2 g
Added Sugars included	0 g
**Protein**	**7 g**
Vitamin D	0 mcg
Calcium	56 mg
Iron	3 mg
Potassium	365 mg

Nutrients will display if the data is available

Please note: nutrient values are subject to change as data is updated

MyPlate Food Groups

Vegetables	1/2 cups
Grains	1 1/2 ounces

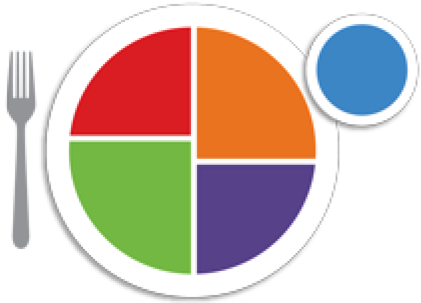

Visit MyPlate.gov

Smoky Mustard-Maple Salmon

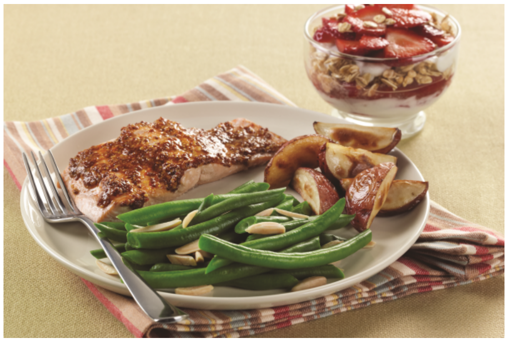

Makes: 4 Servings

It doesn’t get much easier -- or more delicious -- than this ultra-fast salmon. The sweetness of the maple balances the tangy mustard; smoked paprika or ground chipotle adds another layer of flavor. When you buy your salmon, ask at the fish counter to have the salmon cut into four 4-ounce fillets with the skin removed.

Ingredients

- 3 tablespoons whole-grain or Dijon mustard
- 1 tablespoon pure maple syrup
- 1/4 teaspoon smoked paprika or ground chipotle pepper
- 1/4 teaspoon freshly ground pepper
- 1/8 teaspoon salt
- 16 ounces salmon (skinless, about 4 ounces per fillet)

Directions

1. Preheat oven to 450 °F.
2. Line a baking sheet with foil and coat with cooking spray.
3. Combine mustard, maple syrup, paprika (or chipotle), pepper, and salt in a small bowl.
4. Place salmon fillets on the prepared baking sheet.
5. Spread the mustard mixture evenly on the salmon.
6. Roast until just cooked through (approximately 8 to 12 minutes).

Source:

EatingWell Magazine

Nutrition Information

Serving Size: 1 serving

Nutrients	Amount
**Total Calories**	**148**
**Total Fat**	**4 g**
Saturated Fat	1 g
Cholesterol	53 mg
**Sodium**	**276 mg**
**Carbohydrates**	**4 g**
Dietary Fiber	0 g
Total Sugars	3 g
Added Sugars included	3 g
**Protein**	**23 g**
Vitamin D	13 mcg
Calcium	18 mg
Iron	1 mg
Potassium	434 mg

Nutrients will display if the data is available

Please note: nutrient values are subject to change as data is updated

MyPlate Food Groups

Protein Foods 3 1/2 ounces

Visit MyPlate.gov

Mixed Grain Bread

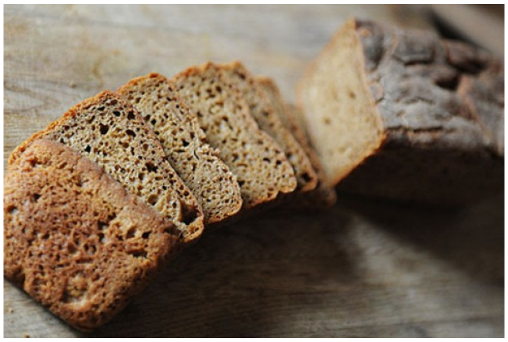

Makes: 20 servings

Using cornmeal, rye, and whole wheat flour gives this chewy and delicious bread a wonderful texture and flavor.

Ingredients

- 1/4 cup yellow cornmeal
- 1/4 cup brown sugar, packed
- 1 teaspoon salt
- 2 tablespoons vegetable oil
- 1/4 cup boiling water
- 1 packet active dry yeast
- 1/4 cup warm water, 105 - 115 degrees
- 1/3 cup whole wheat flour
- 1/4 cup rye flour
- 2 1/4 cups all-purpose flour

Directions

1. Wash hands with soap and water.
2. Mix cornmeal, brown sugar, salt and oil with boiling water, cool to lukewarm (105 to 115 degrees F).
3. Dissolve yeast in 1/4 cup warm water; stir into cornmeal mixture. Add whole wheat and rye flours and mix well. Stir in enough all purpose flour to make dough stiff enough to knead.
4. Turn dough onto lightly floured surface. Knead until smooth and elastic, about 5 to 10 minutes.
5. Place dough in lightly oiled bowl, turning oil top. Cover with clean towel; let rise in warm place until double, about 1 hour.
6. Punch dough down; turn onto clean surface. Cover with clean towel; let rest 10 minutes. Shape dough and place in greased 9x5-inch pan. Cover with clean towel; let rise until almost double, about 1 hour.
7. Preheat oven to 375 degrees F. Bake 35 to 45 minutes or until bread sounds hollow when tapped. Cover with aluminum foil during baking if bread is browning too quickly. Remove bread from pan and cool on wire rack.

Source:

Cooking Up Fun - Yeast Breads
Cornell University Cooperative Extension
Division of Nutritional Sciences

Nutrition Information

Serving Size: 1 slice, 1/20 of recipe

Nutrients	Amount
**Total Calories**	**92**
**Total Fat**	**2 g**
Saturated Fat	0 g
Cholesterol	0 mg
**Sodium**	**118 mg**
**Carbohydrates**	**17 g**
Dietary Fiber	1 g
Total Sugars	3 g
Added Sugars included	3 g
**Protein**	**2 g**
Vitamin D	0 mcg
Calcium	6 mg
Iron	1 mg
Potassium	38 mg

Nutrients will display if the data is available

Please note: nutrient values are subject to change as data is updated

MyPlate Food Groups

Grains 1 ounces

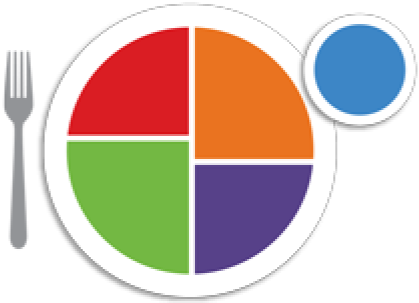

Visit MyPlate.gov

Hummus

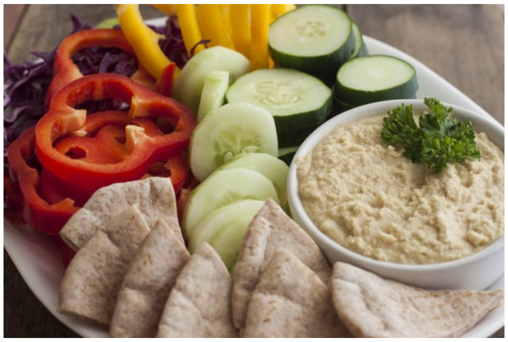

Makes: 8 Servings
Preparation Time: 20 minutes

This classic garbanzo bean dip is easy to make and a versatile dish. Serve hummus with fresh raw vegetables, on a piece of whole wheat pita bread or tortilla, on crackers, or as a sandwich filling.

Ingredients

- 2 cups garbanzo beans, cooked (also known as chickpeas)
- 2 cloves garlic (minced)
- 1/4 cup lemon juice
- 1 tablespoon sesame tahini [sesame paste] (or substitute peanut butter for a sweet taste)
- 2 tablespoons olive oil

Directions

1. Wash hands with soap and water.
2. Mash the garbanzo beans until smooth (if you have a blender, put the beans and lemon juice into it and blend).
3. Add the garlic, lemon juice, tahini and oil. Mix well.

Source:

Simple Healthy Recipes
Oklahoma Nutrition Information and Education
ONIE Project

Nutrition Information

Serving Size: 1/6 of recipe

**Nutrients**	**Amount**
**Total Calories**	**117**
**Total Fat**	**6 g**
Saturated Fat	1 g
Cholesterol	0 mg
**Sodium**	**102 mg**
**Carbohydrates**	**13 g**
Dietary Fiber	4 g
Total Sugars	2 g
Added Sugars included	0 g
**Protein**	**4 g**
Vitamin D	0 mcg
Calcium	29 mg
Iron	1 mg
Potassium	141 mg

Nutrients will display if the data is available

Please note: nutrient values are subject to change as data is updated

MyPlate Food Groups

Vegetables	1/4 cups
Protein Foods	1 ounces

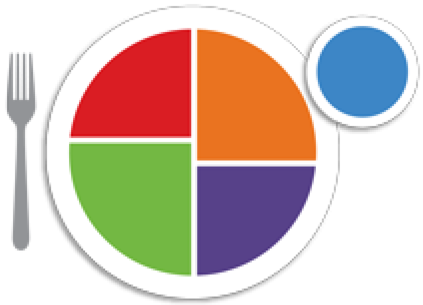

Visit MyPlate.gov

Mediterranean Chicken and White Bean Salad

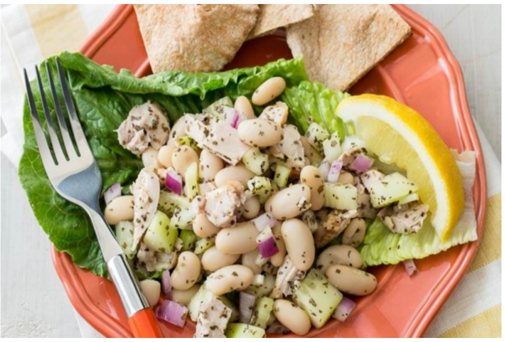

Makes: 4 Servings
Preparation Time: 20 minutes

This refreshing salad starts with cooked chicken, white beans, cucumber, and onion with a splash of fresh lemon juice, oil and seasonings for a delicious combination. Try it with tuna and chopped tomatoes for variety.

Ingredients

- 1 cup skinless cooked chicken (diced into 1/2 inch pieces)
- 1 can 15.5 ounce low-sodium white beans (drained and rinsed with cold water)
- 1 cucumber (peeled and diced into 1/2 inch pieces)
- 1/4 red or white onion (peeled and chopped into 1/2 inch pieces)
- 2 tablespoons vegetable oil
- 1/4 cup lemon juice
- 1 tablespoon dried basil or parsley leaves
- 1/4 teaspoon salt
- 1/4 teaspoon black pepper

Directions

1. Put everything in the bowl and gently toss.
2. Serve right away, or cover and refrigerate for up to 2 days.

Source:

USDA Center for Nutrition Policy and Promotion

Nutrition Information

Serving Size: 1 cup

Nutrients	Amount
**Total Calories**	**297**
**Total Fat**	**11 g**
Saturated Fat	2 g
Cholesterol	32 mg
**Sodium**	**288 mg**
**Carbohydrates**	**31 g**
Dietary Fiber	8 g
Total Sugars	2 g
Added Sugars included	0 g
**Protein**	**20 g**
Vitamin D	0 mcg
Calcium	138 mg
Iron	6 mg
Potassium	820 mg

Nutrients will display if the data is available

Please note: nutrient values are subject to change as data is updated

MyPlate Food Groups

Vegetables	1 cup
Protein Foods	1 ounce

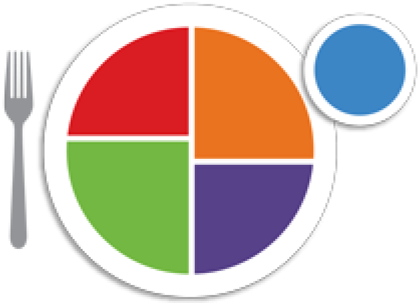

Visit MyPlate.gov

Broccoli-Cheddar Frittata

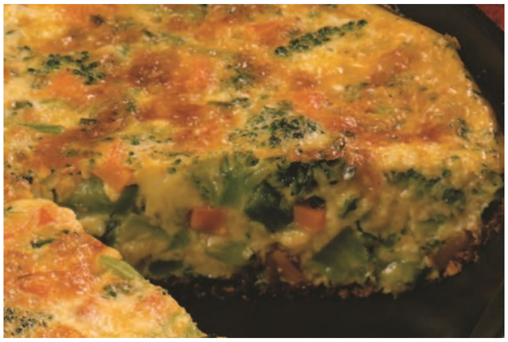

Makes: 6 Servings
Preparation Time: 10 minutes
Cook Time: 25 minutes

This egg dish provides a quarter cup serving of vegetables during breakfast and uses reduced-fat cheddar cheese and non-fat milk to limit fat. Prep and cook in about 30 minutes makes this recipe an easy breakfast for both the weekday or the weekend.

Ingredients

- 1 package (10-oz) frozen chopped broccoli
- 1/4 cup water
- 8 eggs
- 1/4 cup nonfat or low-fat milk
- 2 teaspoons prepared mustard
- 1 teaspoon seasoned salt
- 1/8 teaspoon pepper
- 3/4 cup shredded reduced-fat cheddar cheese (3 ounces)
- 1 tablespoon chopped green onion
- 1 small carrot, diced
- nonstick cooking spray

Directions

1. Combine broccoli, carrot (if desired), and water in 10-inch nonstick skillet.
2. Cook over medium heat until tender, stirring occasionally to break up broccoli, about 10 minutes; drain well.
3. Beat eggs, milk, mustard, salt, and pepper in large bowl until blended.
4. Add broccoli mixture, cheese, and green onion; mix well.
5. Coat same skillet with cooking spray; heat over medium heat until eggs are almost set, 8 to 10 minutes. Remove from heat.
6. Cover and let stand until eggs are completely set and no visible liquid egg remains, 8 to 10 minutes. Cut into wedges.

Source:

Meeting Your MyPlate Goals on a Budget

Nutrition Information

Serving Size: 1/6 of recipe (142 g)

**Nutrients**	**Amount**
**Total Calories**	**160**
**Total Fat**	**8 g**
Saturated Fat	3 g
Cholesterol	257 mg
**Sodium**	**470 mg**
**Carbohydrates**	**3 g**
Dietary Fiber	1 g
Total Sugars	2 g
Added Sugars included	0 g
**Protein**	**13 g**
Vitamin D	1 mcg
Calcium	193 mg
Iron	1 mg
Potassium	184 mg

Nutrients will display if the data is available

Please note: nutrient values are subject to change as data is updated

MyPlate Food Groups

Vegetables	1/4 cups
Protein Foods	1 ounce
Dairy	1/2 cups

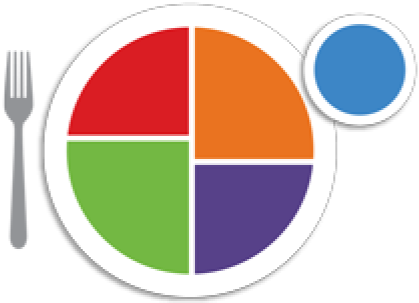

Visit MyPlate.gov

Grilled Asparagus and Shrimp Quinoa Salad with Lemon Vinaigrette

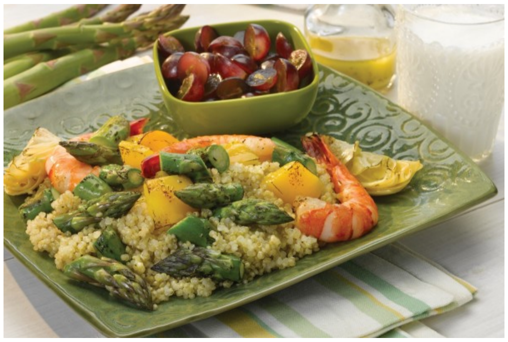

Makes: 4 Servings
Preparation Time: 45 minutes

Grilling brings out the flavor in fresh vegetables like asparagus. Add shrimp, quinoa, and refreshing lemon vinaigrette, and you have a delicious and quick spring or summer salad.

Ingredients

- 2 cups fresh asparagus, large spears (cut into 1" pieces)
- 1/2 yellow or red bell pepper (cut into 1/2" pieces)
- 1 clove garlic (minced)
- 1 14 ounce can quartered artichoke hearts (drained)
- 12 ounces fresh or frozen large raw shrimp (peeled and deveined)
- 1 1/2 cups dry quinoa (cooked according to package directions)

For the Lemon Vinaigrette:

- 1 teaspoon grated lemon peel (optional)
- 3 tablespoons fresh or bottled lemon juice
- 3 tablespoons olive oil
- 1 teaspoon Dijon mustard
- 1/2 teaspoon dried thyme leaves
- 1/2 teaspoon ground black pepper

Directions

1. Place vinaigrette ingredients in a small bowl and whisk; set aside.
2. Cut vegetables as directed.
3. Heat grill and grilling tray.
4. Place vegetables and shrimp in a large bowl; add about 1/3 of the vinaigrette (about 3 tablespoons) and toss.
5. Spread shrimp-vegetable mixture over hot grilling tray.
6. Grill, turning shrimp and vegetables, until the flesh of the shrimp is an opaque color (about 5 to 6 minutes); remove from grill.
7. Serve grill mixture over cooked quinoa, and drizzle with vinaigrette.

Source:

Nutrition Information

Serving Size: 1/4 of the recipe

Nutrients	Amount
**Total Calories**	**460**
**Total Fat**	**16 g**
Saturated Fat	2 g
Cholesterol	115 mg
**Sodium**	**420 mg**
**Carbohydrates**	**51 g**
Dietary Fiber	7 g
Total Sugars	6 g
Added Sugars included	0 g
**Protein**	**29 g**
Vitamin D	0 mcg
Calcium	200 mg
Iron	6 mg
Potassium	860 mg

Nutrients will display if the data is available

Please note: nutrient values are subject to change as data is updated

MyPlate Food Groups

Vegetables	1 1/4 cups
Grains	2 1/2 ounces
Protein Foods	2 1/2 ounces

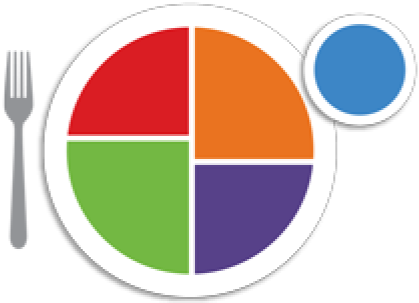

Visit MyPlate.gov

Grilled Vegetables

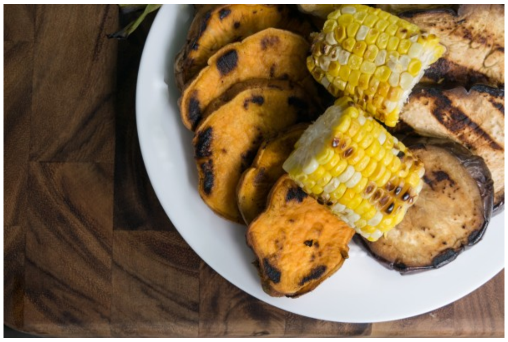

Makes: 6 servings

Cooking out tonight? Try cooking vegetables coated in oil and garlic on the grill for a delicious side dish. If you don't have a grill, you can make these vegetables in the oven.

Ingredients

- 2 tablespoons vegetable oil
- 2 garlic cloves (finely chopped)
- 3 sweet potatoes (cut into 1-inch slices)
- 3 corn cobs (cut into 2-inch sections)
- 1 eggplant (cut into 1/2-inch slices)
- 12 green onions (trimmed)

Directions

1. Wash hands with soap and water.
2. Mix oil and garlic in a large bowl. Add vegetables and toss.
3. Place vegetable on broiler pan or grill. Cook 10 minutes, turning twice until vegetables are tender.
4. Place vegetables on platter. Serve.

Source:

US Department of Health and Human Services
Centers for Disease Control and Prevention (CDC)

Nutrition Information

Serving Size: 1/6 of recipe (254g)

Nutrients	Amount
**Total Calories**	**149**
**Total Fat**	**5 g**
Saturated Fat	1 g
Cholesterol	0 mg
**Sodium**	**48 mg**
**Carbohydrates**	**25 g**
Dietary Fiber	4 g
Total Sugars	7 g
Added Sugars included	0 g
**Protein**	**3 g**
Vitamin D	0 mcg
Calcium	45 mg
Iron	1 mg
Potassium	459 mg

Nutrients will display if the data is available

Please note: nutrient values are subject to change as data is updated

MyPlate Food Groups

Vegetables 1 cups

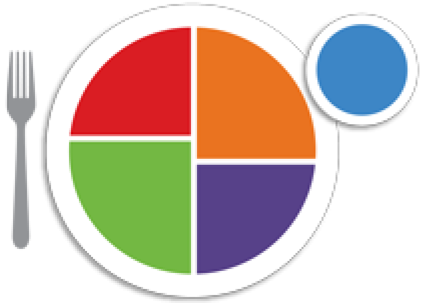

Visit MyPlate.gov

Angel Food Pastry with Fresh Berries and Whipped Cream

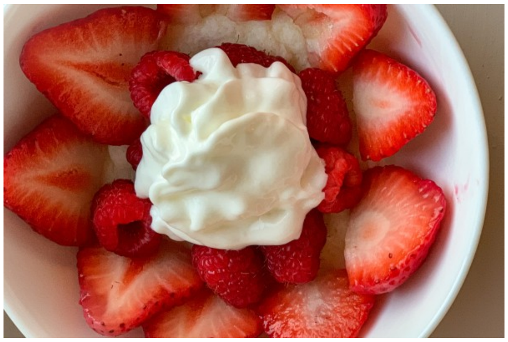

Makes: 1 Servings

Light and fluffy angel food cake is mixed with yogurt and a sprinkle of berries on top.

Ingredients

- 1 ounce angel food cake mix (3 tablespoons)
- 1/8 cup low-fat vanilla yogurt (2 tablespoons)
- 1/4 cup raspberries
- 2 strawberries, sliced
- 1 1/2 tablespoons lite whipped topping

Directions

1. Wash hands with soap and water.
2. Scoop 3 tablespoons of angel food cake mix into a microwave-safe cup or mug.
3. Add 2 tablespoons of vanilla yogurt and stir.
4. Place in the center of the microwave oven and microwave for 1 minute.
5. Take out of microwave and let cool for 1 minute.
6. Using a knife, cut around the sides of the cup to loosen the cake and "dump" it on a plate.
7. Dole 1 tablespoon of whipped topping on top of the cake.
8. Sprinkle raspberries around the angel cake and add sliced strawberries to top.
9. Enjoy.

Source:

Community Services for Autistic Adults and Children (CSAAC)

Nutrition Information

Serving Size: 1 Angel Food Pastry

Nutrients	Amount
**Total Calories**	**174**
**Total Fat**	**2 g**
Saturated Fat	1 g
Cholesterol	2 mg
**Sodium**	**260 mg**
**Carbohydrates**	**37 g**
Dietary Fiber	3 g
Total Sugars	27 g
Added Sugars included	10 g
**Protein**	**4 g**
Vitamin D	0 mcg
Calcium	116 mg
Iron	0 mg
Potassium	204 mg

Nutrients will display if the data is available

Please note: nutrient values are subject to change as data is updated

MyPlate Food Groups

Fruits 1/2 cups

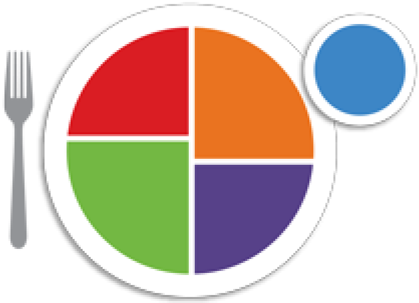

Visit MyPlate.gov

Tropical Yogurt Pops

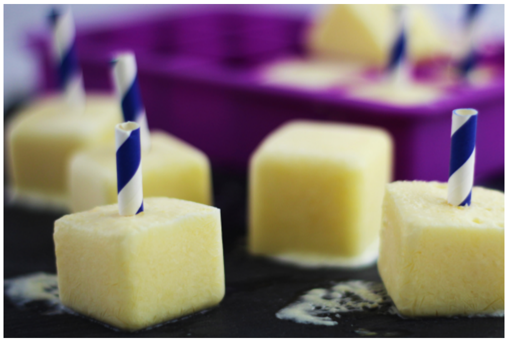

Makes: 4 Servings

Enjoy a tropical treat with these frozen yogurt pops! They are fun to make and have 1/4 cup of fruit per serving!

Ingredients

- 1 cup low-fat vanilla yogurt
- 1/2 cup mango pieces (fresh or frozen)
- 1/2 cup pineapple pieces (fresh, canned, or frozen)
- 1/4 cup cup coconut water
- 1 ice cube tray (or paper cups)

Directions

1. Place all ingredients into blender.
2. Blend on high until smooth.
3. Pour into 4 small paper cups (or 8 ice cubes) and place in popsicle sticks (or cut paper straws).
4. Freeze.
5. Enjoy as a frozen treat!

Source:

USDA Center for Nutrition Policy and Promotion

Nutrition Information

Nutrients	Amount
**Total Calories**	**65**
**Total Fat**	**1 g**
Saturated Fat	1 g
Cholesterol	4 mg
**Sodium**	**43 mg**
**Carbohydrates**	**11 g**
Dietary Fiber	1 g
Total Sugars	10 g
Added Sugars included	0 g
**Protein**	**4 g**
Vitamin D	0 mcg
Calcium	N/A
Iron	N/A
Potassium	N/A

Nutrients will display if the data is available

Please note: nutrient values are subject to change as data is updated

MyPlate Food Groups

Fruits 1/4 cups
Dairy 1/4 cups

Visit MyPlate.gov

Chocolate Chip Yogurt Cookies

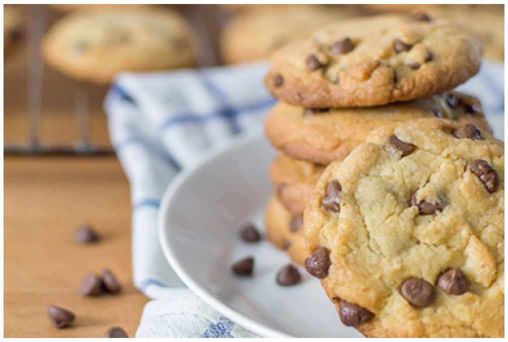

Makes: 36 servings

Add extra calcium to your cookies with yogurt. These cookies also use whole wheat flour which adds fiber and other nutrients.

Ingredients

- 1/2 cup sugar
- 1/2 cup brown sugar, firmly packed
- 1/2 cup margarine (1 stick)
- 1/2 cup plain yogurt, non-fat
- 1 1/2 teaspoons vanilla
- 3/4 cup all-purpose flour
- 1 cup whole wheat flour
- 1/2 teaspoon baking soda
- 1/2 cup chocolate chips (miniature, or carob chips)

Directions

1. Wash hands with soap and water.
2. Heat oven to 375 °F.
3. In a large bowl combine sugar, brown sugar and margarine; beat until light and fluffy.
4. Add yogurt and vanilla; blend well. Stir in flour and baking soda; mix well. Stir in chocolate chips.
5. Drop dough by rounded teaspoonfuls 2 inches apart onto un-greased cookie sheets. Bake at 375 °F for 8 to 12 minutes or until light and golden brown.
6. Cool 1 minute, remove from cookie sheets.

Source:

Eat Smart New York!
Cornell University Cooperative Extension

Nutrition Information

Serving Size: 1 cookie

Nutrients	Amount
**Total Calories**	**79**
**Total Fat**	**3 g**
Saturated Fat	1 g
Cholesterol	0 mg
**Sodium**	**42 mg**
**Carbohydrates**	**12 g**
Dietary Fiber	1 g
Total Sugars	7 g
Added Sugars included	7 g
**Protein**	**1 g**
Vitamin D	0 mcg
Calcium	12 mg
Iron	0 mg
Potassium	37 mg

Nutrients will display if the data is available

Please note: nutrient values are subject to change as data is updated

MyPlate Food Groups

Grains 1/2 ounces

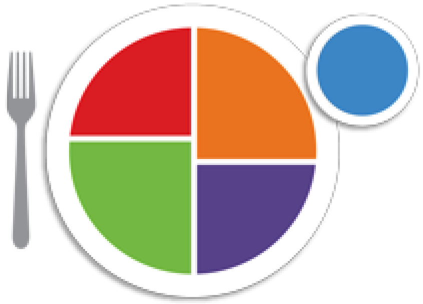

Visit MyPlate.gov

Apple-Stuffed Squash

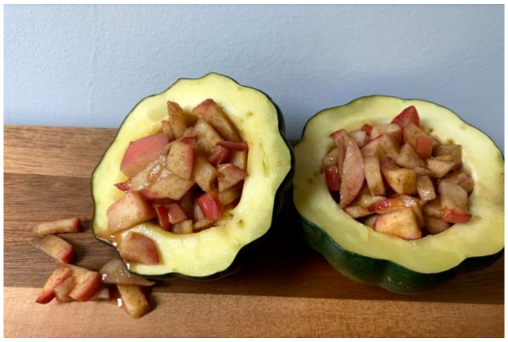

Makes: 8 Servings

All you need is a microwave to cook this delicious and seasonal side dish.

Ingredients

- 4 acorn squashes, washed
- 1 tablespoon butter (or margarine)
- 2 apples, chopped
- 2 teaspoons brown sugar, packed
- 1/2 teaspoon cinnamon

Directions

1. Wash hands with soap and water.
2. Cut squash into halves and remove seeds. Place in a glass dish, cover with plastic wrap and microwave on high for 5 minutes.
3. Melt butter in a separate bowl in the microwave. Mix in apples, sugar, and cinnamon. Microwave for 1 1/2 minutes.
4. Spoon apple filling into each squash half. Cover and microwave on high for 3-5 minutes until squash and apples are tender.
5. Serve warm.

Source:

University of Maryland Cooperative Extension
SNAP-Ed Program

Nutrition Information

Serving Size: 1/2 squash with filling, 1/8 of recipe

Nutrients	Amount
**Total Calories**	**122**
**Total Fat**	**2 g**
Saturated Fat	1 g
Cholesterol	4 mg
**Sodium**	**7 mg**
**Carbohydrates**	**28 g**
Dietary Fiber	4 g
Total Sugars	5 g
Added Sugars included	1 g
**Protein**	**2 g**
Vitamin D	0 mcg
Calcium	76 mg
Iron	2 mg
Potassium	787 mg

Nutrients will display if the data is available

Please note: nutrient values are subject to change as data is updated

MyPlate Food Groups

Fruits	1/4 cups
Vegetables	2 cups

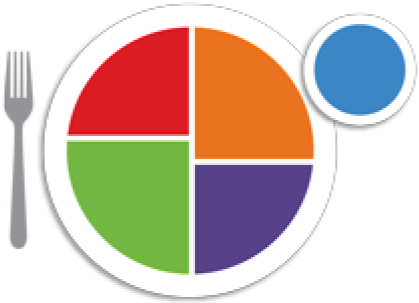

Visit MyPlate.gov

Rainbow Veggie Salad

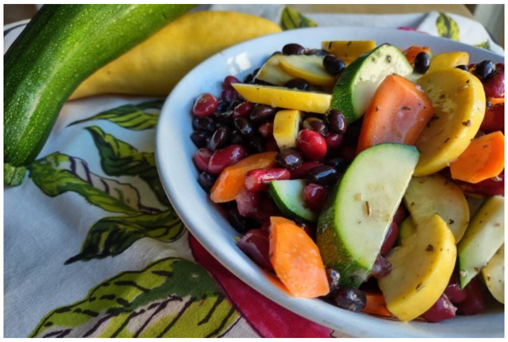

Makes: 10 servings

This easy and colorful salad tastes best after marinating it overnight. Enjoy it as a side dish at lunch or dinner.

Ingredients

- 1 can (15.5 ounces) red kidney beans, low-sodium (drained and rinsed)
- 1 can (15.5 ounces) black beans, low-sodium (drained and rinsed)
- 3 carrots (scrubbed and sliced)
- 1 medium yellow squash (washed and sliced)
- 1 medium zucchini (washed and sliced)
- 1/2 cup light Italian dressing
- 1/2 teaspoon black pepper

Directions

1. Wash hands with soap and water.
2. Mix all the vegetables together in a large bowl.
3. Pour dressing over vegetables.
4. Sprinkle with pepper.
5. Stir gently, coating all vegetables.
6. Cover and refrigerate at least 8 hours.

Source:

2009 Recipe Calendar
University of Maryland Extension
Food Supplement Nutrition Education Program

Nutrition Information

Serving Size: 2/3 cup (106g)

Nutrients	Amount
**Total Calories**	**95**
**Total Fat**	**1 g**
Saturated Fat	0 g
Cholesterol	0 mg
**Sodium**	**257 mg**
**Carbohydrates**	**18 g**
Dietary Fiber	6 g
Total Sugars	4 g
Added Sugars included	0 g
**Protein**	**6 g**
Vitamin D	0 mcg
Calcium	44 mg
Iron	2 mg
Potassium	424 mg

Nutrients will display if the data is available

Please note: nutrient values are subject to change as data is updated

MyPlate Food Groups

Vegetables	1 cups
Protein Foods	2 ounces

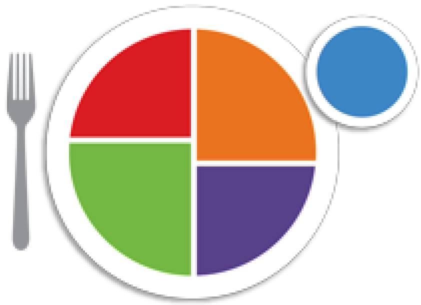

Visit MyPlate.gov

Grilled Steak and Peppers Salad with Pears

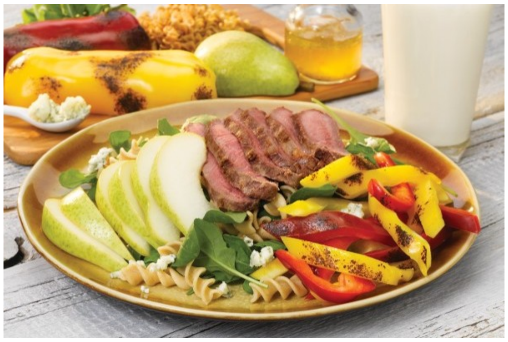

Makes: 4 Servings
Preparation Time: 30 minutes

Grill whole bell peppers along with steak, then add pear slices, and you have the foundation of a delicious and colorful Italian-influenced salad.

Ingredients

- 2 cups (4 oz) white-wheat rotini pasta (uncooked)
- 1 yellow bell pepper
- 1 red bell pepper
- 1/2 teaspoon olive oil
- 12 ounces boneless choice beef top sirloin
- 2 pears
- 3 cups arugula
- 1/4 cup crumbled Gorgonzola cheese

For Vinaigrette

- 1 tablespoon olive oil
- 3 tablespoons distilled vinegar
- 1/4 cup 100% white grape juice or apple juice
- 1/4 teaspoon salt (optional)
- 1 tablespoon Italian herb blend, salt-free

Directions

1. In a small bowl, whisk olive oil, vinegar, grape or apple juice, salt, and herb blend.
2. Boil water and cook pasta according to package instructions. Drain pasta, rinse in cool water.
3. Heat grill, rub peppers with 1/2 tsp olive oil. Grill whole peppers, turning as needed, until skin begins to brown and bubble. At the same time, grill beef sirloin to 145 °F, turning once. Remove from grill, let cool slightly.
4. Slice peppers into strips, discarding seeds and stem. Cut sirloin across the grain into thin slices. Slice pears into thin wedges.
5. Toss arugula and pasta in large bowl.
6. To serve, evenly divide pasta-arugula onto four plates, arrange beef, peppers, and pears on top, drizzle with vinaigrette, and sprinkle with crumbled Gorgonzola cheese.

Source:

Nutrition Information

Serving Size: 1/4 of recipe

Nutrients	Amount
**Total Calories**	**360**
**Total Fat**	**12 g**
Saturated Fat	4 g
Cholesterol	65 mg
**Sodium**	**330 mg**
**Carbohydrates**	**44 g**
Dietary Fiber	7 g
Total Sugars	13 g
Added Sugars included	0 g
**Protein**	**27 g**
Vitamin D	0 mcg
Calcium	108 mg
Iron	3 mg
Potassium	584 mg

Nutrients will display if the data is available

Please note: nutrient values are subject to change as data is updated

MyPlate Food Groups

Fruits	1/2 cups
Vegetables	3/4 cups
Grains	1 ounce
Protein Foods	2 ounces
Dairy	1/4 cups

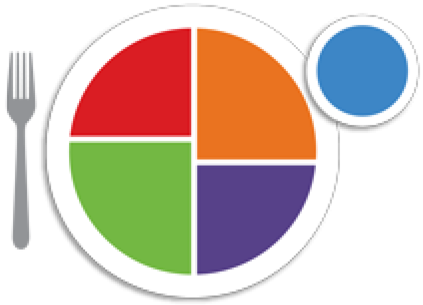

Visit MyPlate.gov

Grilled Lamb Salad

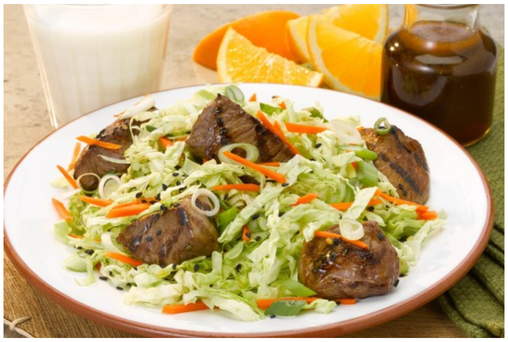

Makes: 4 Servings
Preparation Time: 30 minutes

Grilled lamb is delightful with any green salad, especially this Asian-inspired version.

Ingredients

- 3 green onions (sliced)
- 12 ounces boneless leg of lamb, cut into 1-inch cubes (if cubed lamb is not available at your meat market, ask the butcher to prepare it for you)
- 4 bamboo skewers
- 6 cups shredded Savoy, Napa, or green cabbage
- 2 carrots (shredded)
- 2 tablespoons black or white sesame seeds
- 1 small jicama, julienned (optional)

For the Marinade/Dressing

- 2 tablespoons sugar
- 2 tablespoons rice vinegar
- 3 tablespoons soy sauce, low sodium
- 1/4 cup water
- 1 tablespoon vegetable oil

Directions

1. Mix marinade/dressing ingredients; divide evenly.
2. Place lamb cubes in a glass dish; pour half of the marinade/dressing over the lamb.
3. Cover and refrigerate at least two hours.
4. In a large bowl, toss cabbage, carrots, jicama (optional), onions, and sesame seeds.
5. Remove lamb and discard marinade.
6. Soak bamboo skewers in water for 30 minutes; divide lamb among the 4 skewers.
7. On hot grill, cook the lamb skewers 5 to 6 minutes per side (or to desired degree of doneness).
8. Remove lamb from skewers, add to salad, and toss.
9. To serve, divide salad evenly on four plates. Drizzle with remaining dressing.

Source:

Nutrition Information

Serving Size: 1/4 of the recipe

Nutrients	Amount
**Total Calories**	**260**
**Total Fat**	**9 g**
Saturated Fat	3 g
Cholesterol	70 mg
**Sodium**	**360 mg**
**Carbohydrates**	**20 g**
Dietary Fiber	8 g
Total Sugars	9 g
Added Sugars included	6 g
**Protein**	**23 g**
Vitamin D	0 mcg
Calcium	100 mg
Iron	3 mg
Potassium	643 mg

Nutrients will display if the data is available

Please note: nutrient values are subject to change as data is updated

MyPlate Food Groups

Vegetables	1 3/4 cups
Protein Foods	3 ounces

Visit MyPlate.gov

Grilled Fruit

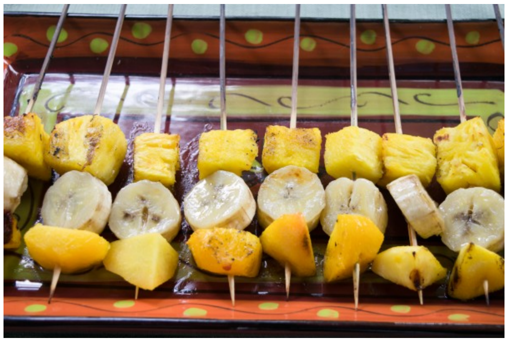

Makes: 3 Servings

Fruit kabobs cooked on the grill make a tasty and healthy dessert.

Ingredients

- 1 cup pineapple chunks (fresh or canned)
- 1 peach (cubed)
- 1 banana (sliced)

Directions

1. Wash hands with soap and water.
2. Place fruit chunks on a skewer to make kabobs.
3. Grill or broil on low heat until the fruit is hot and slightly golden.

Source:

University of Wyoming Extension
Cent$ible Nutrition Program

Nutrition Information

Serving Size: 1/3 of recipe

Nutrients	Amount
**Total Calories**	**104**
**Total Fat**	**0 g**
Saturated Fat	0 g
Cholesterol	0 mg
**Sodium**	**1 mg**
**Carbohydrates**	**27 g**
Dietary Fiber	2 g
Total Sugars	21 g
Added Sugars included	0 g
**Protein**	**1 g**
Vitamin D	0 mcg
Calcium	17 mg
Iron	0 mg
Potassium	337 mg

Nutrients will display if the data is available

Please note: nutrient values are subject to change as data is updated

MyPlate Food Groups

Fruits 1 cups

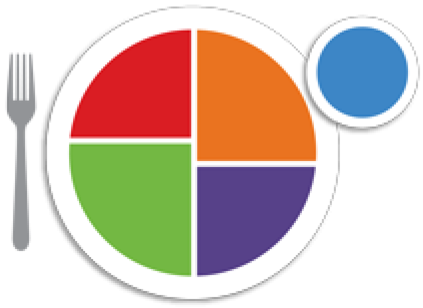

Visit MyPlate.gov

Grilled Vegetable Packets

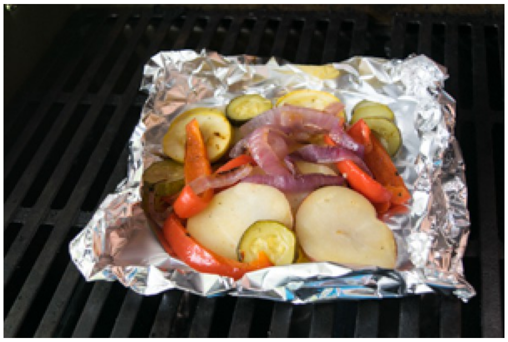

Makes: 5 servings

Veggies are wrapped in foil and cooked on the grill for an easy side dish to your cookout. Don't have a grill? This recipe can also be made in the oven.

Ingredients

- 2 small zucchinis (sliced)
- 2 small yellow squashes (sliced)
- 4 small red potatoes (scrubbed well and sliced)
- 1/2 red onion (sliced)
- 1/2 red or green bell pepper (sliced)
- 1/4 cup light Italian salad dressing
- salt and pepper (optional, to taste)

Directions

1. Wash hands with soap and water.
2. Heat grill to medium heat or 350 °F.
3. Wash vegetables and slice.
4. Toss in a large bowl. Add dressing and toss until all vegetables are coated.
5. Tear 2 large squares of aluminum foil and place half of the vegetable mixture on each piece. Place an equal piece of foil over the top of vegetable mixture and fold bottom piece with top sheet to form a packet.
6. Place on heated grill for 20 to 30 minutes or until the potatoes are tender. If you don't have a grill, bake Veggie Packets in the oven at 400 °F for 20 to 30 minutes.
7. Before you open the packets, poke holes in the foil with a fork. Be very careful opening the foil as the steam will be very hot and could burn you!
8. Empty vegetables onto serving plate or serve from foil packets.

Source:

Connecticut Food Policy Council

Nutrition Information

Serving Size: 3/4 cup (316g)

Nutrients	Amount
**Total Calories**	**133**
**Total Fat**	**0 g**
Saturated Fat	0 g
Cholesterol	0 mg
**Sodium**	**144 mg**
**Carbohydrates**	**29 g**
Dietary Fiber	4 g
Total Sugars	5 g
Added Sugars included	0 g
**Protein**	**4 g**
Vitamin D	0 mcg
Calcium	38 mg
Iron	2 mg
Potassium	868 mg

Nutrients will display if the data is available

Please note: nutrient values are subject to change as data is updated

MyPlate Food Groups

Vegetables 2 cups

[Visit MyPlate.gov]

Skinny Pizza

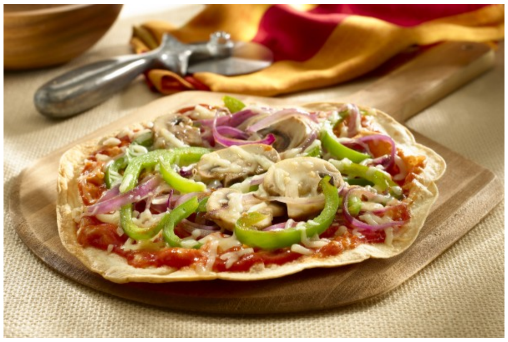

Makes: 4 Servings
Preparation Time: 30 minutes

Next time they ask for pizza, you can feel good about saying "yes!" Flour tortillas make for a crispy crust, perfect for loading with low-sodium tomato sauce, low-fat cheese, and lots of fresh veggies.

Ingredients

- 4 6" flour tortillas
- 1/2 teaspoon extra virgin olive oil
- 2 cups sliced mushrooms (white button or baby Portobello)
- 1 green bell pepper (thinly sliced, about 1 cup)
- 1 red onion (thinly sliced, about 1 cup)
- 2 teaspoons minced garlic
- 1/2 cup low-sodium tomato sauce
- 1/2 cup shredded fat-free mozzarella cheese
- 2 teaspoons grated reduced-fat Parmesan cheese

Directions

1. Heat oven to 400 °F.
2. Place tortillas on 2 large baking sheets.
3. Cook, flipping once, until crisp (about 10 minutes). Set aside.
4. Meanwhile, heat oil in large skillet over medium heat. Add mushrooms, peppers, onions, and garlic.
5. Cook until all vegetables are soft and tender (about 10 minutes). Set aside.
6. Spread tortilla crust with 2 tablespoons tomato sauce, 1/4 cup vegetable mixture, 2 tablespoons mozzarella cheese, and 1/2 teaspoon of Parmesan cheese. Repeat with remaining crusts and topping ingredients.
7. Transfer pizzas to same baking sheets.
8. Cook until cheese is melted and edges of tortillas are golden brown (about 10 minutes).

Source:

The Best of La Cocina GOYA: Healthy, Tasty, Affordable Latin Cooking/ Lo Mejor de la Cocina GOYA: Cocina Latina Saludable, Rica y Económica
GOYA Foods

Nutrition Information

Nutrients	Amount
**Total Calories**	**190**
**Total Fat**	**5 g**
Saturated Fat	1 g
Cholesterol	5 mg
**Sodium**	**450 mg**
**Carbohydrates**	**26 g**
Dietary Fiber	3 g
Total Sugars	6 g
Added Sugars included	2 g
**Protein**	**9 g**
Vitamin D	0 mcg
Calcium	200 mg
Iron	2 mg
Potassium	375 mg

Nutrients will display if the data is available

Please note: nutrient values are subject to change as data is updated

MyPlate Food Groups

Vegetables	1 1/4 cups
Grains	1 1/2 ounces
Dairy	1/2 cups

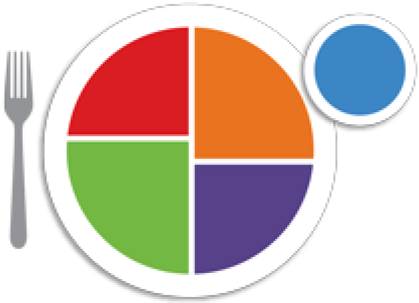

Visit MyPlate.gov

Rice-Crusted Pizza

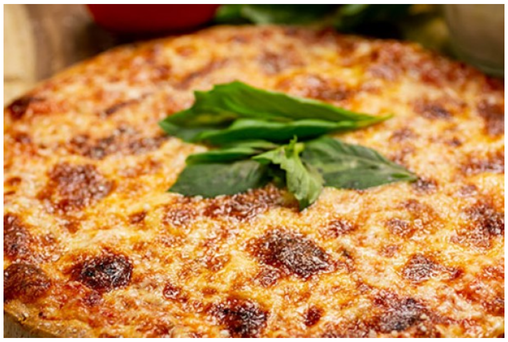

Makes: 12 servings

A fun and unique twist on pizza using rice as the crust. Enjoy this main dish with all of your favorite toppings!

Ingredients

- 2 cups rice (cooked)
- 16 ounces mozzarella cheese, part skim
- 1 egg
- 1/4 teaspoon salt
- 2 cups tomato purée, low-sodium

Directions

1. Wash hands with soap and water.
2. Heat oven to 350 °F. Grease a 12-inch pizza pan or baking sheet.
3. Cook rice following directions on the package; set aside.
4. Grate cheese; set aside.
5. Crack egg and place in mixing bowl, stirring to blend. Measure and add 2 cups cooked rice, 1 cup grated cheese, and salt. Mix well to combine ingredients.
6. Spread rice mixture in prepared pan, pressing firmly and making outer edge slightly raised.
7. Spread tomato purée evenly over rice mixture.
8. Place pan in oven. Bake for 25 minutes.
9. Remove pan from oven sprinkle pizza with remaining cheese. Bake 5 minutes longer or until cheese melts.
10. Remove pizza from oven. Cut into 12 slices.

Create-a-Flavor Changes:

- Try different kinds of rice.
- Try different kinds of cheese.
- Use sliced or chopped green peppers, onions, mushrooms, or other vegetables as toppings.

Source:

Cooking Up Fun - A Pyramid of Snacks
Cornell University Cooperative Extension

Nutrition Information

Serving Size: 1 slice, 1/12 of recipe (110g)

Nutrients	Amount
**Total Calories**	**175**
**Total Fat**	**9 g**
Saturated Fat	4 g
Cholesterol	37 mg
**Sodium**	**313 mg**
**Carbohydrates**	**12 g**
Dietary Fiber	1 g
Total Sugars	2 g
Added Sugars included	3 g
**Protein**	**12 g**
Vitamin D	0 mcg
Calcium	292 mg
Iron	1 mg
Potassium	181 mg

Nutrients will display if the data is available

Please note: nutrient values are subject to change as data is updated

MyPlate Food Groups

Vegetables	1/4 cups
Grains	1/2 ounces
Dairy	1 cups

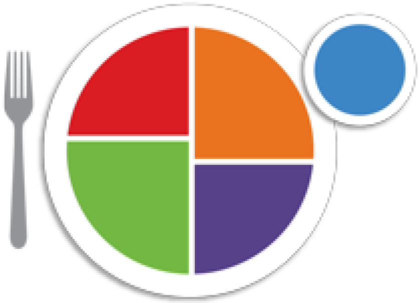

Visit MyPlate.gov

Curried Chickpea Salad

Makes: 6 Servings
Preparation Time: 10 minutes

This dish can be served on whole wheat bread, on a bed of lettuce, or as a dip with your favorite veggies!

Ingredients

- 2 cans (15 ounces each) chickpeas, low-sodium, rinsed and drained
- 1/2 cup celery, diced
- 1/2 cup apple, diced
- 1/4 cup red onion, diced
- 1/4 cup cranberries, dried
- 1/4 cup mayonnaise, low fat
- 1 teaspoon dijon mustard
- 2 teaspoons curry powder
- 1/4 teaspoon thyme, dried
- 1/8 teaspoon black pepper, ground
- salt (1/2 teaspoon, optional)

Directions

1. Wash hands with soap and water.
2. Add the chickpeas to a large bowl and mash with a potato masher. You can mash them to be as smooth or coarse as you desire.
3. Add all of the remaining ingredients to the bowl and mix until everything is combined.

Source:

Penn State Extension
Nutrition Links Program

Nutrition Information

Serving Size: 1/2 cup

Nutrients	Amount
**Total Calories**	**181**
**Total Fat**	**6 g**
Saturated Fat	1 g
Cholesterol	N/A
**Sodium**	**282 mg**
**Carbohydrates**	**26 g**
Dietary Fiber	7 g
Total Sugars	8 g
Added Sugars included	3 g
**Protein**	**7 g**
Vitamin D	N/A
Calcium	59 mg
Iron	2 mg
Potassium	N/A

Nutrients will display if the data is available

Please note: nutrient values are subject to change as data is updated

MyPlate Food Groups

Fruits	1/4 cups
Vegetables	1 cups
Protein Foods	3 ounces

Visit MyPlate.gov

Roasted Chickpeas (Garbanzo Beans)

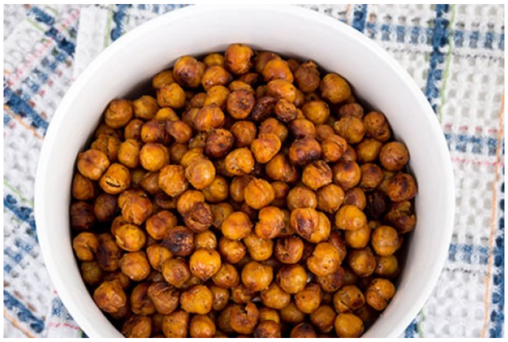

Makes: 4 Servings
Cook Time: 45 minutes

These crunchy chickpeas make a great on-the-go snack or a healthy topping to your favorite salad. Experiment with different spices to see which ones you like best.

Ingredients

- 1 can (15 ounces) low-sodium garbanzo beans, drained and rinsed
- 1 tablespoon canola oil (or cooking oil of choice)
- 1/8 teaspoon salt
- spices of your choice, optional (try garlic, paprika, sage, etc.)

Directions

1. Wash hands with soap and water.
2. Preheat oven to 400 degrees F.
3. Drain and rinse the beans until there is no foamy residue. Pick out the skins and discard. Wrap the drained beans in a clean towel to dry thoroughly.
4. Measure oil into a 9x13-inch baking dish or foil lined pan. Pour beans into dish, sprinkle with salt and spices. Mix oil and spices well to coat all the beans.
5. Bake for 45 to 60 minutes, stirring or shaking every 15 minutes. Longer makes more crunch, but watch to prevent burning!

Source:

USDA Supplemental Nutrition Assistance Program (SNAP)

Nutrition Information

Serving Size: 1/4 cup

Nutrients	Amount
**Total Calories**	**124**
**Total Fat**	**5 g**
Saturated Fat	0 g
Cholesterol	0 mg
**Sodium**	**216 mg**
**Carbohydrates**	**14 g**
Dietary Fiber	5 g
Total Sugars	3 g
Added Sugars included	0 g
**Protein**	**5 g**
Vitamin D	0 mcg
Calcium	37 mg
Iron	1 mg
Potassium	153 mg

Nutrients will display if the data is available

Please note: nutrient values are subject to change as data is updated

MyPlate Food Groups

Vegetables	1/2 cups
Protein Foods	2 1/2 ounces

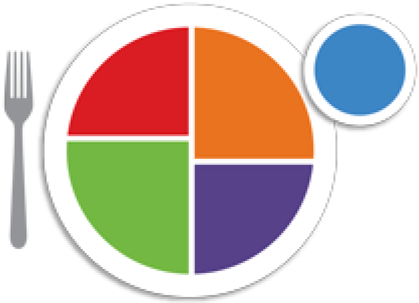

Visit MyPlate.gov

Heavenly Deviled Eggs

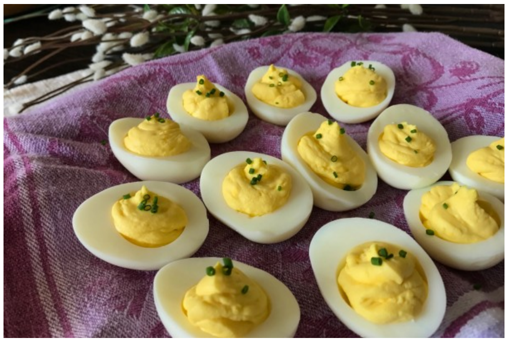

Makes: 6 Servings

Versatile and easy to prepare, these deviled eggs are great as an appetizer, side dish, or snack.

Ingredients

- 6 eggs (in shell)
- 2 tablespoons light mayonnaise
- 1 teaspoon mustard

Directions

1. Wash hands with soap and water.
2. Put eggs into a saucepan. Cover with cold water.
3. Bring eggs to a simmer (small bubbles) and cook for 12 minutes.
4. Remove from the heat and drain.
5. Crack eggs under cold water and allow to cool. Remove shells.
6. Split eggs in half, lengthwise and remove yolks.
7. Put yolks, dressing and mustard into a small zipper-lock plastic bag to mix.
8. Cut a small hole in a lower corner of the bag. Squeeze mixture into egg white halves. Garnish as desired.

Source:

Simple Healthy Recipes
Oklahoma Nutrition Information and Education
ONIE Project

Nutrition Information

Serving Size: 2 prepared egg halves, 1/6 of recipe

Nutrients	Amount
**Total Calories**	**90**
**Total Fat**	**6 g**
Saturated Fat	2 g
Cholesterol	212 mg
**Sodium**	**174 mg**
**Carbohydrates**	**1 g**
Dietary Fiber	0 g
Total Sugars	1 g
Added Sugars included	0 g
**Protein**	**6 g**
Vitamin D	40 mcg
Calcium	25 mg
Iron	1 mg
Potassium	65 mg

Nutrients will display if the data is available

Please note: nutrient values are subject to change as data is updated

MyPlate Food Groups

Protein Foods 1 ounce

Visit MyPlate.gov

Black Bean Quesadillas

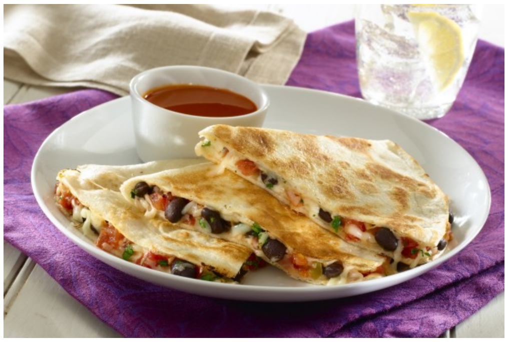

Makes: 8 Servings
Preparation Time: 15 minutes

Enjoy a quesadilla any night of the week! This quesadilla recipe can be served as a tasty lunch, dinner, or side dish.

Ingredients

- 3/4 cup chunky salsa (or pico de gallo)
- 1 can (15.5 oz) low-sodium black beans (drained and rinsed)
- 2 cups shredded reduced-fat Colby & Monterey Jack cheese
- 2 tablespoons fresh cilantro (finely chopped)
- 4 8 inch flour tortillas
- 1/2 teaspoon extra virgin olive oil

Directions

1. Using a small-hole strainer, drain liquid from salsa; discard liquid.
2. Transfer leftover tomato mixture to a medium bowl.
3. Mix in black beans, cheese, and cilantro until combined.
4. Divide black bean mixture evenly over half of each tortilla (about 1/2 cup each).
5. Fold tortillas in half.
6. Heat large griddle or skillet over medium-high heat.
7. Brush with oil.
8. Place filled tortillas on a griddle.
9. Cook, carefully flipping once, until tortillas are gold brown and crisp and cheese filling melts, about 5 minutes.
10. Cut quesadillas into wedges.

Source:

The Best of La Cocina GOYA: Healthy, Tasty, Affordable Latin Cooking/ Lo Mejor de la Cocina GOYA: Cocina Latina Saludable, Rica y Económica
GOYA Foods

Nutrition Information

Nutrients	Amount
**Total Calories**	**160**
**Total Fat**	**5 g**
Saturated Fat	2 g
Cholesterol	5 mg
**Sodium**	**490 mg**
**Carbohydrates**	**25 g**
Dietary Fiber	4 g
Total Sugars	1 g
Added Sugars included	0 g
**Protein**	**7 g**
Vitamin D	0 mcg
Calcium	96 mg
Iron	2 mg
Potassium	287 mg

Nutrients will display if the data is available

Please note: nutrient values are subject to change as data is updated

MyPlate Food Groups

Vegetables	1/4 cups
Grains	1 ounce

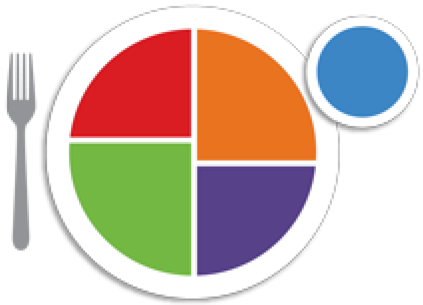

Visit MyPlate.gov

Broccoli Potato Soup

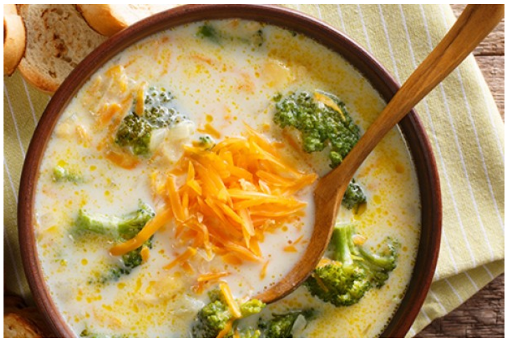

Makes: 4 servings

A tasty and made-from-scratch soup that comes together quickly with the help of instant mashed potatoes and powdered milk.

Ingredients

- 4 cups broccoli, chopped
- 1 small onion, chopped
- 4 cups chicken or vegetable broth, low-sodium
- 1 cup non-fat evaporated milk
- 1/2 cup instant mashed potatoes, prepare with water for 1 cup potatoes (or 1 cup leftover mashed potatoes.)
- salt and pepper (to taste, optional)
- 1/4 cup cheddar cheese, shredded

Directions

1. Wash hands with soap and water.
2. Combine broccoli, onion, and broth in large sauce pan.
3. Bring to a boil.
4. Reduce heat. Cover and simmer about 10 minutes or until vegetables are tender.
5. Add milk to soup. Slowly stir in potatoes.
6. Cook, stirring constantly, until bubbly and thickened.
7. Season with salt and pepper; stir in a little more milk or water if soup starts to become too thick.
8. Ladle into serving bowls.
9. Sprinkle about 1 tablespoon cheese over each serving.

Source:

Don’t Play With Your Food: Fall and Winter Cookbook
Arizona Nutrition Network

Nutrition Information

Serving Size: 1/4 of recipe

Nutrients	Amount
**Total Calories**	**178**
**Total Fat**	**4 g**
Saturated Fat	2 g
Cholesterol	10 mg
**Sodium**	**225 mg**
**Carbohydrates**	**23 g**
Dietary Fiber	3 g
Total Sugars	10 g
Added Sugars included	0 g
**Protein**	**15 g**
Vitamin D	1 mcg
Calcium	295 mg
Iron	2 mg
Potassium	806 mg

Nutrients will display if the data is available

Please note: nutrient values are subject to change as data is updated

MyPlate Food Groups

Vegetables	1 1/2 cups
Dairy	3/4 cups

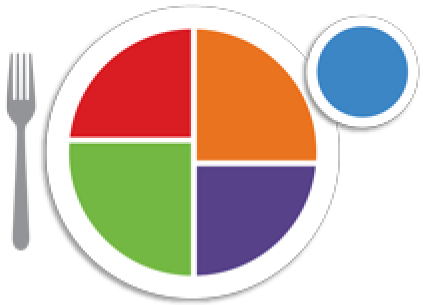

Visit MyPlate.gov

Marinated Beef

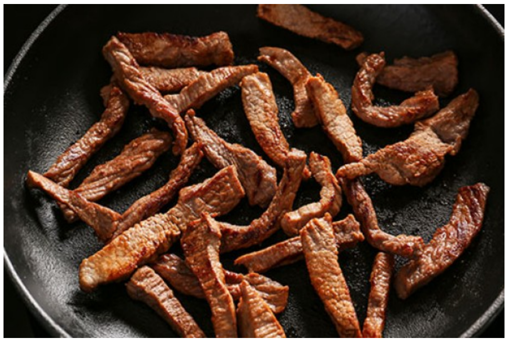

Makes: 4 servings

Tangy, marinated beef is full of flavor and pairs well with roasted potatoes, steamed broccoli, and fresh, sliced tomatoes.

Ingredients

- 12 ounces beef round steak
- 1 garlic clove
- 2 tablespoons lemon juice
- 4 tablespoons vegetable oil, divided (or cooking oil of choice)
- 1/2 teaspoon salt
- 1/2 teaspoon black pepper

Directions

1. Wash hands with soap and water.
2. Using a cutting board and sharp knife, cut round steak across the grain into thin strips about 1/2 inch wide and 2 to 3 inches long.
3. In a medium glass mixing bowl, combine garlic, lemon juice, 2 tablespoons of vegetable oil, salt, and pepper.
4. Add beef strips and stir to coat with the oil mixture (marinade sauce). Cover bowl with plastic wrap and refrigerate for about 2 hours.
5. In a medium skillet over medium-high heat, heat 2 tablespoons of the oil for 1-2 minutes until hot.
6. Drain marinade from beef. Put beef in skillet, stir and cook for 5 to 7 minutes or until meat is thoroughly browned.

Source:

Kids a Cookin'
Kansas Family Nutrition Program

Nutrition Information

Serving Size: 1/4 of recipe

Nutrients	Amount
**Total Calories**	**219**
**Total Fat**	**18 g**
Saturated Fat	3 g
Cholesterol	42 mg
**Sodium**	**310 mg**
**Carbohydrates**	**1 g**
Dietary Fiber	0 g
Total Sugars	0 g
Added Sugars included	0 g
**Protein**	**14 g**
Vitamin D	0 mcg
Calcium	6 mg
Iron	1 mg
Potassium	131 mg

Nutrients will display if the data is available

Please note: nutrient values are subject to change as data is updated

MyPlate Food Groups

Protein Foods 2 ounces

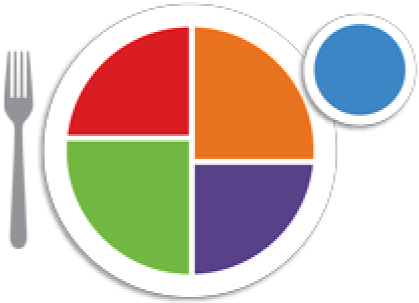

[Visit MyPlate.gov]

Overnight Oatmeal with Berries

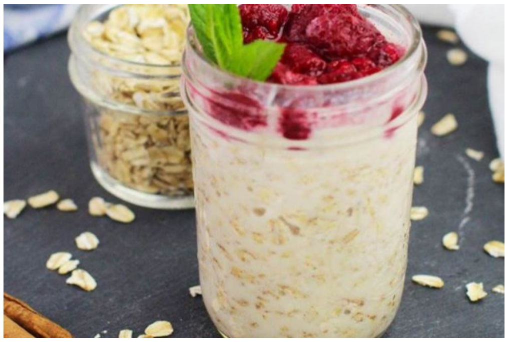

Makes: 1 Serving
Preparation Time: 15 minutes

A mixture of milk, yogurt, and raspberries combine with dry oats to become a smooth make-ahead breakfast. Just mix and refrigerate overnight for the next day.

Ingredients

- 1/2 cup low-fat milk (or less for thicker oatmeal)
- 1/4 cup Greek yogurt, fat-free
- 2 teaspoons honey
- 1/4 teaspoon cinnamon
- 1/4 teaspoon vanilla extract
- 1/2 cup uncooked rolled oats
- 1/4 cup raspberries, frozen

Directions

1. Combine milk, Greek yogurt, sugar, cinnamon, and vanilla extract in a container or jar with a lid.
2. Add oats and mix well.
3. Gently fold in raspberries.
4. Cover and refrigerate 8 hours to overnight.
5. Enjoy cold or heat as desired.

Source:

USDA Center for Nutrition Policy and Promotion

Nutrition Information

Serving Size: 1 serving

Nutrients	Amount
**Total Calories**	**311**
**Total Fat**	**4 g**
Saturated Fat	1 g
Cholesterol	7 mg
**Sodium**	**86 mg**
**Carbohydrates**	**53 g**
Dietary Fiber	9 g
Total Sugars	21 g
Added Sugars included	11 g
**Protein**	**17 g**
Vitamin D	1 mcg
Calcium	268 mg
Iron	2 mg
Potassium	513 mg

Nutrients will display if the data is available

Please note: nutrient values are subject to change as data is updated

MyPlate Food Groups

Fruits	1/4 cups
Grains	1 1/2 ounces
Dairy	3/4 cups

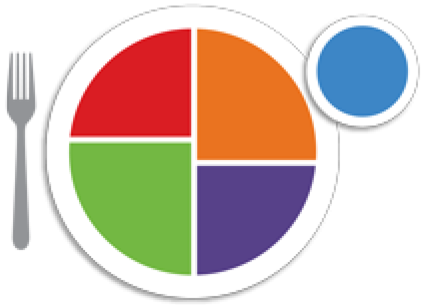

Visit MyPlate.gov

Quinoa and Black Bean Salad

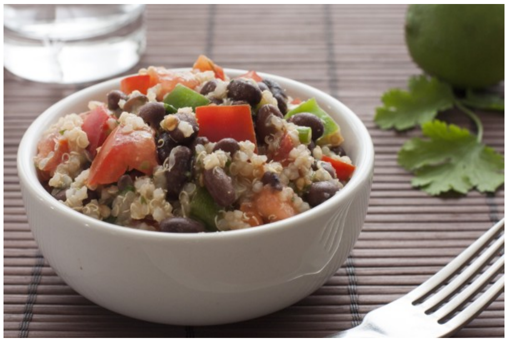

Makes: 6 servings

Quinoa (pronounced "KEEN-wah") is a whole grain with origins in South America. It is combined in this recipe with black beans, savory vegetables, and spices for a cold salad that is light and refreshing. This salad is high in protein and can be served as a main or side dish.

Ingredients

- 1/2 cup quinoa (dry)
- 1 1/2 cups water
- 1 1/2 tablespoons olive oil
- 3 teaspoons lime juice
- 1/4 teaspoon cumin
- 1/4 teaspoon coriander (ground, dried cilantro seeds)
- 2 tablespoons cilantro (chopped)
- 2 scallions (medium, minced)
- 1 can black beans, low-sodium (15.5 ounce can, rinsed and drained)
- 2 cups tomato (chopped)
- 1 red bell pepper (medium, chopped)
- 1 green bell pepper (medium, chopped)
- 2 green chiles (minced, to taste)
- black pepper (to taste)

Directions

1. Wash hands with soap and water.
2. Rinse the quinoa in cold water. Boil water in a saucepan, and then add the quinoa.
3. Return to boil, and then simmer until the water is absorbed, 10 to 15 minutes. Cool for 15 minutes.
4. While quinoa is cooking, mix olive oil, lime juice, cumin, coriander, chopped cilantro, and scallions in a small bowl, and set aside.
5. Combine chopped vegetables with the black beans in a large bowl, and set aside.
6. Once quinoa has cooled, combine all ingredients and mix well.
7. Cover and refrigerate until ready to serve.

Source:

Delicious Heart-Healthy Latino Recipes/ Platillos Latinos Sabrosos y Saludables

Nutrition Information

Serving Size: 1 cup (254g)

Nutrients	Amount
**Total Calories**	**173**
**Total Fat**	**5 g**
Saturated Fat	1 g
Cholesterol	0 mg
**Sodium**	**112 mg**
**Carbohydrates**	**27 g**
Dietary Fiber	8 g
Total Sugars	4 g
Added Sugars included	0 g
**Protein**	**7 g**
Vitamin D	0 mcg
Calcium	50 mg
Iron	3 mg
Potassium	554 mg

Nutrients will display if the data is available

Please note: nutrient values are subject to change as data is updated

MyPlate Food Groups

Vegetables	1 1/4 cups
Grains	1/2 ounces
Protein Foods	1 1/2 ounces

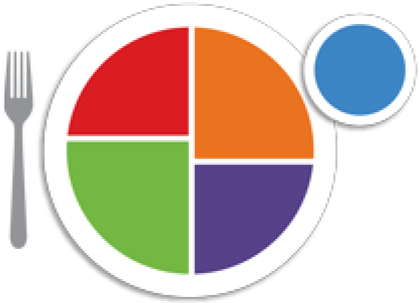

Visit MyPlate.gov

Stuffed Bell Peppers

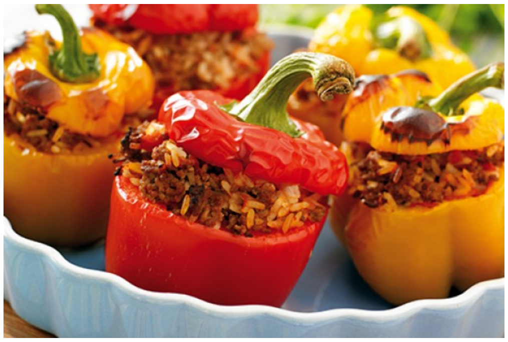

Makes: 5 Servings

Peppers stuffed with a combination of ground beef, brown rice, veggies, and spices create a filling dish. Try using red, green, yellow, and orange bell peppers for a colorful meal. Enjoy with a side of low-fat cottage cheese and canned pineapple.

Ingredients

- 5 bell peppers (red, orange, yellow, or green)
- 1 pound 90% lean ground beef
- 3/4 cup brown rice
- 1/2 can (14.5 ounces) low-sodium diced tomatoes
- 3 tablespoons lemon juice
- 1/4 teaspoon cinnamon
- 1/4 teaspoon allspice
- 1/4 teaspoon ground black pepper

Directions

1. Wash hands with soap and watr.
2. Cut a circular hole in the tops of the bell peppers. Remove seeds and membrane and set aside.
3. In a large bowl, combine remaining ingredients and stir until completely mixed. Fill the bell peppers with meat mixture.
4. Place the stuffed bell peppers in large stock pot on stove, with the tops facing up. Add 1-inch water to bottom of pot and cover.
5. Place heat on medium, keeping covered for 30 to 40 minutes until rice is done. Serve.

Source:

Simple Healthy Recipes
Oklahoma Nutrition Information and Education
ONIE Project

Nutrition Information

Serving Size: 1 stuffed pepper, 1/5 of recipe

Nutrients	Amount
**Total Calories**	**277**
**Total Fat**	**8 g**
Saturated Fat	3 g
Cholesterol	57 mg
**Sodium**	**66 mg**
**Carbohydrates**	**29 g**
Dietary Fiber	4 g
Total Sugars	4 g
Added Sugars included	0 g
**Protein**	**21 g**
Vitamin D	0 mcg
Calcium	48 mg
Iron	3 mg
Potassium	588 mg

Nutrients will display if the data is available

Please note: nutrient values are subject to change as data is updated

MyPlate Food Groups

Vegetables	1 1/4 cups
Grains	1 ounces
Protein Foods	2 1/2 ounces

Visit MyPlate.gov

Julia’s Sautéed & Steamed Collards

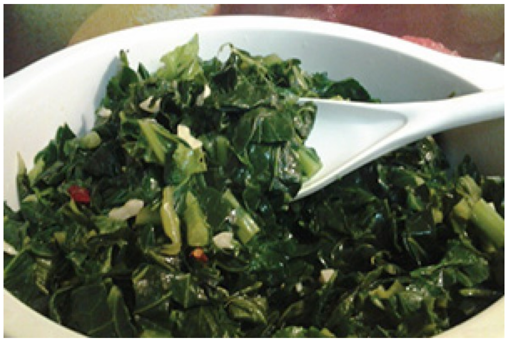

Makes: 8 Servings

Collards are an all-time favorite side to any meal. This recipe starts with tender collards and adds a hint of “heat” from hot pepper flakes. Try this easy and tasty recipe at your next family gathering.
- Julia, CNPP

Ingredients

- 16 cups chopped collards (small leaves)
- 2 tablespoons olive oil
- 4 large garlic cloves
- 1/2 teaspoon hot pepper flakes
- 1 teaspoon salt-free seasoning

Directions

1. Cut bottom stems from collards.
2. Wash and cut collards into bite-sized pieces. Place in colander to drain.
3. Chop garlic cloves into small pieces.
4. Place olive oil into large skillet. Place on high heat to start.
5. Place collards in skillet. Keep on high heat, turning collards as they saute, about 1 minute in the uncovered skillet. (You will not need to add water because of the water already on the washed collards.)
6. Add garlic cloves and red pepper flakes.
7. Place cover on the skillet to allow collards to steam until they are half their original volume.
8. Turn heat to low and continue steaming until the collards are tender.

Source:

USDA Center for Nutrition Policy and Promotion
Recipe submitted by Julia

Nutrition Information

Serving Size: 1 portion of Julia's Sauteed & Steamed Collards

Nutrients	Amount
**Total Calories**	**54**
**Total Fat**	**4 g**
Saturated Fat	1 g
Cholesterol	0 mg
**Sodium**	**15 mg**
**Carbohydrates**	**5 g**
Dietary Fiber	3 g
Total Sugars	0 g
Added Sugars included	0 g
**Protein**	**2 g**
Vitamin D	0 mcg
Calcium	111 mg
Iron	0 mg
Potassium	135 mg

Nutrients will display if the data is available

Please note: nutrient values are subject to change as data is updated

MyPlate Food Groups

Vegetables 1 cup

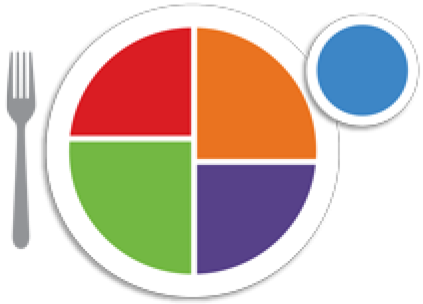

Visit MyPlate.gov

Peanutty Stew

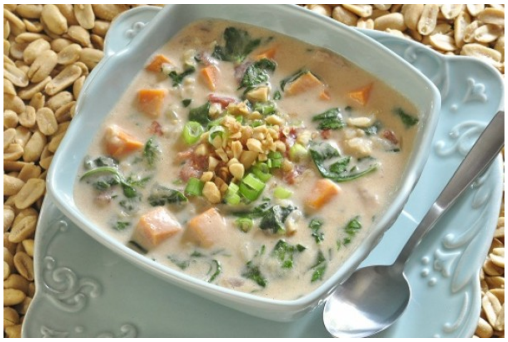

Makes: 8 servings

A spicy and savory one pot dish that the whole family will enjoy. Add some cayenne pepper for an extra kick.

Ingredients

- 1 cup instant brown rice
- 2 cups low-sodium chicken broth
- 1/3 tablespoon dehydrated onion, minced
- 1/2 teaspoon garlic powder
- 1/2 teaspoon ground ginger
- red pepper (optional, 2 tablespoons)
- 1 medium sweet potato, peeled and diced (about 2 cups)
- 1 can (14.5 ounces) low-sodium tomatoes, not drained
- salt (optional, 1/2 teaspoon)
- 8 tablespoons low-fat creamy peanut butter (1/2 cup)
- 1 1/4 cups fat-free (skim) milk
- 3 cups baby spinach, coarsely chopped
- 1/4 cup peanuts, chopped
- green onion (optional, thinly sliced for garnish)

Directions

1. Wash hands with soap and water.
2. Combine first 9 ingredients in a soup pot. Bring to boil, then turn down to medium low, cover and simmer for 10 minutes.
3. Stir in peanut butter and milk. Return to gentle simmer and cook uncovered for 5 minutes.
4. Stir in spinach and cook until wilted, 2 to 3 minutes. Remove from heat.
5. Ladle stew into bowls and top with peanuts and green onion, if desired.

Source:

Food Hero
Oregon State University Cooperative Extension Service

Nutrition Information

Serving Size: 1 cup

**Nutrients**	**Amount**
**Total Calories**	**263**
**Total Fat**	**10 g**
Saturated Fat	2 g
Cholesterol	1 mg
**Sodium**	**170 mg**
**Carbohydrates**	**35 g**
Dietary Fiber	4 g
Total Sugars	6 g
Added Sugars included	1 g
**Protein**	**12 g**
Vitamin D	0 mcg
Calcium	101 mg
Iron	2 mg
Potassium	554 mg

Nutrients will display if the data is available

Please note: nutrient values are subject to change as data is updated

MyPlate Food Groups

Vegetables	1/2 cups
Grains	1 ounces
Protein Foods	1 1/2 ounces
Dairy	1/4 cups

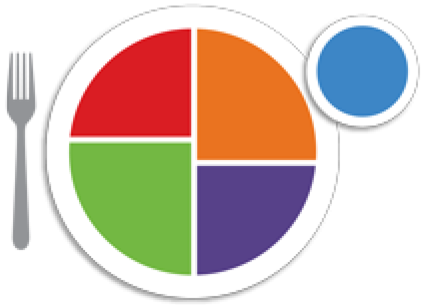

Visit MyPlate.gov

Bell Pepper and Apple Coleslaw

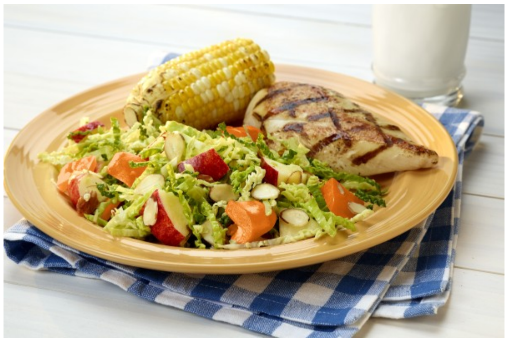

Makes: 4 Servings
Preparation Time: 20 minutes

Apples and bell peppers liven up this coleslaw made with an easy vinegar mustard dressing. Add more color with red, orange or yellow bell peppers. Use your favorite sweet and crisp apple, such as Gala or Fuji.

Ingredients

For the Dressing:

- 1 tablespoon vegetable oil
- 1/4 cup apple juice
- 2 tablespoons cider vinegar
- 2 teaspoons Dijon mustard
- 1/4 teaspoon salt
- 1 dash black pepper

For the Salad:

- 1 small head green or Napa cabbage
- 1 orange or red bell pepper
- 1 Gala or Fuji apple
- 1/4 cup sliced almonds

Directions

1. Whisk together dressing ingredients.
2. Shred or thinly slice cabbage.
3. Cut apple and pepper into small chunks.
4. Place cabbage, apples, pepper and almonds in large bowl.
5. Drizzle with dressing and toss.

Source:

Produce for Better Health Foundation

Nutrition Information

Nutrients	Amount
**Total Calories**	**154**
**Total Fat**	**9 g**
Saturated Fat	1 g
Cholesterol	0 mg
**Sodium**	**229 mg**
**Carbohydrates**	**17 g**
Dietary Fiber	5 g
Total Sugars	9 g
Added Sugars included	0 g
**Protein**	**4 g**
Vitamin D	0 mcg
Calcium	83 mg
Iron	1 mg
Potassium	386 mg

Nutrients will display if the data is available

Please note: nutrient values are subject to change as data is updated

MyPlate Food Groups

Fruits	1/2 cups
Vegetables	1 1/2 cups
Protein Foods	1/2 ounces

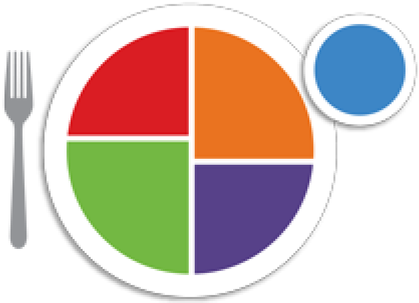

Visit MyPlate.gov

Fabulous Fig Bars

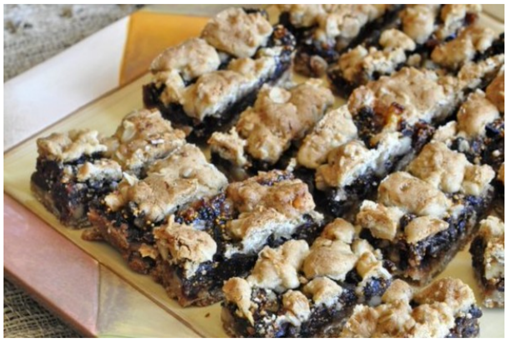

Makes: 24 Bars
Preparation Time: 45 minutes
Cook Time: 30 minutes

Fig bars are a great on-the-go snack. The sweet, nutty flavors in these bars are sure to be a hit with all ages.

Ingredients

- 2 cups dried figs (chopped, 16 ounces)
- 1/2 cup walnuts (chopped)
- 1/3 cup sugar
- 1/4 cup orange juice (juice from 1/2 orange)
- 2 tablespoons hot water
- 1/2 cup margarine (softened, or butter)
- 1 cup packed brown sugar
- 1 large egg
- 1 1/2 cups all-purpose flour
- 1/2 teaspoon baking soda
- 1 1/4 cups old fashioned rolled oats

Directions

1. Wash hands with soap and water.
2. Preheat oven to 350 °F. Lightly grease a 9x13 inch baking pan.
3. Combine figs, walnuts, sugar, orange juice, and hot water in a mixing bowl and set aside.
4. Mix together margarine or butter and brown sugar until creamy. Add egg and mix until smooth.
5. Mix flour and baking soda. Stir into egg mixture. Blend in oats to make soft dough.
6. Reserve 1 cup of dough for topping. With floured fingertips, press the remaining dough into a thin layer on the bottom of the baking pan.
7. Spread fig mixture evenly over the dough. Crumble reserved dough over top, allowing fig mixture to show.
8. Bake 30 minutes or until golden brown. Cool completely in baking pan. Cut into 24 bars (about 2 1/2 x 2 inches).

Source:

Food Hero
Oregon State University Cooperative Extension Service

Nutrition Information

Serving Size: 1 bar

Nutrients	Amount
**Total Calories**	**185**
**Total Fat**	**6 g**
Saturated Fat	1 g
Cholesterol	8 mg
**Sodium**	**65 mg**
**Carbohydrates**	**32 g**
Dietary Fiber	2 g
Total Sugars	20 g
Added Sugars included	11 g
**Protein**	**3 g**
Vitamin D	0 mcg
Calcium	42 mg
Iron	1 mg
Potassium	169 mg

Nutrients will display if the data is available

Please note: nutrient values are subject to change as data is updated

MyPlate Food Groups

Fruits 1/4 cups
Grains 1/2 ounces

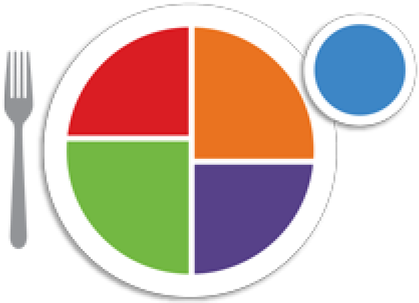

Visit MyPlate.gov

Slow Cooker Lentil Soup

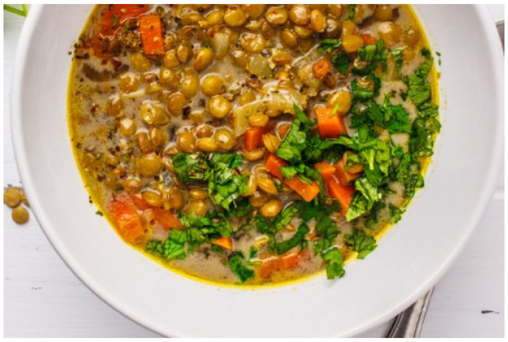

Makes: 6 servings

Quick to make but rich and delicious flavors of carrots, onion, and celery flavor in this slow cooker lentil soup. Add your favorite spices if you want to add a little kick.

Ingredients

- 6 cups water
- 1/4 cup fresh parsley, chopped (or 2 tablespoons dried)
- 2 teaspoons beef bouillon (or 2 cubes beef bouillon)
- 1 1/2 cups dry lentils
- 2 medium carrots, sliced
- 1 medium onion, chopped
- 2 medium celery stalks, sliced

Directions

1. Wash hands with soap and water.
2. Mix all ingredients together in slow cooker.
3. Cook on low for 8 to 10 hours or high for 4 to 5 hours.
4. Serve hot with crackers or bread.

Source:

Montana State University Extension Service
Nutrition Education Program

Nutrition Information

Serving Size: 1/6 of recipe

Nutrients	Amount
**Total Calories**	**177**
**Total Fat**	**1 g**
Saturated Fat	0 g
Cholesterol	0 mg
**Sodium**	**186 mg**
**Carbohydrates**	**32 g**
Dietary Fiber	12 g
Total Sugars	5 g
Added Sugars included	0 g
**Protein**	**13 g**
Vitamin D	0 mcg
Calcium	53 mg
Iron	5 mg
Potassium	642 mg

Nutrients will display if the data is available

Please note: nutrient values are subject to change as data is updated

MyPlate Food Groups

Vegetables	1 cups
Protein Foods	3 ounces

Visit MyPlate.gov

Argentinean Grilled Steak with Salsa Criolla

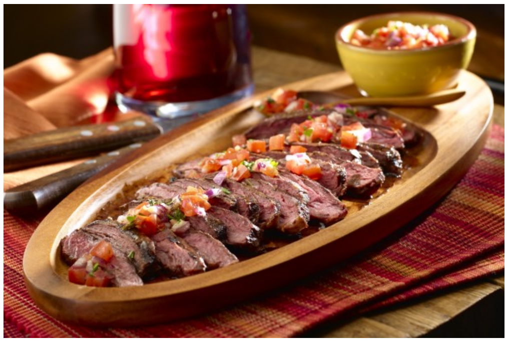

Makes: 4 Servings
Preparation Time: 25 minutes

Tonight, try a recipe from South America! Enjoy these Argentinean-style steaks with a homemade Criolla Sauce, packed with flavor.

Ingredients

For the sauce:

- 1 large, ripe tomato (cored, seeded, and finely chopped, about 1/2 cup)
- 1/4 small red onion (finely chopped, about 1/4 cup)
- 2 tablespoons fresh parsley (finely chopped)
- 2 teaspoons extra virgin olive oil
- 2 teaspoons red wine vinegar
- 1/2 teaspoon minced garlic
- 1/4 teaspoon oregano leaf
- 1/8 teaspoon low-sodium adobo seasoning
- 1/8 teaspoon crushed red pepper

For the steak:

- 1 pound skirt steak
- 1/8 teaspoon low-sodium adobo seasoning

Directions

For the sauce:

1. In a small bowl, mix together tomato, onions, parsley, olive oil, vinegar, garlic, oregano, low-sodium adobo seasoning and crushed red pepper.
2. Cover and refrigerate for at least 1 hour or up until 48 hours.

For the steak:

1. Heat grill to medium-high heat. Sprinkle steak on both sides with low-sodium adobo seasoning. Place steak on hot, greased grill grates.
2. Cook, flipping once, until steak is well browned on both sides and cooked to 145 °F (about 6 minutes for

medium-rare).
3. Let rest for 5 minutes. Thinly slice steak.
4. Divide steak evenly among serving plates. Top with reserved Salsa Criolla.

Source:

The Best of La Cocina GOYA: Healthy, Tasty, Affordable Latin Cooking/ Lo Mejor de la Cocina GOYA: Cocina Latina Saludable, Rica y Económica
GOYA Foods

Nutrition Information

Nutrients	Amount
**Total Calories**	**220**
**Total Fat**	**11 g**
Saturated Fat	4 g
Cholesterol	70 mg
**Sodium**	**80 mg**
**Carbohydrates**	**5 g**
Dietary Fiber	1 g
Total Sugars	3 g
Added Sugars included	0 g
**Protein**	**25 g**
Vitamin D	0 mcg
Calcium	29 mg
Iron	2 mg
Potassium	433 mg

Nutrients will display if the data is available

Please note: nutrient values are subject to change as data is updated

MyPlate Food Groups

Vegetables	1/4 cups
Protein Foods	2 1/2 ounces

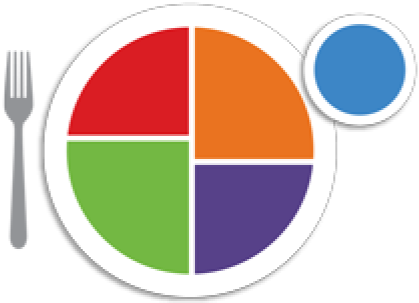

Visit MyPlate.gov
